# AKR1C2 promotes lymph node metastasis in cervical cancer via a non-canonical nuclear function

**DOI:** 10.1038/s41420-026-03287-5

**Published:** 2026-08-13

**Authors:** Fangjie He, Yuan Li, Shuiling Zu, Shimin Huang, Haijun Yang, Fuqiang Wang, Yujiao Tang, Shunhe Lin, Xia Chen

**Affiliations:** 1 Department of Gynecology, Fujian Maternity and Child Health Hospital, College of Clinical Medicine for Obstetrics & Gynecology and Pediatrics, Fujian Medical University, Fuzhou, China; 2 Department of Obstetrics and Gynecology, The First People’s Hospital of Foshan, Foshan, China; 3 Department of Gynecology, Jiangxi Maternal and Child Health Hospital, Nanchang, China; 4 Department of Pathology, Anyang Tumor Hospital, Anyang, China; 5 Department of Pathology, The First People’s Hospital of Foshan, Foshan, China; 6 Department of Obstetrics and Gynecology, The First Affiliated Hospital of Ningbo University, Ningbo, China; 7 Tianfu Jincheng Laboratory, Chengdu, China

**Keywords:** Tumour biomarkers, Proteomics, Metastasis, Predictive markers, Cervical cancer

## Abstract

The presence or absence of lymph node metastasis (LNM) determines the staging and treatment strategy for cervical cancer. However, preoperative diagnosis of LNM remains insufficiently accurate and the underlying mechanisms are not fully understood. This study aimed to identify proteomic biomarkers associated with LNM and investigate the role of AKR1C2 in promoting LNM. Data-independent acquisition proteomic analysis indicated that LNM was significantly associated with extracellular matrix organization. A model with AKR1C2, ARHGAP26 and ATP6AP2 demonstrated optimal sensitivity of 95.8% and specificity of 72.7% (AUC, 0.890; 95% CI, 0.824–0.955; *p* < 0.001), which was confirmed in the validation cohort. Functional assays showed that AKR1C2 promotes migration, invasion and LNM in cervical cancer cells. RNA sequencing further supported its involvement in extracellular matrix reorganization. In vitro and rescue assays showed that SPINT2 mediates the effect of AKR1C2 on migration and invasion. GEPIA database analysis also revealed that SPINT2 is associated with worse prognosis. Furthermore, we found that AKR1C2 directly interacts with H4C13 in the nucleus, regulating SPINT2 expression and consequently influencing migration and invasion ability of cervical cancer cells. In conclusion, a proteomic model based on AKR1C2, ARHGAP26 and ATP6AP2 accurately predicts LNM in cervical cancer and could assist in clinical decision-making between radical surgery and concurrent chemoradiotherapy. AKR1C2-H4C13 interaction regulates SPINT2 expression, ultimately changing extracellular matrix and promoting LNM in cervical cancer.

**Figure:**
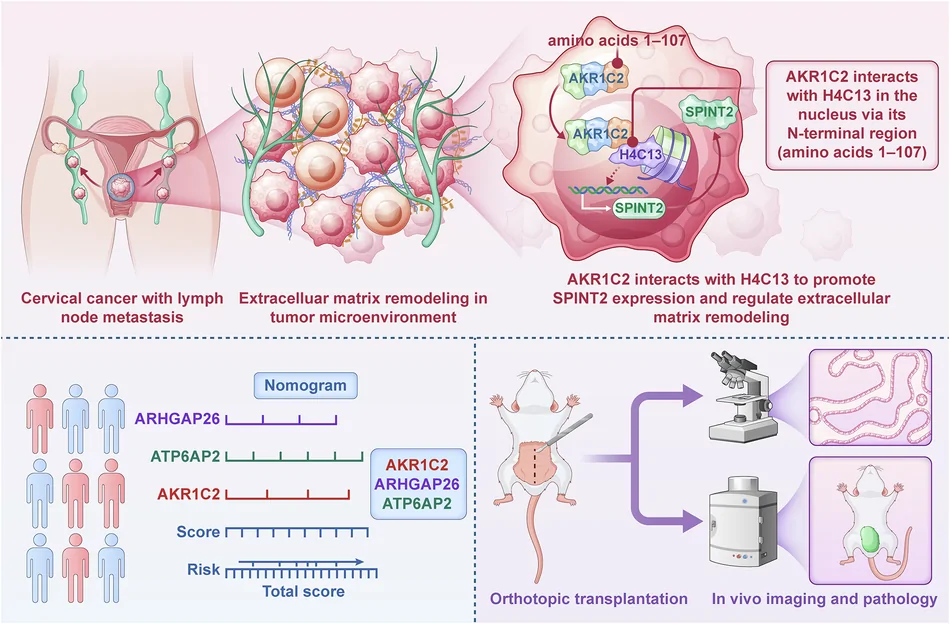

## Introduction

Cervical cancer remains a significant global health concern, with an estimated 661,021 new cases and 348,189 deaths in 2020, ranking as the fourth most common cancer in terms of both incidence and mortality in women [1, 2]. Currently, concurrent chemoradiotherapy is the primary treatment for patients with stage IIB-IVA cervical cancer, while radical surgery or concurrent chemoradiotherapy is recommended for those with stage IA2-IIA2 cervical cancer [3].

In clinical practice, surgery is preferred for patients wishing to preserve fertility or ovarian function, as radiotherapy can lead to ovarian failure. However, some patients with stage IA2-IIA2 cervical cancer are found to have lymph node metastasis (LNM) upon postoperative pathological examination. These patients require adjuvant radiotherapy with or without chemotherapy to reduce the risk of tumor metastasis and recurrence [3]. Unfortunately, unnecessary surgery followed by adjuvant radiotherapy can result in severe comorbidities, including gastrointestinal complications, lymphedema, lymphocysts and genitourinary complications [4]. Therefore, preoperative determination of LNM status is crucial to avoid these adverse effects. Additionally, lymph node status has been incorporated into the latest 2018 International Federation of Gynecology and Obstetrics (FIGO) staging system for cervical cancer [5]. In summary, the presence or absence of LNM determines both staging and treatment strategy.

Computed tomography (CT) and magnetic resonance imaging (MRI) are commonly used to assess LNM status, but exhibit limited sensitivity (generally <65%) and moderate specificity [6–8]. The low sensitivity of these imaging examinations leads to a high rate of missed LNM diagnoses and over 35% of surgical patients require additional adjuvant radiotherapy. In a previous study, we improved the sensitivity to 95.2% using preoperative MRI parameters; however, specificity and accuracy were only 34.2% and 64.7%, respectively [9]. Several pathological risk factors, including stromal invasion, lymphovascular space invasion (LVSI), parametrial involvement (PMI), and uterine corpus invasion, have been shown to predict LNM [10–12]. However, these factors are identified through postoperative pathological examinations and thus can’t be used for preoperative diagnosis of LNM.

Tumor-relevant proteins may be useful for evaluating LNM status [13, 14]. Some previous studies have identified serum microRNAs or SCC-Ag as potential biomarkers for LNM. However, these studies showed low area under the receiver operating characteristic curve (AUC) [15–17]. Several studies have also identified genes associated with LNM from The Cancer Genome Atlas (TCGA) database. However, these studies either showed low AUC or didn’t further validate the predictive performance (e.g., accuracy, sensitivity and specificity) of these genes [18–20]. Therefore, gene-based models have obvious limitations in the diagnosis of LNM. Currently, protein detection can be performed on preoperative biopsies for cervical cancer. However, few studies have identified valuable biomarkers at the protein level. Therefore, a proteomic-based approach holds promise for preoperative diagnosis of LNM.

Aldo-keto reductase family 1 member C2 (AKR1C2) was identified based on proteomics from cervical cancer patients with LNM in the current study. Aldo-keto reductase belongs to the oxidoreductases superfamily. AKR1C2 is a member of the AKR1 family, which is located on human chromosome 10, with a total of 9 exons and 10 transcripts. This gene encodes a 37 kDa protein that is mostly found in the cytoplasm. Human liver, stomach and bladder tissue exhibit high levels of AKR1C2 protein abundance [21].

The aim of this study was to establish a model for preoperative diagnosis of LNM based on proteomics in patients with stage IB1-IIA2 cervical cancer. Furthermore, the function and mechanism of AKR1C2 in promoting LNM were investigated through in vivo and in vitro experiments.

## Results

### Data-independent acquisition (DIA) data analysis and differentially expressed proteins (DEPs) with Immunohistochemistry (IHC)

**Fig. 1:**
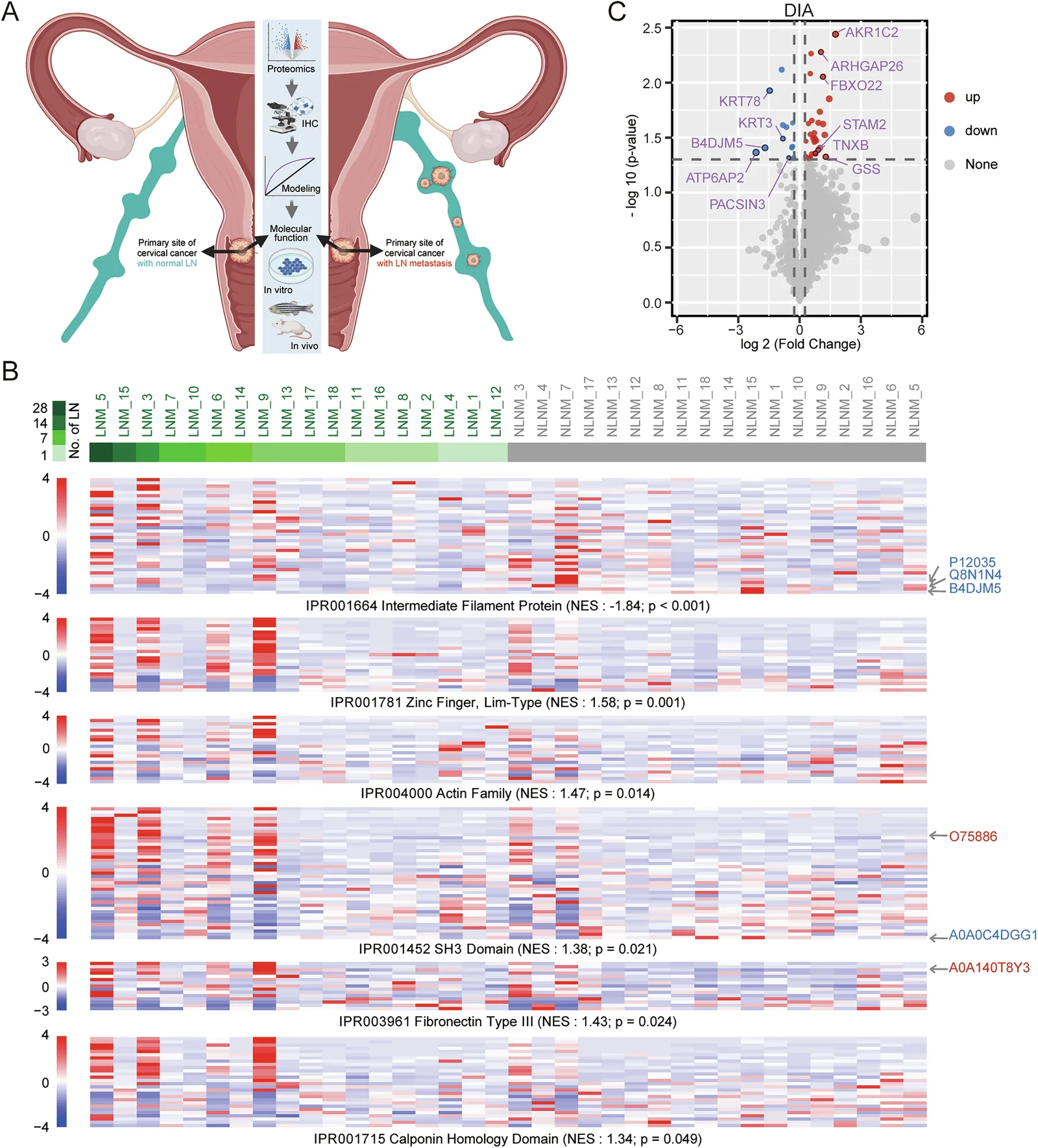
Proteomic analysis of in situ lesions from patients with and without LNM. **A** Experimental flow chart of the study. **B** Heatmaps of GSEA based on IPR terms. Samples were ordered from left to right according to the number of metastatic lymph nodes. Proteins in each IPR term were ordered from top to bottom according to the rank metric score of GSEA. Proteins with a statistically significant difference in expression were labeled on the right side of the heatmaps. **C** Volcano plot of DEPs.

In this study, we first analyzed proteomic changes and DEPs in in situ lesions from patients with LNM and non-LNM (NLNM) using DIA, followed by validation and screening of these DEPs by IHC. We then established and validated a predictive model for LNM in real-world cohorts, and finally verified the ability of a core molecule to regulate cell invasion and metastasis through in vivo and in vitro models (Fig. 1A). Gene set enrichment analysis (GSEA) of DIA data by InterPro (IPR) terms revealed that six protein families or domains were different between in situ samples of LNM and NLNM (Fig. 1B). In total, 42 DEPs were identified (fold-change >1.2 or <0.83, *p* < 0.05) (Fig. 1C). Among these, intermediate filament proteins, including many keratins representing the epithelial nature of the tumor, such as P12035 (KRT3, type II cytoskeletal 3), Q8N1N4 (KRT78, type II cytoskeletal 78) and B4DJM5 (cDNA FLJ61294, highly similar to keratin, type I cytoskeletal 17), showed decreased expression trends in in situ lesions from LNM cases (Fig. 1B, C). Other protein families or domains regulating the cytoskeleton (actin family, zinc finger, calponin homology and SH3 domains) and extracellular matrix organization (fibronectin type III) showed increased expression trends in in situ lesions of LNM patients (Fig. 1B, C).

To further explore the ECM remodeling signature at the protein level, we examined the 42 DEPs identified by DIA-MS and screened for ECM-associated proteins. Among the DEPs, four ECM-related proteins were identified: Tenascin-X (TNXB) was significantly upregulated in LNM samples (log2FC = +0.766, *p* = 0.044), while three keratins—KRT3 (log2FC = −0.818, *p* = 0.032), KRT17 (log2FC = −1.691, *p* = 0.039), and KRT78 (log2FC = −1.465, *p* = 0.012)—were significantly downregulated (Supplementary file). Heatmap analysis revealed distinct expression patterns of these ECM-related proteins between LNM and NLNM groups (Supplementary Fig. S2A). Spearman correlation analysis showed that AKR1C2 abundance was positively correlated trend with KRT78 (*r* = 0.304, *p* = 0.072) in the MS dataset (Supplementary Fig. S2B). These MS-based findings independently support the involvement of ECM remodeling in LNM at the protein level, consistent with our transcriptomic and functional results.

**Fig. 2:**
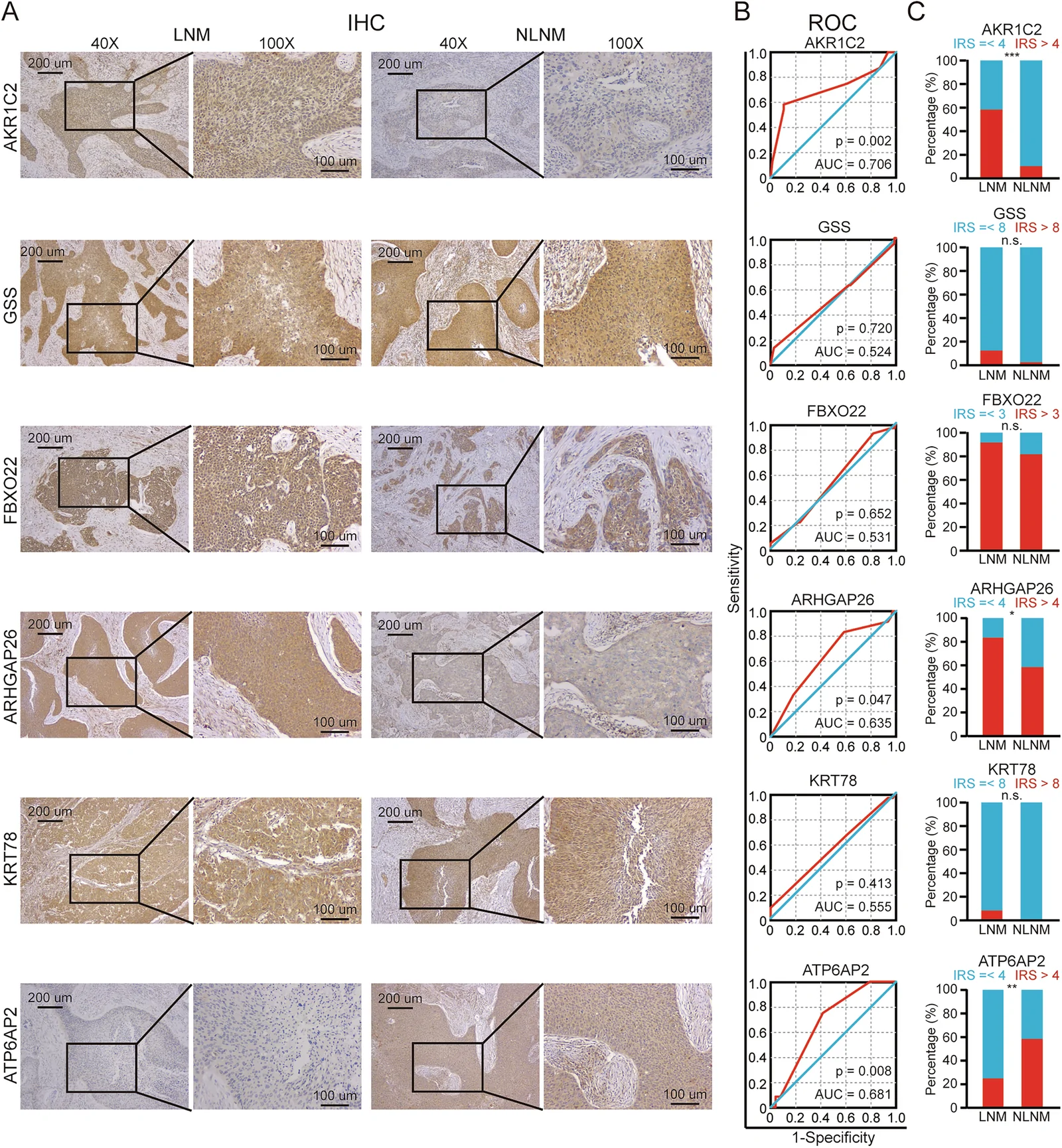
IHC validation and LNM prediction of each DEPs. **A** IHC for AKR1C2, GSS, FBXO22, ARHGAP26, KRT78 and ATP6AP2. **B** ROC curves based on IRS of AKR1C2, GSS, FBXO22, ARHGAP26, KRT78 and ATP6AP2. The positive and negative expression was divided according to the cut-off value. **C** Cumulative bar charts represented the proportion of patients with different IRS in LNM and NLNM subgroups. Cut-off values of IRS of different proteins were based on (**B**). *p* values in (**C**) were calculations of chi-squared test. *p* values: *p < 0.05, **p < 0.01, ***p < 0.001, ****p < 0.001, ns not significantly different.

Among the DEPs, 10 proteins were identified with a fold-change >2.0 (Supplementary Table S3). Excluding four proteins for which commercial antibodies were unavailable, six proteins including AKR1C2, GSS, FBXO22, ARHGAP26, KRT78 and ATP6AP2 were validated by IHC. AKR1C2 and ARHGAP26 were localized in the cytoplasm and nucleus, while GSS, FBXO22, KRT78 and ATP6AP2 were localized in the cytoplasm (Fig. 2A). Based on the immunoreactive score (IRS), receiver operating characteristic (ROC) curves and corresponding cut-off values of these six proteins in predicting LNM are shown in Fig. 2B, C. Patients with LNM had a significantly higher rate of positive staining for AKR1C2 (58.3% vs. 10.4%, *p* < 0.001) and ARHGAP26 (83.3% vs. 58.4%, *p* = 0.030). Patients without LNM had a significantly higher rate of positive staining for ATP6AP2 (25.0% vs. 58.4%, *p* = 0.005). However, the rate of positive staining for GSS, FBXO22 and KRT78 was not significantly different between the two groups (*p* > 0.05) (Fig. 2C).

### Protein biomarkers for LNM and survival analysis in the training set

In the training set of 101 patients, univariate and multivariate forward stepwise logistic regression analysis were performed to identify independent risk factors for LNM among the six proteins estimated by IHC and based on IRS. The results showed that AKR1C2, ARHGAP26 and ATP6AP2 were independently associated with LNM (Supplementary Table S4). The characteristics of the ROC curve analysis for each protein are shown in the Supplementary Table S5.

**Fig. 3:**
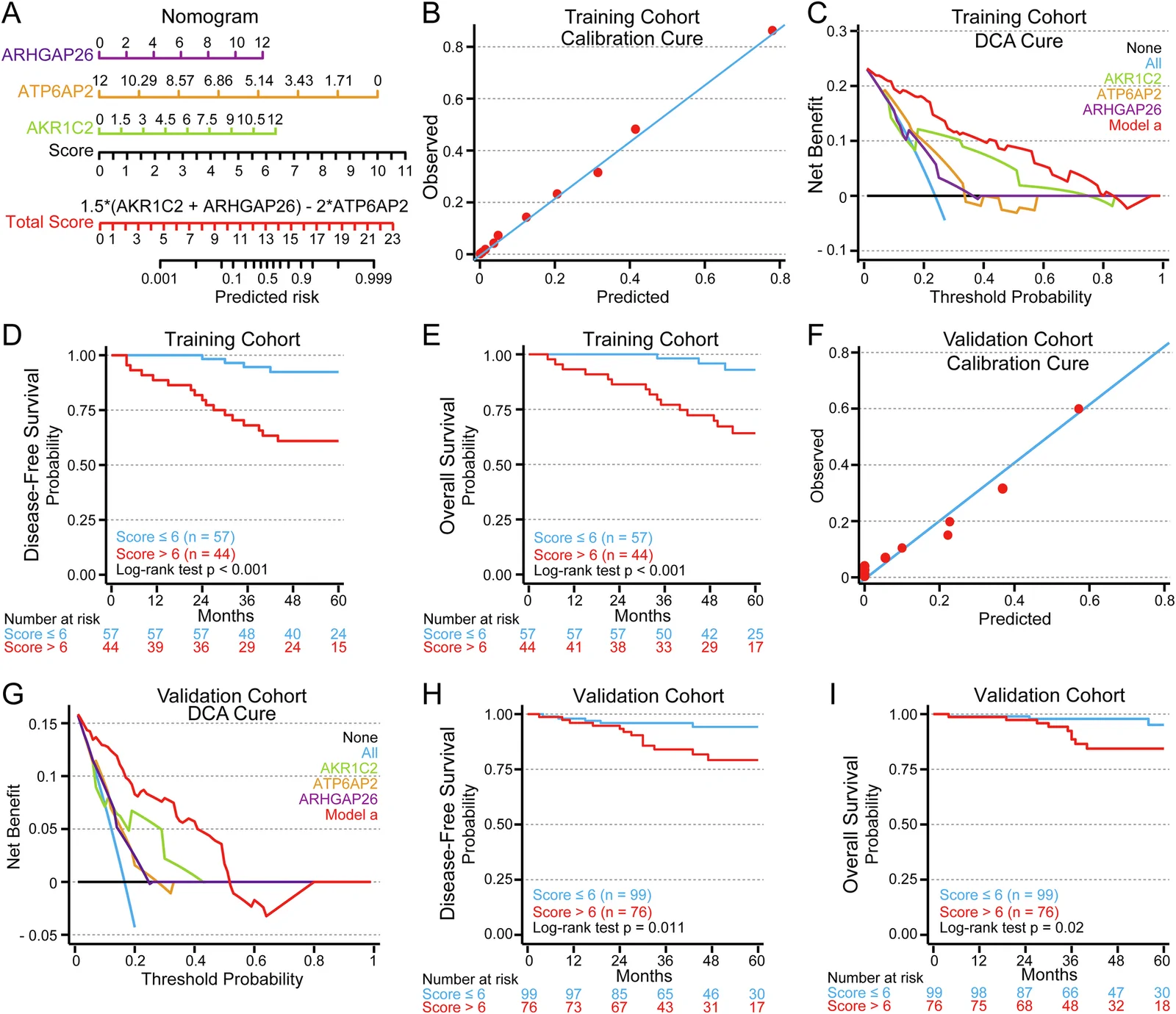
A model for predicting LNM based on DEPs of in situ lesions. **A** The nomogram of Model a for predicting LNM based on the training set. **B** The calibration curve for Model a in the training set. **C** The DCA curve for Model a in the training set. **D**–**E** Kaplan–Meier curves for 5-year DFS and OS, respectively, based on the score of Model a in the training set. **F** The calibration curve for Model a in the validation set. **G** The DCA curve for Model a in the validation set. **H**–**I** Kaplan–Meier curves for 5-year DFS and OS, respectively, based on the score of Model a in the validation set.

According to the parameters of the model, values were assigned as follows: AKR1C2, 1.5x; ARHGAP26, 1.5x; and ATP6AP2, −2.0x. The total score of each patient was calculated, and the optimal cut-off value determined by ROC curve analysis was 6 points. This yielded a sensitivity of 95.8% and specificity of 72.7% (AUC = 0.890; 95% confidence interval [CI], 0.824–0.955; *p* < 0.001) (Supplementary Table S5). Among the 101 patients, 57 (56.4%) were classified as low-risk group (total score ≤6), and only one (1.8%) had LNM. The nomogram, calibration curve and decision curve analysis (DCA) for the model and each protein are shown in Fig. 3A–C and Supplementary Fig. S3A–D, respectively. The combination of three factors significantly improved the predictive value compared to each factor alone (Supplementary Table S5). Furthermore, when preoperative clinical variables were included in the multivariate logistic regression model, tumor diameter and histological type were retained (Supplementary Table S6), and the ROC curve was slightly improved (AUC = 0.920; 95% CI, 0.865–0.975; *p* < 0.001) (Supplementary Table S5). However, due to increased model complexity and reduced practicality, the model with three DEPs was selected for further validation.

Median follow-up for the training set was 52 months. On multivariate analysis controlling for protein biomarkers identified in univariate analysis, AKR1C2 was an independent prognostic factor associated with decreased 5-year DFS (aHR, 3.359, 95% CI, 1.414–7.981, *p* = 0.006) and 5-year OS (aHR, 4.369, 95% CI, 1.733–11.017, *p* = 0.002) (Supplementary Table S7). When preoperative clinical variables were additionally included in the Cox regression model, AKR1C2 remained an independent prognostic factor associated with decreased 5-year DFS (aHR, 2.726, 95% CI, 1.137–6.537, *p* = 0.025) and 5-year OS (aHR, 3.558, 95% CI, 1.397–9.066, *p* = 0.008) (Supplementary Table S8). For the current model in the training set, compared to the low-risk group (total score ≤6), 5-year DFS and OS were significantly shorter for the high-risk group (5-year DFS, 92.3% versus 60.9%, *p* < 0.001; 5-year OS, 93.0% versus 64.2%, *p* < 0.001; Fig. 3D, E). Furthermore, 5-year DFS and OS were significantly shorter for AKR1C2 ( > 4) than for AKR1C2 ( ≤ 4) (Supplementary Fig. S4A–B). However, no difference in 5-year DFS and OS was observed for ARHGAP26 ( > 4 or ≤4) and ATP6AP2 ( ≤ 4 or >4) (Supplementary Fig. S4C-F).

### Proteins for LNM and survival analysis in the validation set

An independent cohort of 175 patients with stage IB1-IIA2 cervical cancer from Anyang Cancer Hospital was used to validate the value of the model. The clinical and pathological characteristics of these 175 patients are summarized in the Supplementary Table S9. Among them, 29 (16.6%) had LNM and 146 (83.4%) did not, as confirmed by pathology review. Protein biomarkers AKR1C2, ARHGAP26 and ATP6AP2 were validated by IHC in this set. Logistic regression analysis confirmed that these 3 proteins were independently associated with LNM (Supplementary Table S10). The characteristics of the ROC curve analysis for each protein are shown in the Supplementary Table S5. Furthermore, ROC curve analysis validated that the model had a sensitivity of 93.1% and a specificity of 66.4% (AUC = 0.861; 95% CI, 0.801–0.920; *p* < 0.001) (Supplementary Table S5). In total, 99 patients (56.6%) were classified as low-risk group (total score ≤6), and only 2 patients (2.0%) had LNM. The calibration curve and DCA for the model and each protein are also shown in Fig. 3F, G and Supplementary Fig. S3E–H, respectively. When preoperative clinical variables were included in the multivariate logistic regression model, the results showed that tumor diameter and histological type weren’t retained in the model. However, AKR1C2, ARHGAP26 and ATP6AP2 remained independent risk factors for LNM (Supplementary Table S11).

Median follow-up for the validation set was 44 months. On multivariate analysis controlling for protein biomarkers identified in univariate analysis, AKR1C2 was an independent prognostic factor associated with decreased 5-year DFS (aHR, 3.122, 95% CI, 1.208–8.068, *p* = 0.019) and 5-year OS (aHR, 4.369, 95% CI, 1.733–11.017, *p* = 0.002) (Supplementary Table S12). When preoperative clinical variables were additionally considered in the Cox regression model, AKR1C2 remained an independent prognostic factor associated with decreased 5-year DFS (aHR, 3.122, 95% CI, 1.208–8.068, *p* = 0.019) and 5-year OS (aHR, 3.558, 95% CI, 1.397–9.066, *p* = 0.008) (Supplementary Table S13). For the current model in the validation set, compared to the low-risk group (total score ≤6), 5-year DFS and OS were significantly shorter for the high-risk group (5-year DFS, 94.2% versus 79.2%, *p* = 0.011; 5-year OS, 95.1% versus 84.4%, *p* = 0.020; Fig. 3H, I). Furthermore, 5-year DFS and OS were significantly shorter for AKR1C2 ( > 4) than for AKR1C2 ( ≤ 4) (Supplementary Fig. S4G–H). However, no difference in 5-year DFS and OS was observed for ARHGAP26 ( > 4 or ≤4) and ATP6AP2 ( ≤ 4 or >4) (Supplementary Fig. S4I-L).

### AKR1C2 changes tumor cells function in cervical cancer

**Fig. 4:**
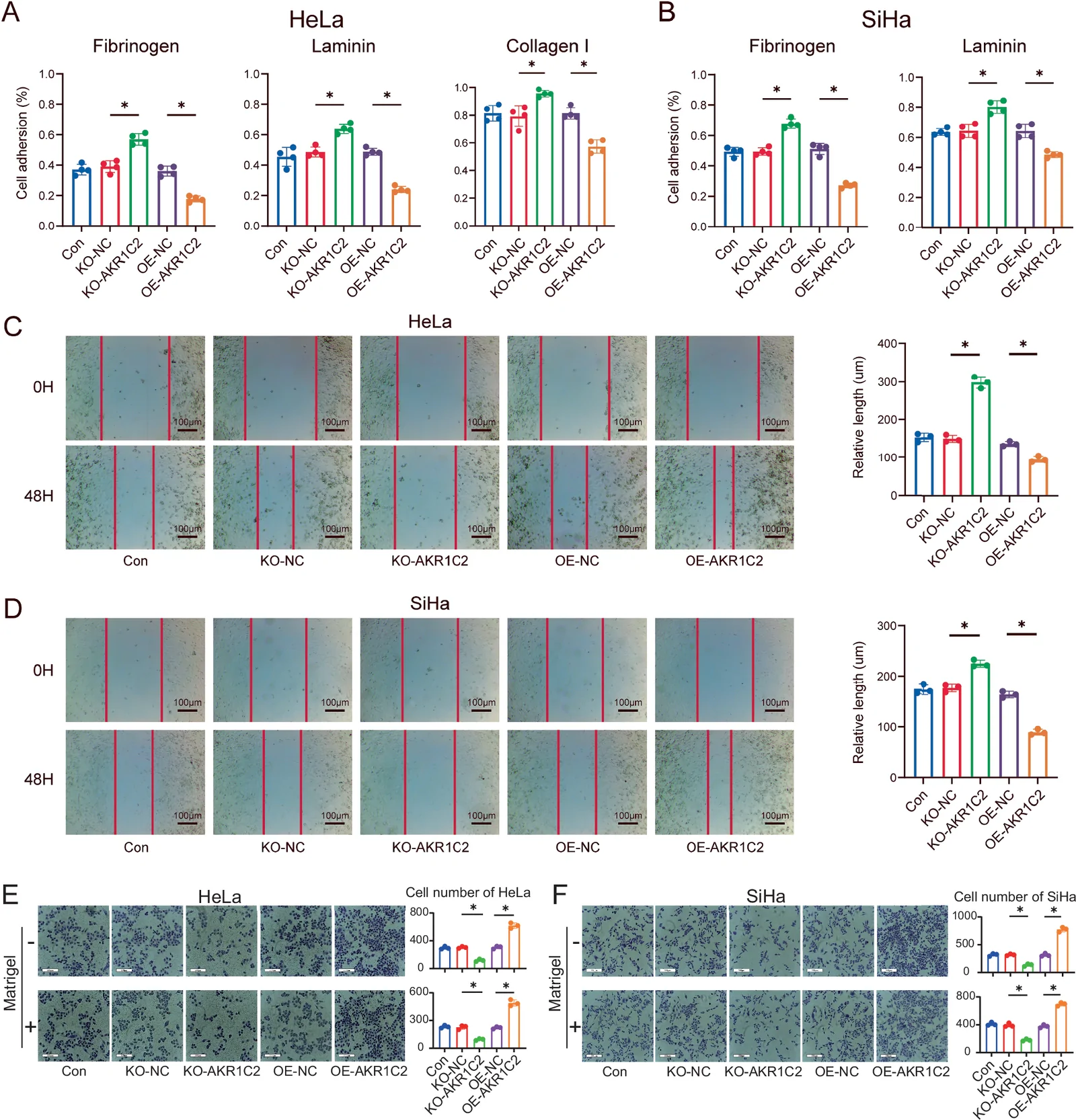
AKR1C2 regulated the invasion and metastasis of cervical cancer cells in vitro. **A**–**B** Bar plots showing the relative adhesion ability of cells with different AKR1C2 expression cultured on plates precoated with different extracellular matrix components. **C**–**D** Wound-healing assays for HeLa and SiHa cells to determine the effect of AKR1C2 expression on cell migration. Bar plots counting the relative length of cell migration were shown on the right of representative bright field images. **E**–**F** Transwell assays with and without Matrigel to determine the effect of AKR1C2 expression on invasion and migration of HeLa and SiHa cells. Bar plots counting the number of cells were shown on the right of representative bright field images. *p* values in all bar plots were calculated by non-parametric *t*-test. *p* values: *p < 0.05, **p < 0.01, ***p < 0.001, ****p < 0.001, ns not significantly different.

AKR1C2 was selected as a prominent candidate gene of interest for two reasons: first, it was significantly up-regulated in LNM samples; second, it was the only independent risk factor for oncological survival among the proteins of the model. Gene sequencing and Western blotting confirmed the high knockout efficiency of AKR1C2 in HeLa and SiHa cells (Supplementary materials and methods). RT-qPCR and Western blotting confirmed high knock-in efficiency in these cells (Supplementary materials and methods). Subsequently, when AKR1C2 expression is elevated, cells exhibited lower adhesion ability and vice versa, and this feature is not affected by specific adhesion proteins (Fig. 4A, B and Supplementary Fig. S5A–B). Wound healing and Transwell invasion assays showed that cell motility, migration and invasion were suppressed upon AKR1C2 knockout and promoted upon overexpression (Fig. 4C–F). The CCK-8 assays showed that AKR1C2 wasn’t associated with cell proliferation (Supplementary Fig. S5C–D). Apoptosis assays showed that cell apoptosis was suppressed and promoted when AKR1C2 was overexpressed and knocked out, respectively (Supplementary Fig. S5E–F).

Western blotting revealed that AKR1C2 knockout and overexpression regulated the expression of multiple extracellular matrix (ECM)-related proteins (including COL2A1, COL3A1, and COL4A1), adhesion complex proteins, integrins, as well as epithelial-mesenchymal transition (EMT) markers and transcription factors in HeLa (Supplementary Fig. S6A–B) and SiHa cells (Supplementary Fig. S7A–B).

**Fig. 5:**
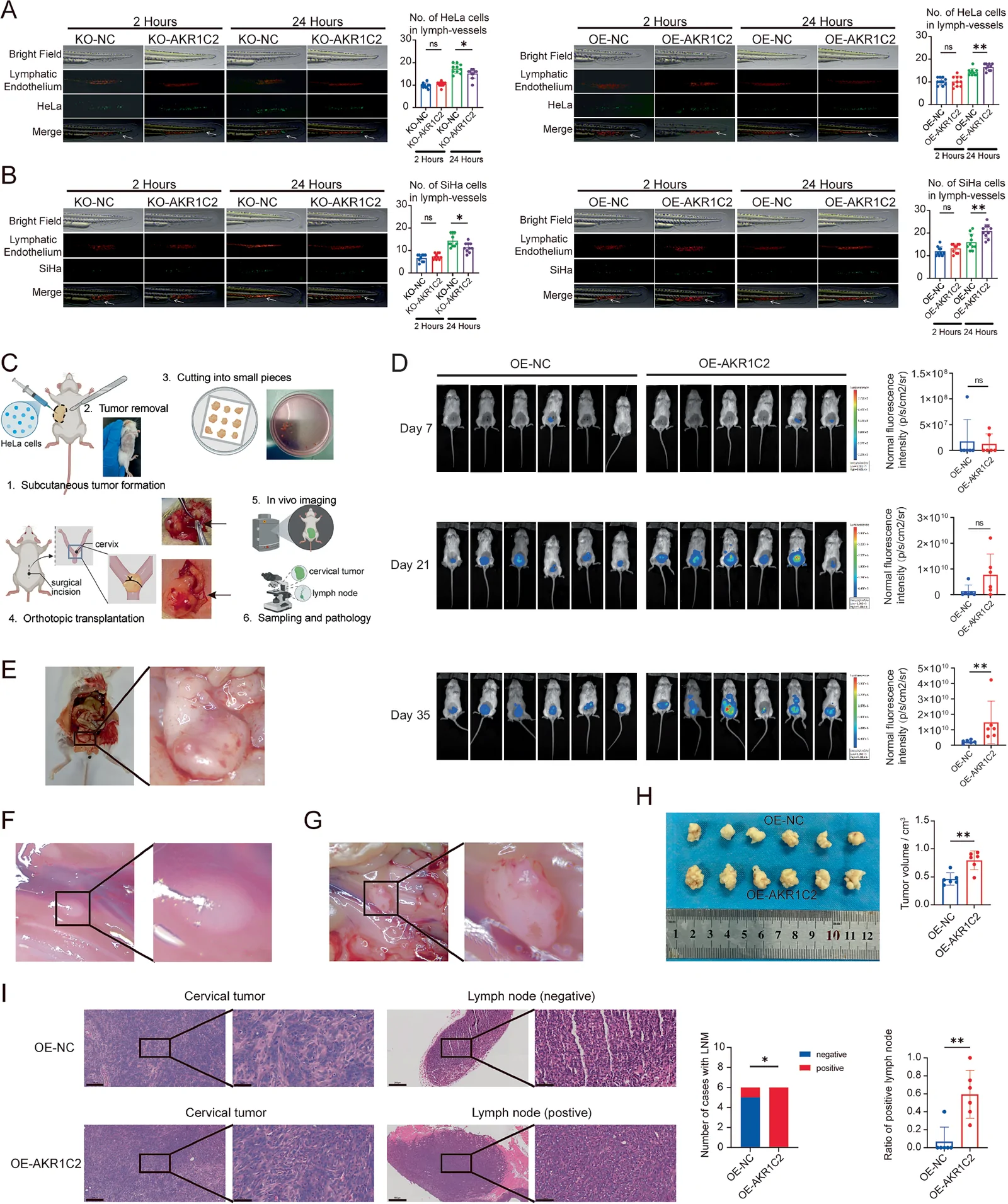
AKR1C2 promoted LNM of cervical cancer cells in vivo. **A**–**B** Zebrafish xenograft models of cervical cancer for HeLa and SiHa cells, respectively. Red and green fluorescence represented lymphatic vessels and tumor cells, respectively. Arrows indicated tumor cells with LNM. Bar plots counting the number of cells with LNM were shown on the right of the representative fluorescence images. **C** The experimental flow chart about orthotopic xenograft model of NOD/SCID mice. The arrow indicates a cervical transplant tumor. **D** On days 7, 21, and 35 post-transplantation, in vivo imaging to determine the effect of AKR1C2 expression on tumor metastasis. Bar plots analyzing‌ the fluorescence intensity and scope were shown on the right of the fluorescence images. **E**–**G** Anatomical diagram of cervical lesions, normal and enlarged lymph node, respectively. **H** The measurement of cervical lesion volume to determine the effect of AKR1C2 expression on tumor proliferation. Bar plots analyzing the volume of cervical tumor were shown on the right of representative bright field images. **I** H&E staining to determine the effect of AKR1C2 expression on LNM of cervical cancer. Bar plots counting the number of positive lymph node were shown on the right of the representative bright field images. *p* values in all bar plots were calculated by non-parametric t-test or Fisher exact probability test. *p* values: *p < 0.05, **p < 0.01, ***p < 0.001, ****p < 0.001, ns not significantly different.

Tumor-implanted zebrafish model assays demonstrated that lymphatic metastasis of tumor cells was suppressed and promoted when AKR1C2 was knocked out and overexpressed, respectively (Fig. 5A, B).

The experimental flow chart for the orthotopic xenograft model of NOD/SCID mice is shown in Fig. 5C. On days 7 and 21 post-transplantation, no difference was observed between AKR1C2-overexpressing and control groups by in vivo imaging. However, on day 35 post-transplantation, the AKR1C2-overexpressing group showed significantly higher fluorescence intensity and area than control group (*p* < 0.01) (Fig. 5D). Upon dissection, cervical lesions, normal and enlarged lymph nodes from pelvic and abdominal cavities were observed, respectively (Fig. 5E–G). The AKR1C2-overexpressing group had a significantly larger volume of cervical tumor than the control group (*p* < 0.05) (Fig. 5H). Furthermore, H&E staining showed that the AKR1C2-overexpressing group had a significantly higher rate of LNM than control group (Fig. 5I).

### AKR1C2 regulates SPINT2 to induce extracellular matrix remodeling

**Fig. 6:**
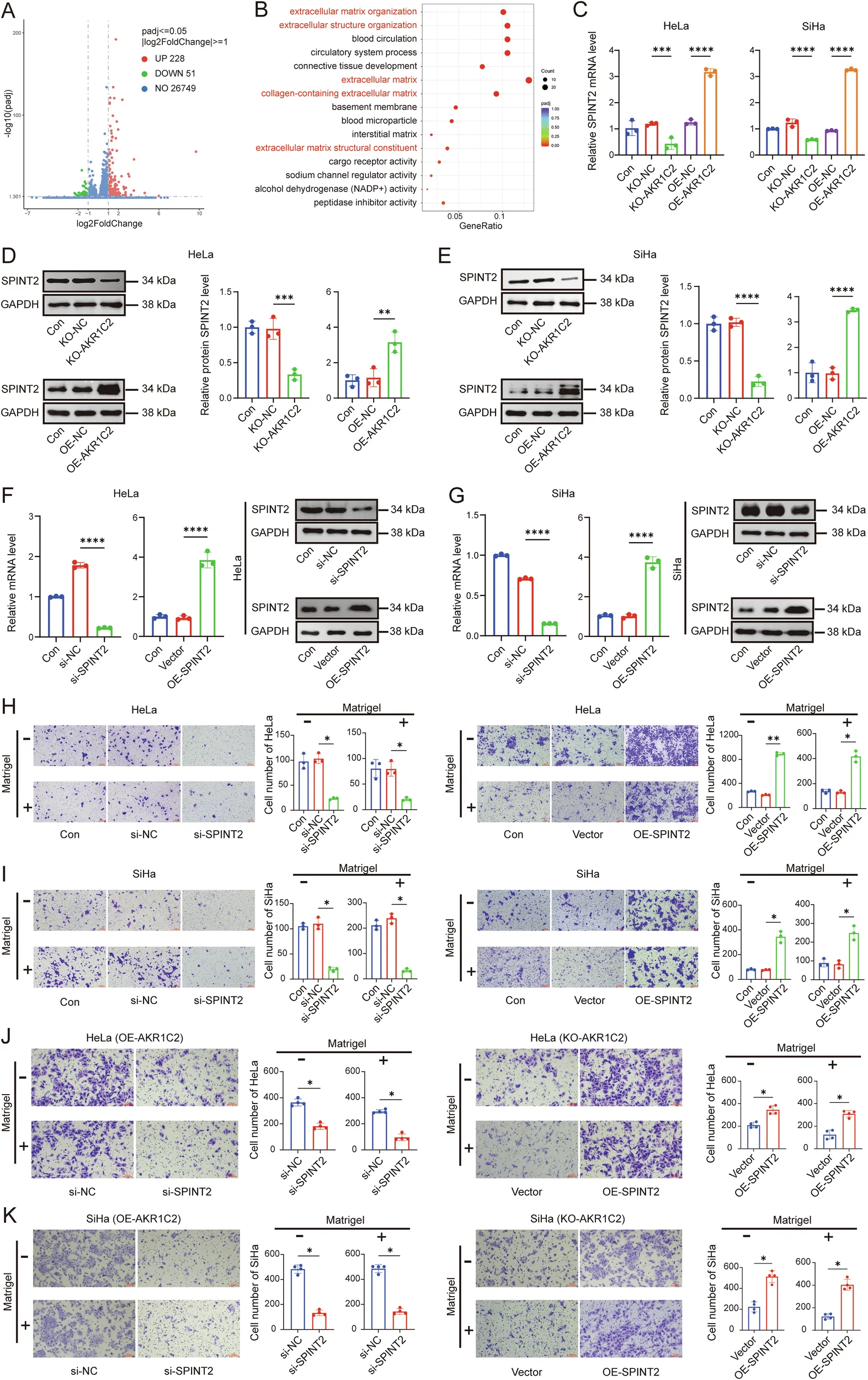
SPINT2 was regulated by AKR1C2 in vitro. **A** Volcano plot of different expression of mRNA (fold-change > 2.0) in HeLa cells with stable AKR1C2 overexpression. **B** Scatterplot of different expression of mRNA based on GO term enrichment. **C**–**E** The expression of SPINT2 in HeLa and SiHa cells were demonstrated by RT-qPCR and Western blotting, respectively, when AKR1C2 were knocked out or overexpressed. GAPDH was used as an internal control. **F**–**G** HeLa and SiHa cells transfected with control or SPINT2 siRNA and control or SPINT2 plasmid were demonstrated by RT-qPCR and Western blotting, respectively. GAPDH was used as a loading control. **H**–**I** Transwell assays with and without Matrigel to determine the effect of SPINT2 expression on invasion and migration in HeLa and SiHa cells. **J**–**K** Rescue assays regarding SPINT2 affecting the effect of AKR1C2 on migration and invasion ability in HeLa and SiHa cells. Bar plots counting the number of cells were shown on the right of representative bright field images. *p* values in all bar plots were calculated by non-parametric *t*-test. *p* values: *p < 0.05, **p < 0.01, ***p < 0.001, ****p < 0.001, ns not significantly different.

Differentially expressed genes were identified by RNA sequencing of stable AKR1C2 overexpression and its control in HeLa cells. The results showed that 228 genes were upregulated and 51 genes were downregulated with a fold-change >2.0 (Fig. 6A). Enriched Gene Ontology (GO) terms indicated that extracellular matrix organization, extracellular structure organization, extracellular matrix, collagen-containing extracellular matrix and extracellular matrix structural constituent were associated with overexpression of AKR1C2 (Fig. 6B). Among these 34 differentially expressed genes that were annotated to the GO term “extracellular matrix organization”, the expression of SPINT2 was found to be consistently associated with AKR1C2 by RT-qPCR and Western blotting in both HeLa and SiHa cells (Fig. 6C–E). The mRNA expression of the other 33 genes wasn’t completely associated with AKR1C2 in HeLa and SiHa cells (Supplementary Figs. S8–9). Among 34 genes analyzed in the GEPIA database, only SPINT2 and LOXL2 mRNA expression were found to be associated with worse DFS and OS for cervical cancer (Supplementary Figs. S10–11). Hence, SPINT2 was selected for further investigation. RT-qPCR and Western blotting confirmed high transfection efficiency (Fig. 6F, G). Transwell invasion assays showed that cell motility, migration and invasion were suppressed or elevated when SPINT2 was silenced or overexpressed (Fig. 6H, I). Furthermore, rescue experiment demonstrated that when AKR1C2 was overexpressed, silencing of SPINT2 could reduce the effect of AKR1C2 on cell motility, migration and invasion ability, and vice versa (Fig. 6J, K).

### AKR1C2 interacts with H4C13 in the nucleus

**Fig. 7:**
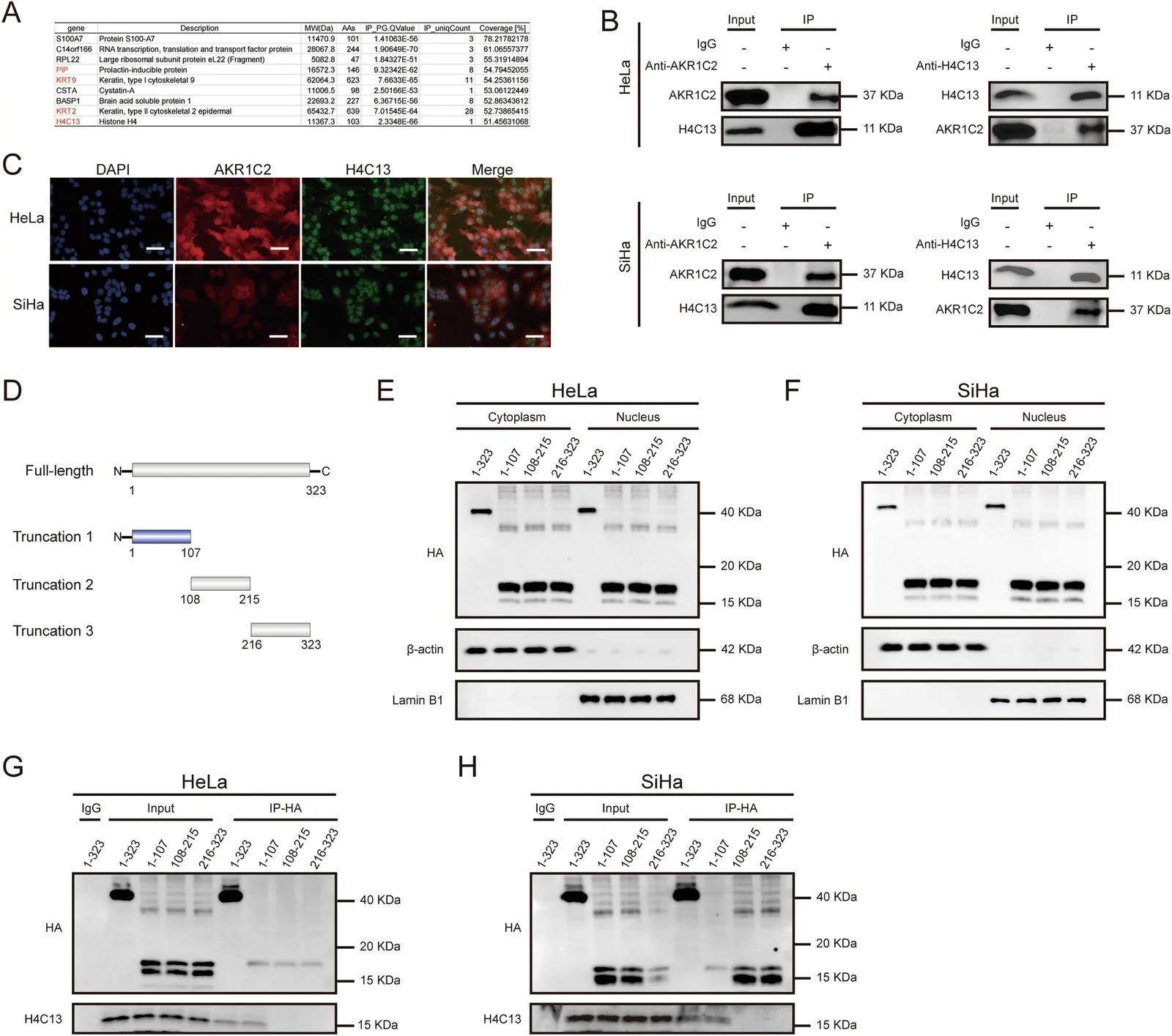
AKR1C2 interacts with H4C13 in the nucleus via its N-terminal domain. **A** High-confidence candidates identified by IP-MS using selection criteria of coverage > 50% and IP_uniqCount ≥ 1. Those marked in red were further investigated. **B** Co-IP assays confirming the interaction between protein AKR1C2 and H4C13. **C** IF assays showing co-localization of protein AKR1C2 and H4C13. Scale bars, 20 μm. **D** Schematic representation of full-length AKR1C2 and three truncated mutants (Trunc 1: 1–107 aa, Trunc 2: 108–215 aa, Trunc 3: 216–323 aa). The N-terminal region (1–107 aa) is highlighted in blue, indicating its critical role in H4C13 interaction. **E**–**F** Subcellular fractionation assays showing the distribution of HA-tagged AKR1C2 full-length and truncation mutants (1–107 aa, 108–215 aa, and 216–323 aa) in HeLa and SiHa cells. β-actin and Lamin B1 served as cytoplasmic and nuclear loading controls, respectively. **G**–**H** Co-IP assays using nuclear extracts demonstrating the interaction between endogenous H4C13 and HA-tagged AKR1C2 full-length or truncation mutants (1–107 aa, 108–215 aa, and 216–323 aa) in HeLa and SiHa cells. Immunoprecipitation was performed with anti-HA antibody, and immunoprecipitates were immunoblotted with anti-H4C13 antibody.

IP-MS assays identified a series of proteins that may interact with AKR1C2 (Supplementary file). Using selection criteria of coverage >50% and IP_uniqCount ≥1, we identified several high-confidence candidates, including PIP, KRT9, KRT2 and H4C13. Other proteins not marked in red were excluded due to the lack of commercially available reagents (Fig. 7A). Among the total proteins identified by mass spectrometry, the STRING database (version 12.0) suggested that SOD1, NQO1, GCLM and ALDH3A1 may interact with AKR1C2 (Supplementary file). These potential interactions were further investigated. Ultimately, Co-IP assays confirmed a significant interaction between AKR1C2 and H4C13 in HeLa and SiHa cells (Fig. 7B). Under the same experimental conditions, a weak interaction was detected with GCLM, whereas no discernible interaction was observed with the other tested proteins (Supplementary Fig. S12). IF assays further determined the colocalization of AKR1C2 and H4C13 in the nucleus (Fig. 7C). To map the H4C13-interacting domain of AKR1C2, we generated three truncated mutants covering the full-length AKR1C2 protein (1–107 aa, 108–215 aa, and 216–323 aa), along with the full-length construct as a control (Fig. 7D). To identify the specific domain of AKR1C2 responsible for its interaction with H4C13, three truncated mutants were generated covering the full-length AKR1C2 protein (1–107 aa, 108–215 aa, and 216–323 aa), along with the full-length construct as a control. Subcellular fractionation assays revealed that all three truncation mutants were localized in both the cytoplasmic and nuclear fractions, similar to the full-length AKR1C2 protein, indicating that nuclear entry does not require a specific domain within the tested regions (Fig. 7E, F). Co-IP assays were then performed using nuclear extracts to evaluate the binding capacity of each truncation mutant to endogenous H4C13. Notably, among the three truncation mutants, only the 1–107 aa fragment retained the ability to interact with H4C13, whereas the 108–215 aa and 216–323 aa fragments showed no interaction with H4C13 in Co-IP assays (Fig. 7G, H). These results suggest that the N-terminal region of AKR1C2 (1–107 aa) is necessary and sufficient for its interaction with H4C13.

### H4C13 mediates the effect of AKR1C2 on migration, invasion and SPINT2 expression

**Fig. 8:**
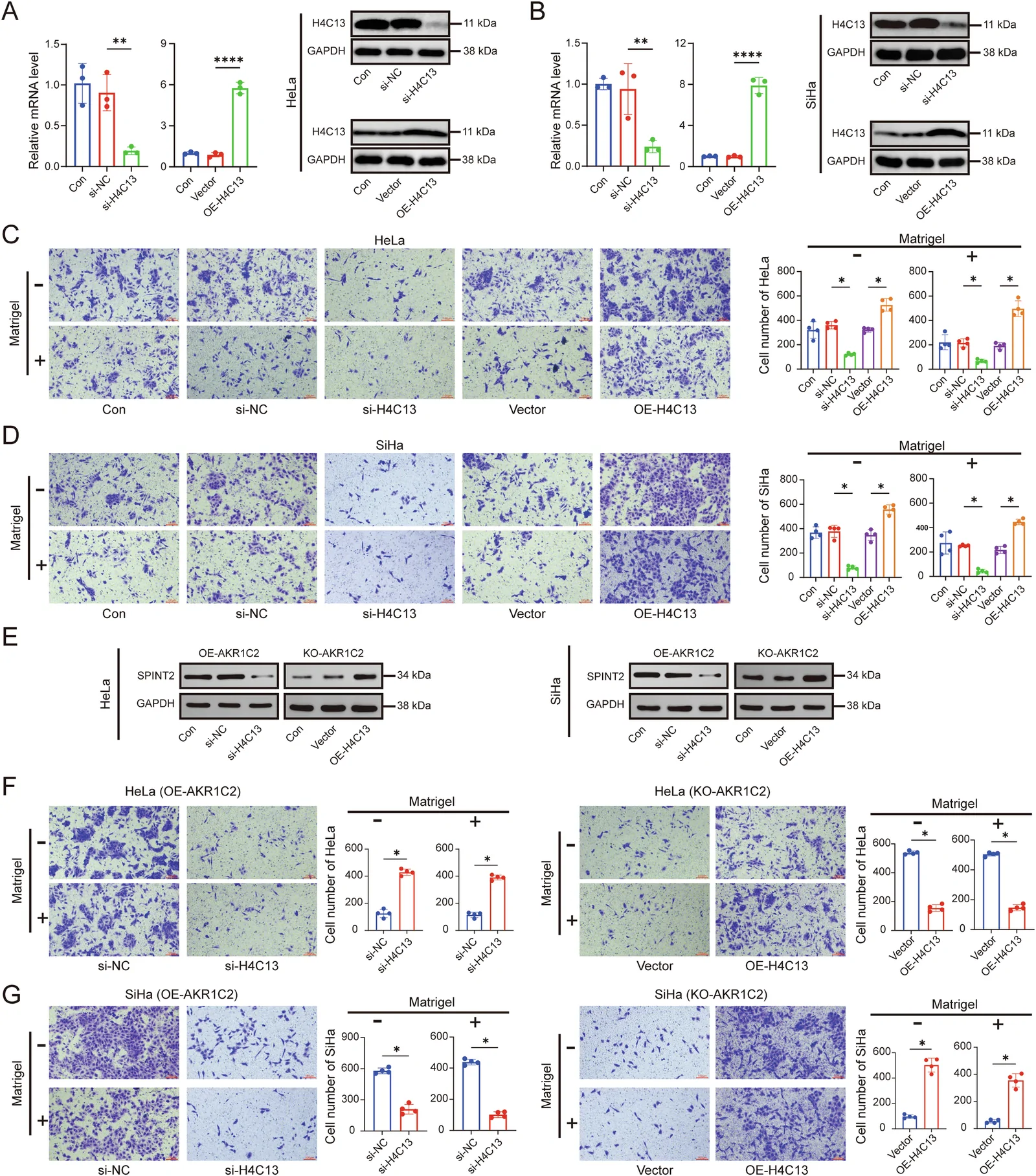
H4C13 mediates the effect of AKR1C2 on migration, invasion and SPINT2 expression. **A**–**B** RT-qPCR and Western blotting confirming the efficient knockdown and overexpression of H4C13 in HeLa and SiHa cells. GAPDH served as a loading control. **C**–**D** Transwell assays with and without Matrigel showing the effect of H4C13 silencing or overexpression on migration and invasion in HeLa and SiHa cells. **E** Rescue assays by Western blotting showing that H4C13 silencing reversed the upregulation of SPINT2 induced by AKR1C2 overexpression, while H4C13 overexpression restored SPINT2 expression in AKR1C2-knockout cells. Results are shown for both HeLa and SiHa cells. **F**–**G** Rescue assays by Transwell showing that H4C13 silencing reduced the enhanced migration and invasion induced by AKR1C2 overexpression, while H4C13 overexpression increased the reduced migration and invasion in AKR1C2-knockout cells, in HeLa and SiHa cells. Bar plots counting the number of cells were shown on the right of representative bright field images. *p* values in all bar plots were calculated by non-parametric *t*-test. *p* values: *p < 0.05, **p < 0.01, ***p < 0.001, ****p < 0.001, ns not significantly different.

RT-qPCR and Western blotting confirmed the efficient knockdown and overexpression of H4C13 in HeLa and SiHa cells (Fig. 8A, B). Transwell assays demonstrated that H4C13 silencing suppressed, while H4C13 overexpression promoted, the migration and invasion of cervical cancer cells (Fig. 8C, D). To investigate whether H4C13 mediates the effect of AKR1C2, rescue experiments were performed. Western blotting showed that in AKR1C2-overexpressing cells, H4C13 silencing reversed the upregulation of SPINT2 induced by AKR1C2 overexpression; conversely, in AKR1C2-knockout cells, H4C13 overexpression restored SPINT2 expression (Fig. 8E). Consistently, Transwell assays revealed that in AKR1C2-overexpressing cells, H4C13 silencing reduced the enhanced migration and invasion, while in AKR1C2-knockout cells, H4C13 overexpression increased the reduced migration and invasion in both HeLa and SiHa cells (Fig. 8F, G).

## Discussion

This study identified a set of proteomic biomarkers associated with LNM in cervical cancer. Furthermore, LNM was significantly associated with intermediate filament proteins, cytoskeletal proteins and extracellular matrix organization. We demonstrated that a proteomic model including AKR1C2, ARHGAP26 and ATP6AP2 could accurately predict LNM in patients with stage IB1-IIA2 cervical cancer. In vitro assays and dual in vivo models showed that AKR1C2 interacts with H4C13 in the nucleus via its N-terminal region (amino acids 1–107) to regulate SPINT2, thereby influencing the motility, migration and invasion of cervical cancer cells, ultimately remodeling the extracellular matrix and promoting LNM.

Previous studies have attempted to preoperatively identify LNM by imaging, usually including CT and MRI [22]. However, these imaging modalities exhibit low sensitivity for predicting LNM, with a missed diagnosis rate exceeding 20%, likely due to their inability to detect LNM in nodes with normal size and shape [8]. The use of positron emission tomography-CT has been limited due to high cost and issues with false-positive and false-negative results [23]. In addition, radiomics-based studies have reported accuracies between 80% and 90%, sensitivities of 80–85%, and specificities of 75–85% for predicting LNM [24–26]. However, these sensitivity rates remain slightly lower than those achieved by our model. This would still lead to a considerable missed diagnosis rate for LNM and more patients requiring adjuvant radiotherapy after radical surgery.

Several previous studies have identified some proteins associated with LNM by IHC. However, these protein biomarkers showed low predictive value with sensitivity around 80% and specificity around 55% [27, 28]. This may be due to reliance on a single protein biomarker. Few studies have attempted to identify LNM by proteomics. One previous study identified three proteins (SERPINB5, FABP5, and ZNF512) as predictors of LNM by cervical tissue-based DIA proteomics [29]. The results were promising, whereas the study mainly focused on biomarker discovery and didn’t extensively validate their predictive model through independent cohorts. In addition, they didn’t verify the protein function through in vitro experiments. In contrast, the current study established a valuable model based on DIA proteomics, with AUC values of 89.0% and 86.1% in the training set and validation set, respectively. The parameters of the model suggested good clinical utility. The survival analysis also showed that the current model was an independent risk factor for tumor recurrence and death in cervical cancer patients. Moreover, in vitro and in vivo experiments revealed that AKR1C2 promotes invasion and metastasis in cervical cancer cells. This suggests that AKR1C2 is a potential therapeutic target for cervical cancer patients.

One previous study found that microRNA-21 was significantly higher in patients with LNM, with an AUC value of 0.856 [30]. However, the sensitivity and specificity of this biomarker were not further clarified. Several studies also identified serum SCC-Ag as an independent risk factor for LNM. However, its predictive value was also significantly lower than that of our model [16, 17]. Previous studies have attempted to predict LNM in cervical cancer using gene expression levels. For example, one study identified hsa-miR-508, hsa-miR-509-2, and hsa-miR-526b as significantly associated with LNM through TCGA data [18]. Another previous study identified 7 key genes significantly associated with LNM from TCGA [19]. However, these two studies didn’t further demonstrate the validation of predictive performance, such as accuracy, sensitivity and specificity. In addition, another study identified 8 biomarkers as a model from the TCGA cohort to predict LNM. In the training cohort, the model had an AUC of 0.931 with a sensitivity of 79.2% and a specificity of 86.9%. However, in the other two validation cohorts, the AUC value decreased significantly to 0.713 and 0.660 [20]. Therefore, this gene-based model has obvious limitations in predicting LNM. Compared to previous studies, the current model had a significantly higher predictive accuracy for LNM. Another advantage is the low incidence of LNM in the low-risk group. Only one (1.8%) and two (2.0%) patients missed the diagnosis in the training and validation sets, respectively. Therefore, the current model can be used to reduce the risk of false-negative LNM diagnosis before radical surgery. This characteristic makes the model more practical by reducing severe comorbidities associated with surgery followed by adjuvant chemoradiotherapy.

Previous studies have reported that AKR1C2 protein abundance is associated with the occurrence of various tumors [31, 32]. It is related to the expression of dihydrotestosterone, which affects prostate cancer metastasis by regulating the androgen receptor signaling pathway [33]. Epidermal growth factor receptor signaling may mediate nasopharyngeal carcinoma metastasis by activating AKR1C2 [34]. In addition, AKR1C2 is closely related to the incidence of liver cancer and breast cancer [35, 36]. However, few studies have explored the relationship between AKR1C2 and cervical cancer pathogenesis, with most focusing on platinum resistance. One previous study showed that AKR1C2 was overexpressed in cisplatin-resistant HeLa cells. After the protein abundance of AKR1C2 was downregulated by small interfering RNA, HeLa cells regained their sensitivity to cisplatin. When mefenamic acid, the AKR1C2 inhibitor, was administered, the sensitivity to cisplatin could also be enhanced [37]. Other studies also showed that AKR1C2 is associated with resistance to cisplatin, oxaliplatin and doxorubicin [38, 39]. Furthermore, AKR1C2 has been found to be associated with LNM through TCGA and the FerrDb database, and is more highly expressed in lung adenocarcinoma tissues than in normal lung epithelial cells [40]. However, the relationship between AKR1C2 and the incidence of cervical cancer, especially LNM, has not been reported. In the current study, we found that LNM was significantly associated with intermediate filament proteins, cytoskeletal proteins and extracellular matrix organization. In vitro assays demonstrated that AKR1C2 reduces the adhesion ability of cervical cancer cells and promotes migration and invasion. Furthermore, dual in vivo models, including tumor-implanted zebrafish and orthotopic xenograft mice, confirmed that AKR1C2 enhances LNM. Therefore, AKR1C2 plays a significant role in promoting LNM in cervical cancer. The extracellular matrix-related protein SPINT2 was regulated by AKR1C2. Therefore, the decrease in cell adhesion ability may be an important mechanism by which AKR1C2 promotes lymphatic metastasis. Similarly, several studies have reported that SPINT2 contributes to metastasis and poor clinical outcomes in multiple types of tumors, including colon cancer and prostate cancer [41, 42]. However, SPINT2 is significantly reduced in metastatic melanoma compared to early-stage primary melanomas, which is related to DNA hypomethylation [43]. These findings suggest that the expression and regulation of SPINT2 vary significantly depending on tumor types.

Notably, our MS-based proteomic analysis independently identified ECM-related protein changes in LNM samples. Among the 42 DEPs, TNXB was upregulated while KRT3, KRT17, and KRT78 were downregulated in LNM tissues, consistent with the GSEA enrichment of “fibronectin type III” and “intermediate filament” signatures. Only four ECM proteins reached statistical significance in the MS dataset, likely due to the limited discovery cohort size (*n* = 36) and the inherent lower sensitivity of MS for low-abundance ECM proteins. Nevertheless, these findings provide direct protein-level evidence supporting ECM remodeling in LNM. This convergence of proteomic and transcriptomic data strengthens the conclusion that ECM reorganization is a hallmark of LNM in cervical cancer. In the present study, RNA sequencing revealed that AKR1C2 overexpression enriched genes involved in extracellular matrix organization and collagen-containing extracellular matrix. Consistently, the expression of SPINT2, a key ECM-related regulator, was tightly correlated with AKR1C2 at both mRNA and protein levels. Moreover, AKR1C2 knockout and overexpression modulated the expression of multiple ECM-associated proteins (including COL2A1, COL3A1, and COL4A1), adhesion complex proteins, integrins, as well as EMT markers and transcription factors. These molecular changes were accompanied by altered cell adhesion, migration, and invasion. Collectively, these mutually reinforcing findings suggest that AKR1C2 promotes lymph node metastasis in cervical cancer via extracellular matrix remodeling, with SPINT2 serving as a critical mediator. Notably, our study reveals an unprecedented nuclear role for AKR1C2 in cervical cancer, where it interacts with H4C13 to regulate SPINT2 expression, a mechanism that has not been previously described.

AKR1C2 is traditionally characterized as a cytosolic aldo-keto reductase that catalyzes the NAD(P)H-dependent reduction of ketosteroids, playing a key role in steroid hormone metabolism and detoxification [44, 45]. In the present study, we found that AKR1C2 is localized in the nucleus, where it directly interacts with H4C13, a histone H4 family member, to upregulate SPINT2 expression, thereby promoting lymph node metastasis. Domain mapping experiments further identified the N-terminal region (amino acids 1–107) of AKR1C2 as the critical domain responsible for this interaction, providing a molecular basis for targeting this axis. Notably, this nuclear function appears to be independent of its canonical enzymatic activity, suggesting that AKR1C2 may exert non-canonical functions beyond metabolic regulation. In support of the relevance of SPINT2 in cervical cancer, a previous study reported that HPV E6/E7 infection affects SPINT2 methylation in cervical cancer cell lines, and aberrant methylation of the SPINT2 gene may contribute to cervical carcinogenesis [46]. Collectively, these findings support a model in which AKR1C2, through its N-terminal domain, interacts with H4C13 in the nucleus to regulate SPINT2 expression, ultimately facilitating lymph node metastasis in cervical cancer. In addition, exosomal lncAKR1C2 derived from gastric cancer cells has been shown to promote lymphangiogenesis and LNM in vivo [47]. These findings suggest that AKR1C2 may influence lymphatic metastasis through multiple mechanisms in different tumor types.

This study had several limitations. First, due to the retrospective nature of this study, selection bias was inherent. Second, although the model had perfect performance in diagnosing the risk of false-negative LNM diagnoses, it has not yet been directly compared with CT or MRI. Third, this study did not investigate how the AKR1C2-H4C13 interaction regulates SPINT2 at a molecular level. Fourth, while we observed nuclear localization of ARHGAP26 in cervical cancer tissues using a validated antibody, the functional significance of this nuclear distribution and whether it contributes to ARHGAP26’s role as a predictive biomarker remain unknown and warrant further investigation.

## Conclusion

This study identified a set of proteomic biomarkers associated with LNM in patients with stage IB1-IIA2 cervical cancer. A proteomic model including AKR1C2, ARHGAP26 and ATP6AP2 accurately predicts LNM, and can be determined by cervical biopsy prior to radical surgery. Therefore, the model could guide patients to choose radical surgery or concurrent chemoradiotherapy. In addition, we uncovered a non-canonical nuclear function of AKR1C2. Specifically, it interacts with H4C13 in the nucleus via its N-terminal region (amino acids 1–107) to regulate SPINT2, ultimately remodeling the extracellular matrix and promoting LNM in cervical cancer.

## Methods

### Ethics statement

This retrospective study was approved by the Institutional Review Board of the First People’s Hospital of Foshan (2020-17) and registered in the Chinese Clinical Trial Registry (http://www.chictr.org.cn; ChiCTR2000040945). The requirement of informed consent was waived due to the retrospective nature of the study. The study was conducted in accordance with the tenets of the Declaration of Helsinki.

### Patient samples

Paraffin-embedded cervical tissue and data of patients who underwent surgical treatment for cervical cancer at our institution between 2013 and 2016 were collected and reviewed. Inclusion criteria were as follows: (1) age ≥ 18 years; (2) clinical stage IB1-IIA2 cervical cancer according to the 2009 FIGO staging system [48]; (3) squamous cell carcinoma, adenocarcinoma, or adenosquamous carcinoma of the cervix; (4) those who underwent type C open radical hysterectomy and bilateral pelvic lymphadenectomy (Querleu-Morrow classification). Exclusion criteria were: (1) adjuvant chemotherapy or radiotherapy prior to surgery; (2) carcinoma of the cervical stump; (3) pregnancy; (4) presence of cancer other than cervical cancer; or (5) loss to follow-up.

A total of 101 patients were included in the training set. The patient flow chart was shown in the Supplementary Fig. S1. The clinical and pathological characteristics of the 101 patients with stage IB1-IIA2 cervical cancer were summarized in the Supplementary Table S1. Of the 101 patients, 24 (23.8%) were found to have LNM and 77 (76.2%) did not have LNM, as diagnosed by pathology review. From this cohort, 18 LNM and 18 NLNM cases were selected via 1:1 PSM for the DIA-MS discovery phase, and the full cohort (*n* = 101) was used for IHC validation in the training set.

The clinical and pathological characteristics of patients were compared between the LNM and NLNM groups after propensity score matching (PSM), and the results are shown in the Supplementary Table S2. The baseline characteristics of patients with LNM or NLNM were comparable after PSM.

### Protein extraction from paraffin-embedded cervical tumors

All hematoxylin and eosin-stained slides of 101 cases were reviewed, and those containing abundant tumor tissue (≥ 80%) were selected for subsequent case matching [30]. Finally, 18 cervical tumor tissues with LNM and 18 without LNM were selected after 1:1 PSM. Clinicopathological variables were selected a priori based on their potential to influence LNM, and included age, stage, tumor size, histologic type, grade, stromal invasion depth, LVSI, PMI, and resection margin involvement (RMI).

Protein extraction was performed on formalin-fixed paraffin-embedded cervical tissue [49]. The procedure in detail is provided in the Supplementary Materials and Methods.

### DIA proteomics

Detailed protocols for DIA proteomics and data analysis are provided in the Supplementary Materials and Methods.

### IHC and predictive modeling

DEPs with a fold-change >2.0 were validated by IHC. IHC was performed according to the manufacturer’s instructions, and details were provided in the Supplementary Materials and Methods. Antibodies included rabbit polyclonal antibodies against AKR1C2, GSS, FBXO22, ARHGAP26, KRT78, and ATP6AP2. Detailed antibody information was provided in the Supplementary Materials and Methods.

IHC evaluation of the above proteins was performed independently by two pathologists who were blinded to the clinical information of the patients. An IRS system was used to evaluate the immunohistochemical results. The percentage of positively stained tumor cells was scored as 0 (<1%), 1 (1%–10%), 2 (11%–50%), 3 (51%–80%), or 4 (˃80%), and the intensity of staining was scored as 0 (none), 1 (weak), 2 (moderate), or 3 (strong). Multiplication of these scores resulted in an IRS ranging from 0 to 12 [50].

Multivariate forward stepwise logistic regression analysis was performed to identify the independent risk factors for LNM. Furthermore, a preoperative predictive model was constructed based on three independent risk factors, including AKR1C2, ARHGAP26 and ATP6AP2. The value of this model was also evaluated by ROC curve analysis, calibration curve and DCA.

An independent validation cohort of 175 patients with stage IB1-IIA2 cervical cancer between 2013 and 2016, from Anyang Cancer Hospital was used to validate the value of the model. The details of inclusion and exclusion criteria, immunohistochemical evaluation and ROC curve analysis were the same as above.

### Cell culture

Human cervical cancer cell lines HeLa (RRID: CVCL_0030) and SiHa (RRID: CVCL_0032) were obtained from the American Type Culture Collection (ATCC, USA) in 2021. Cells were resuscitated and cultured in DMEM complete medium (supplemented with 10% FBS and 1% penicillin/streptomycin), at 37 °C in a 5% CO2 humidity incubator. When cells reached approximately 80% confluence, they were digested with 0.25% trypsin (containing 0.02% EDTA) and passaged, or frozen in complete medium containing 10% serum and 10% DMSO for storage. Cells were authenticated in 2024 by STR profiling using the Axygen genomic DNA extraction kit with a 21-STR amplification system, detected on an ABI 3730XL genetic analyzer, and routinely tested for mycoplasma contamination using a PCR-based assay (all negative).

### Construction of stable overexpression and gene knockout cell lines

Stable overexpression of AKR1C2 was achieved using lentivirus transduction in HeLa and SiHa cell lines. The procedure included vector construction, lentivirus packaging and production, target cell transfection, stable cell line selection, and expression validation. Further details are provided in the Supplementary Materials and Methods.

AKR1C2 gene knockout in HeLa and SiHa cell lines was constructed and the procedure included cell genotype identification, RNP method for electroporation of cells, detection of cutting efficiency, extreme dilution method for monoclonal plating and cultivation, and screening of homozygous knockout clones. Further details are provided in the Supplementary Materials and Methods.

### Cell transfection

siRNA targeting SPINT2 (Serine peptidase inhibitor, kunitz type 2), H4C13 (H4 Clustered Histone 13) and scramble negative control were designed and synthesized by RIBOBIO. Plasmids for overexpression of SPINT2 (Product No. P32047) and H4C13 (Product No. P48189) were obtained from MiaoLingBio, China. HeLa and SiHa cells were transfected with the siRNA and plasmids using Lipofectamine 2000 (Invitrogen), according to the manufacturer’s instructions. After 60 h, the transfection efficacy was evaluated by real-time quantitative PCR (RT-qPCR) and Western blotting. Information on the SPINT2 and H4C13 siRNA showed as follows: SPINT2 sense: 5’-CCAGCGAUAUGUUCAACUA-3’, anti-sense: 5’-UAGUUGAACAUAUCGCUGG-3’; H4C13 sense: 5’-GCAAAGUUCUGCGCGACAACA-3’, anti-sense: 5’-UUGUCGCGCAGAACUUUGCGG-3’.

### RT-qPCR

HeLa or SiHa cells (1 × 10^6^ cells/well) were seeded into six-well plates and transfected. Cell total RNA was then extracted with TRIzol (Invitrogen) according to the manufacturer’s instructions. Reverse transcription was performed using PrimeScript RT Master Mix Kit (TAKARA). RT-qPCR was performed with SYBR Premix Ex Taq (TAKARA), followed by a Roche 480 Real-Time PCR System (Roche). The invariant housekeeping gene GAPDH was used as an internal control, and mRNA expression levels relative to the control were determined by calculating ΔΔCt and then analyzed using 2^﹣ΔΔCt^. Primers used are listed in the Supplementary Materials and Methods.

### Western blotting

Western blotting was performed according to previously described standard methods. Briefly, the procedure included SDS-PAGE gel configuration, sample loading, electrophoresis, membrane transfer, blocking, incubation with primary and HRP-conjugated secondary antibodies, and membrane washing. Primary antibodies against AKR1C2 (abcam, No. ab96087), SPINT2 (abcam, No. ab128926), H4C13 (Immunoway, No. YM8088) and GAPDH (Proteintech, No.60004-1-Ig) were used. A total of 21 antibodies against common tumor proteins involved in invasion and metastasis are listed in the Supplementary Materials and Methods. The dilution ratio (1:1000) was used according to the manufacturer’s instructions. GAPDH was used as internal reference. Protein bands on the membranes were detected using an electrochemical luminescence kit and a chemiluminescence system (iBright^TM^ FL1500 imaging system). The intensities of the protein bands were determined using ImageJ (v1.43) software and compared with those of the GAPDH control.

### Function studies

Protocols for cell proliferation (CCK-8), cell adhesion, wound healing, Transwell invasion and apoptosis assays are provided in the Supplementary Materials and Methods.

### RNA sequencing

Total RNA was extracted from AKR1C2-overexpressing and control HeLa cells and sequenced using the Illumina Novaseq platform. The data analysis procedure included quality control, reads mapping to the reference genome, quantification of gene expression level, differential expression analysis and GO enrichment analysis of differentially expressed genes. Further details are provided in the Supplementary Materials and Methods.

### Immunofluorescence (IF) assays

IF assays were used to determine the co-localization of AKR1C2 and H4C13. Primary antibodies against AKR1C2 (abcam, No. ab179448) and H4C13 (Abmart, No. M015925) were used. Protocols in detail are provided in the Supplementary Materials and Methods.

### Immunoprecipitation-mass spectrometry (IP-MS) and Co-IP

Total protein was extracted from HeLa cells lysates. Protein concentration in the supernatants was quantified using the BCA method. AKR1C2 antibody and control IgG antibody were added to the IP samples and IgG control samples, respectively. Protein A/G magnetic beads were then added and incubated. The supernatant was obtained after elution according to the manufacturer’s instructions (Thermo Scientific™, No.88803). Subsequently, the IP samples were subjected to electrophoresis followed by Coomassie blue staining. The stained gel bands were excised and analyzed by Q Exactive^TM^ HF-X mass spectrometer (Thermo Fisher). Additionally, Co-IP assays were performed to determine the interaction between AKR1C2 protein and H4C13 in HeLa and SiHa cells. Primary antibodies against AKR1C2 (abcam, No. ab179448) and H4C13 (Immunoway, No. YM8088) were used. The other primary antibodies in detail are provided in the Supplementary Materials and Methods.

### Tumor-implanted zebrafish model and orthotopic xenograft model in NOD/SCID mice

The animal experiments were approved by the Institutional Animal Care and Use Committee (IACUC) of Fujian Maternity and Child Health Hospital (IACUC-FMCHH-2024-054) and conducted in accordance with institutional guidelines. The animal experiments were used to determine the effect of AKR1C2 on tumor cell lymphatic metastasis. NOD/SCID mice are inherently immunodeficient and lack functional T and B lymphocytes. Various tumor cells can be implanted in these mice with minimal rejection and graft-versus-host disease. Therefore, NOD/SCID mice have become a valuable model in research of cervical cancer with LNM [51]. Female NOD/SCID mice (4-5 weeks old; weight 17–18 g) were purchased from SPF Biotechnology Co., Ltd. (Suzhou, China). Orthotopic xenograft experiments were established in a specific pathogen-free (SPF) animal facility. Mice had ad libitum access to food and water. Following acclimatization, mice were randomly assigned to two groups (*n* = 6 per group): OE-NC group and OE-AKR1C2 group using a random number table. Further details about model construction are provided in the Supplementary Materials and Methods.

### Validation of key genes

The National Human Genome Research Institute (NHGRI) and the National Cancer Institute (NCI) collaborated to create TCGA, a comprehensive cancer research project. It is a free and open cancer genetic research database that molecularly characterized more than 20,000 primary cancers and matched normal samples from 33 different cancer types. Based on the Genotype-Tissue Expression (GTEx) database and TCGA, the Peking University team created the cancer data analysis website called Gene Expression Profiling Interactive Analysis (GEPIA) (http://gepia2.cancer-pku.cn/). The expression of important genes in cervical cancer samples was confirmed by GEPIA [52].

### Statistical analysis

Normality was assessed using the Shapiro–Wilk test, and homogeneity of variances was assessed using Levene’s test. For data that did not meet normality assumptions, non-parametric tests (Mann–Whitney or Kruskal–Wallis) were used.

Student’s *t*-test, one-way ANOVA and their non-parametric counterparts (Mann–Whitney and Kruskal–Wallis tests) were used to compare continuous variables, and chi-squared test was used to compare categorical variables between the two groups. Univariate and multivariate forward stepwise logistic regression analysis were performed to identify the independent risk factors for LNM. The performance of parameters for predicting LNM was determined using ROC curve analysis, calibration curve and DCA. Five-year DFS and OS were estimated and compared between groups using the Kaplan-Meier method and the log-rank test, respectively. For animal experiments, image analysis was performed by investigators blinded to group allocation.

To estimate the effects of LNM, 1:1 PSM was performed using nearest neighbor matching with a caliper of 0.2 standard deviations [58]. The appropriateness of the propensity score specification was assessed using standardized differences and the likelihood ratio test obtained from conditional logistic regression modeling.

All statistical analyses were performed with SPSS v26.0 (IBM Inc, Chicago, IL, USA), STATA (v17; STATA, College Station, TX; Computing Resource Center, Santa Monica, CA), GraphPad Prism 8 (GraphPad Software Inc, San Diego, CA), and the Matching package in R (v3.6.1; The R Foundation, Vienna, Austria). A two-tailed *p* < 0.05 was considered statistically significant. All quantitative data are presented as mean ± SD from at least three independent experiments unless otherwise indicated in the figure legends.

## Supplementary information

Supplementary data

Figure S1

Figure S2

Figure S3

Figure S4

Figure S5

Figure S6

Figure S7

Figure S8

Figure S9

Figure S10

Figure S11

Figure S12

Supplementary file

Full and uncropped western blots

## Data Availability

The raw data of mass spectrometry-based proteomics and IP-MS has been submitted to the ProteomeXchange (http://www.proteomexchange.org/; PXD044849 and PXD067378) or iProX (https://www.iprox.cn/; IPX0006959000 and IPX0013047000). The raw data of RNA sequencing has been submitted to the NCBI (PRJNA1250037). Any information required to reanalyze the data reported in this paper is available from the corresponding author. Other data generated in this study are available within the article and its supplementary data files.

## References

[1] Bray F, Laversanne M, Sung H, Ferlay J, Siegel RL, Soerjomataram I, et al. Global cancer statistics 2022: GLOBOCAN estimates of incidence and mortality worldwide for 36 cancers in 185 countries. CA Cancer J Clin. 2024;74:229–63. 10.3322/caac.21834 38572751

[2] Siegel RL, Kratzer TB, Wagle NS, Sung H, Jemal A. Cancer statistics, 2026. CA Cancer J Clin. 2026;76:e70043. 10.3322/caac.70043 PMC12798275 41528114

[3] Abu-Rustum NR, Yashar CM, Bean S, Bradley K, Campos SM, Chon HS, et al. NCCN guidelines insights: cervical cancer, version 1.2020. J Natl Compr Canc Netw. 2020;18:660–6. 10.6004/jnccn.2020.0027 32502976

[4] Mora-Soto N, Morante-Caicedo C, Caicedo-Martínez M, Viveros-Carreño D, Díaz ES, Rodríguez J, et al. Morbidity of radical surgery and postoperative radiotherapy in cervical cancer. Int J Gynaecol Obstet. 2025;171:189–98. 10.1002/ijgo.70281 PMC12321200 40735857

[5] Bhatla N, Aoki D, Sharma DN, Sankaranarayanan R. Cancer of the cervix uteri. Int J Gynaecol Obstet. 2018;143:22–36. 10.1002/ijgo.12611 30306584

[6] Özen S, Demircivi E, Turgut A, Sancı M. Evaluation of lymph node metastasis in cervical cancer: a retrospective comparison of preoperative MRI and PET/CT with postoperative histopathology results. Turk J Obstet Gynecol. 2025;22:129–33. 10.4274/tjod.galenos.2025.27482 PMC12136126 40462410

[7] Jiang CQ, Li XJ, Zhou ZY, Xin Q, Yu L. Imaging based artificial intelligence for predicting lymph node metastasis in cervical cancer patients: a systematic review and meta-analysis. Front Oncol. 2025;15:1532698. 10.3389/fonc.2025.1532698 PMC11906327 40094016

[8] Olthof EP, Bergink-Voorthuis BJ, Wenzel HHB, Mongula J, van der Velden J, Spijkerboer AM, et al. Diagnostic accuracy of MRI, CT, and [^18^F]FDG-PET-CT in detecting lymph node metastases in clinically early-stage cervical cancer - a nationwide Dutch cohort study. Insights Imaging. 2024;15:36. 10.1186/s13244-023-01589-1 PMC10853153 38332397

[9] He F, Zu S, Chen X, Liu J, Yi Y, Yang H, et al. Preoperative magnetic resonance imaging criteria for predicting lymph node metastasis in patients with stage IB1-IIA2 cervical cancer. Cancer Med. 2021;10:5429–36. 10.1002/cam4.4075 PMC8366085 34278729

[10] Wang X, Xu J, Zhang H, Qu P. Integrating the Silva pattern into clinical practice: a prognostic tool for tailoring surgery and adjuvant therapy in cervical adenocarcinoma. Cancer Treat Res Commun. 2026;47:101187. 10.1016/j.ctarc.2026.101187 41966556

[11] Margolis B, Cagle-Colon K, Chen L, Tergas AI, Boyd L, Wright JD. Prognostic significance of lymphovascular space invasion for stage IA1 and IA2 cervical cancer. Int J Gynecol Cancer. 2020;30:735–43. 10.1136/ijgc-2019-000849 32179697

[12] He F, Li W, Liu P, Kang S, Sun L, Zhao H, et al. Influence of uterine corpus invasion on prognosis in stage IA2-IIB cervical cancer: a multicenter retrospective cohort study. Gynecol Oncol. 2020;158:273–81. 10.1016/j.ygyno.2020.05.005 32467057

[13] Liu J, Li Y, Chen X, Xu X, Zhao H, Wang S, et al. Upregulation of miR-205 induces CHN1 expression, which is associated with the aggressive behaviour of cervical cancer cells and correlated with lymph node metastasis. BMC Cancer. 2020;20:1029. 10.1186/s12885-020-07478-w PMC7590479 33109127

[14] Ke Y, Zu S, Chen L, Liu M, Yang H, Wang F, et al. Combination of estrogen receptor alpha and histological type helps to predict lymph node metastasis in patients with stage IA2 to IIA2 cervical cancer. Cancer Manag Res. 2022;14:317–25. 10.2147/CMAR.S343518 PMC8802323 35115830

[15] Hou Y, Zhang L, Wang H, Wang W, Hao M. Development and validation of a risk prediction model for lymph node metastasis in stage IA2-IIA1 cervical cancer based on laboratory parameters. Am J Cancer Res. 2025;15:1081–95. 10.62347/EOXM6715 PMC11982735 40226455

[16] Xu D, Zhao X, Ye D, Huo C, Peng X, Liu Y, et al. A gene-based predictive model for lymph node metastasis in cervical cancer: superior performance over imaging techniques. J Transl Med. 2025;23:397. 10.1186/s12967-025-06327-3 PMC11969859 40181462

[17] Xu D, Wang D, Wang S, Tian Y, Long Z, Ren X. Correlation between squamous cell carcinoma antigen level and the clinicopathological features of early-stage cervical squamous cell carcinoma and the predictive value of squamous cell carcinoma antigen combined with computed tomography scan for lymph node metastasis. Int J Gynecol Cancer. 2017;27:1935–42. 10.1097/IGC.0000000000001112 28914639

[18] Chen Q, Zeng X, Huang D, Qiu X. Identification of differentially expressed miRNAs in early-stage cervical cancer with lymph node metastasis across The Cancer Genome Atlas datasets. Cancer Manag Res. 2018;10:6489–504. 10.2147/CMAR.S183488 PMC6276827 30568508

[19] Chen S, Gao C, Wu Y, Huang Z. Identification of prognostic miRNA signature and lymph node metastasis-related key genes in cervical cancer. Front Pharm. 2020;11:544. 10.3389/fphar.2020.00544 PMC7226536 32457603

[20] Zhang Y, Sun D, Song J, Yang N, Zhang Y. Integrated profiles analysis identified a coding-non-coding signature for predicting lymph node metastasis and prognosis in cervical cancer. Front Cell Dev Biol. 2020;8:631491. 10.3389/fcell.2020.631491 PMC7859285 33553172

[21] Zhao W, Yue F, Jiayu S, Di S, Yun-Yan Z. Function, drug resistance and prognostic effect of AKR1C2 in human cancer. Neoplasma. 2023;70:319–32. 10.4149/neo_2023_230206N66 37498066

[22] Zhu Y, Shen B, Pei X, Liu H, Li G. CT, MRI, and PET imaging features in cervical cancer staging and lymph node metastasis. Am J Transl Res. 2021;13:10536–44. PMC8507065 34650724

[23] Huang BX, Fang F. Progress in the study of lymph node metastasis in early-stage cervical cancer. Curr Med Sci. 2018;38:567–74. 10.1007/s11596-018-1915-0 30128863

[24] Ren J, Li Y, Liu XY, Zhao J, He YL, Jin ZY, et al. Diagnostic performance of ADC values and MRI-based radiomics analysis for detecting lymph node metastasis in patients with cervical cancer: a systematic review and meta-analysis. Eur J Radio. 2022;156:110504. 10.1016/j.ejrad.2022.110504 36108474

[25] Li L, Zhang J, Zhe X, Tang M, Zhang X, Lei X, et al. A meta-analysis of MRI-based radiomic features for predicting lymph node metastasis in patients with cervical cancer. Eur J Radio. 2022;151:110243. 10.1016/j.ejrad.2022.110243 35366583

[26] Shi J, Dong Y, Jiang W, Qin F, Wang X, Cui L, et al. MRI-based peritumoral radiomics analysis for preoperative prediction of lymph node metastasis in early-stage cervical cancer: a multi-center study. Magn Reson Imaging. 2022;88:1–8. 10.1016/j.mri.2021.12.008 34968703

[27] Liu J, Li S, Lin L, Jiang Y, Wan Y, Zhou S, et al. Co-expression network analysis identified atypical chemokine receptor 1 (ACKR1) association with lymph node metastasis and prognosis in cervical cancer. Cancer Biomark. 2020;27:213–23. 10.3233/CBM-190533 PMC12662291 32083574

[28] Guo H, Dai Y, Wang A, Wang C, Sun L, Wang Z. Association between expression of MMP-7 and MMP-9 and pelvic lymph node and para-aortic lymph node metastasis in early cervical cancer. J Obstet Gynaecol Res. 2018;44:1274–83. 10.1111/jog.13659 29767419

[29] Hao Y, Liu Q, Wang B, Zhang W, Jiao X, Zhang T, et al. Proteomics-based mapping of the lymph node metastasis landscape in cervical cancer. J Proteome Res. 2025;24:5226–41. 10.1021/acs.jproteome.5c00570 40932219

[30] Zhang L, Zhan X, Yan D, Wang Z. Circulating MicroRNA-21 is involved in lymph node metastasis in cervical cancer by targeting RASA1. Int J Gynecol Cancer. 2016;26:810–6. 10.1097/IGC.0000000000000694 27101583

[31] Jonnalagadda SK, Duan L, Dow LF, Boligala GP, Kosmacek E, McCoy K, et al. Coumarin-based Aldo-Keto reductase family 1C (AKR1C) 2 and 3 inhibitors. ChemMedChem. 2024;19:e202400081. 10.1002/cmdc.202400081 PMC11537819 38976686

[32] Carmona AV, Jonnalagadda S, Case AM, Maddeboina K, Jonnalagadda SK, Dow LF, et al. Discovery of an Aldo-Keto reductase 1C3 (AKR1C3) degrader. Commun Chem. 2024;7:95. 10.1038/s42004-024-01177-4 PMC11059152 38684887

[33] Zhang WF, Li T, Lin SX. Meta-Analysis of steroid-converting enzymes and related receptors in prostate cancer suggesting novel combined therapies. J Steroid Biochem Mol Biol. 2020;198:105559. 10.1016/j.jsbmb.2019.105559 31783154

[34] Chen S, Youhong T, Tan Y, He Y, Ban Y, Cai J, et al. EGFR-PKM2 signaling promotes the metastatic potential of nasopharyngeal carcinoma through induction of FOSL1 and ANTXR2. Carcinogenesis. 2020;41:723–33. 10.1093/carcin/bgz180 PMC7351130 31665243

[35] Zhao SF, Wang SG, Zhao ZY, Li WL. AKR1C1-3, notably AKR1C3, are distinct biomarkers for liver cancer diagnosis and prognosis: Database mining in malignancies. Oncol Lett. 2019;18:4515–22. 10.3892/ol.2019.10802 PMC6781771 31611960

[36] Berrandou T, Mulot C, Cordina-Duverger E, Arveux P, Laurent-Puig P, Truong T, et al. Association of breast cancer risk with polymorphisms in genes involved in the metabolism of xenobiotics and interaction with tobacco smoking: a gene-set analysis. Int J Cancer. 2019;144:1896–908. 10.1002/ijc.31917 30303517

[37] Shiiba M, Yamagami H, Yamamoto A, Minakawa Y, Okamoto A, Kasamatsu A, et al. Mefenamic acid enhances anticancer drug sensitivity via inhibition of aldo-keto reductase 1C enzyme activity. Oncol Rep. 2017;37:2025–32. 10.3892/or.2017.5480 28259989

[38] Kyuno D, Asano H, Okumura R, Takasawa K, Takasawa A, Konno T, et al. The Role of Claudin-1 in enhancing pancreatic cancer aggressiveness and drug resistance via metabolic pathway modulation. Cancers (Basel). 2025;17:1469. 10.3390/cancers17091469 PMC12070999 40361399

[39] Xing S, Liu Y, Xie H, Guo C, Wang X, Lv B, et al. Discovery of highly potent AKR1Cs pan-inhibitors as chemotherapeutic potentiators to restore breast cancer drug resistance. Eur J Med Chem. 2025;289:117413. 10.1016/j.ejmech.2025.117413 40015157

[40] Cui Y, Liu Z. AKR1C2 silencing promotes ferroptosis and inhibits proliferation, migration, and invasion in lung cancer cells. PLoS One. 2025;20:e0325995. 10.1371/journal.pone.0325995 PMC12176218 40531841

[41] Zhou Y, Guo Y, Wang Y. Identification and validation of a seven-gene prognostic marker in colon cancer based on single-cell transcriptome analysis. IET Syst Biol. 2022;16:72–83. 10.1049/syb2.12041 PMC8965382 35352485

[42] Ren F, Jin Q, Liu T, Ren X, Zhan Y. Proteome-wide mendelian randomization study implicates therapeutic targets in common cancers. J Transl Med. 2023;21:646. 10.1186/s12967-023-04525-5 PMC10512580 37735436

[43] Hwang S, Kim HE, Min M, Raghunathan R, Panova IP, Munshi R, et al. Epigenetic silencing of SPINT2 promotes cancer cell motility via HGF-MET pathway activation in melanoma. J Invest Dermatol. 2015;135:2283–91. 10.1038/jid.2015.160 PMC4537358 25910030

[44] Nie M, Li T, Liu P, Wang X. Therapeutic potential of targeting AKR1C2 in the treatment of prostate cancer. Mol Biol Rep. 2024;51:994. 10.1007/s11033-024-09917-4 39292292

[45] Zhu Z, Zhang Y, Zhang X, Chen Q, Tang S, Zhou X, et al. Potential molecular metabolic mechanisms underlying the effects of cimifugin in gastric cancer through single-cell and bulk RNA sequencing combined with network pharmacology. J Gastrointest Oncol. 2024;15:1409–30. 10.21037/jgo-24-413 PMC11399845 39279957

[46] Wang N, Che Y, Yin F, Yu F, Bi X, Wang Y. Study on the methylation status of SPINT2 gene and its expression in cervical carcinoma. Cancer Biomark. 2018;22:435–42. 10.3233/CBM-171050 PMC13078461 29843210

[47] Zhu KG, Yang J, Zhu Y, Zhu Q, Pan W, Deng S, et al. The microprotein encoded by exosomal lncAKR1C2 promotes gastric cancer lymph node metastasis by regulating fatty acid metabolism. Cell Death Dis. 2023;14:708. 10.1038/s41419-023-06220-1 PMC10616111 37903800

[48] Pecorelli S, Zigliani L, Odicino F. Revised FIGO staging for carcinoma of the cervix. Int J Gynaecol Obstet. 2009;105:107–8. 10.1016/j.ijgo.2009.02.009 19342051

[49] Buczak K, Kirkpatrick JM, Truckenmueller F, Santinha D, Ferreira L, Roessler S, et al. Spatially resolved analysis of FFPE tissue proteomes by quantitative mass spectrometry. Nat Protoc. 2020;15:2956–79. 10.1038/s41596-020-0356-y 32737464

[50] Zhang E, Zhang Y, Fan Z, Cheng L, Han S, Che H. Apigenin inhibits histamine-induced cervical cancer tumor growth by regulating estrogen receptor expression. Molecules. 2020;25:1960. 10.3390/molecules25081960 PMC7221565 32340124

[51] Wei WF, Han LF, Liu D, Wu LF, Chen XJ, Yi HY, et al. Orthotopic xenograft mouse model of cervical cancer for studying the role of MicroRNA-21 in promoting lymph node metastasis. Int J Gynecol Cancer. 2017;27:1587–95. 10.1097/IGC.0000000000001059 28945212

[52] Tang Z, Li C, Kang B, Gao G, Li C, Zhang Z. GEPIA: a web server for cancer and normal gene expression profiling and interactive analyses. Nucleic Acids Res. 2017;45:W98–102. 10.1093/nar/gkx247 PMC5570223 28407145

