# Supplementary material for: AKR1C2 promotes lymph node metastasis in cervical cancer via a non-canonical nuclear function
Source: Cell Death Discov. 2026 Aug 13;12:450. doi: 10.1038/s41420-026-03287-5 (PMC13752304; doi:10.1038/s41420-026-03287-5)
Supplement: Supplementary file 1 [file 41420_2026_3287_MOESM15_ESM.pdf]

Figure 6D

HeLa-si-AKR1C2

SPINT2(34KDa)

GAPDH(38KDa)

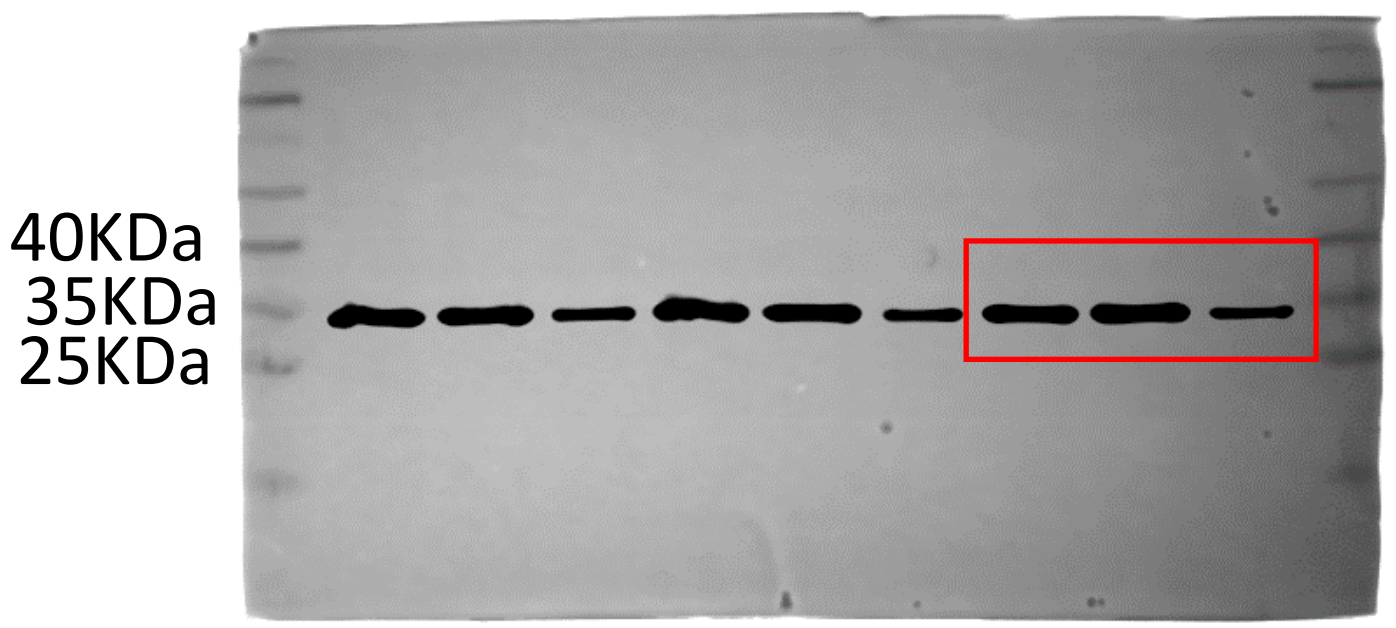

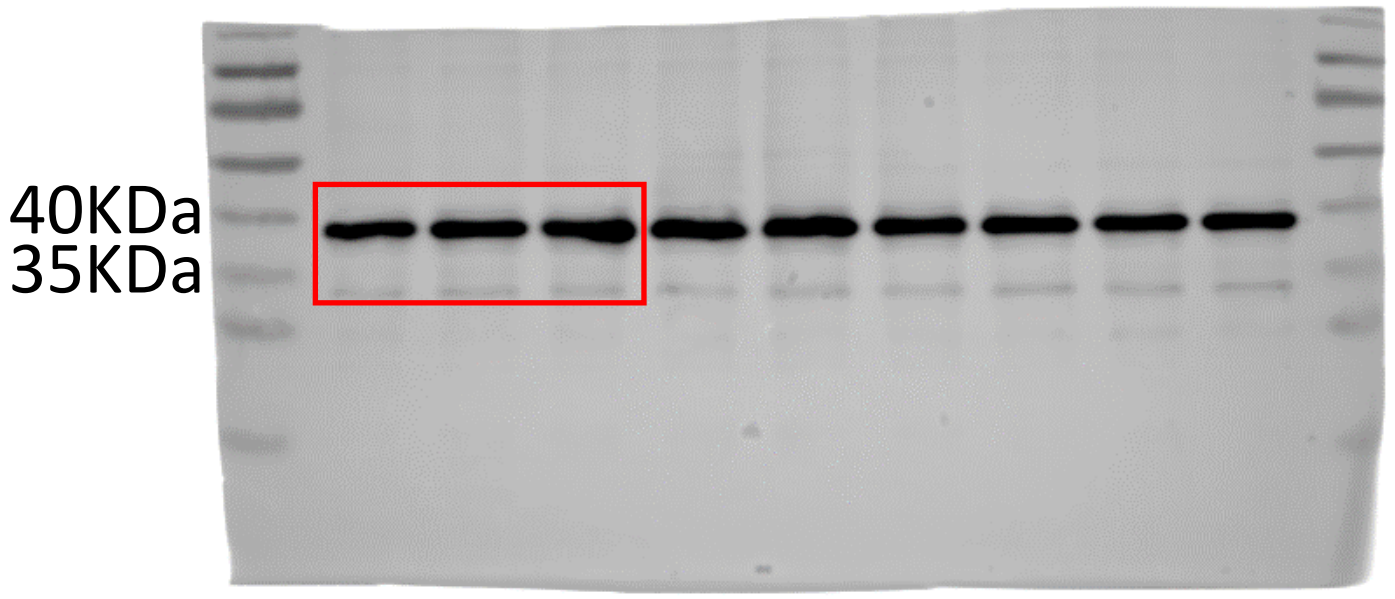

Figure 6D

HeLa-OE-AKR1C2

SPINT2(34KDa)

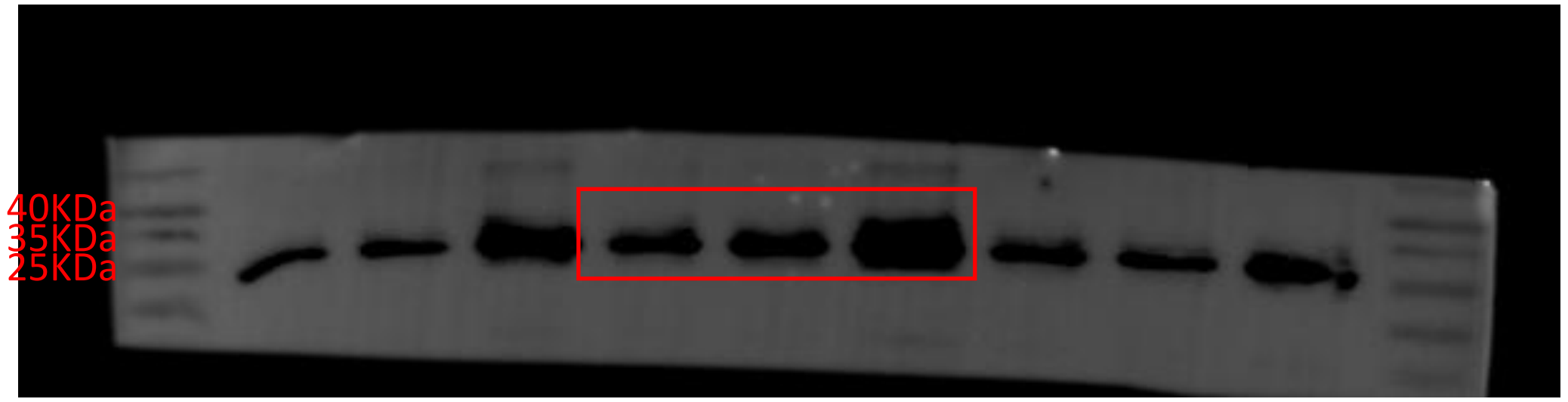

GAPDH(38KDa)

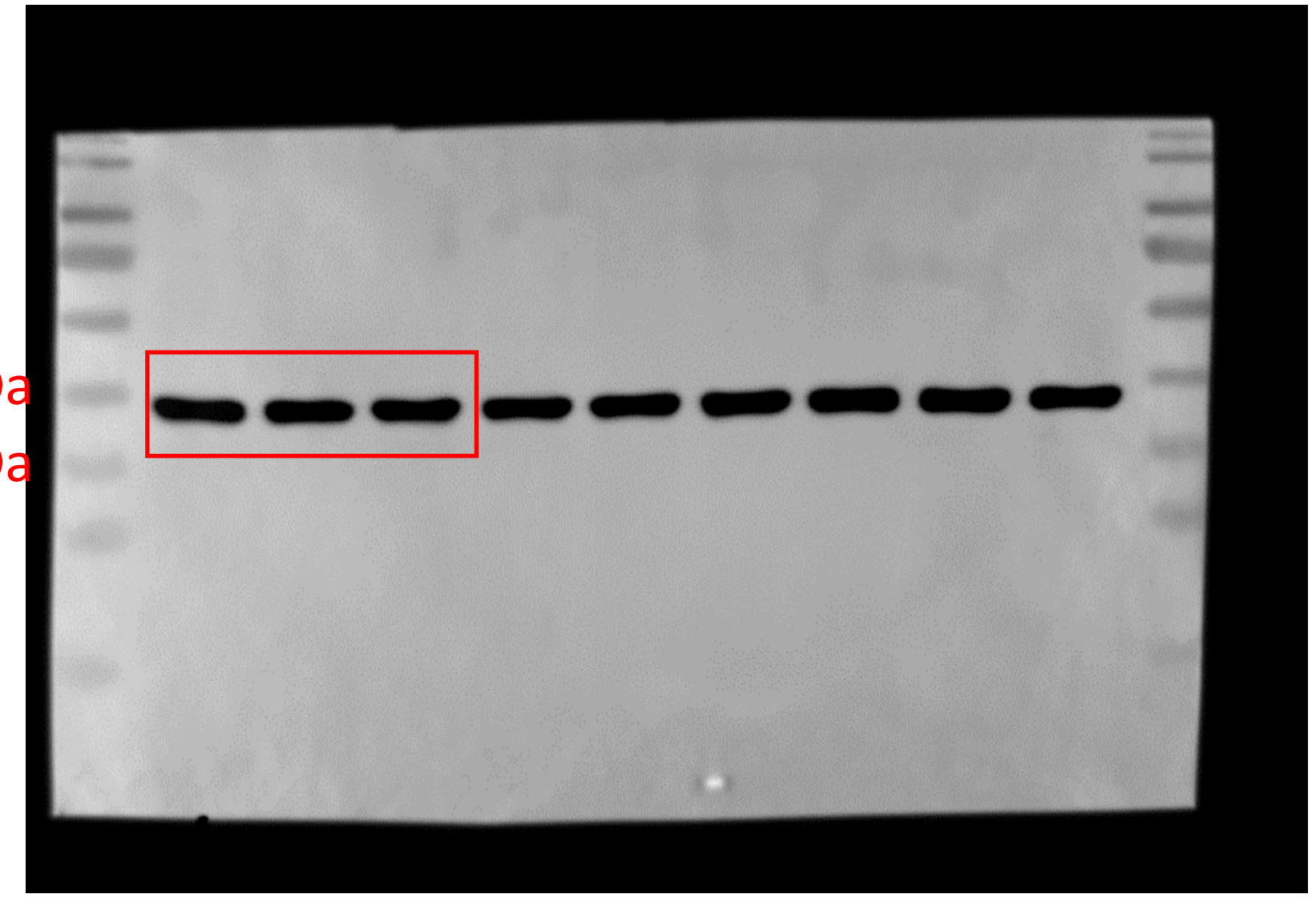

Figure 6E

SiHa-si-AKR1C2

SPINT2(34KDa)

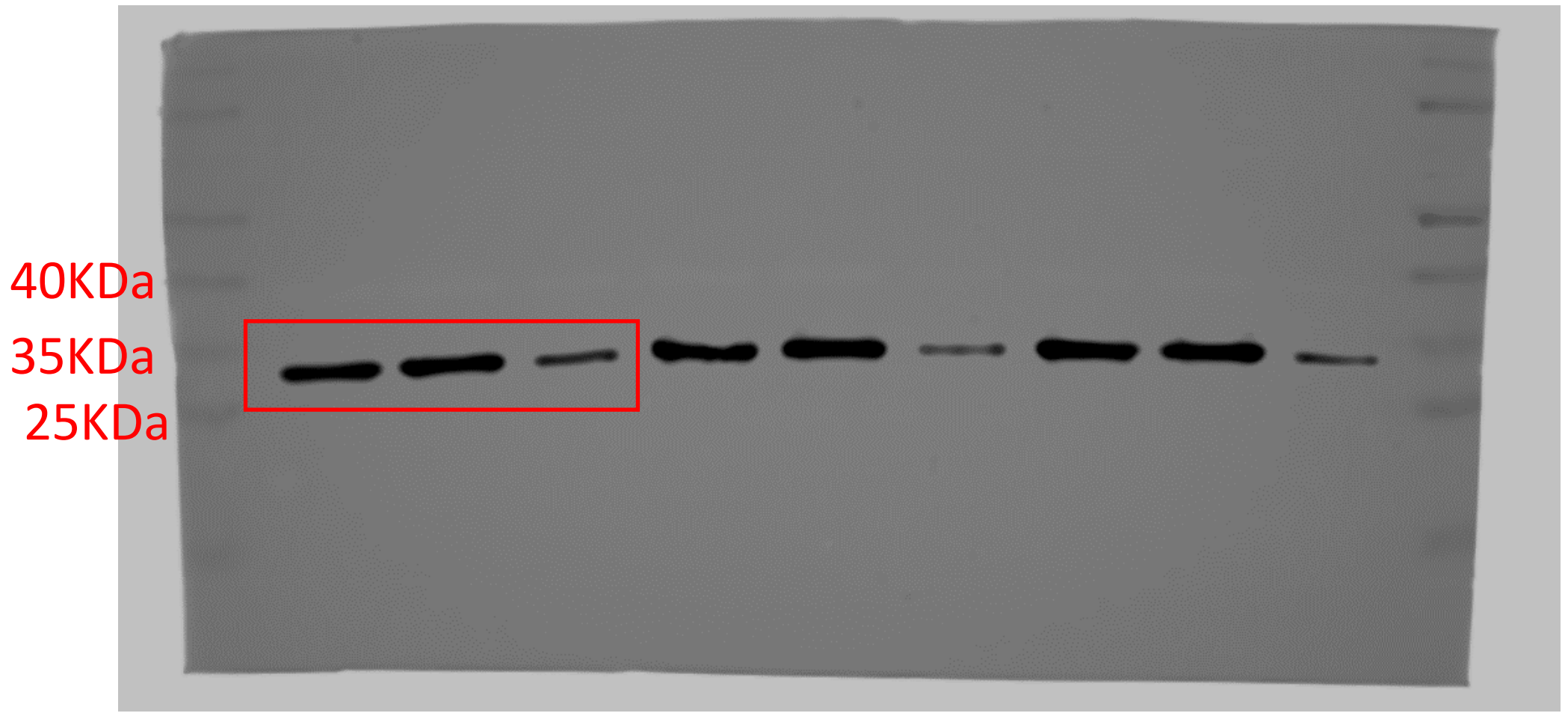

GAPDH(38KDa)

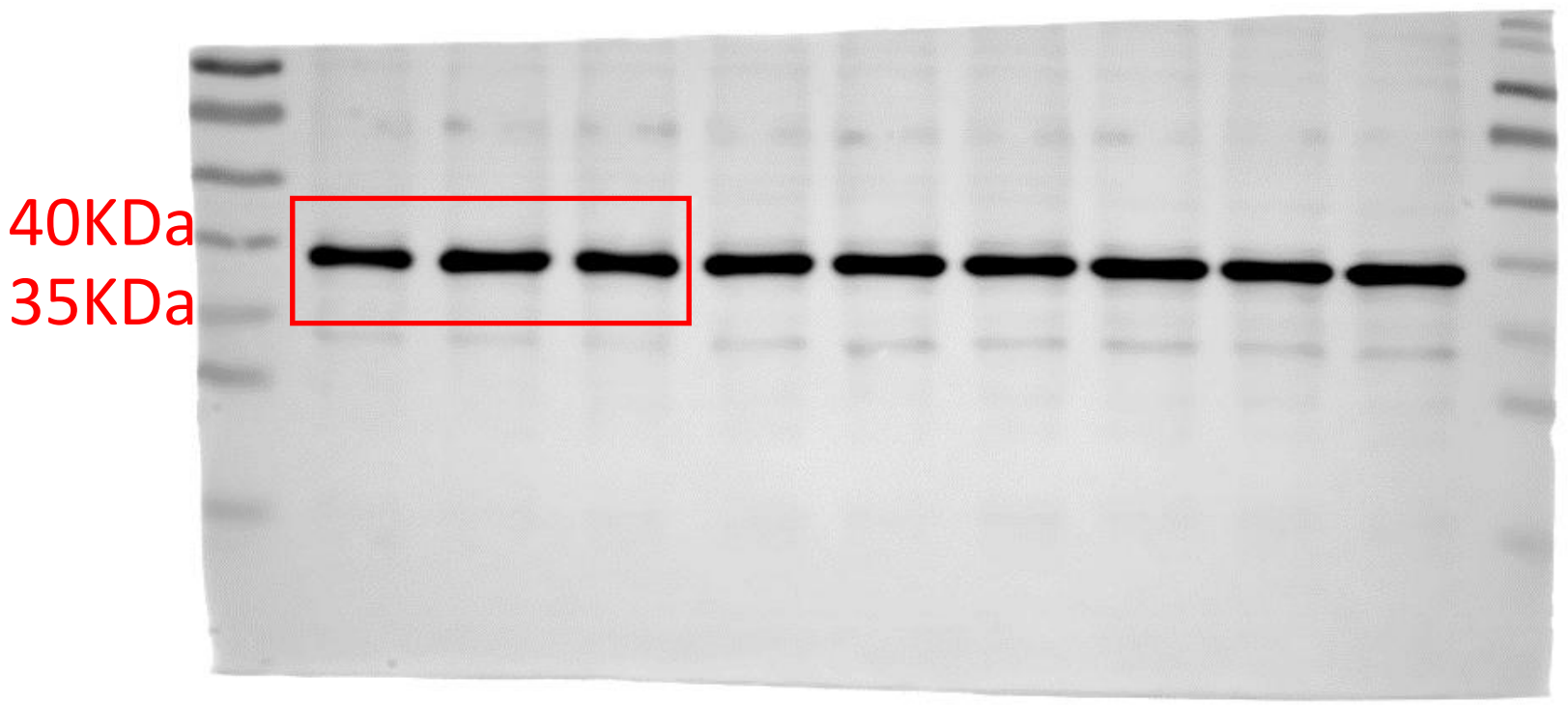

Figure 6E

SiHa-OE-AKR1C2

SPINT2(34KDa)

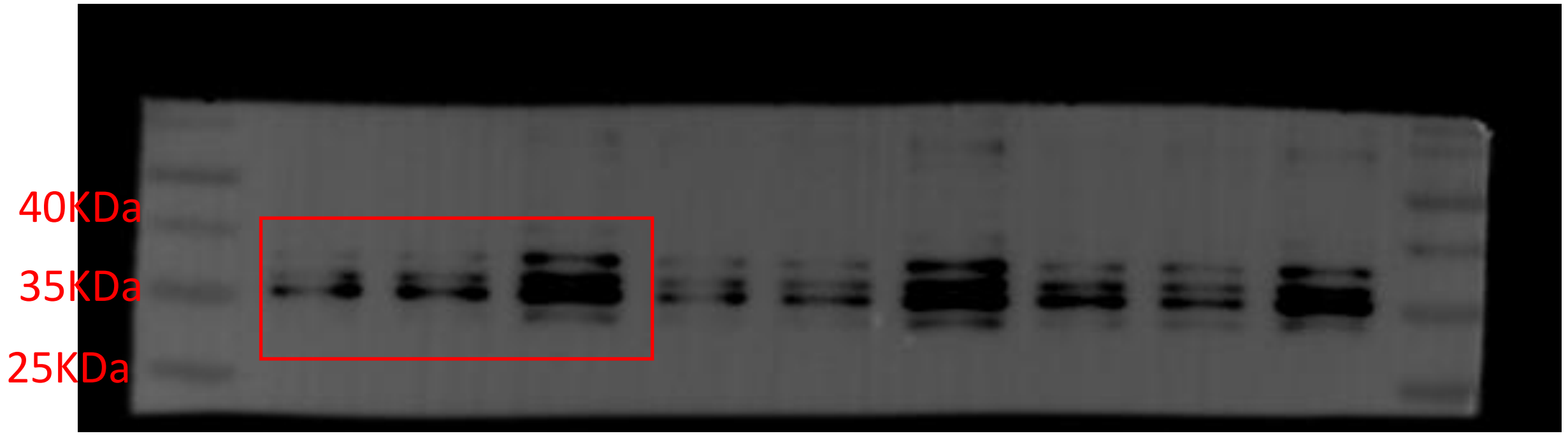

GAPDH(38KDa)

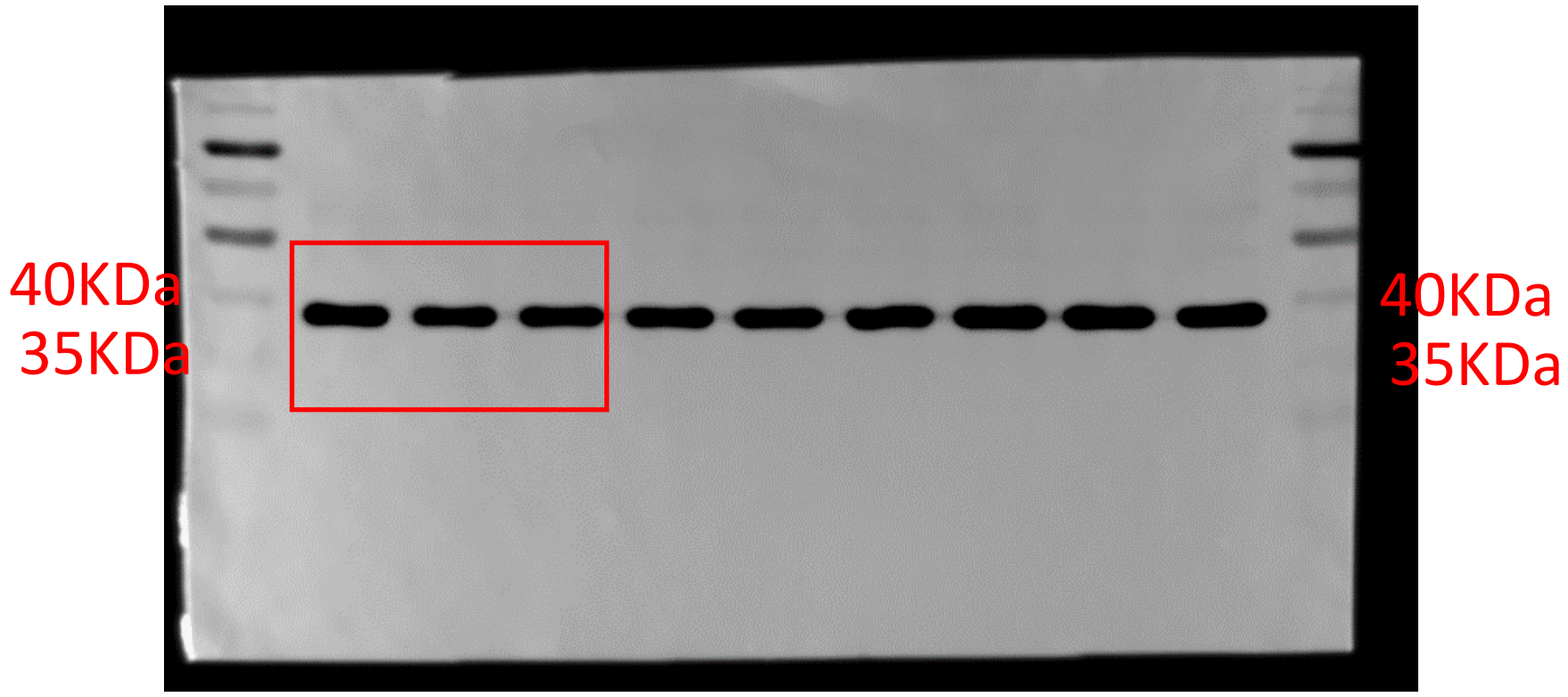

Figure 6F

Hela-si-SPINT2

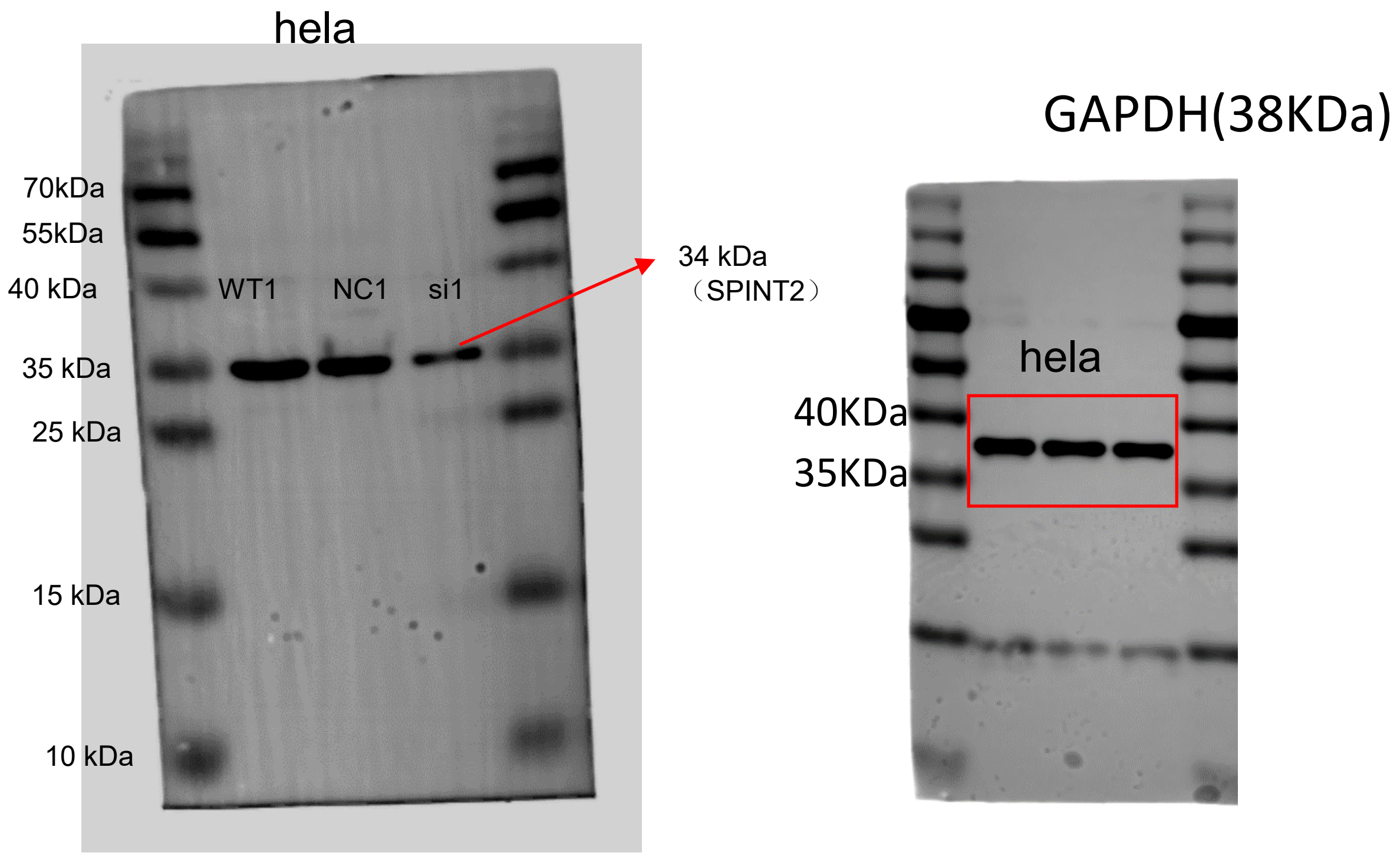

Figure 6F

Hela-OE-SPINT2

SPINT2(34KDa)

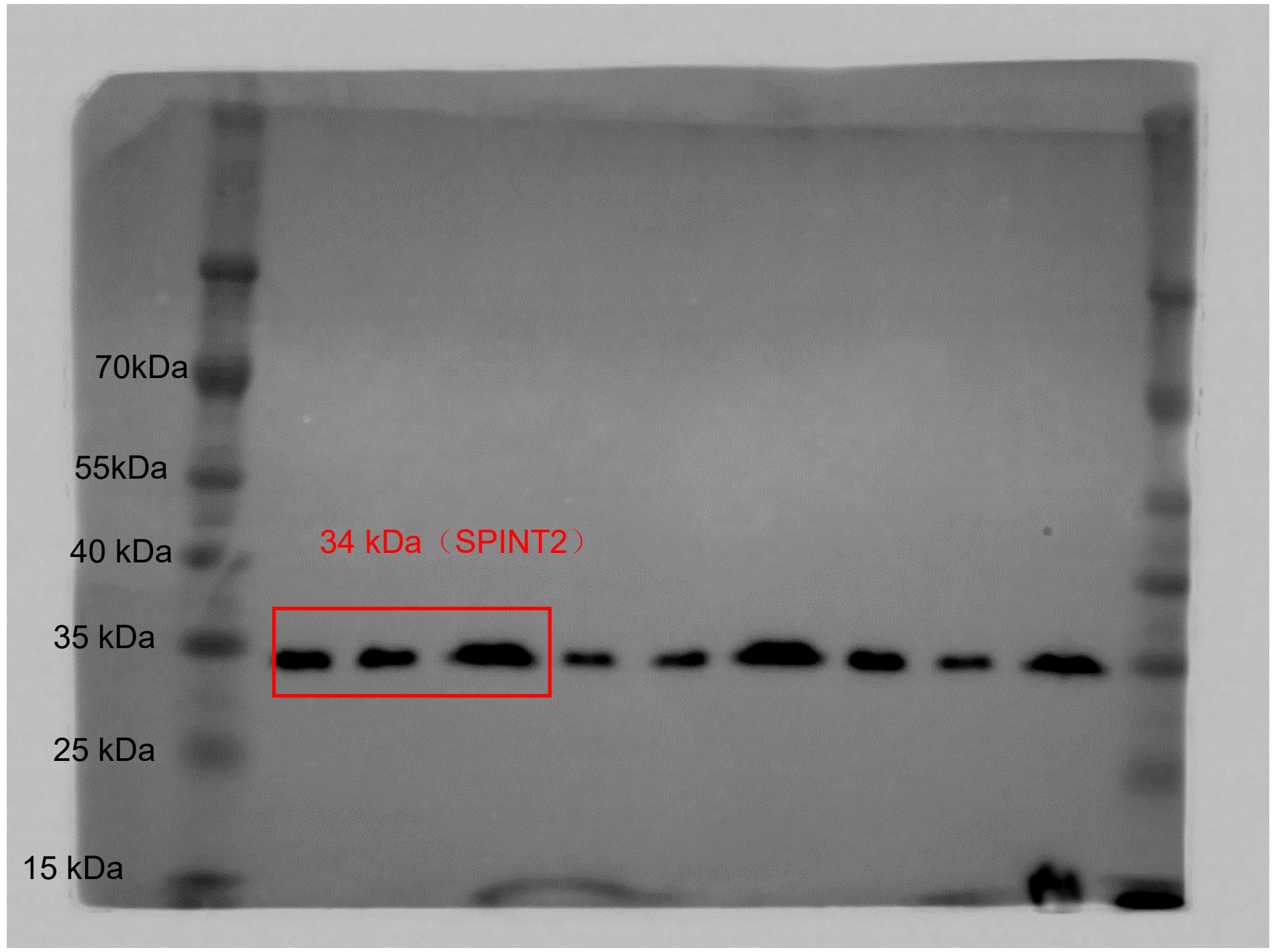

GAPDH(38KDa)

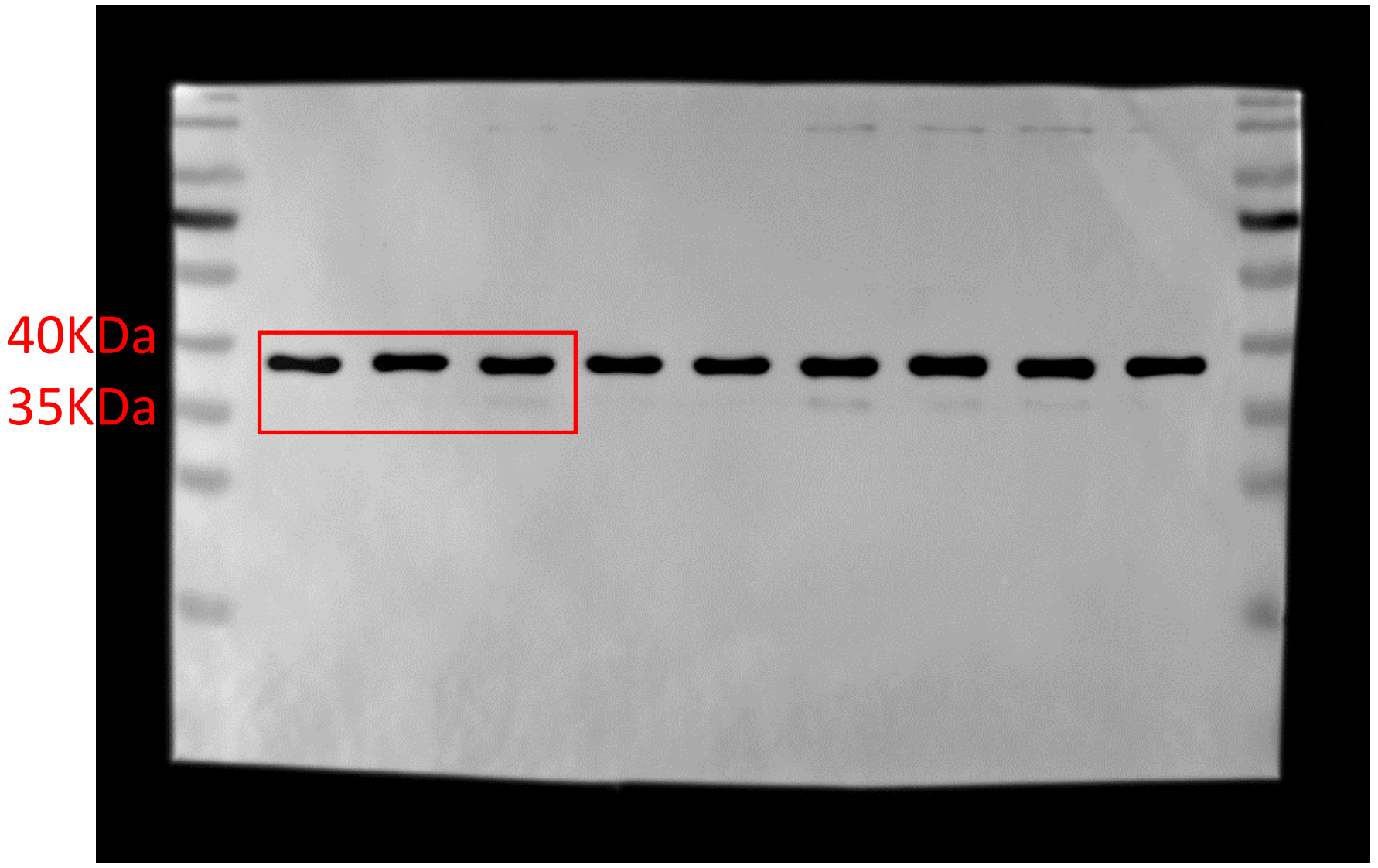

Figure 6G

SiHa-si-SPINT2

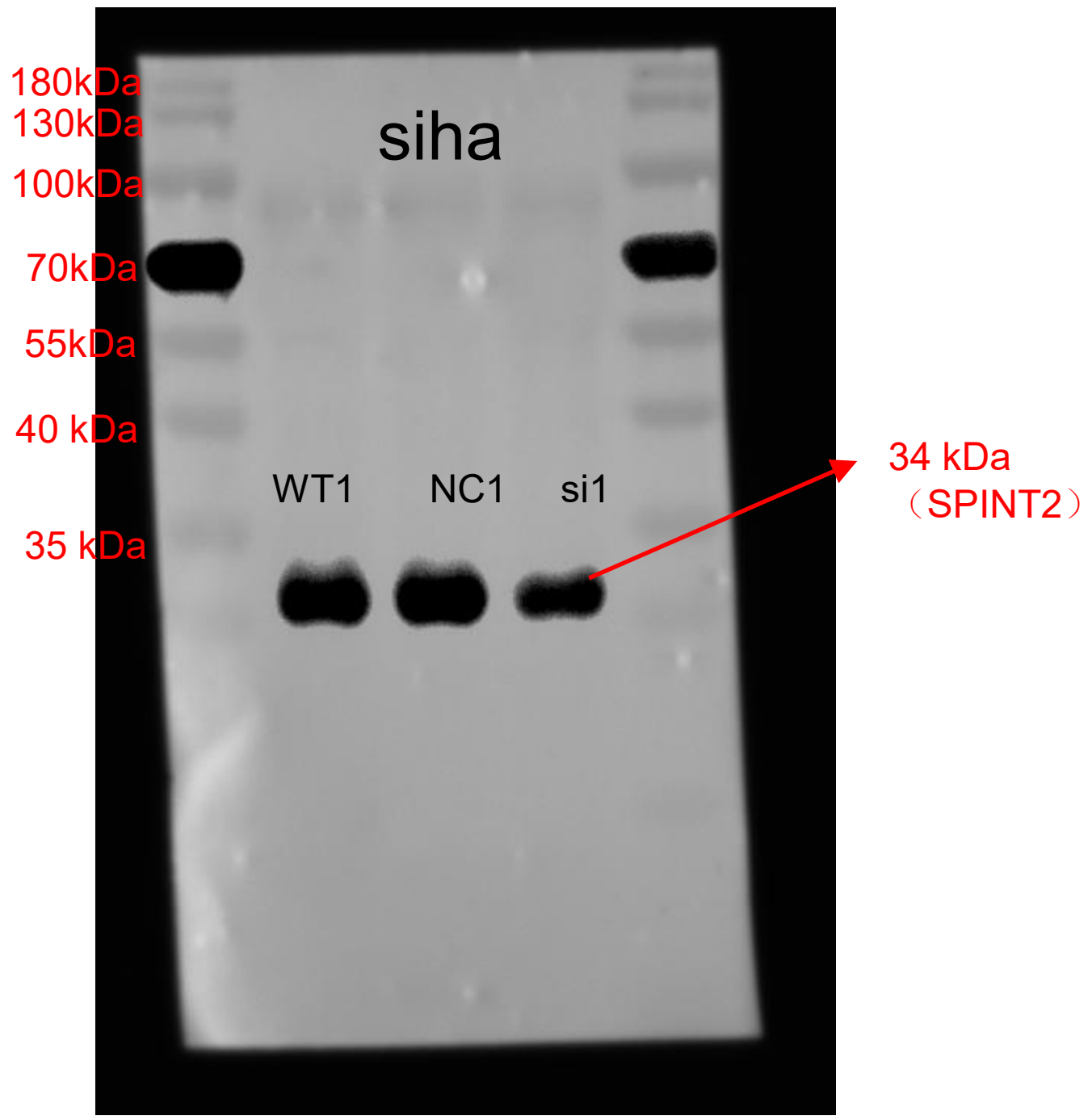

GAPDH(38KDa)

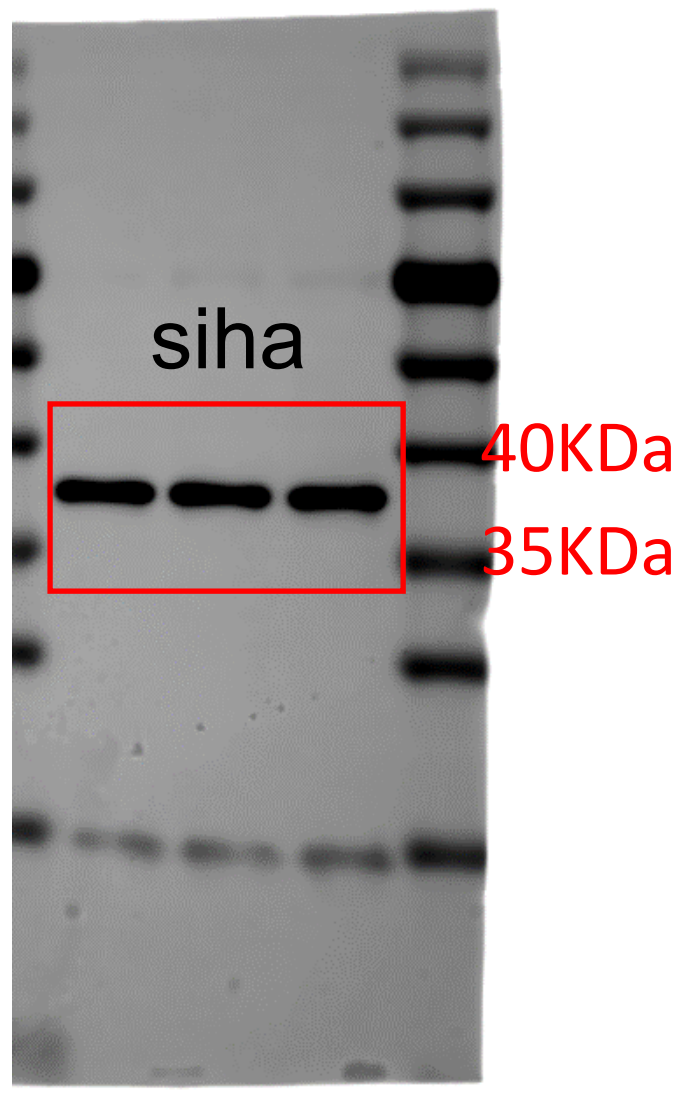

Figure 6G

SiHa-OE-SPINT2

SPINT2(34KDa)

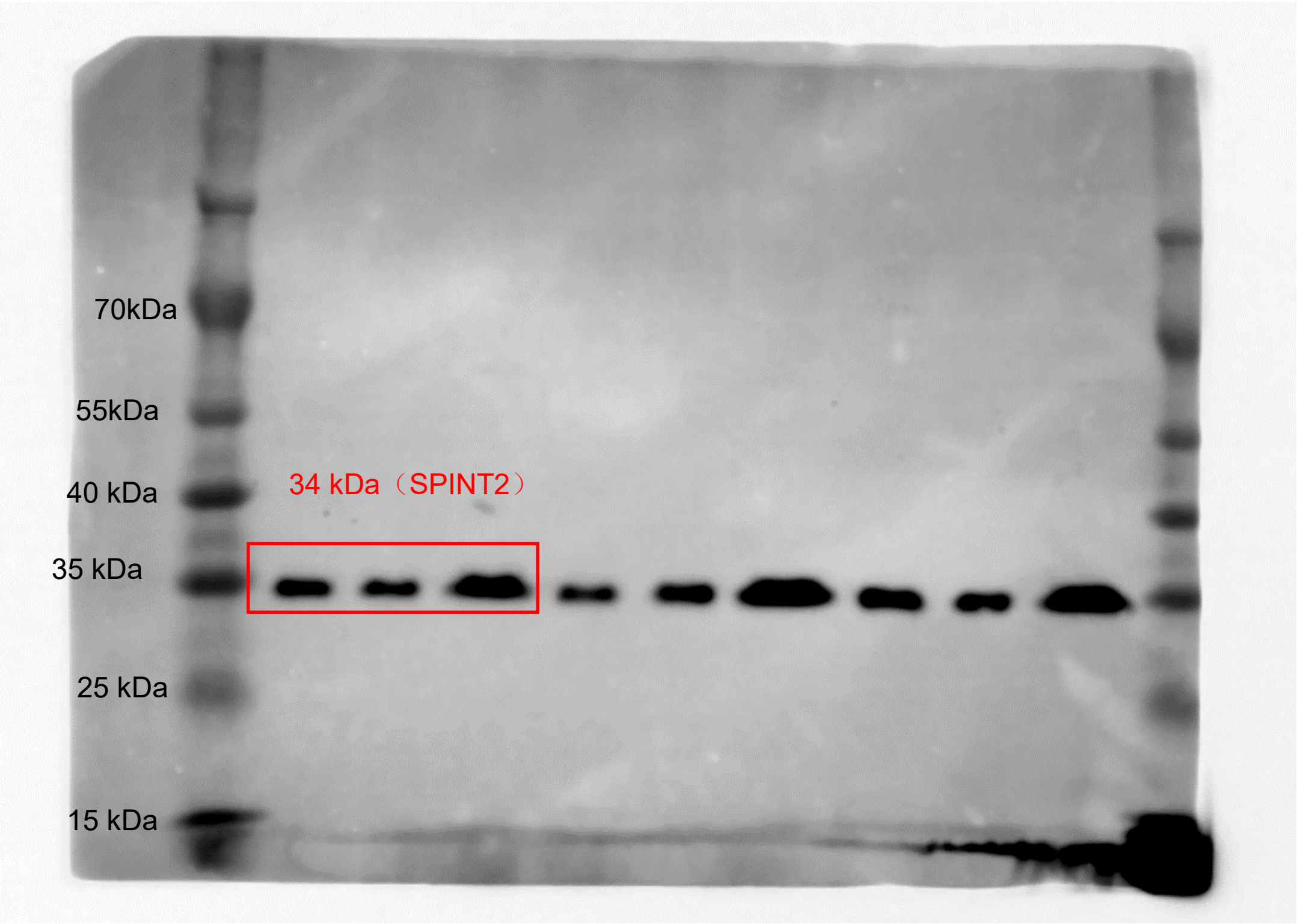

GAPDH(38KDa)

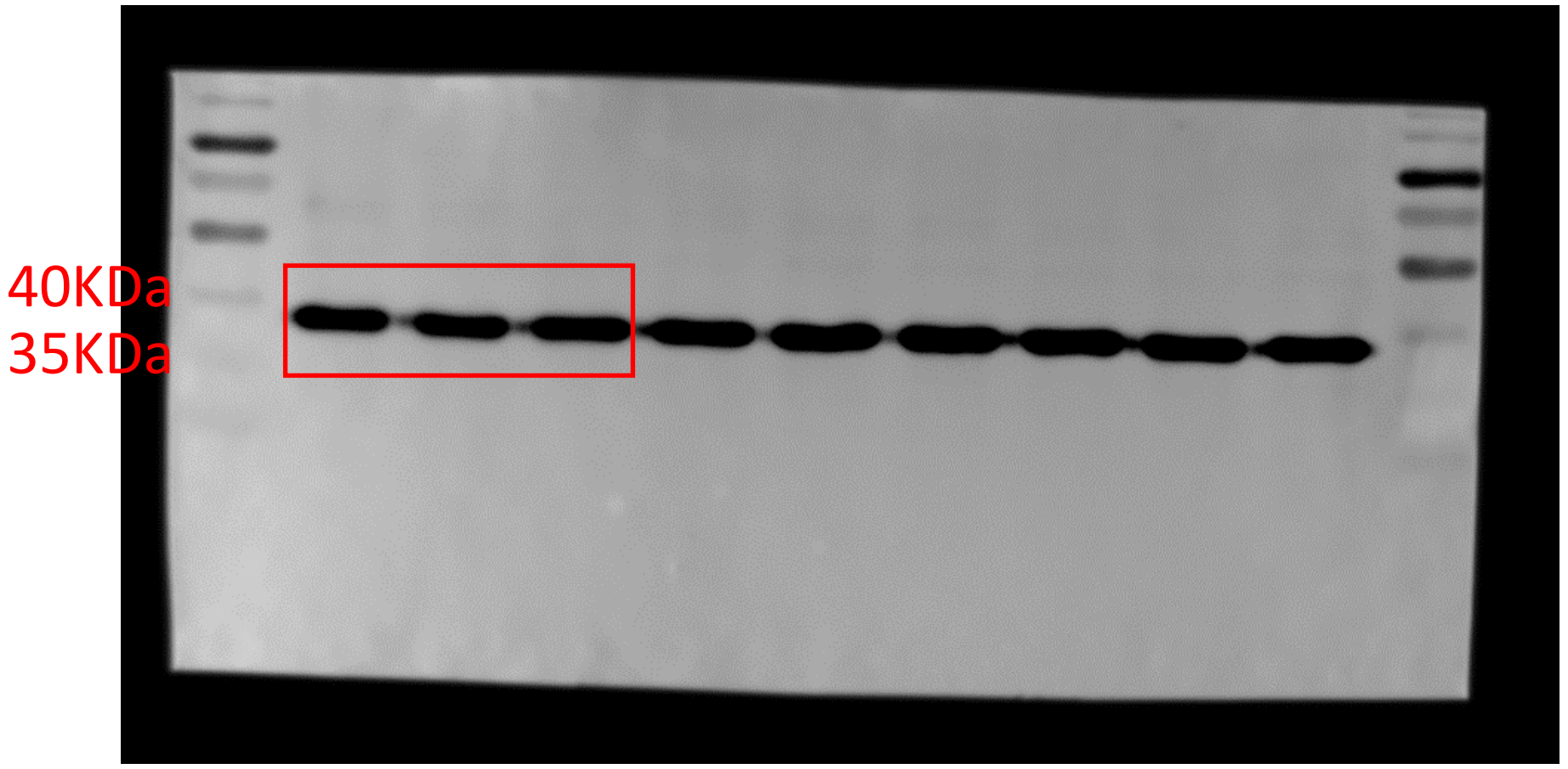

Figure 7B

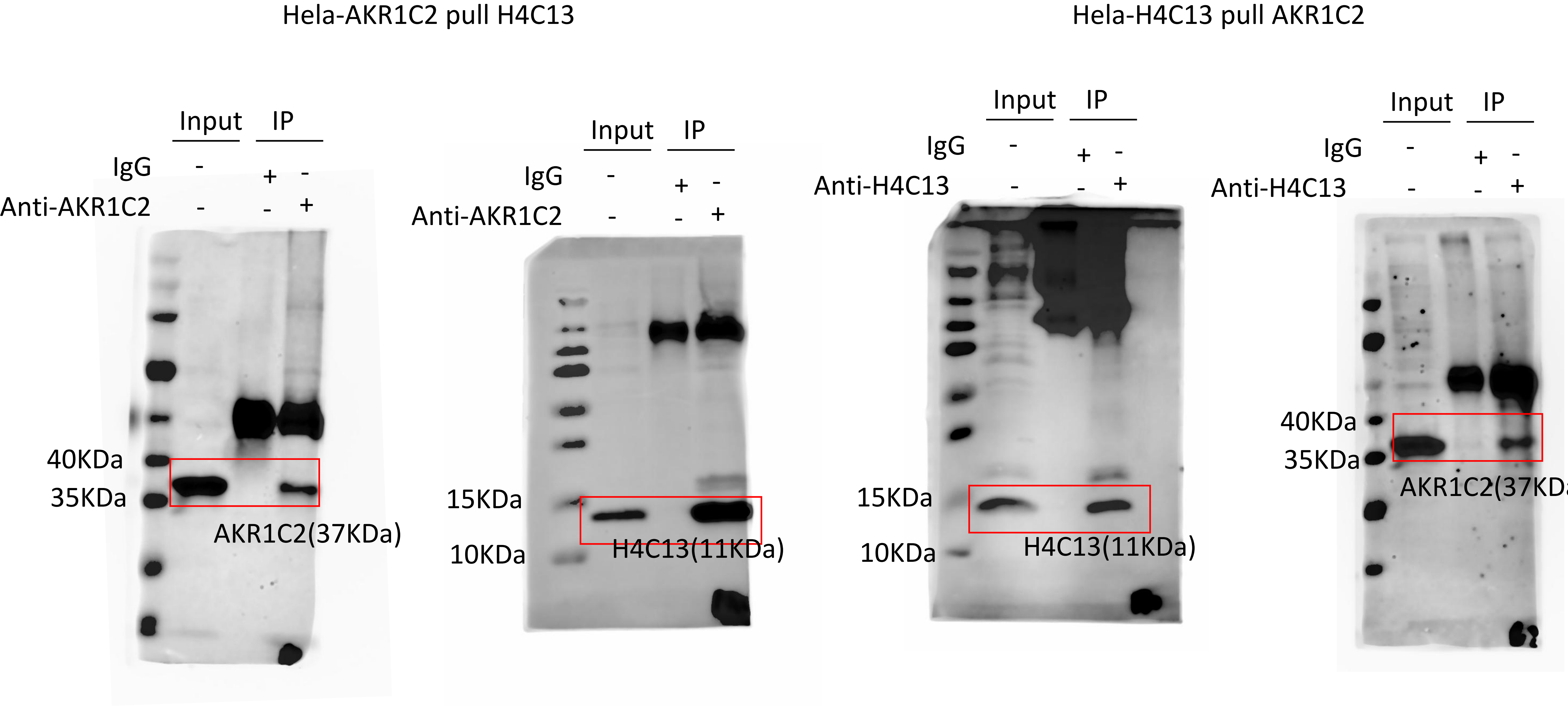
Hela-AKR1C2 pull H4C13
Hela-H4C13 pull AKR1C2
Input
IP
IgG
Anti-AKR1C2
40KDa
35KDa
AKR1C2(37KDa)
15KDa
10KDa
H4C13(11KDa)
Anti-H4C13

Figure 7B

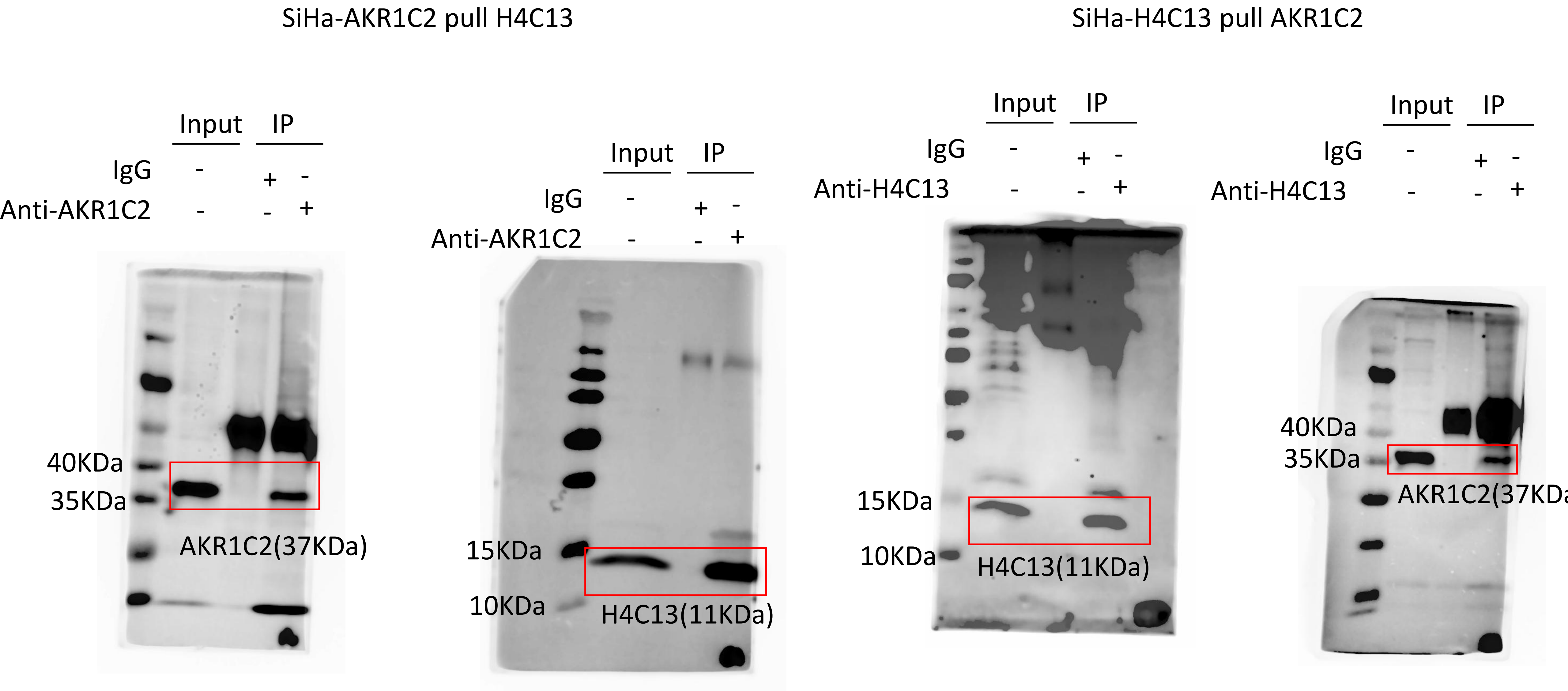
SiHa-AKR1C2 pull H4C13
SiHa-H4C13 pull AKR1C2
Input
IP
IgG
Anti-AKR1C2
- + -
- - +
40KDa
35KDa
AKR1C2(37KDa)
Input
IP
IgG
Anti-AKR1C2
15KDa
10KDa
H4C13(11KDa)
Input
IP
IgG
Anti-H4C13
15KDa
10KDa
H4C13(11KDa)
Input
IP
IgG
Anti-H4C13
40KDa
35KDa

Figure 7E

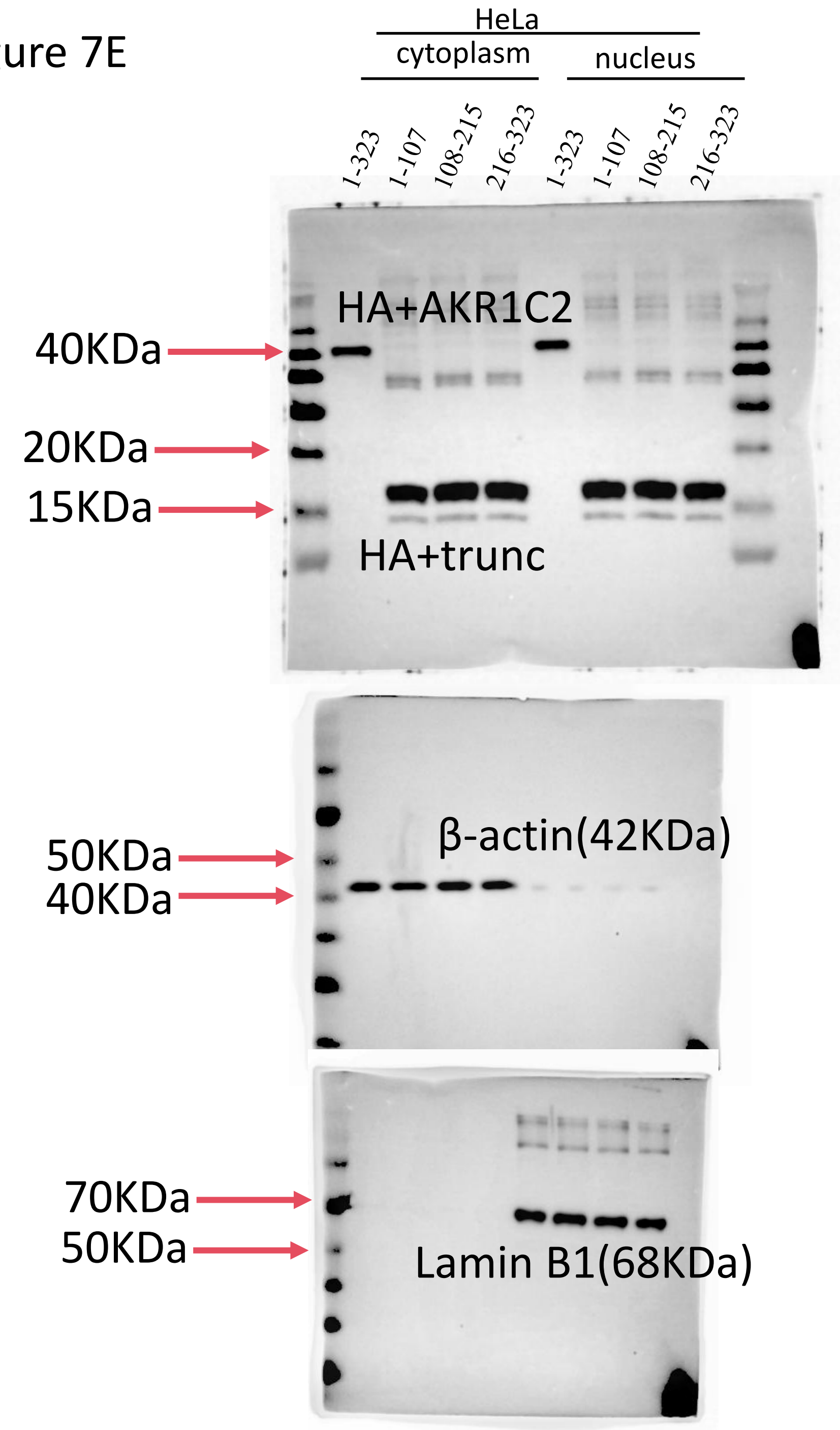

Figure 7F

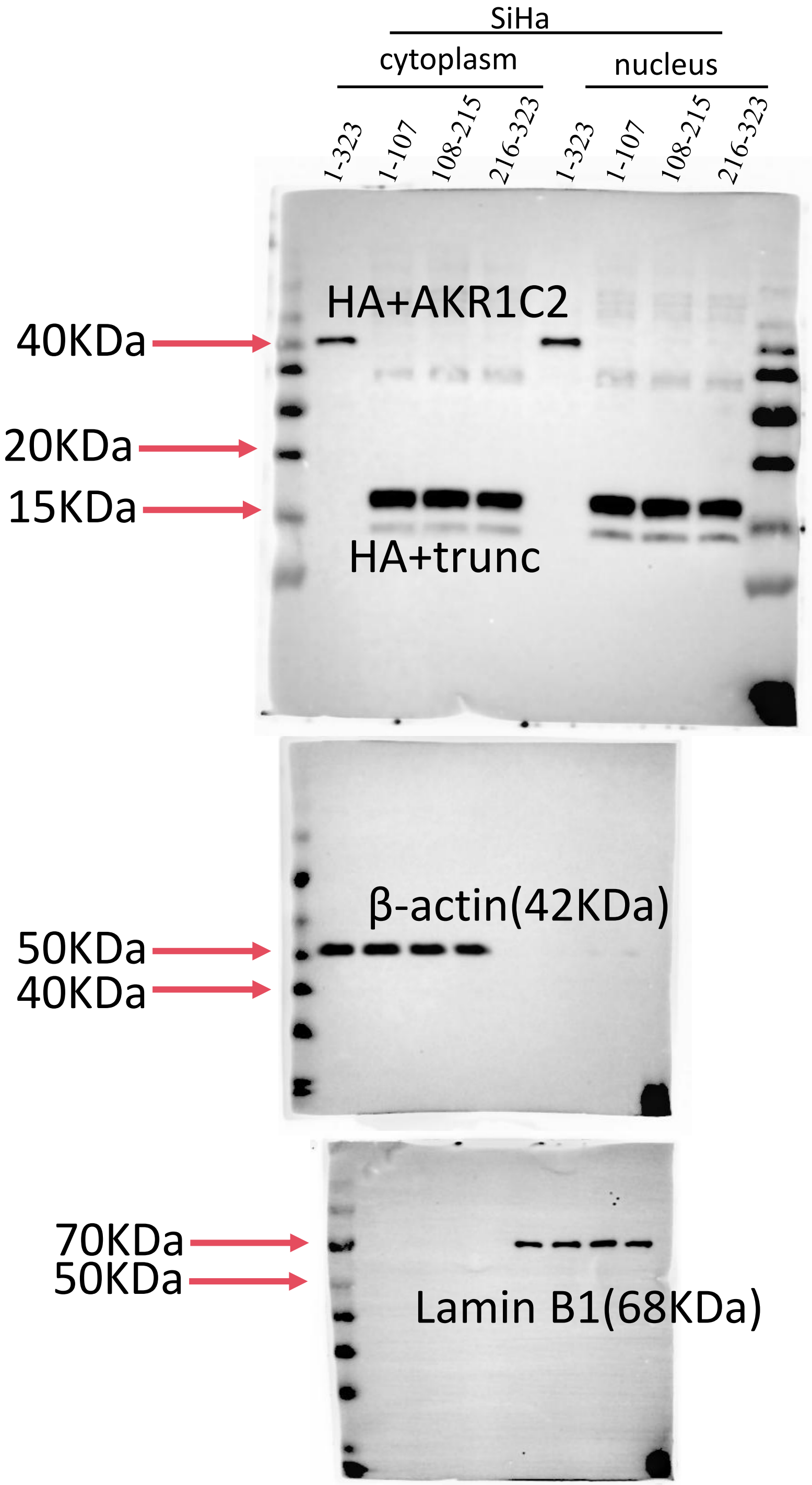

Figure 7G

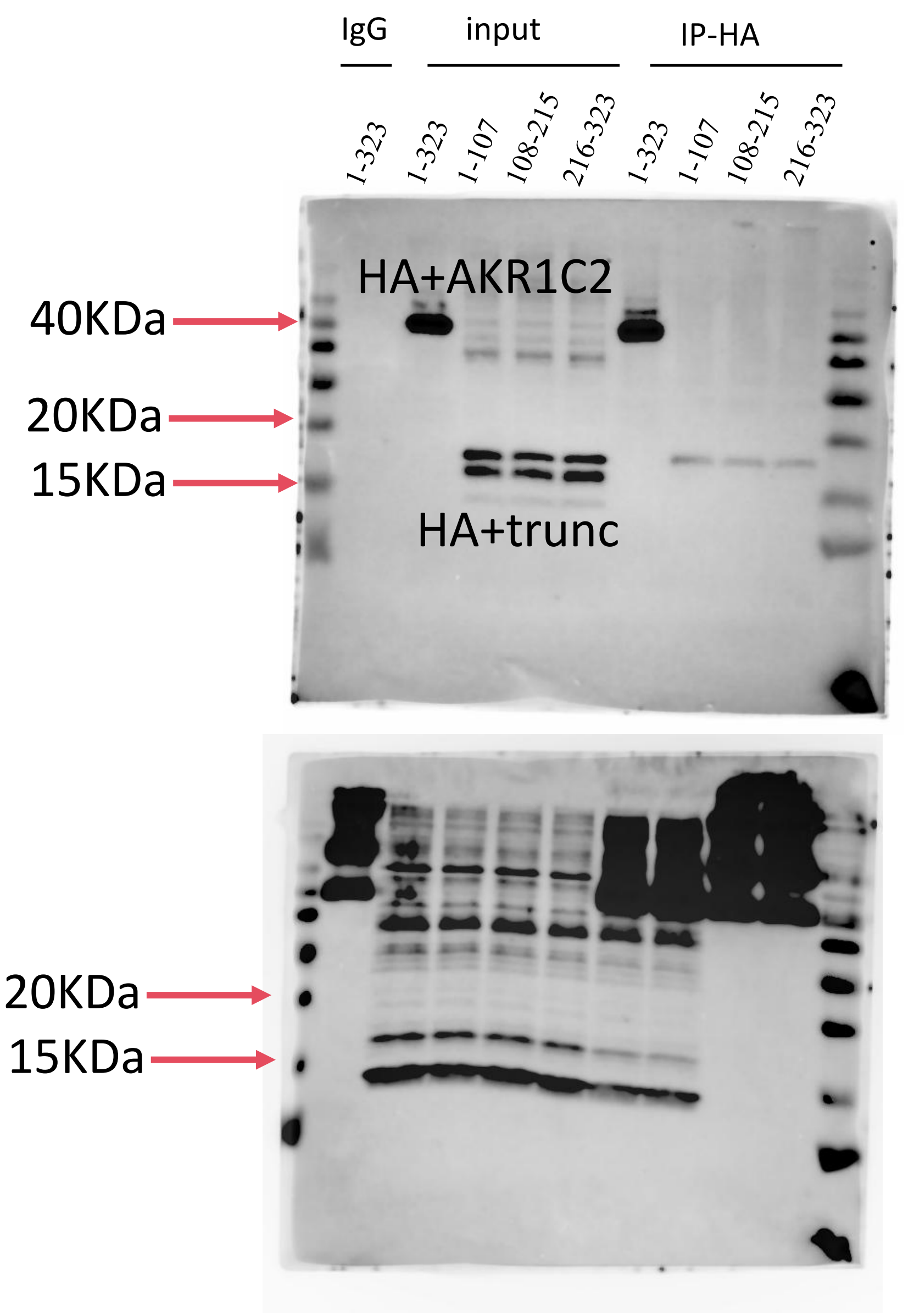

Figure 7H

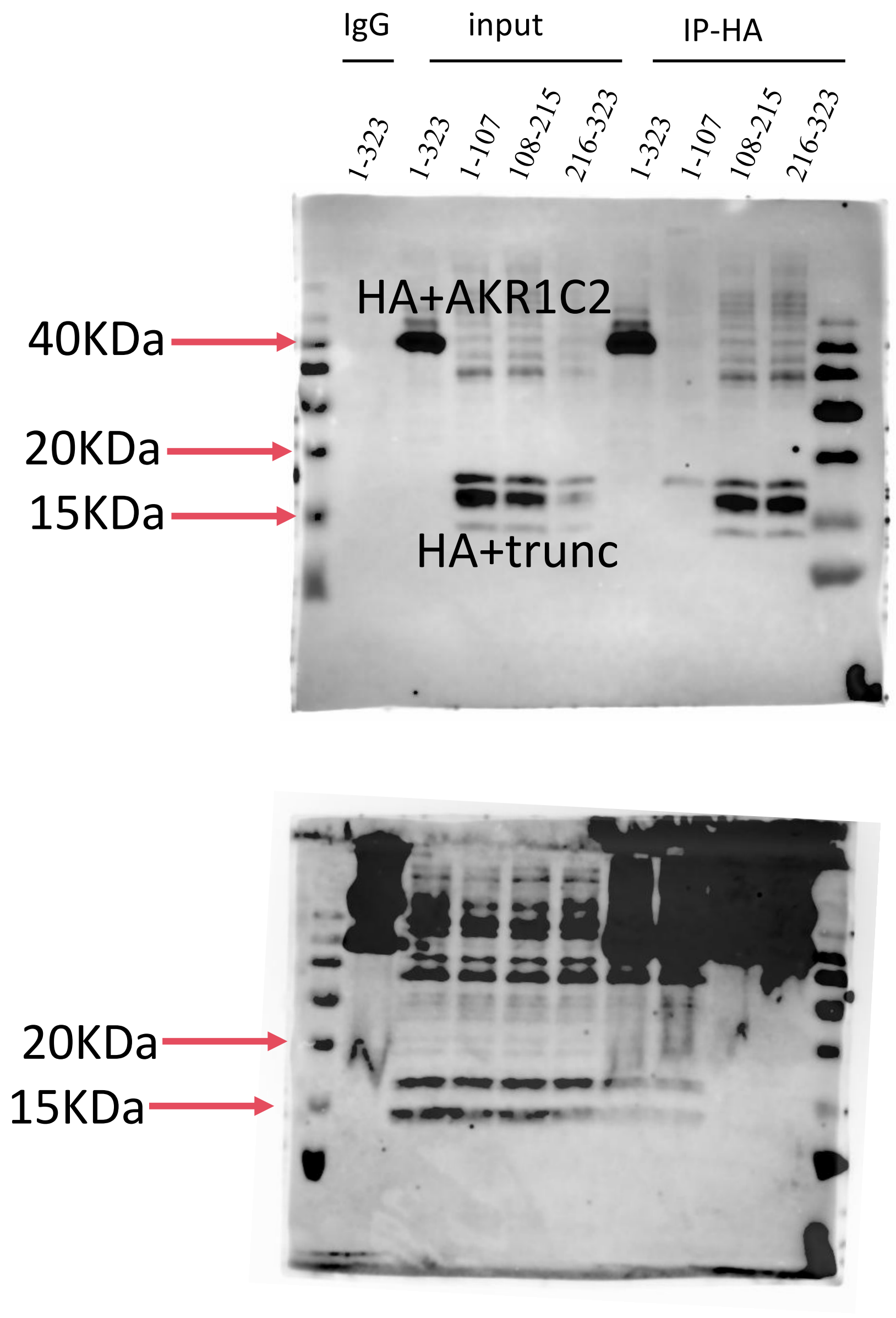

Figure 8A

HeLa-si-H4C13

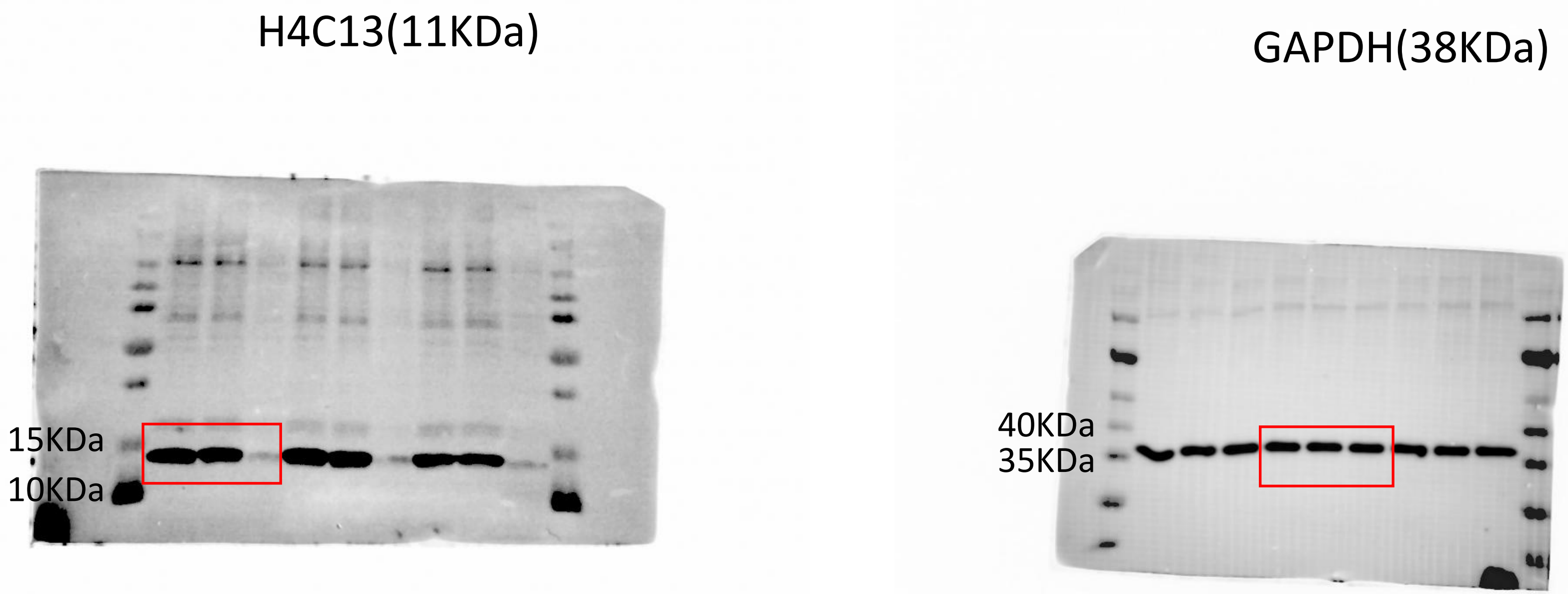

Figure 8A

HeLa-OE-H4C13

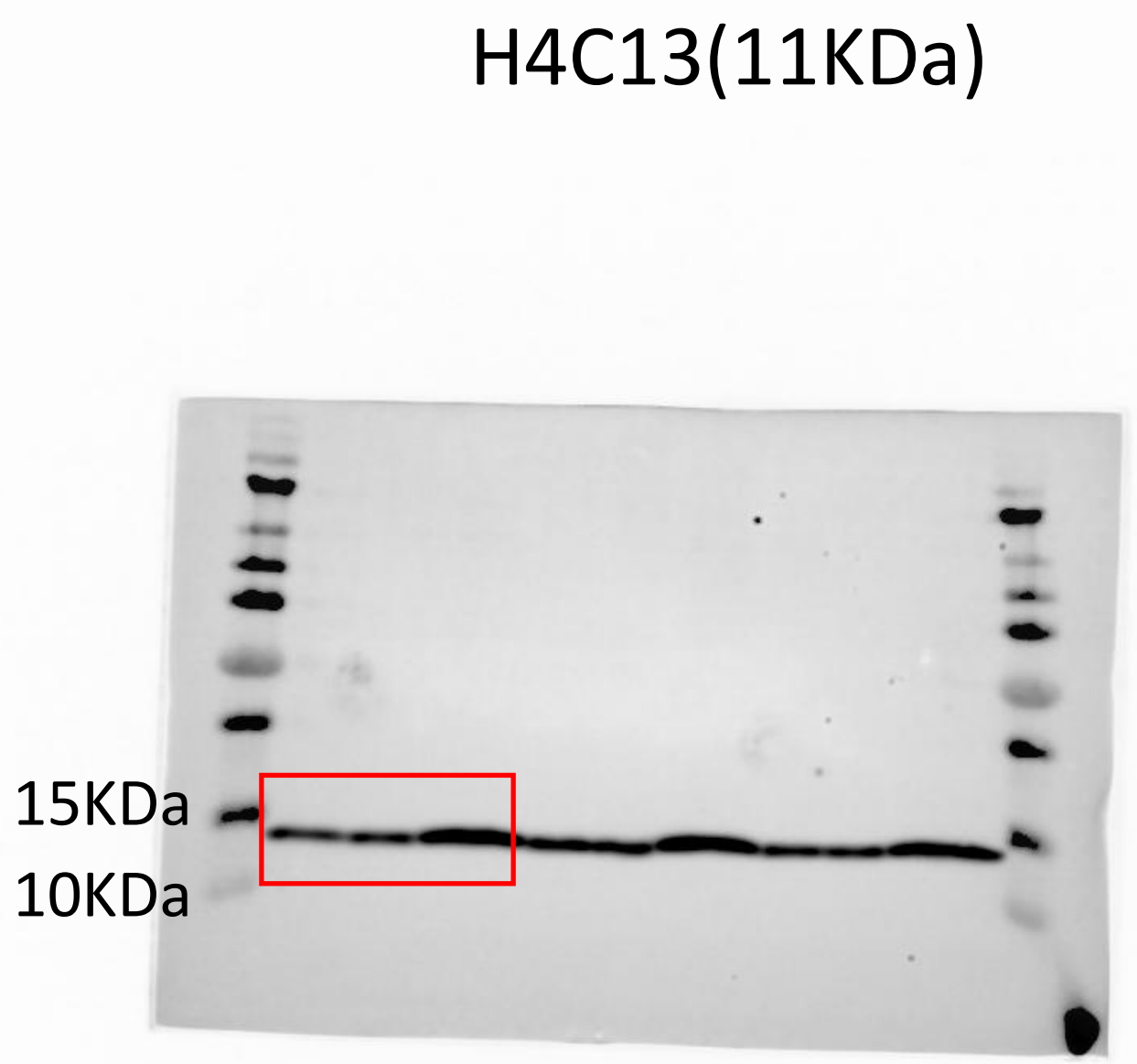

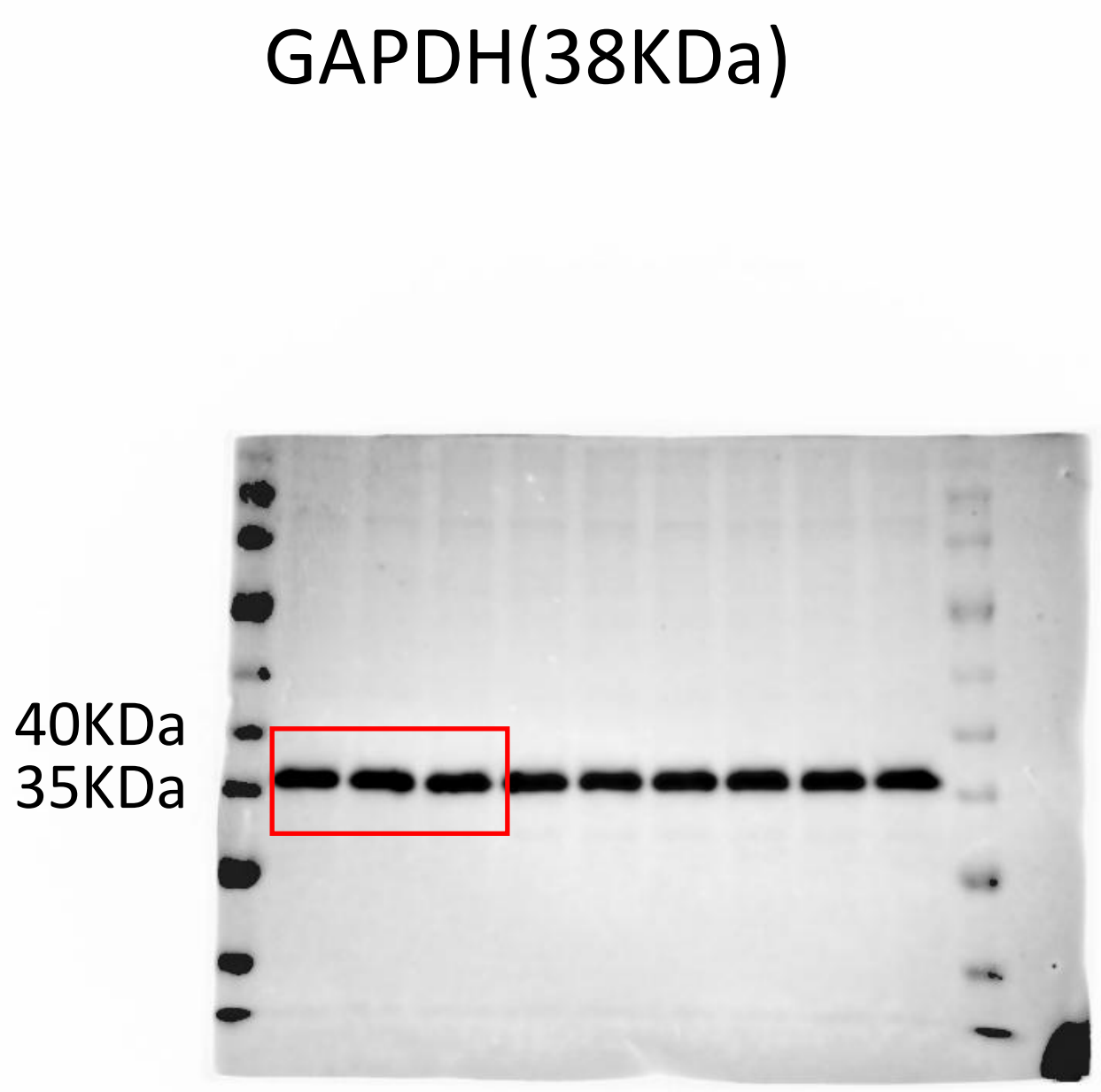

Figure 8B

SiHa-si-H4C13

H4C13(11KDa)

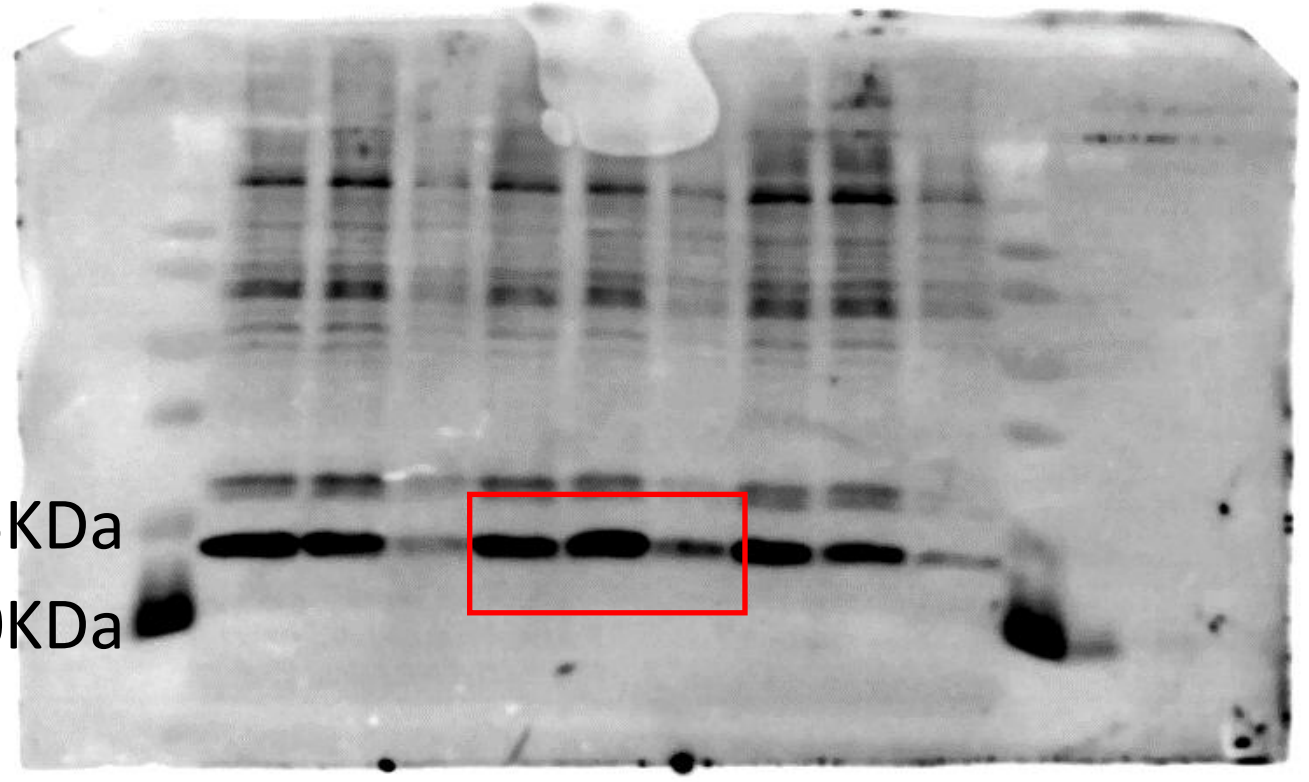

GAPDH(38KDa)

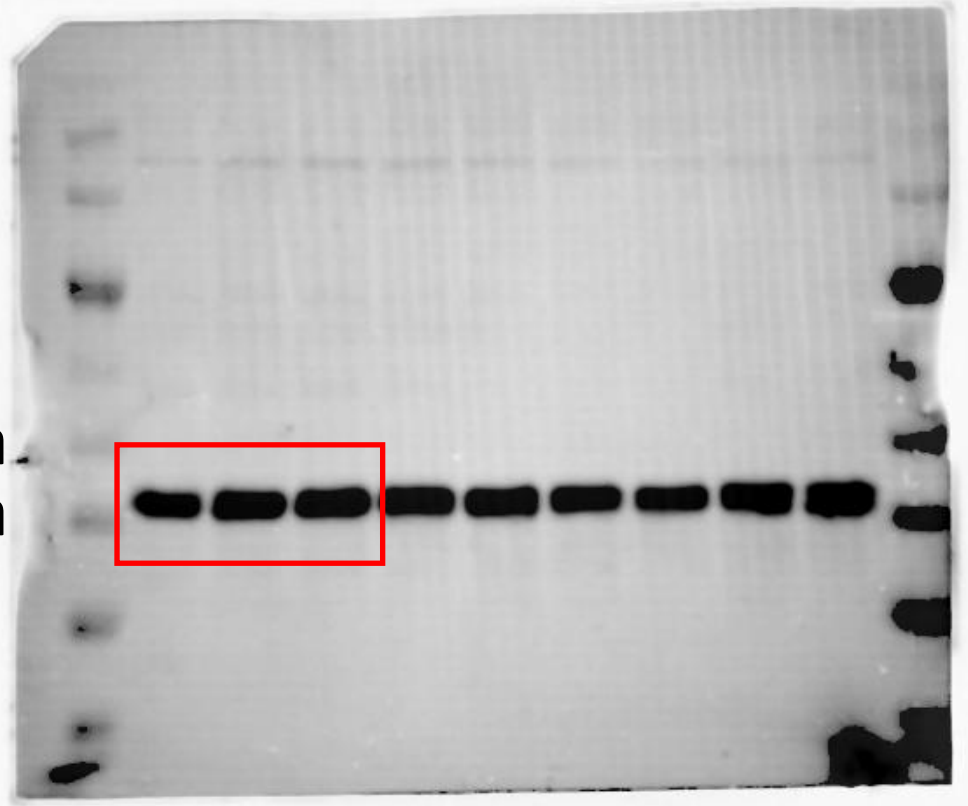

Figure 8B

SiHa-OE-H4C13

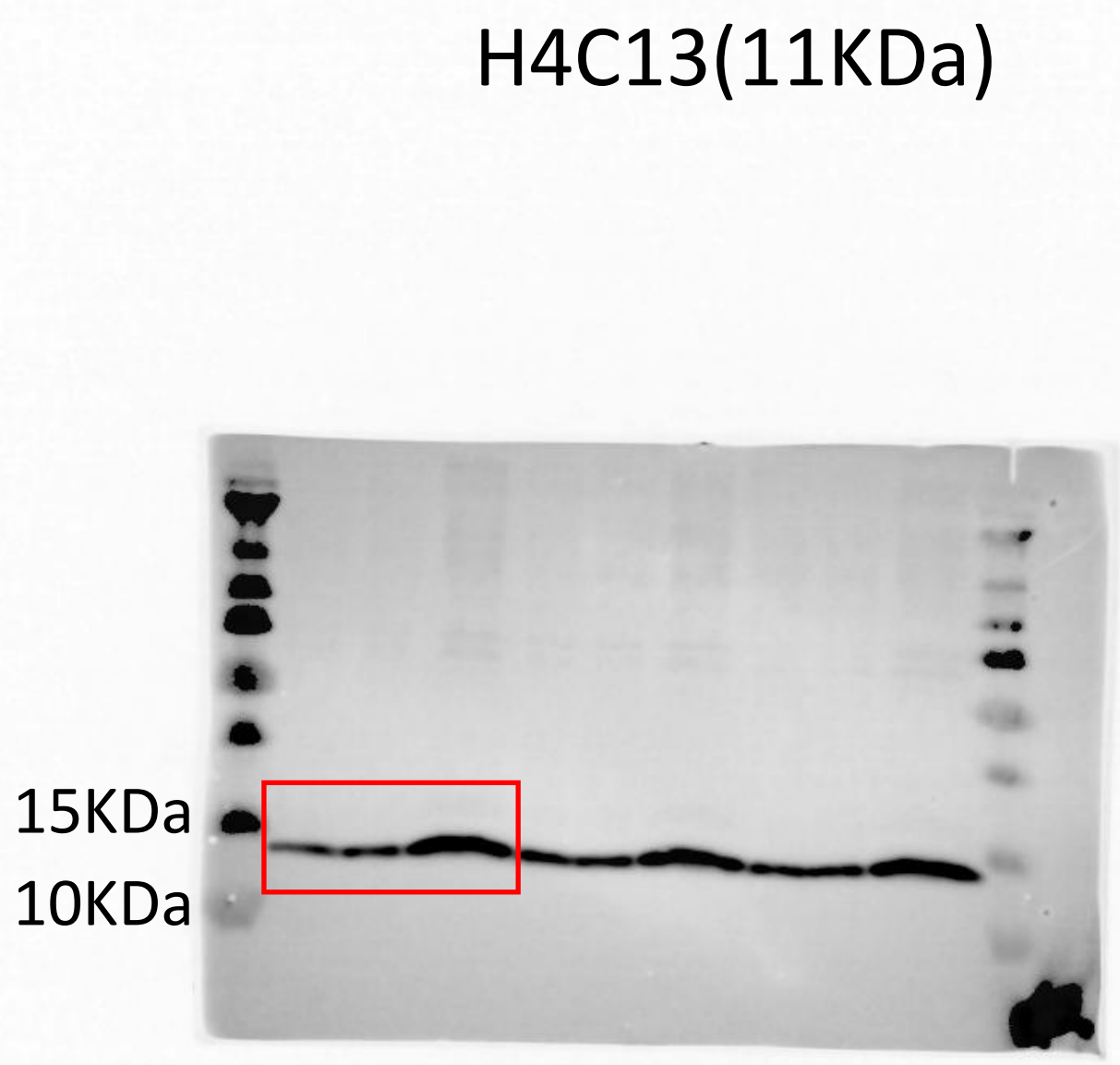

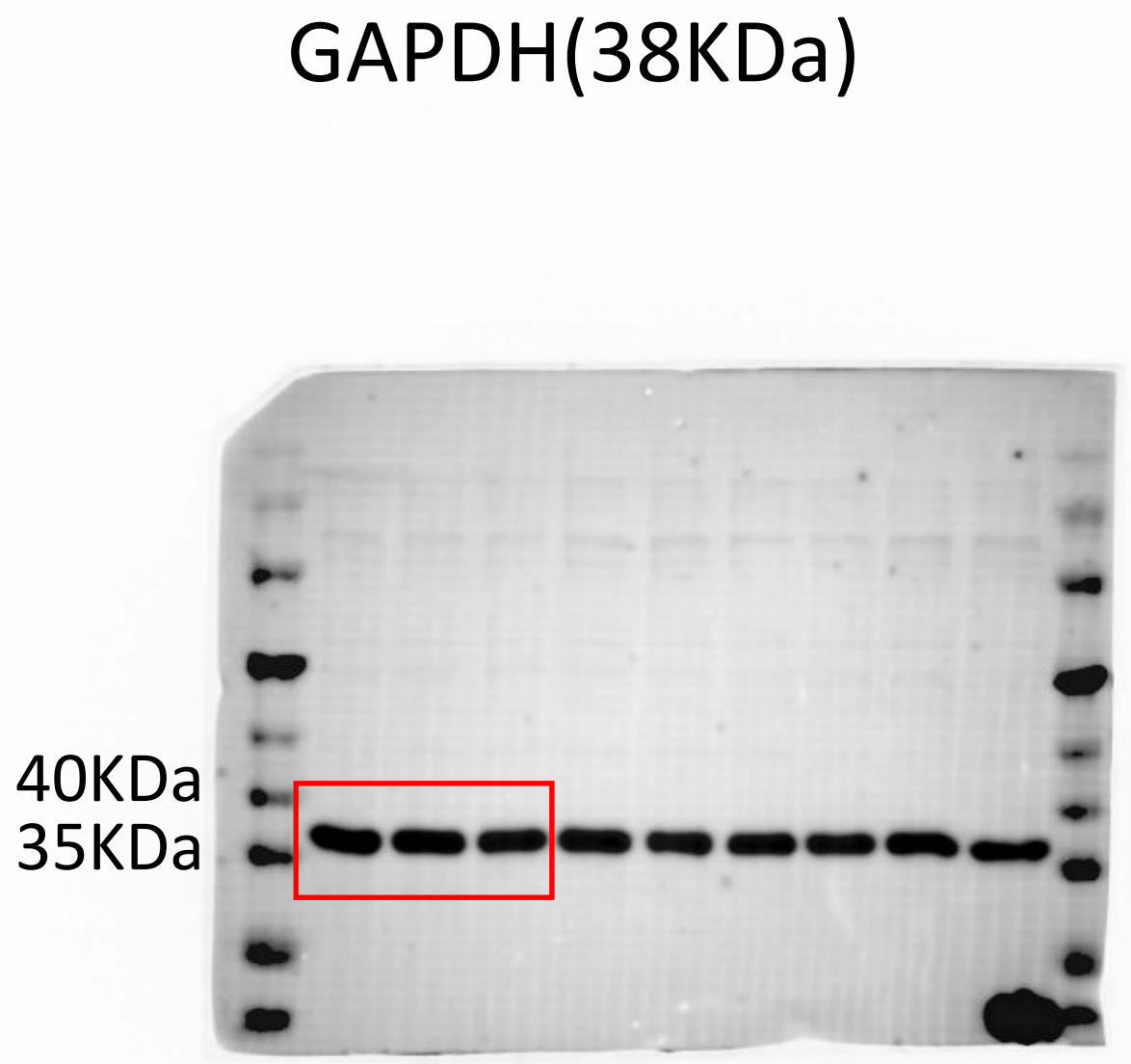

Figure 8E

HeLa-si-H4C13

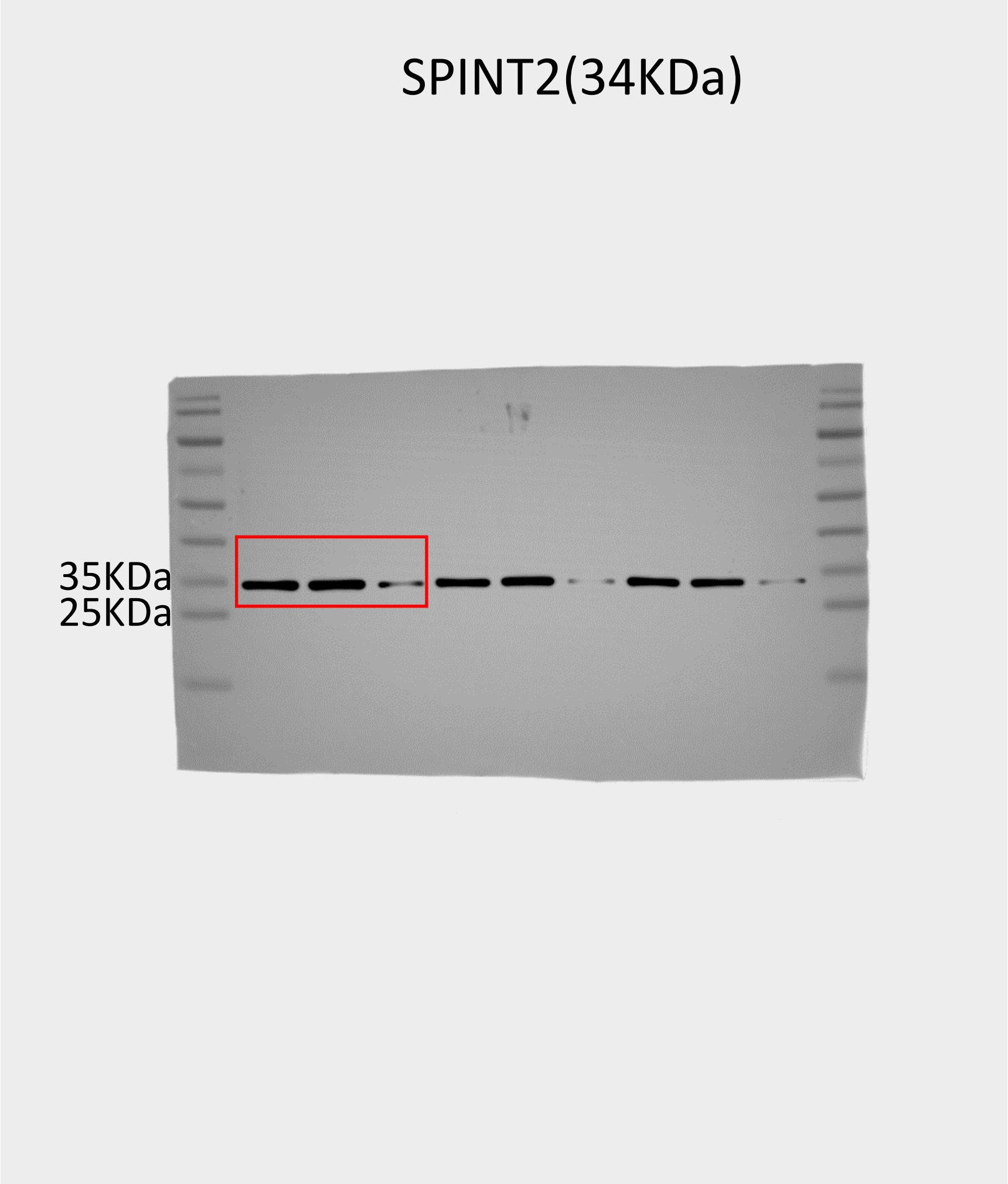

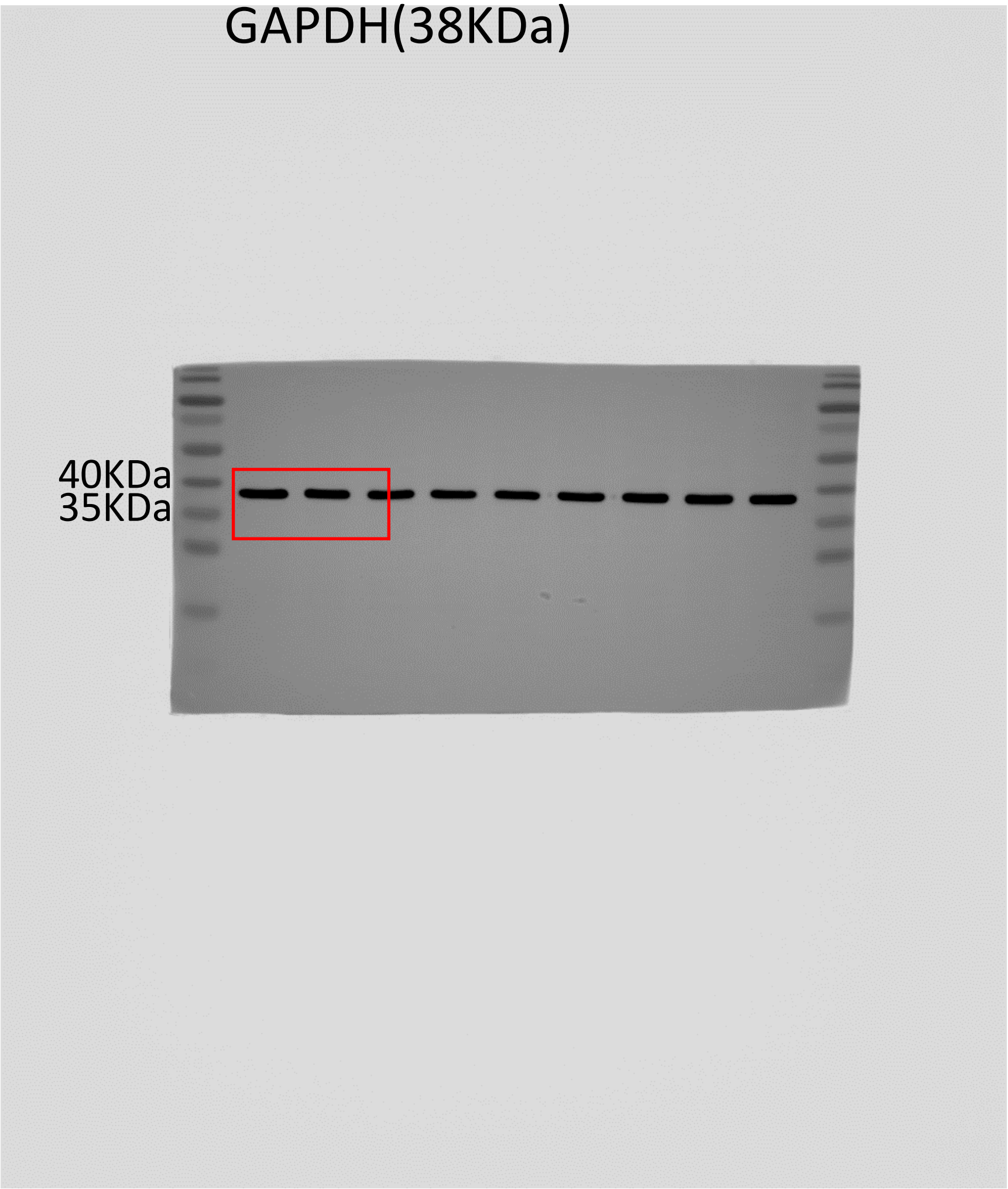

Figure 8E

HeLa-OE-H4C13

SPINT2(34KDa)

35KDa
25KDa

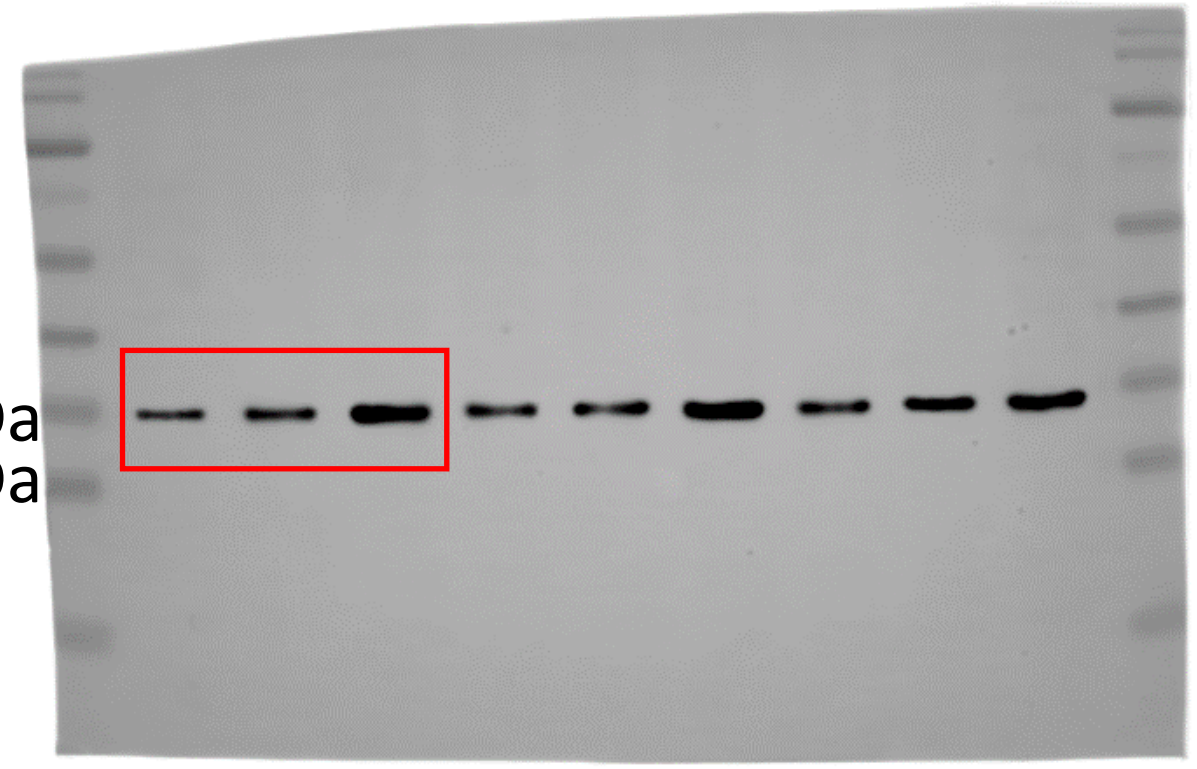

GAPDH(38KDa)

40KDa
35KDa

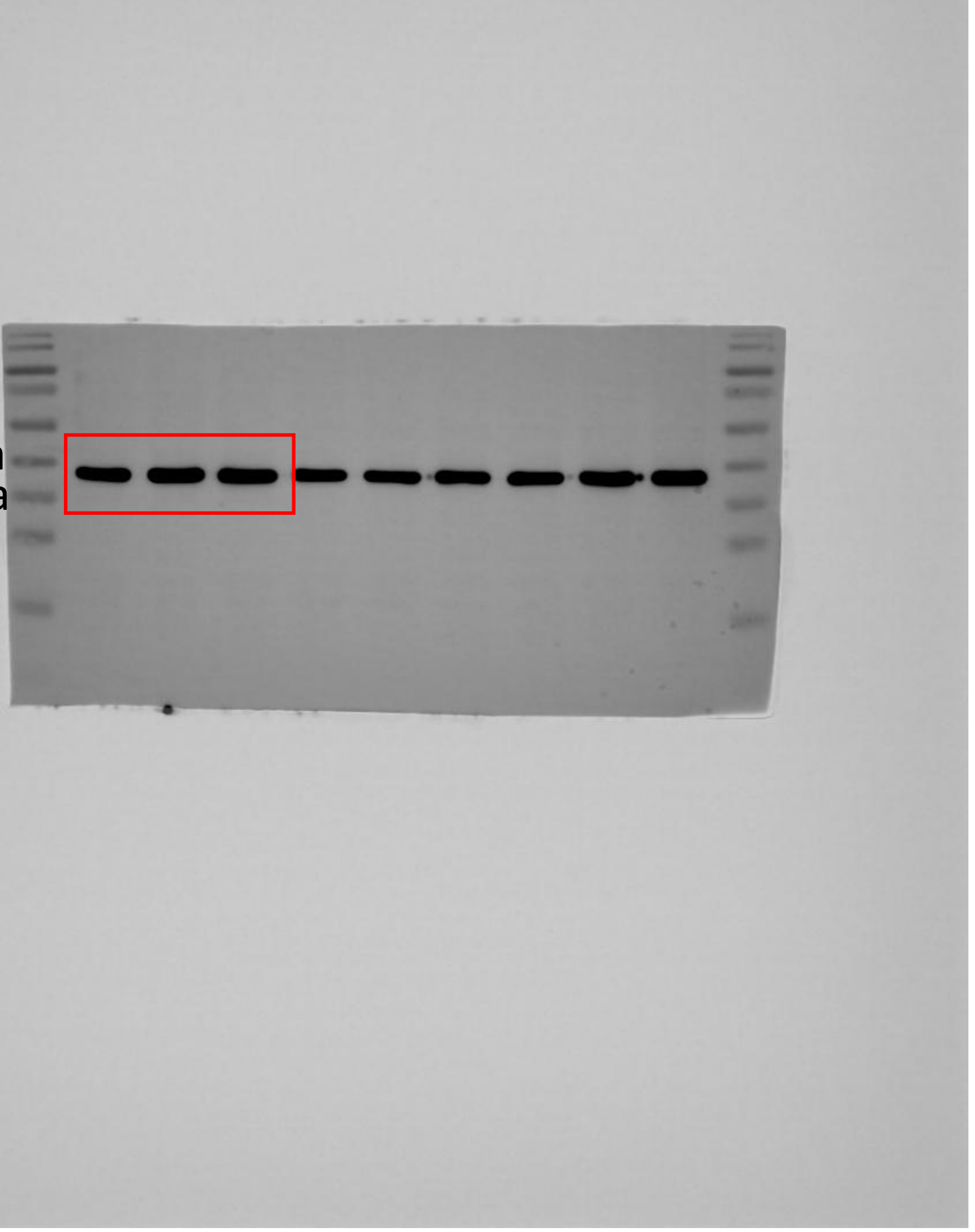

Figure 8E

SiHa-si-H4C13

SPINT2(34KDa)

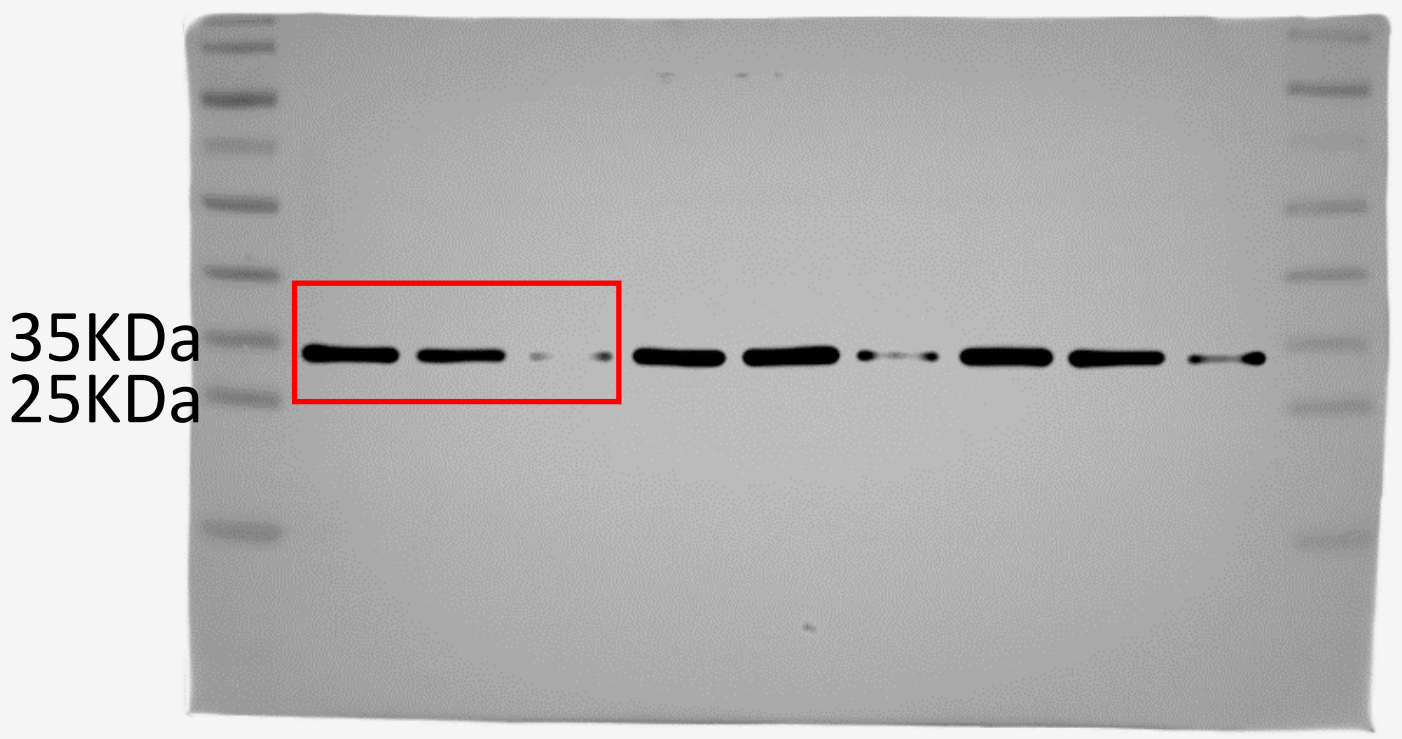

GAPDH(38KDa)

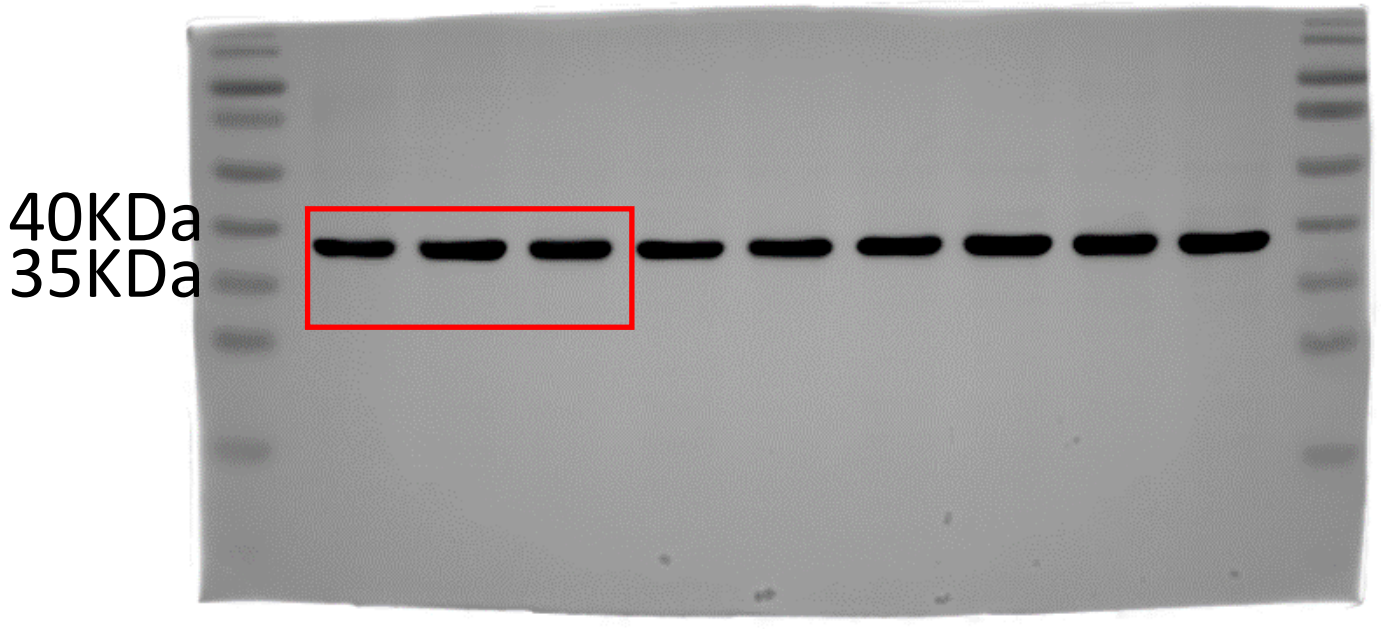

Figure 8E

SiHa-OE-H4C13

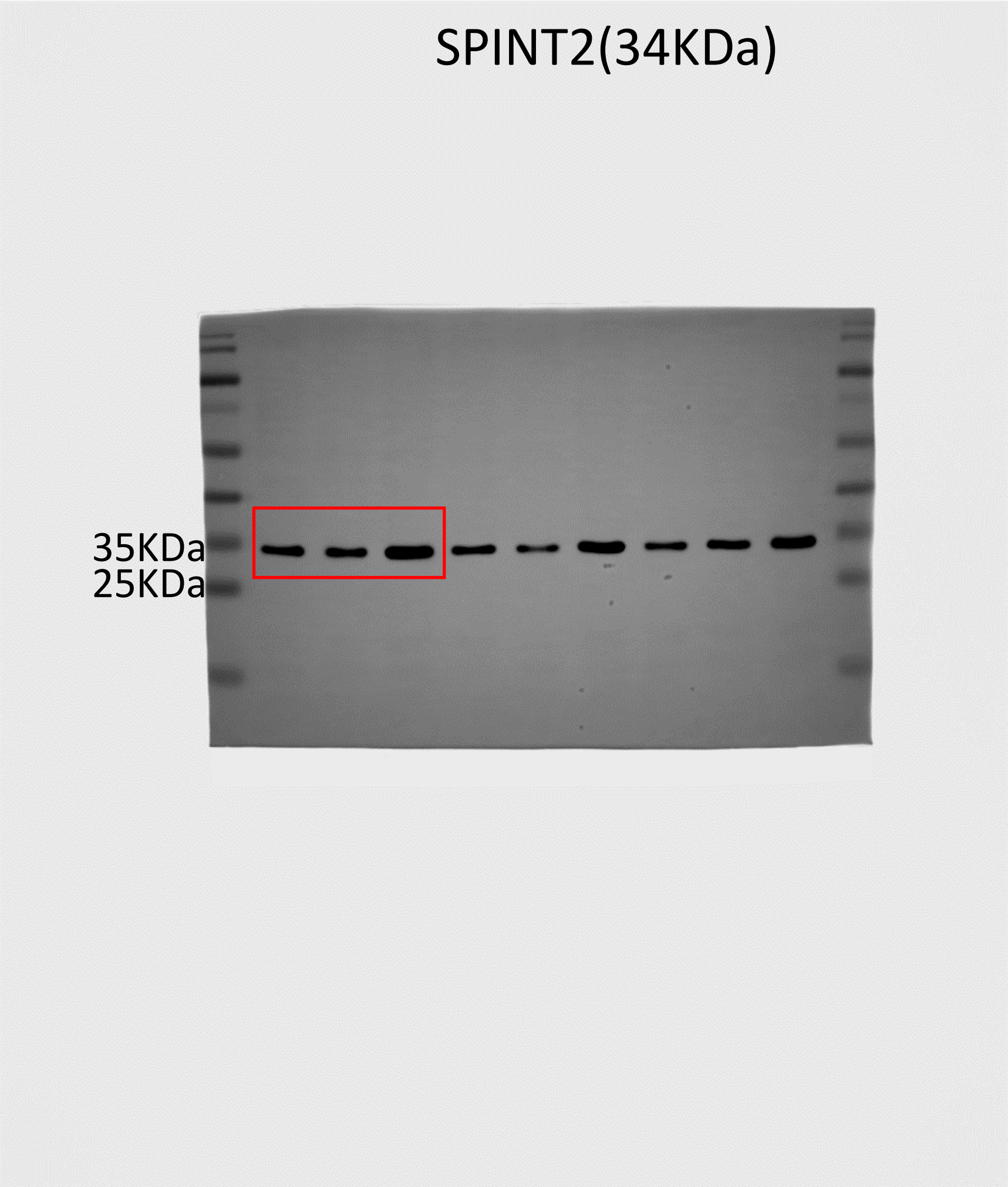

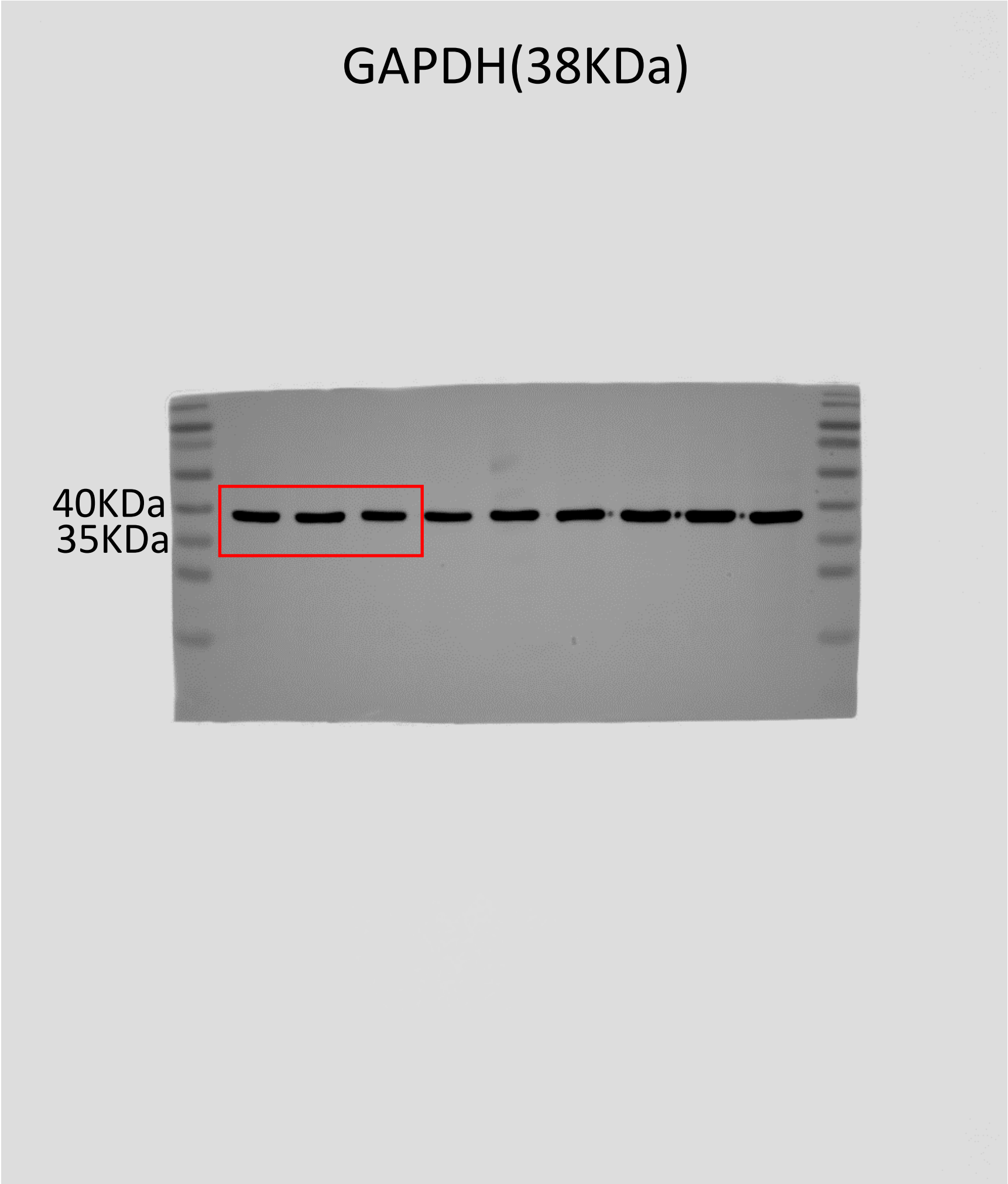

Figure S5 (KO-AKR1C2)

Collagen II (COL2A1) 134 kDa

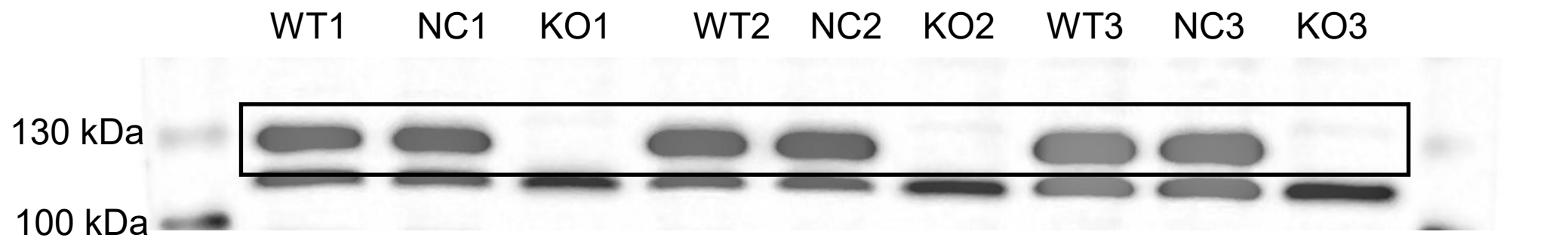

Twist2 60 kDa

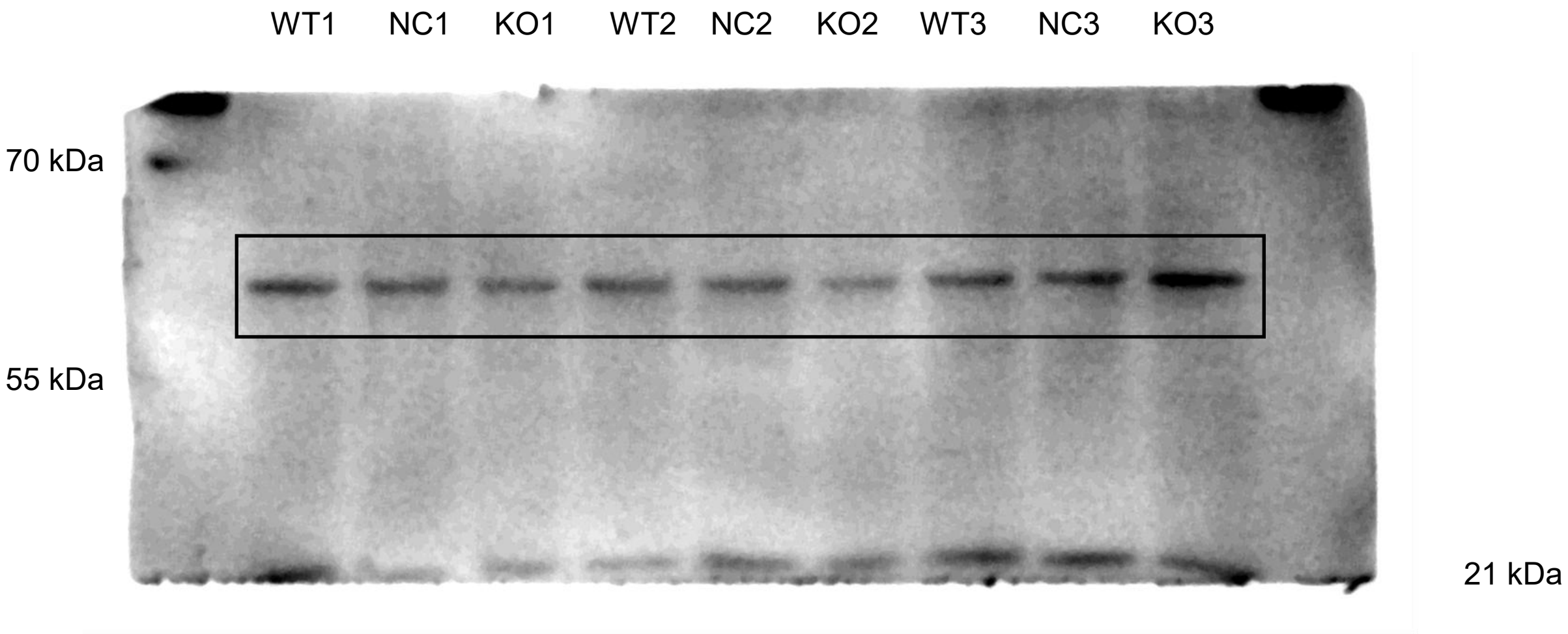

Twist 21 kDa

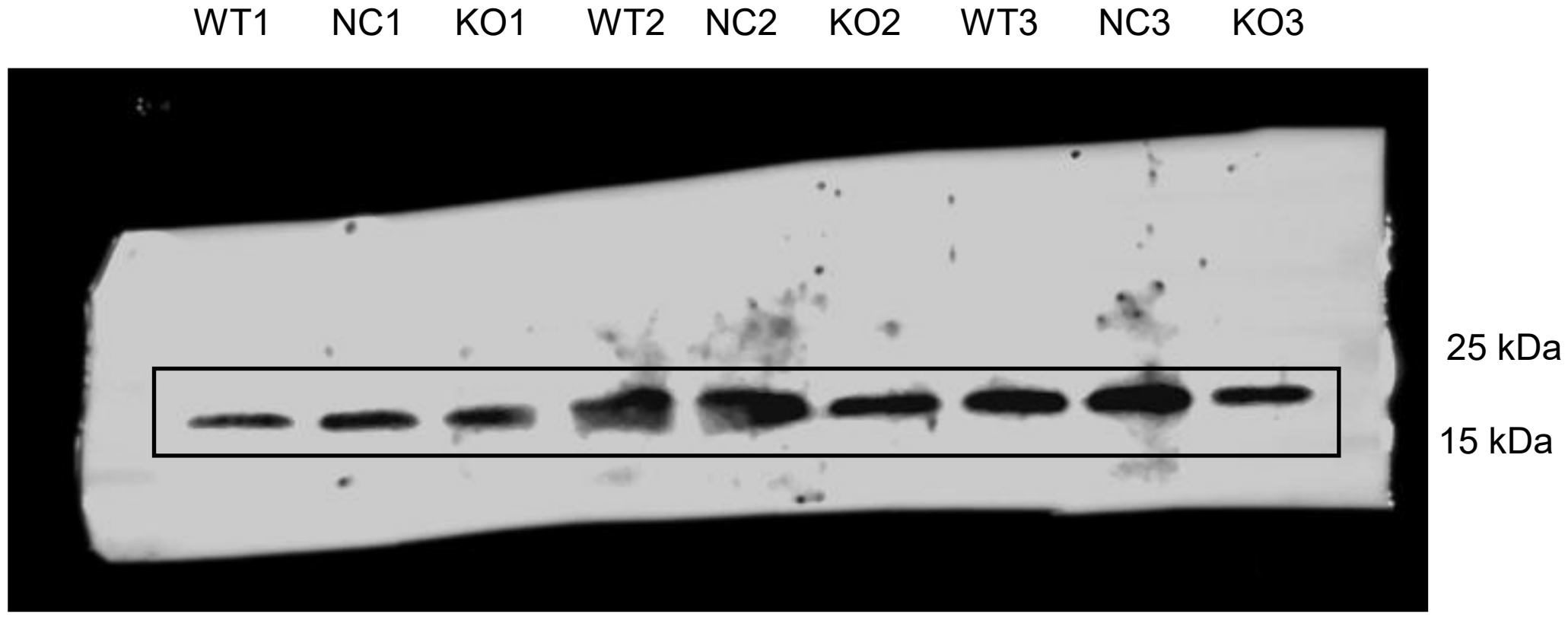

Figure S5 (KO-AKR1C2)

SNAIL + SLUG 29 kDa

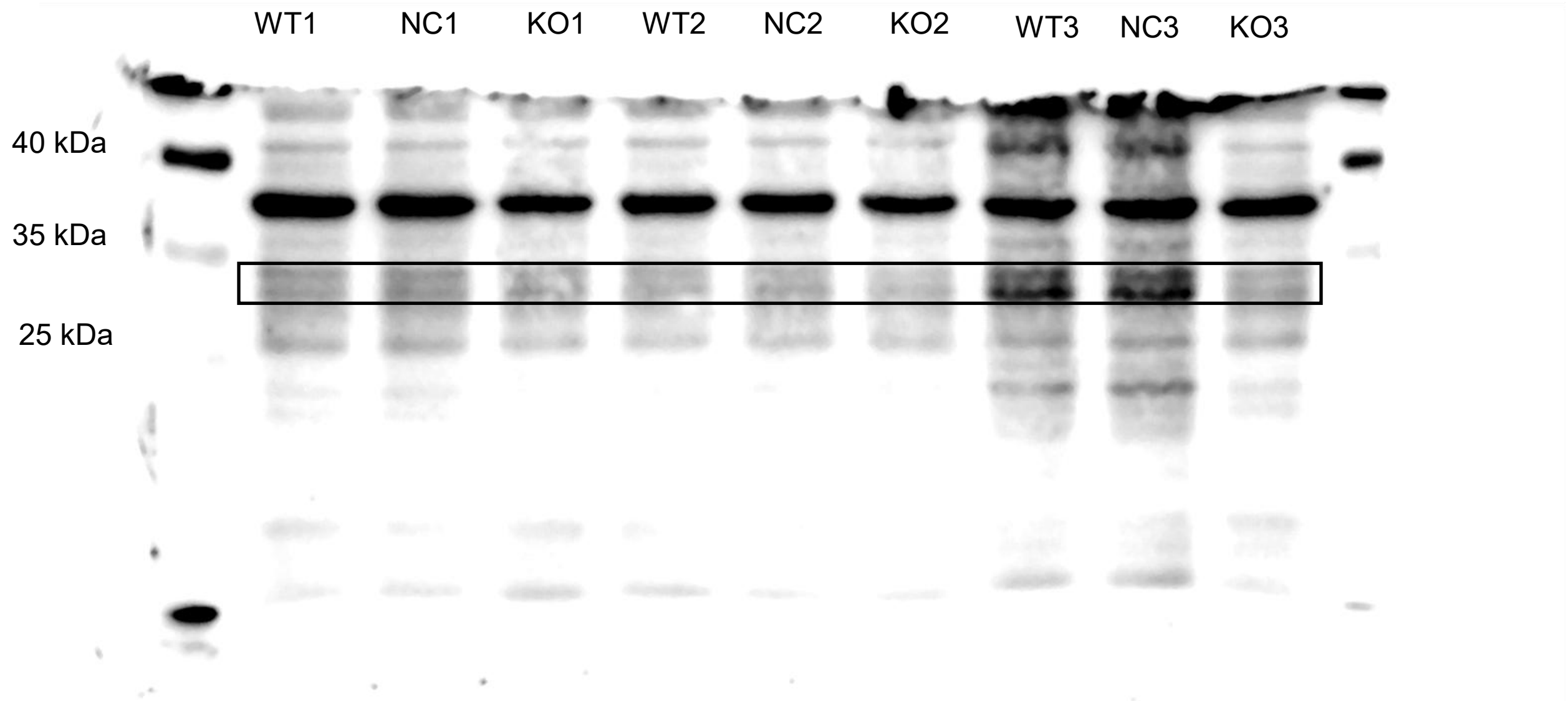

Figure S5 (KO-AKR1C2)

E-cadherin 125 kDa

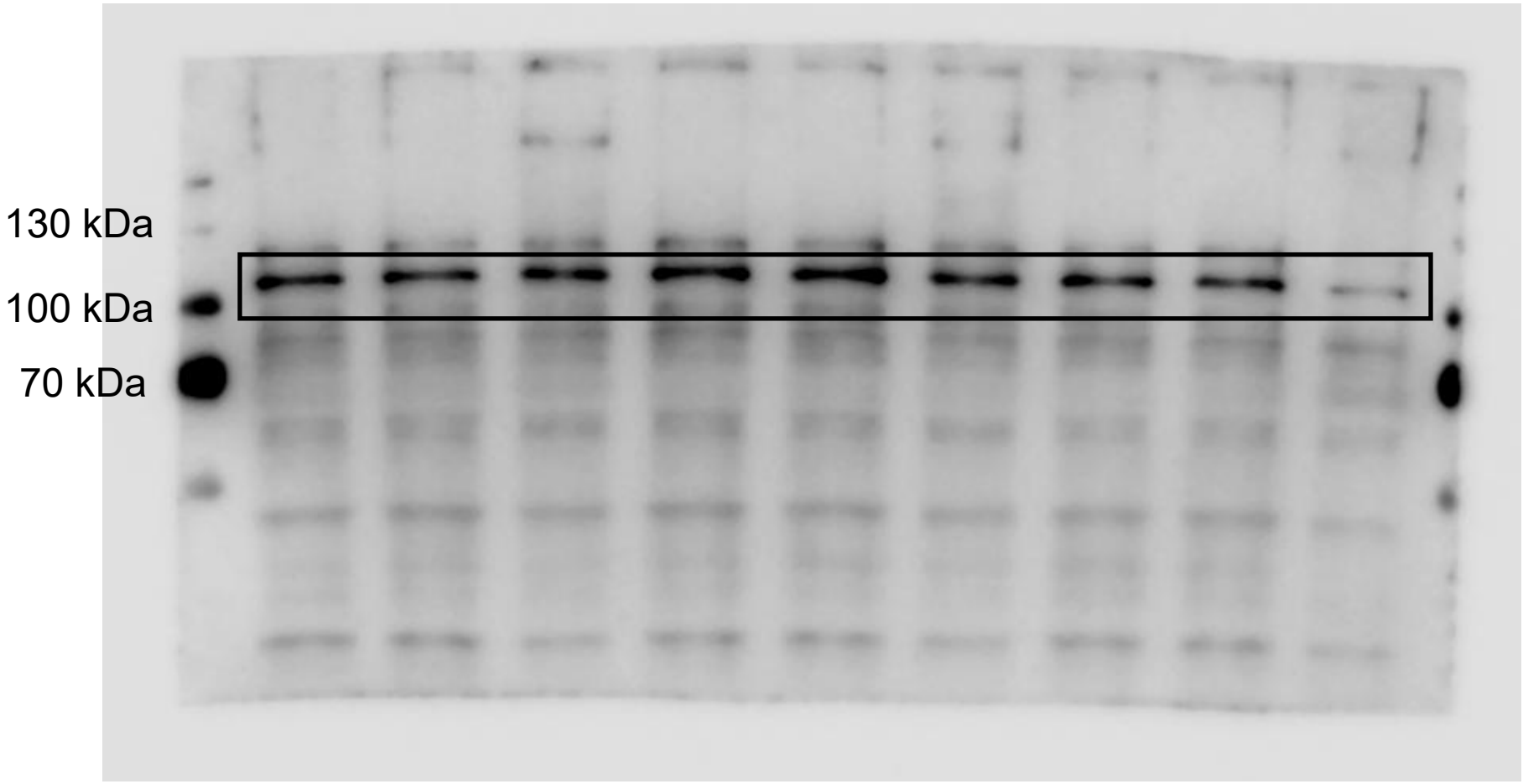

integrin-β1 135 kDa

WT1 NC1 KO1 WT2 NC2 KO2 WT3 NC3 KO3

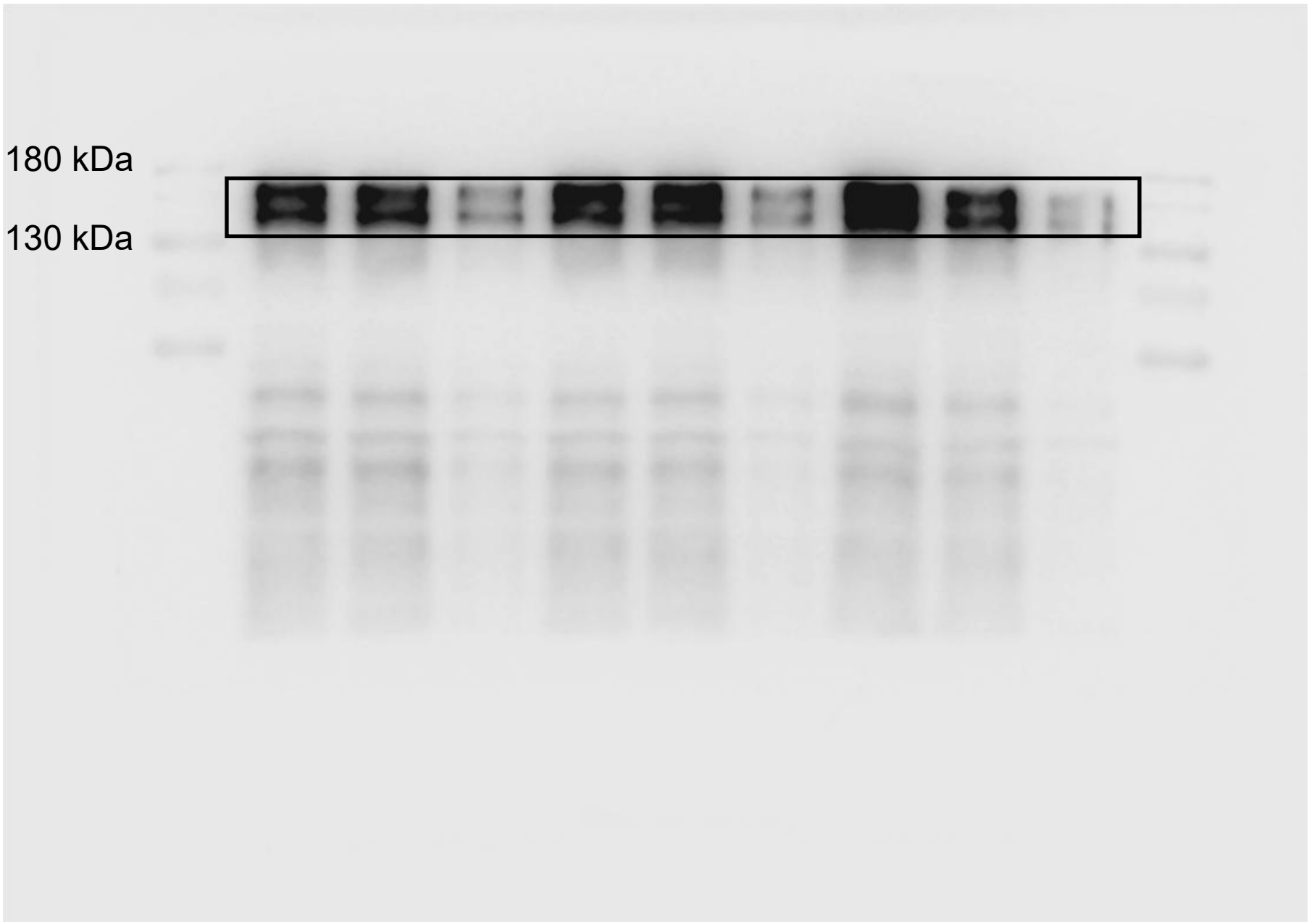

Figure S5 (KO-AKR1C2)

vinculin 124 kDa

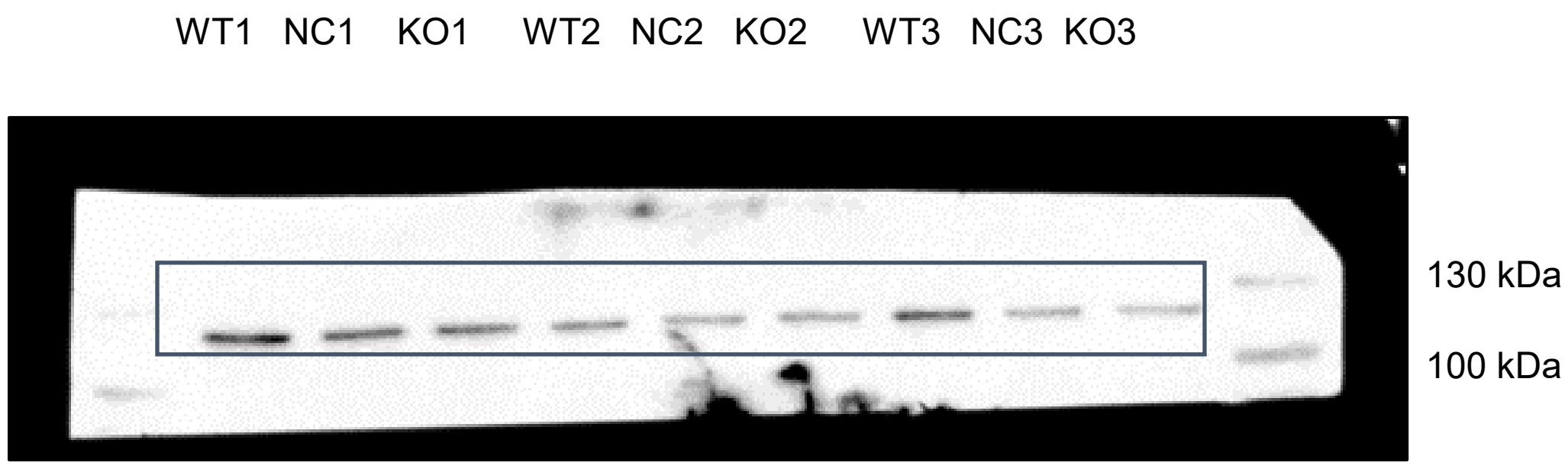

integrin α1 200 KD

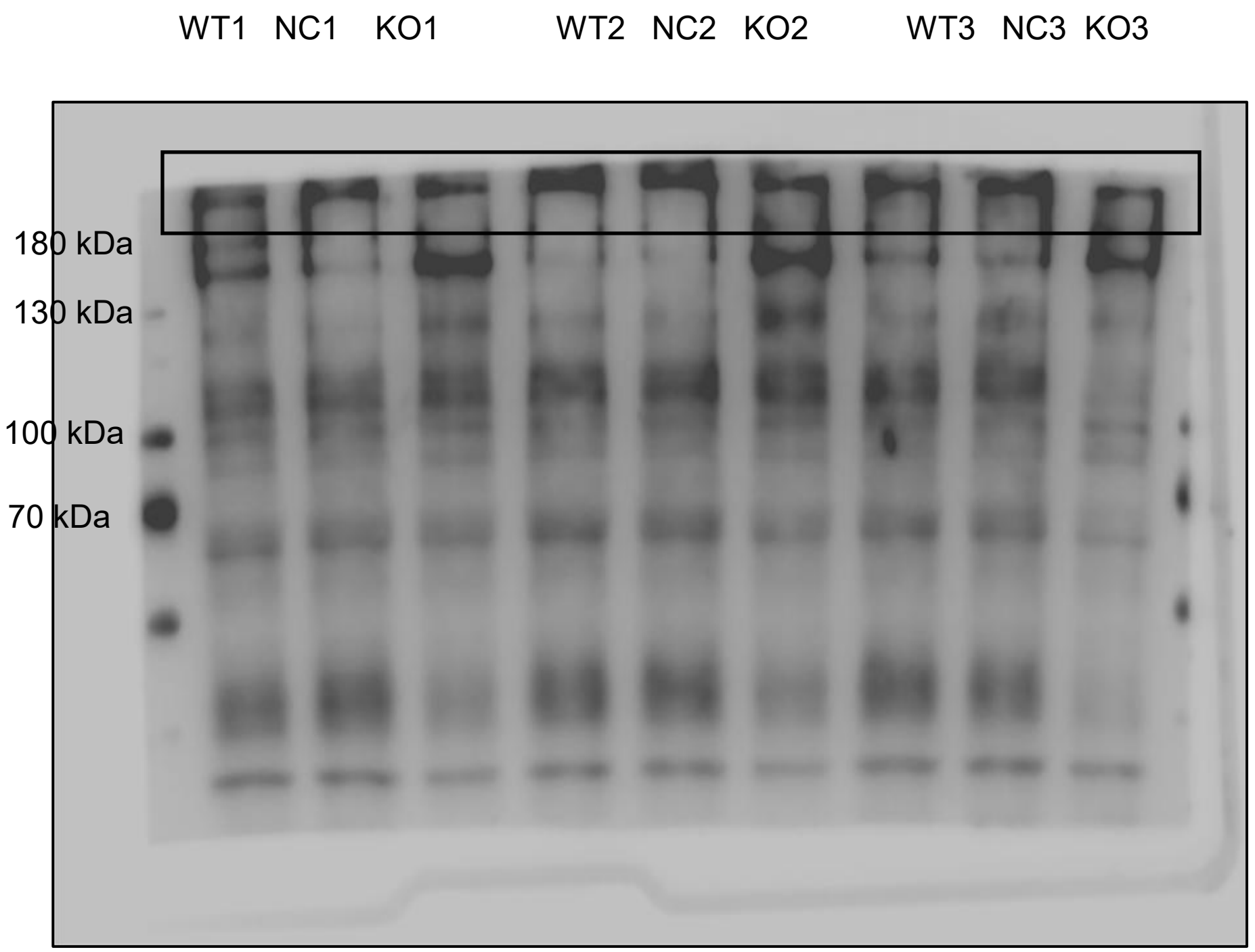

Figure S5 (KO-AKR1C2)

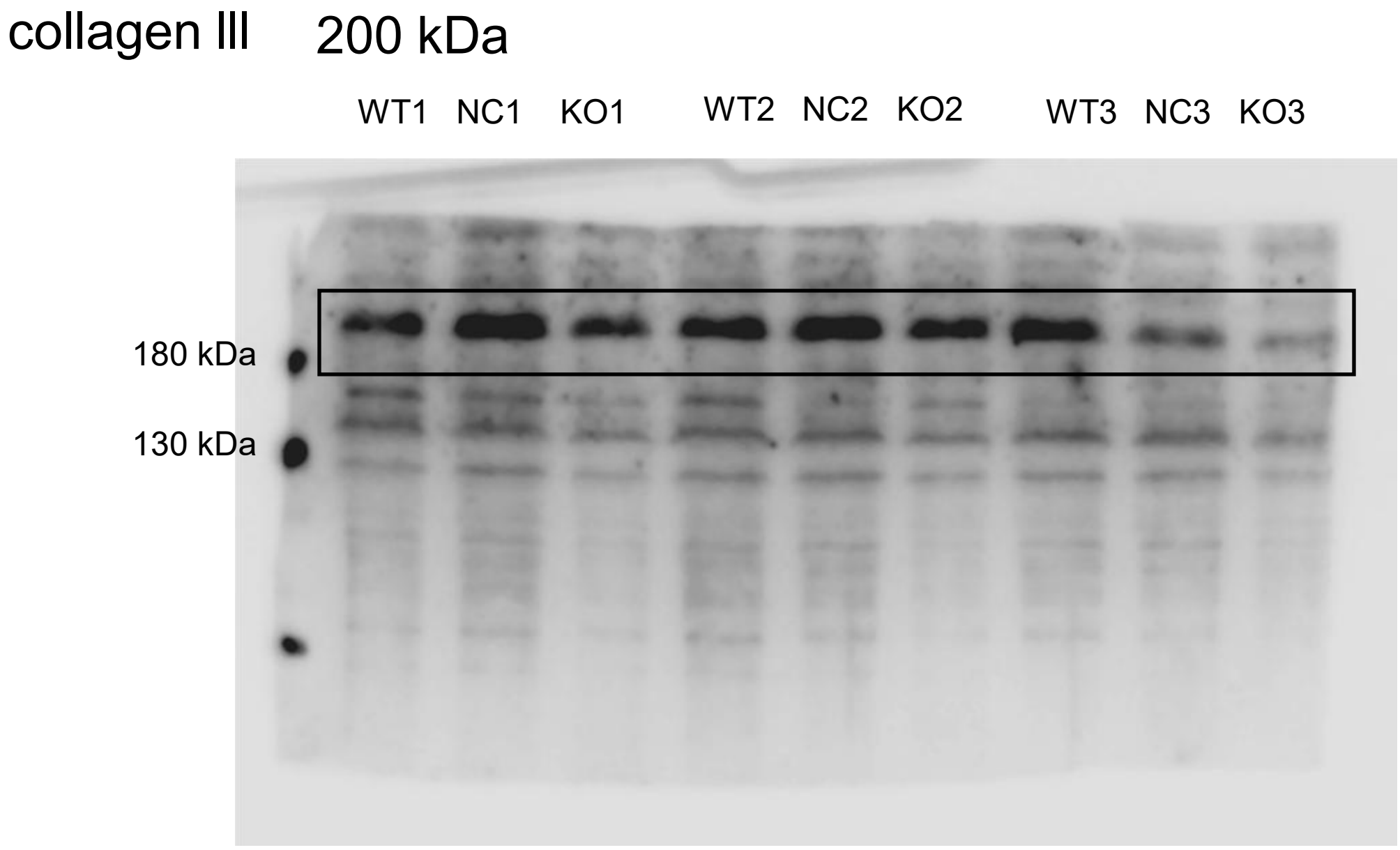

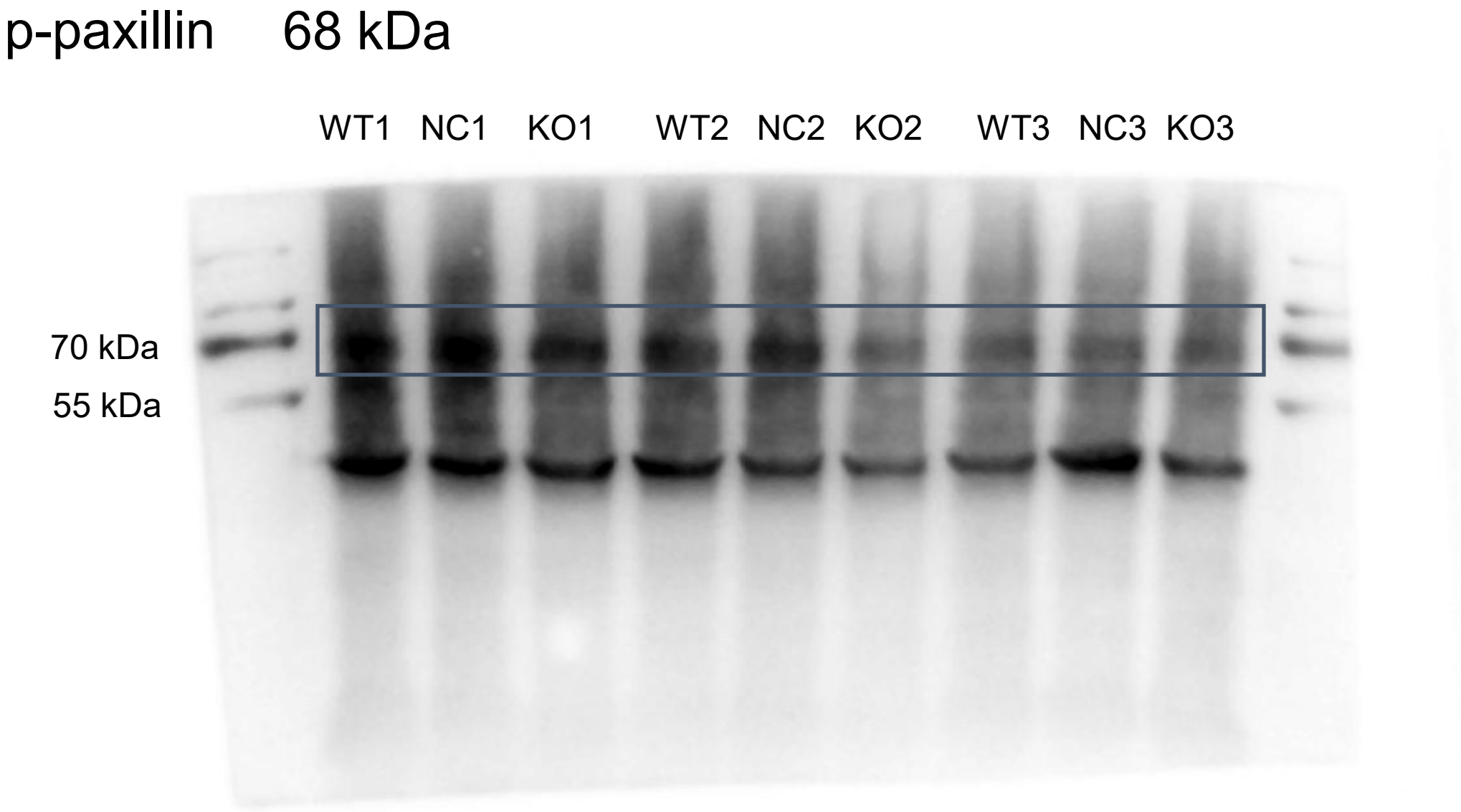

Figure S5 (KO-AKR1C2)

ILK 59 kDa

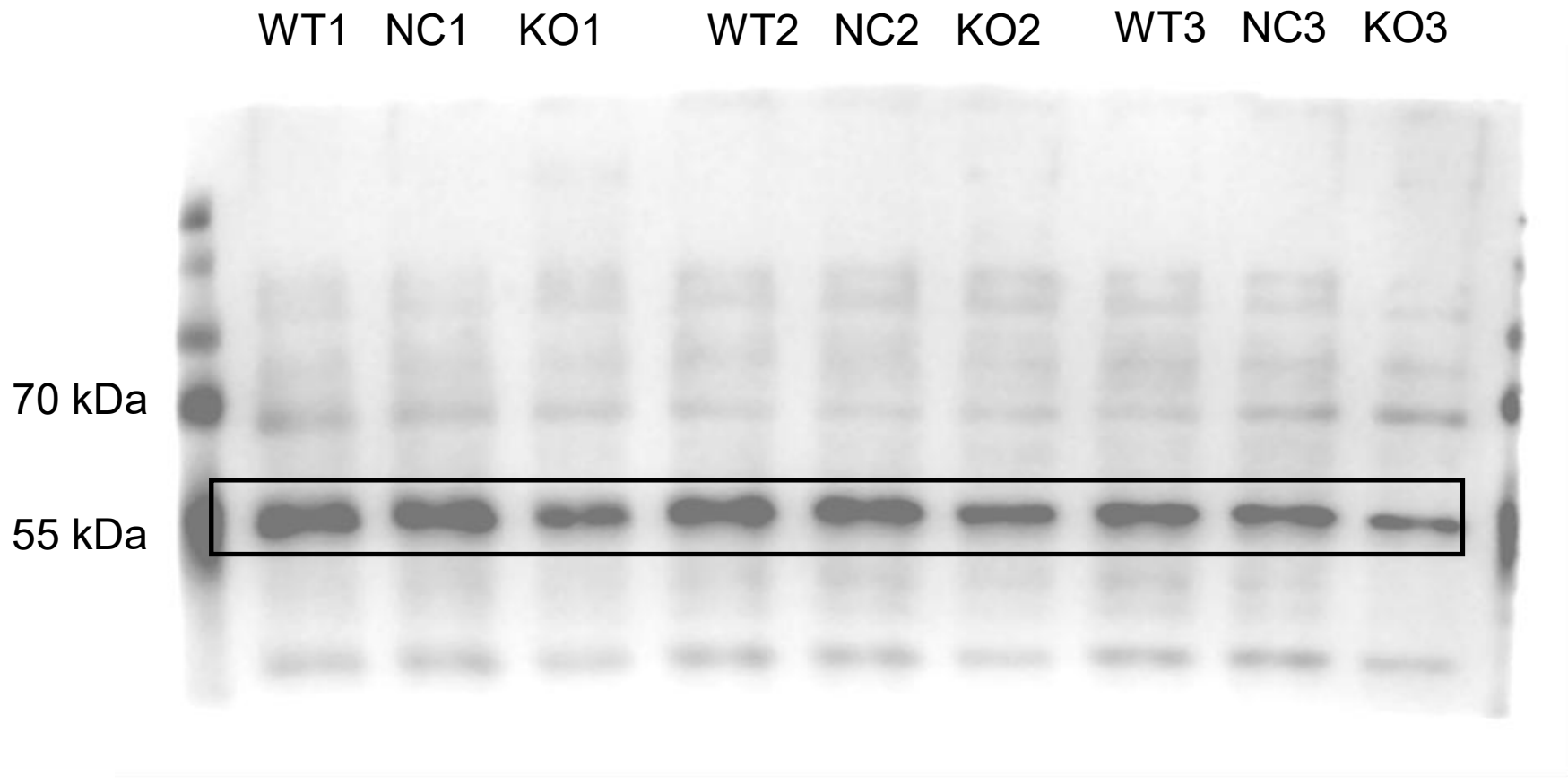

Tensin 2 145 kDa

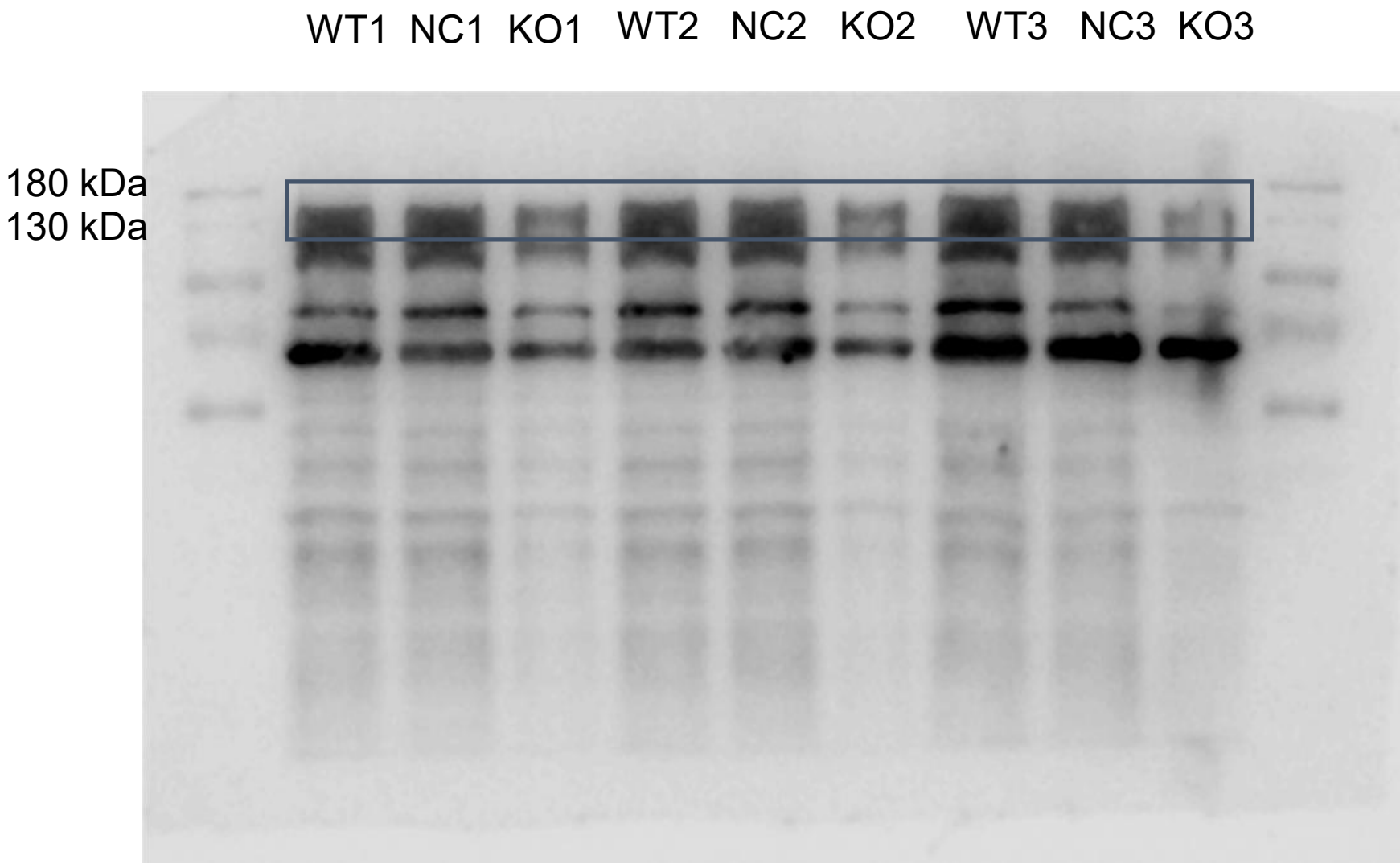

Figure S5 (KO-AKR1C2)

talin-1 270 kDa

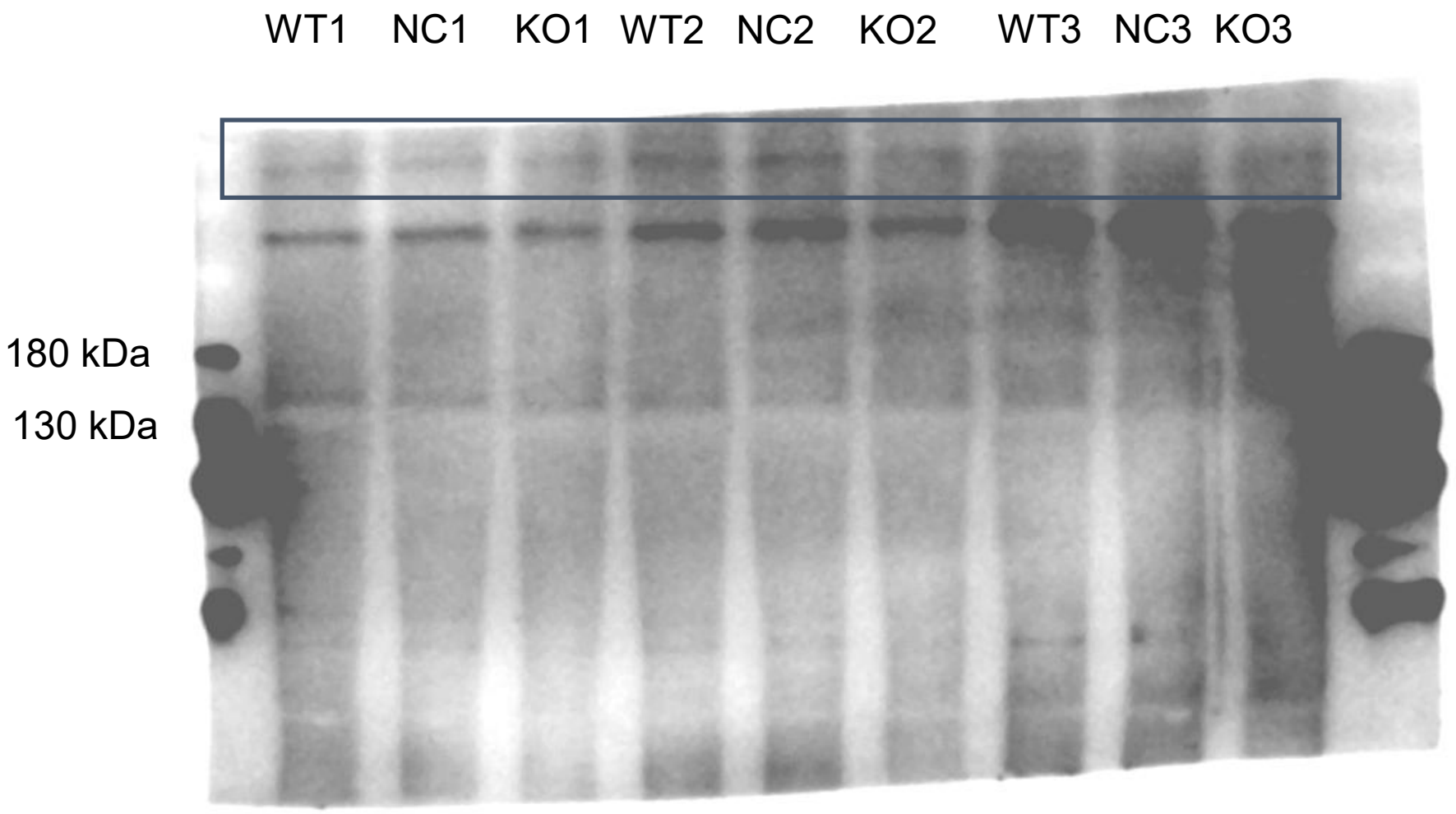

ZEB1 220 kDa

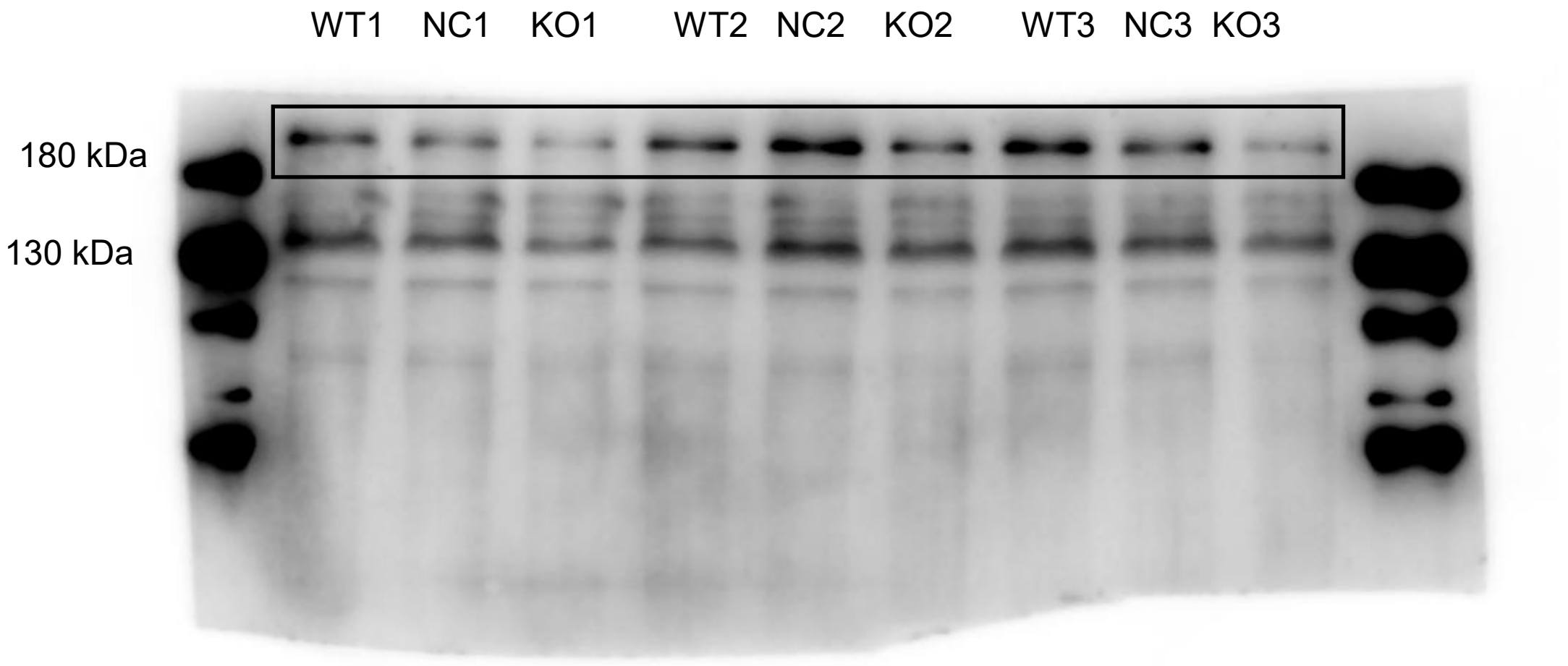

Figure S5 (KO-AKR1C2)

P-FAK 125 kDa

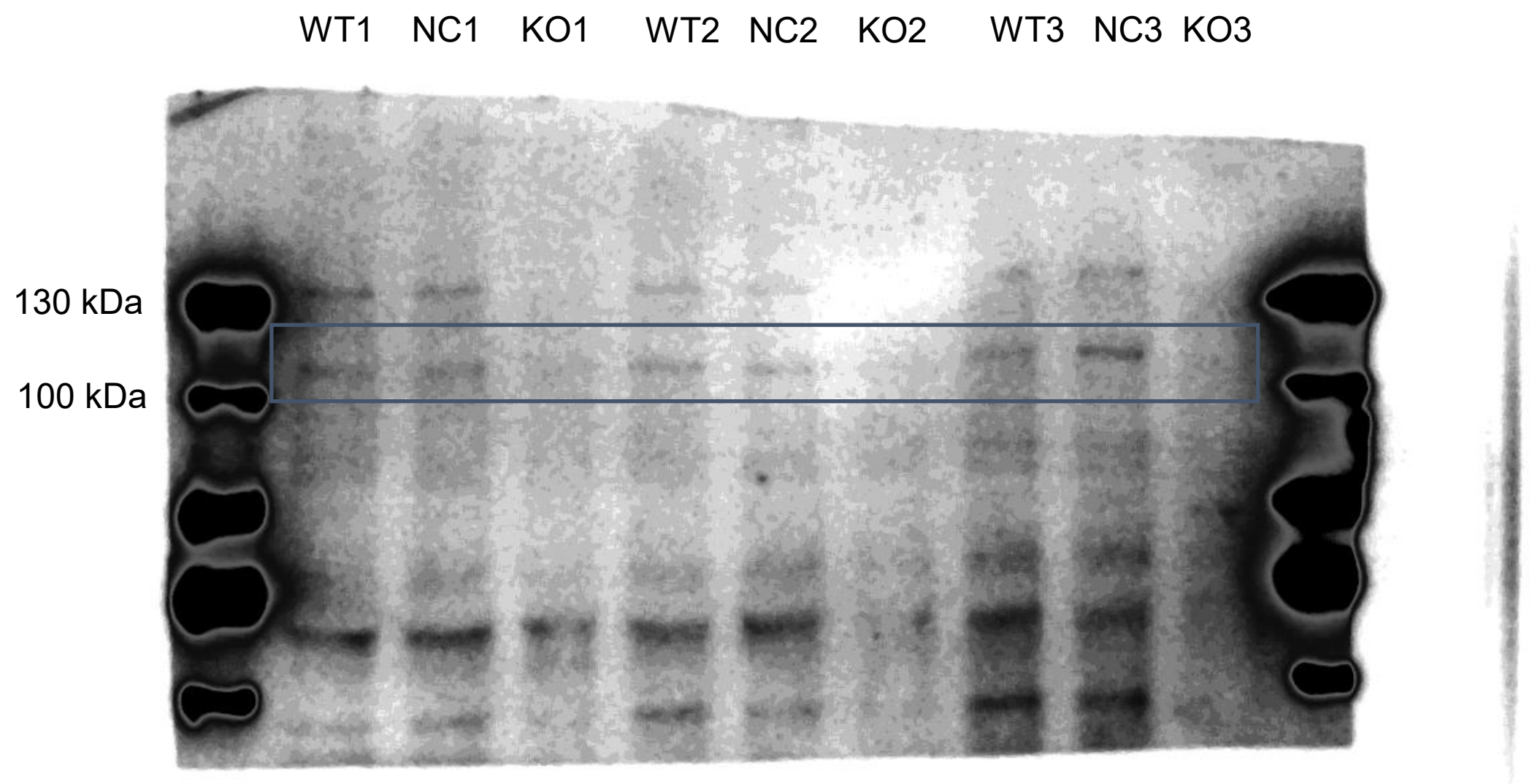

Figure S5 (KO-AKR1C2)

Vimentin 54KDa

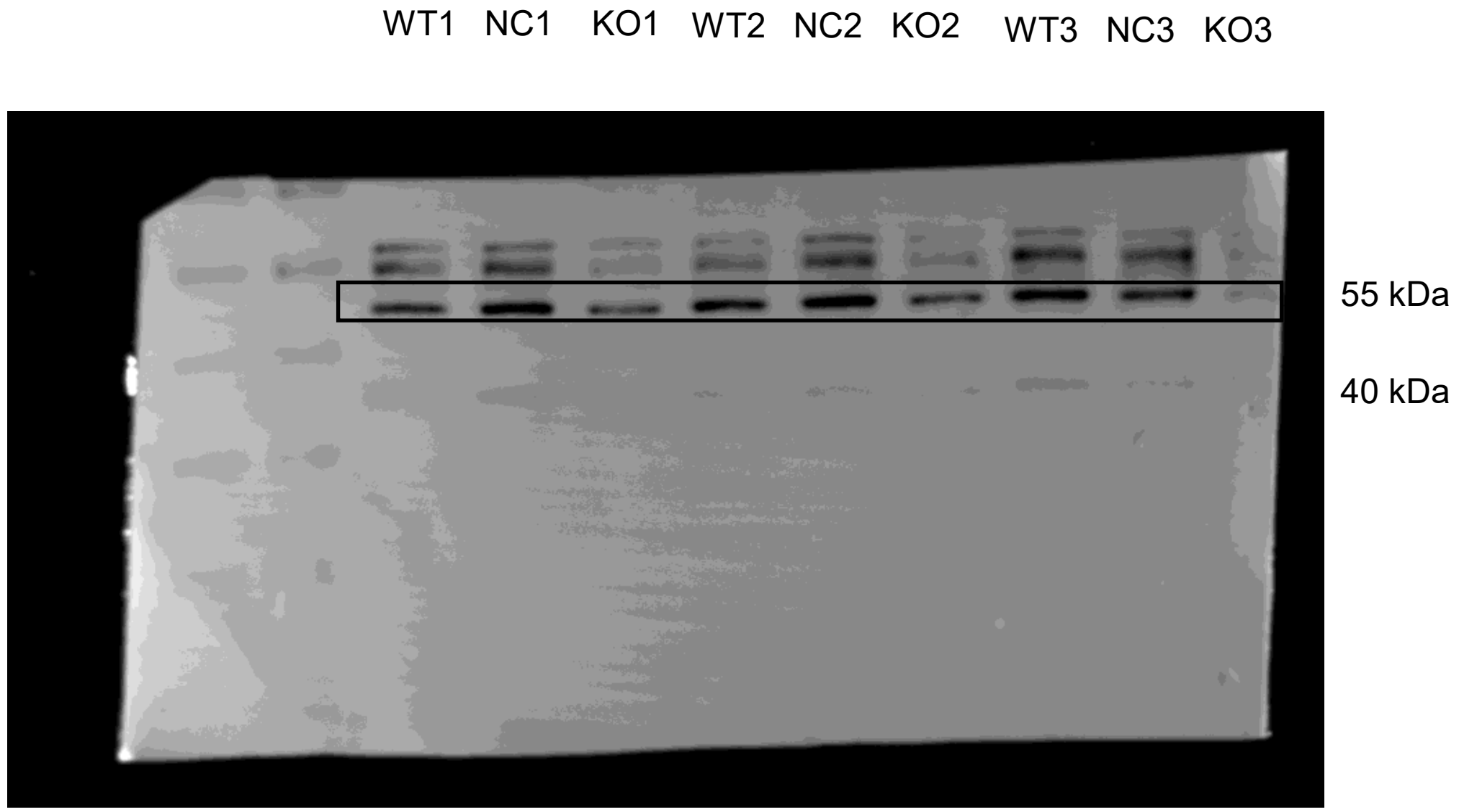

Paxilin 68KDa

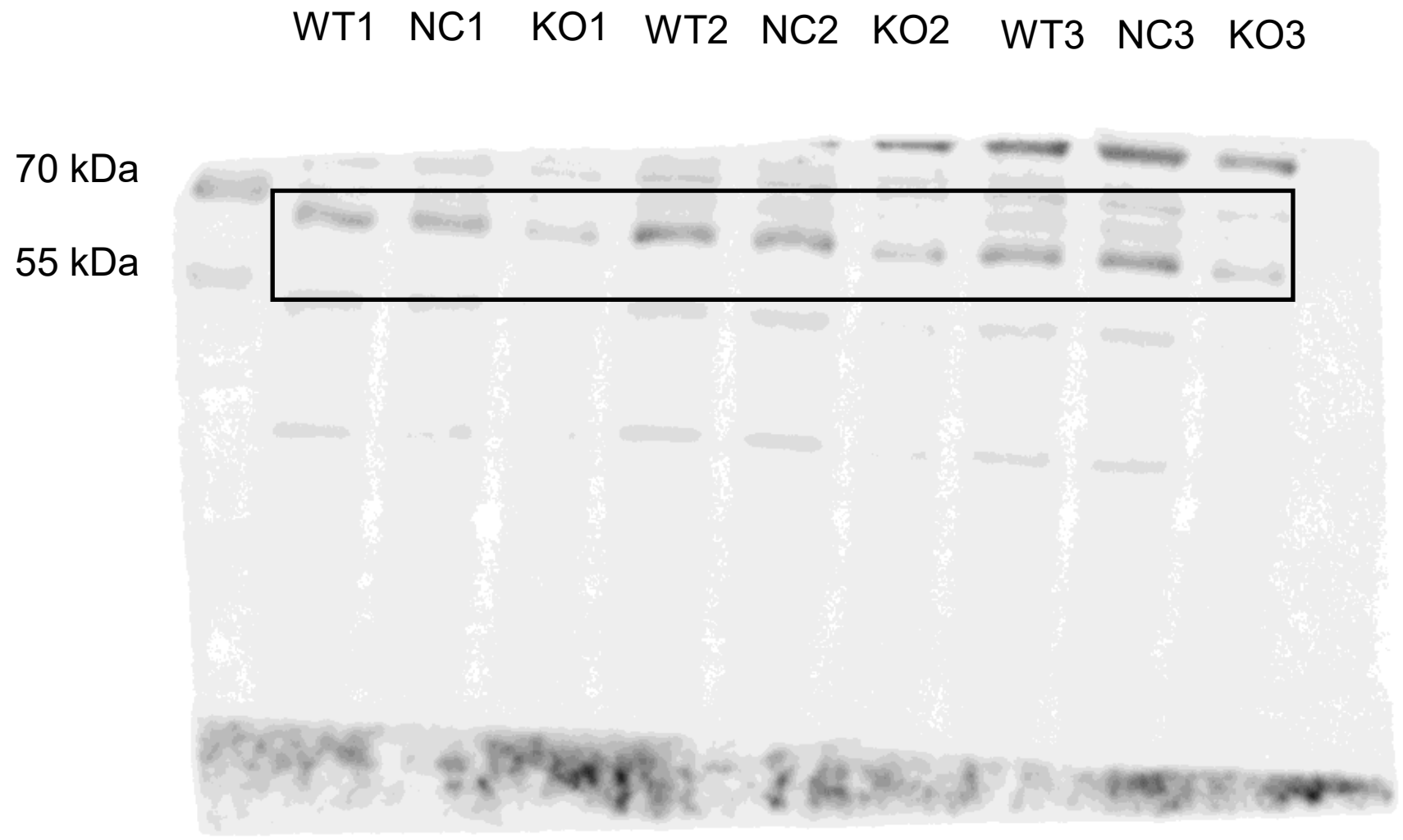

Figure S5 (KO-AKR1C2)

COL4A1

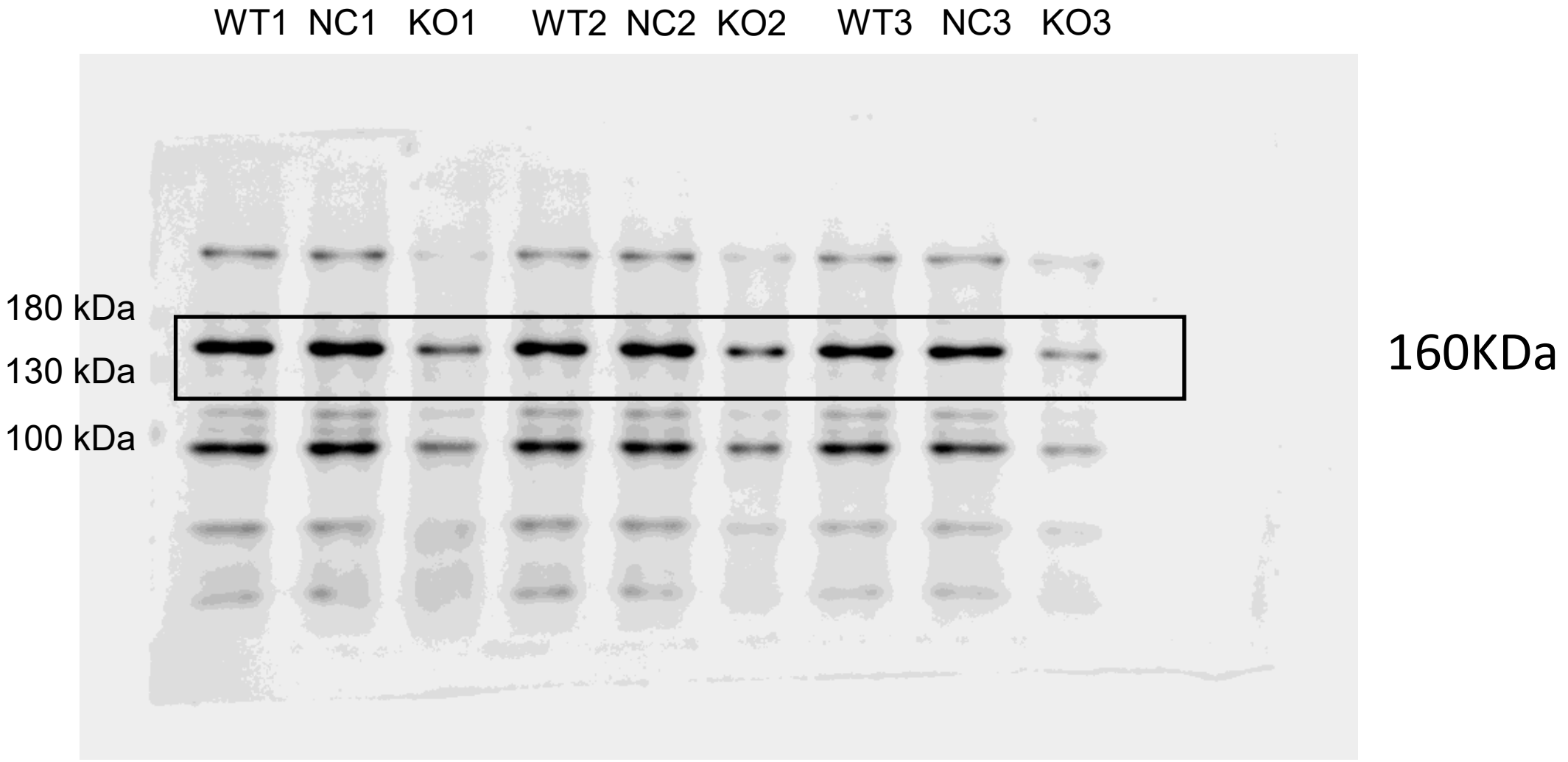

FAK

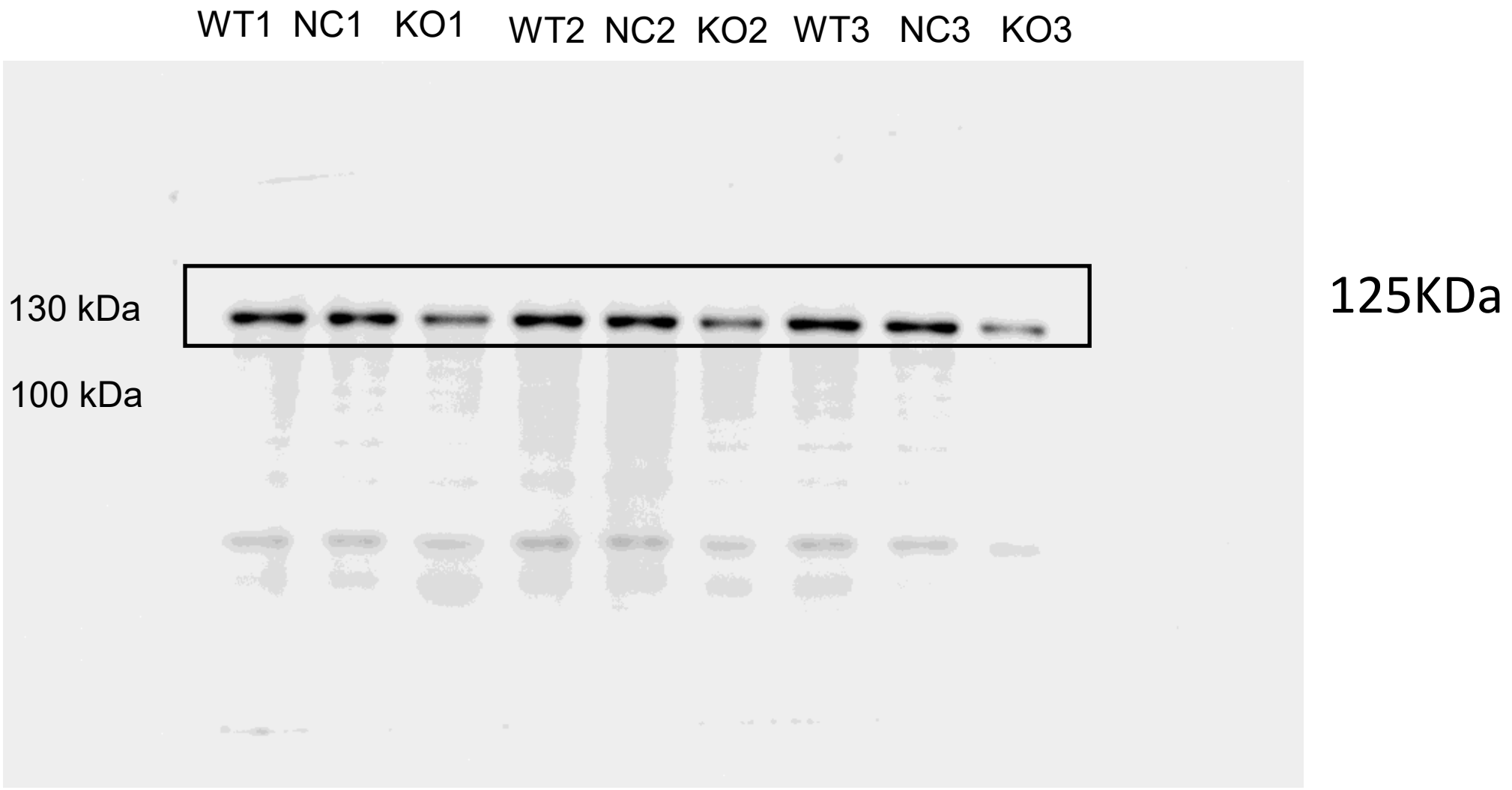

Figure S5 (KO-AKR1C2)

N-cadherin 130KDa

WT1 NC1 KO1 WT2 NC2 KO2 WT3 NC3 KO3

130 kDa

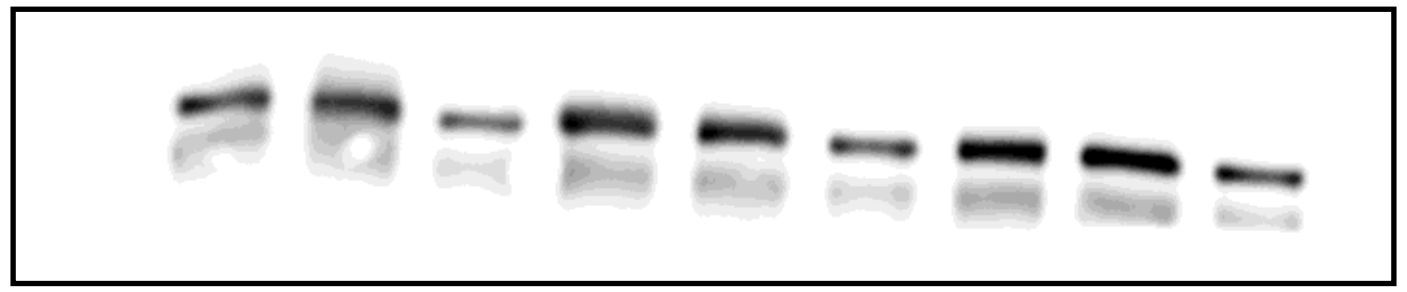

Integrin α4 70KDa

WT1 NC1 KO1 WT2 NC2 KO2 WT3 NC3 KO3

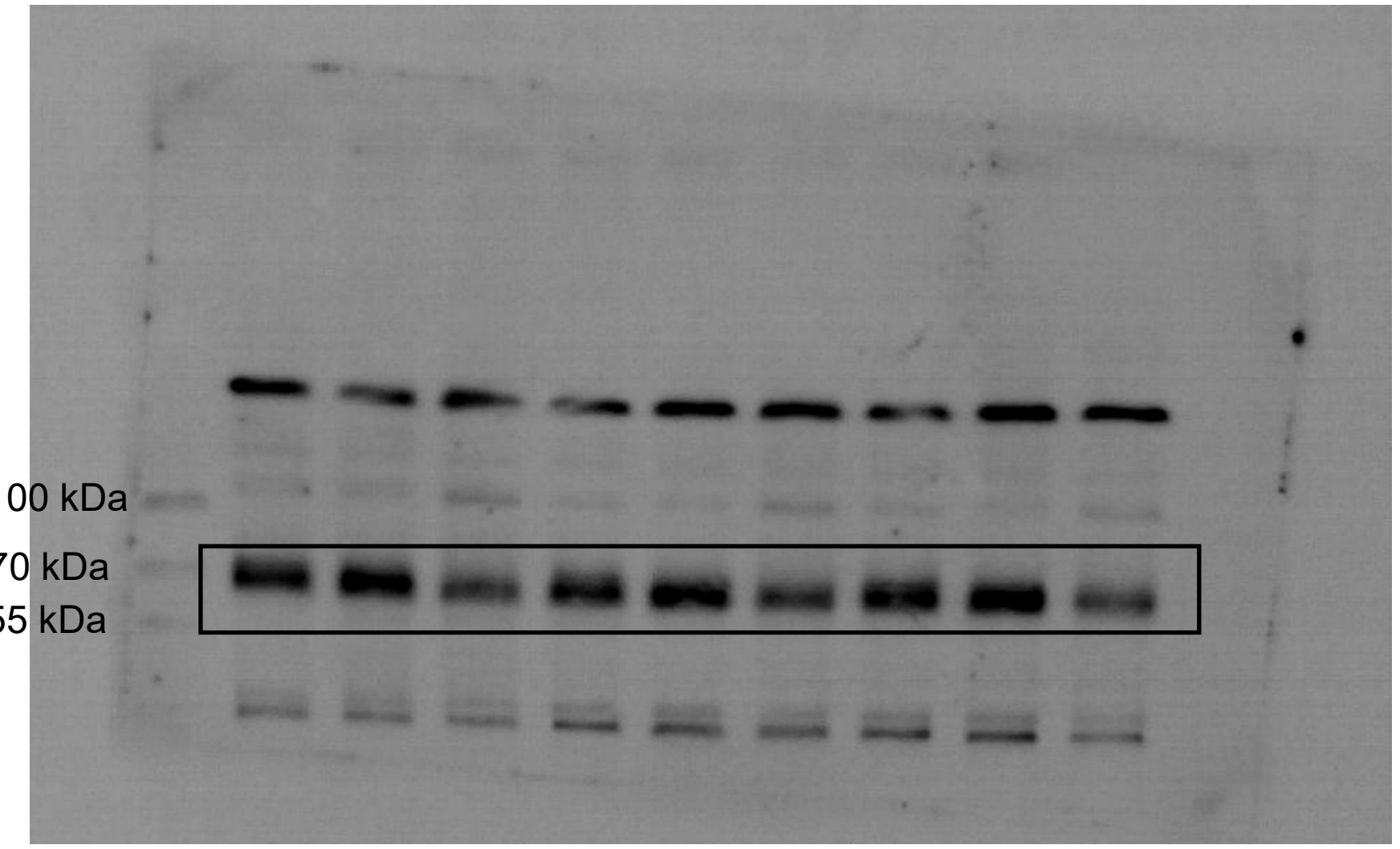

Figure S5 (OE-AKR1C2)

Integrin α1 200KDa

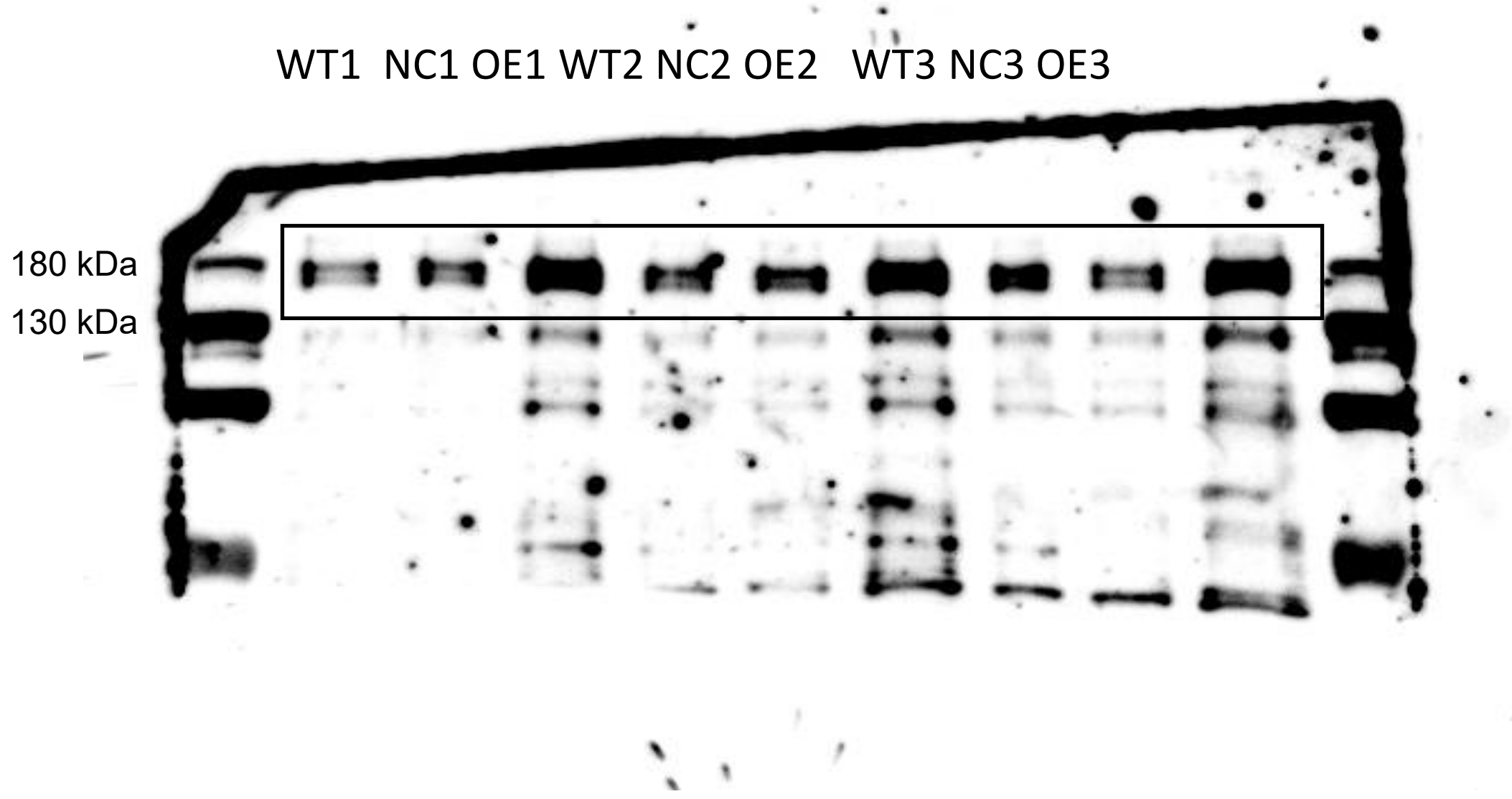

Vinculin 124KDa

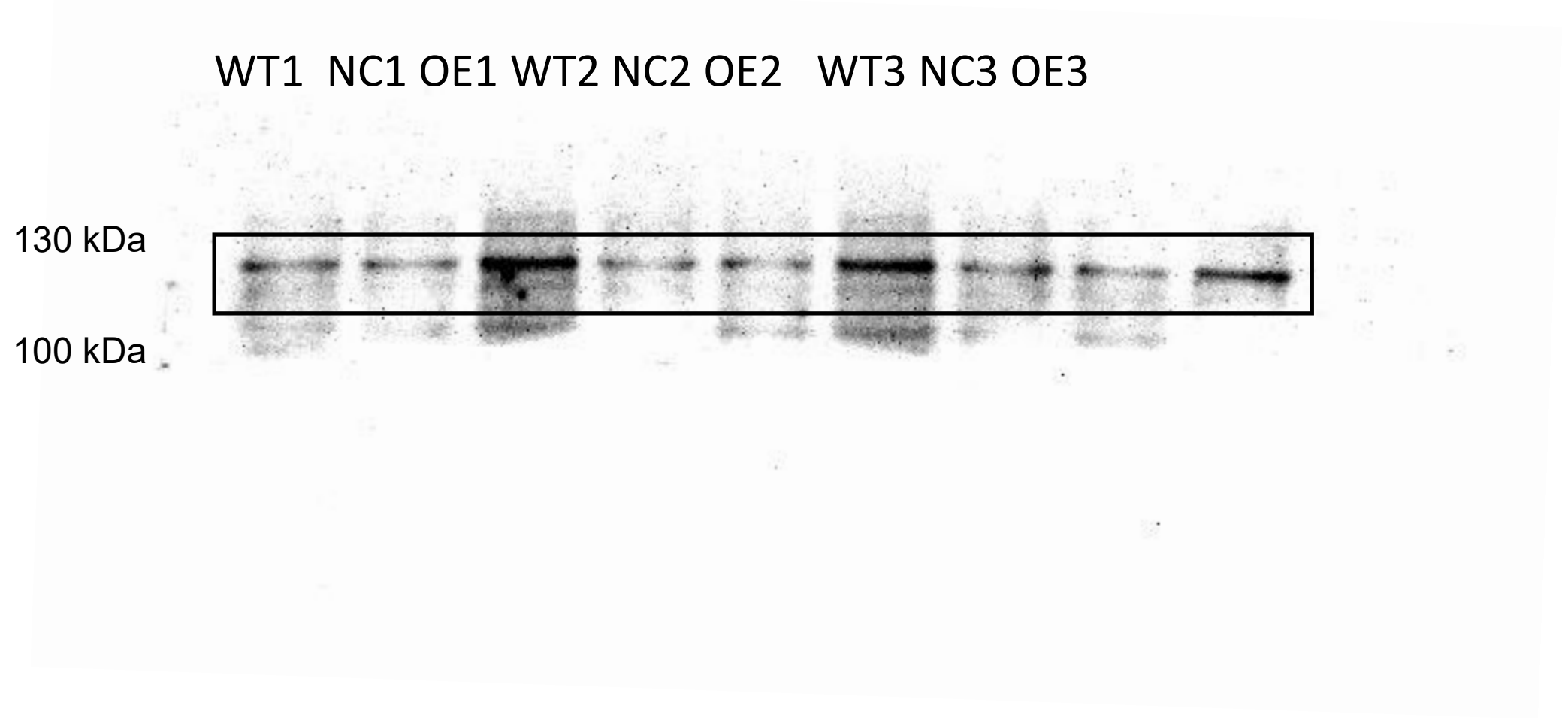

Figure S5 (OE-AKR1C2)

COL4A1 160KDa

WT1 NC1 OE1 WT2 NC2 OE2 WT3 NC3 OE3

180 kDa

130 kDa

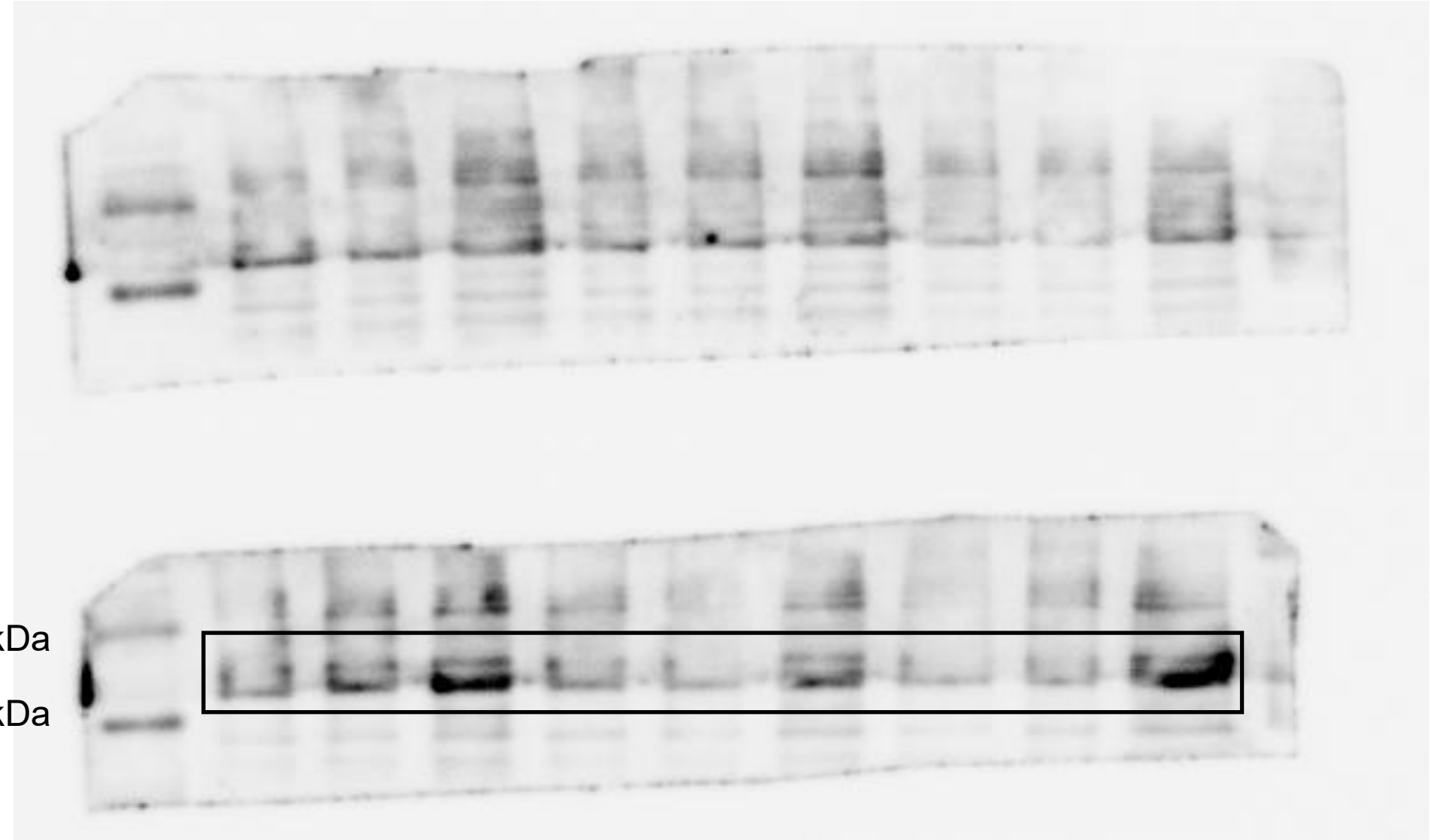

Collagen Ⅱ 134KDa

WT1 NC1 OE1 WT2 NC2 OE2 WT3 NC3 OE3

180 kDa

130 kDa

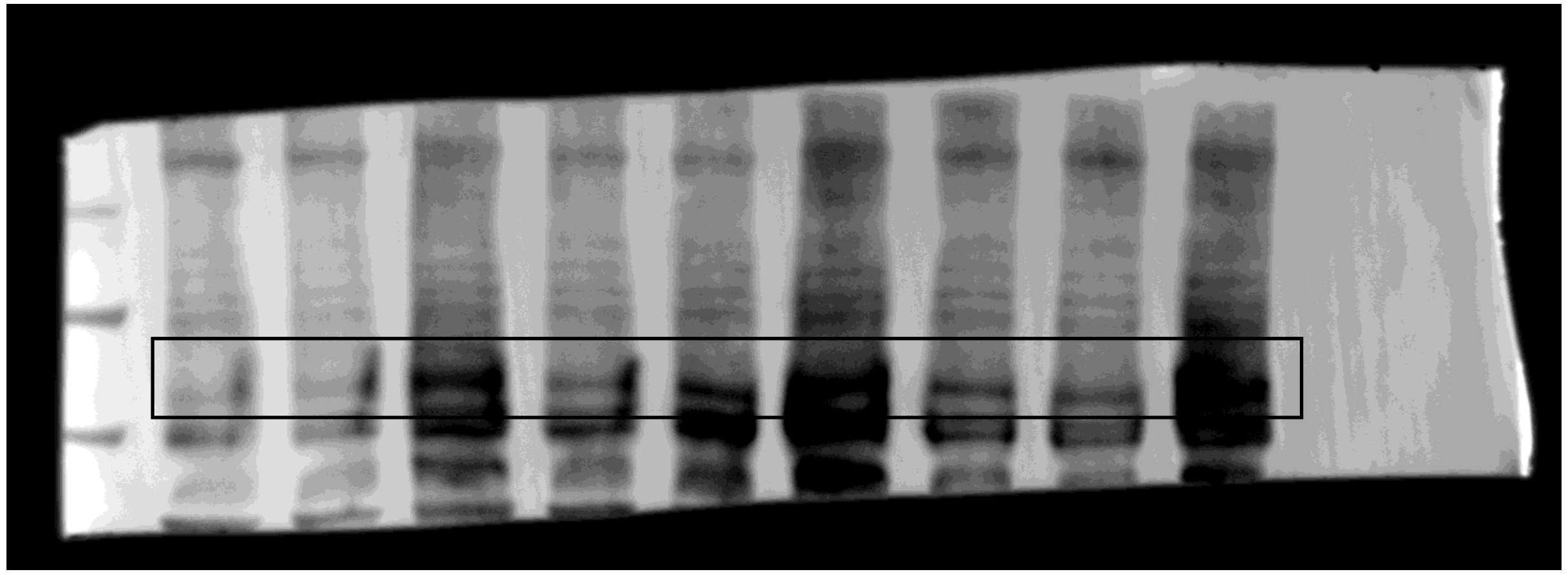

Figure S5 (OE-AKR1C2)

p-FAK 125KDa

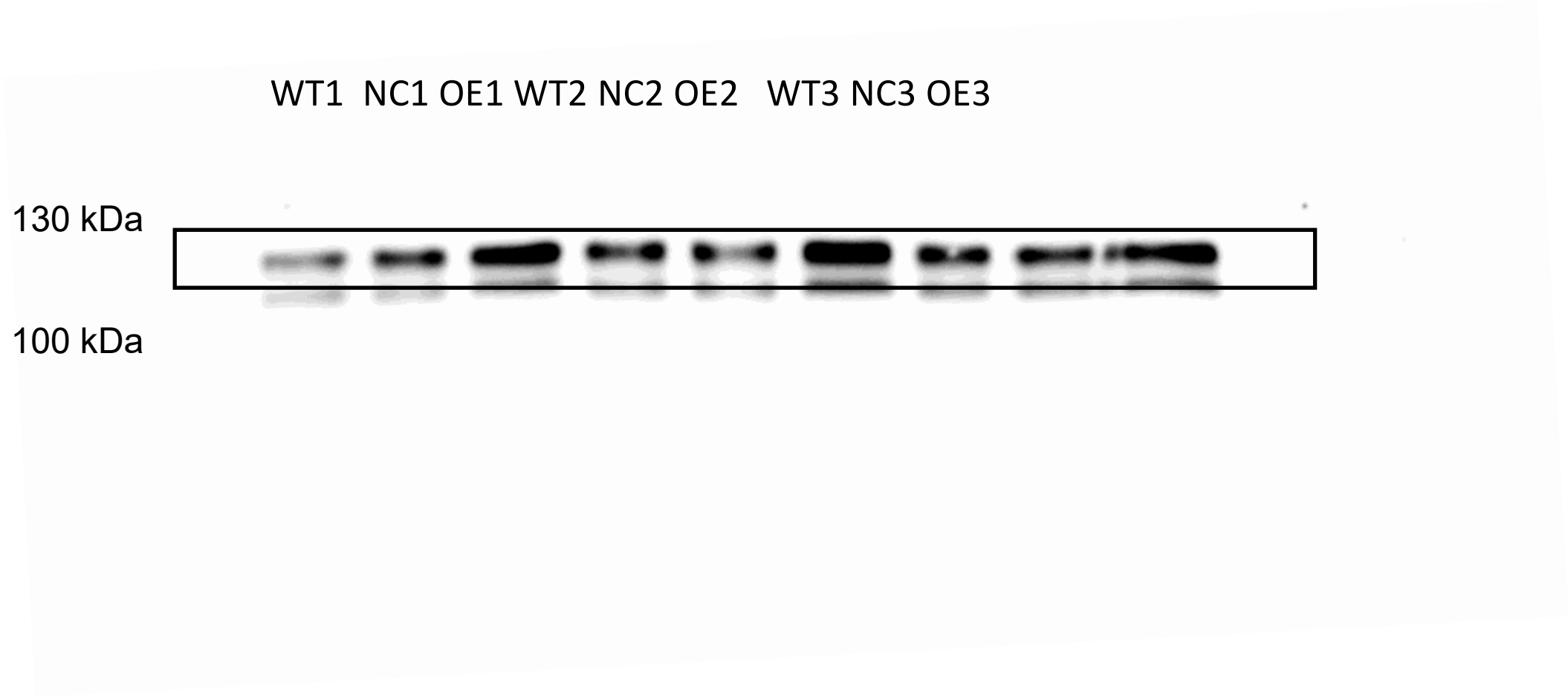

FAK 125KDa

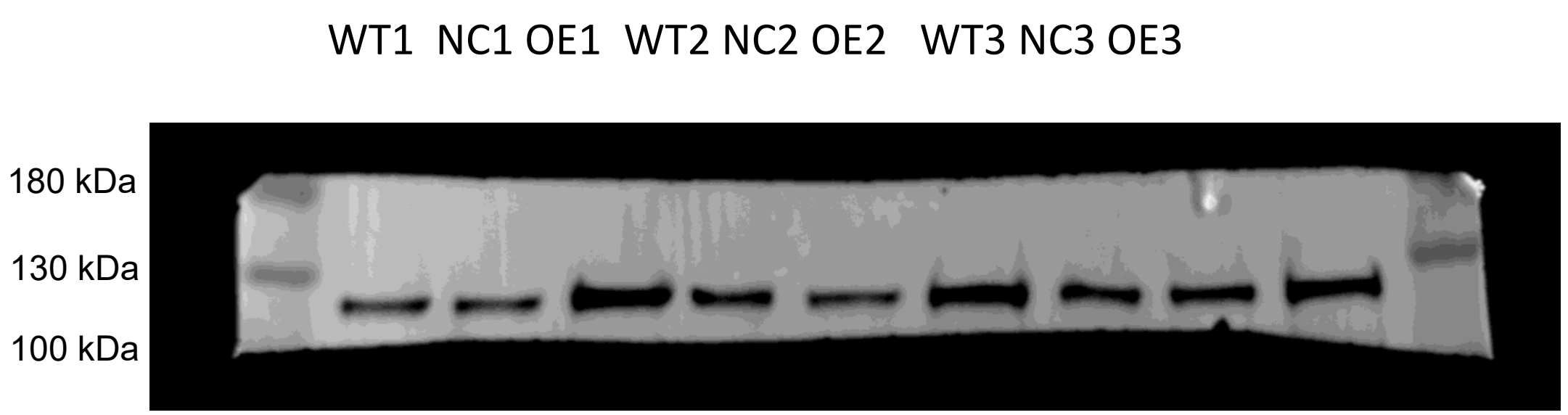

Figure S5 (OE-AKR1C2)

Talin1 270KDa

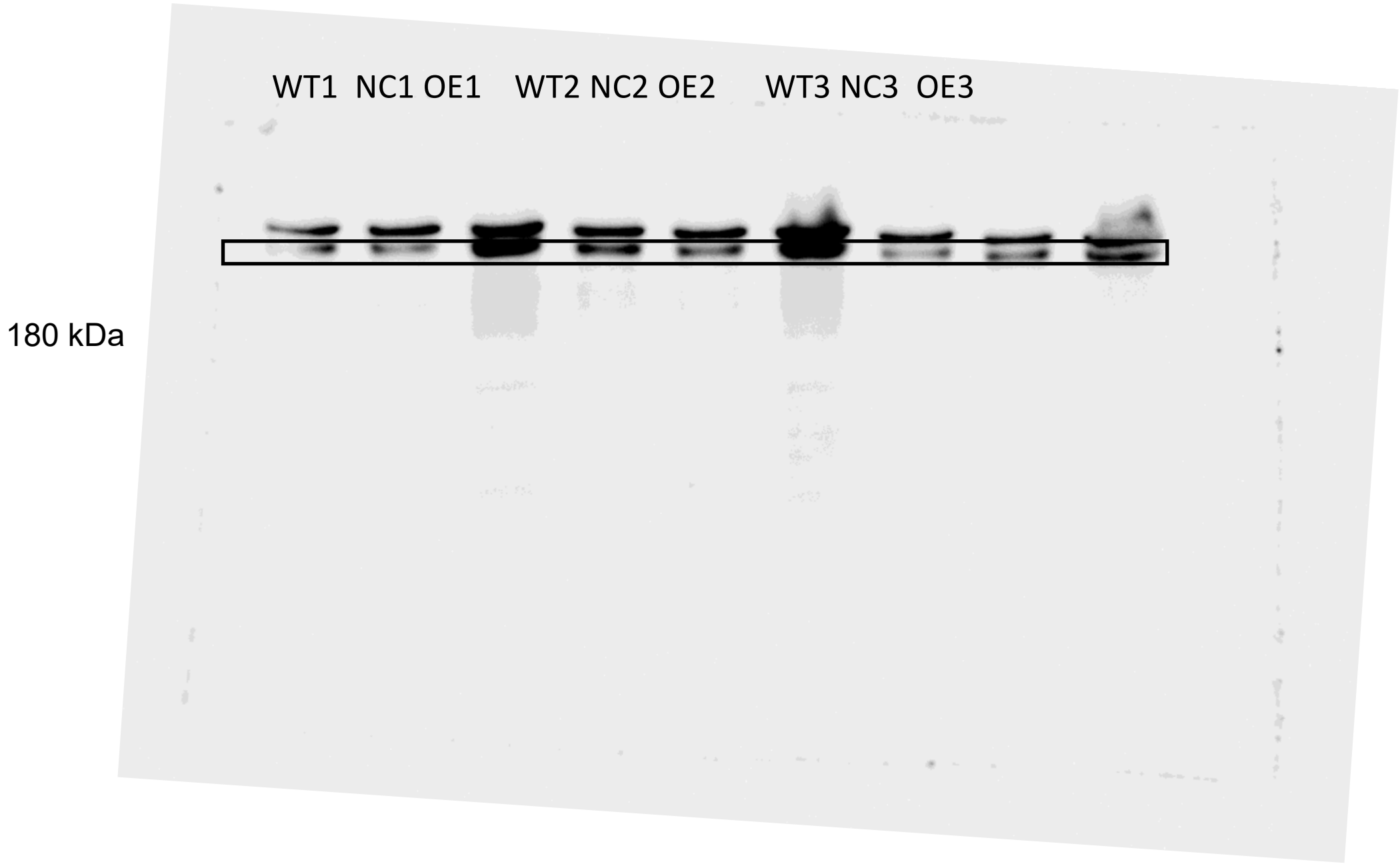

Tensin2 145KDa

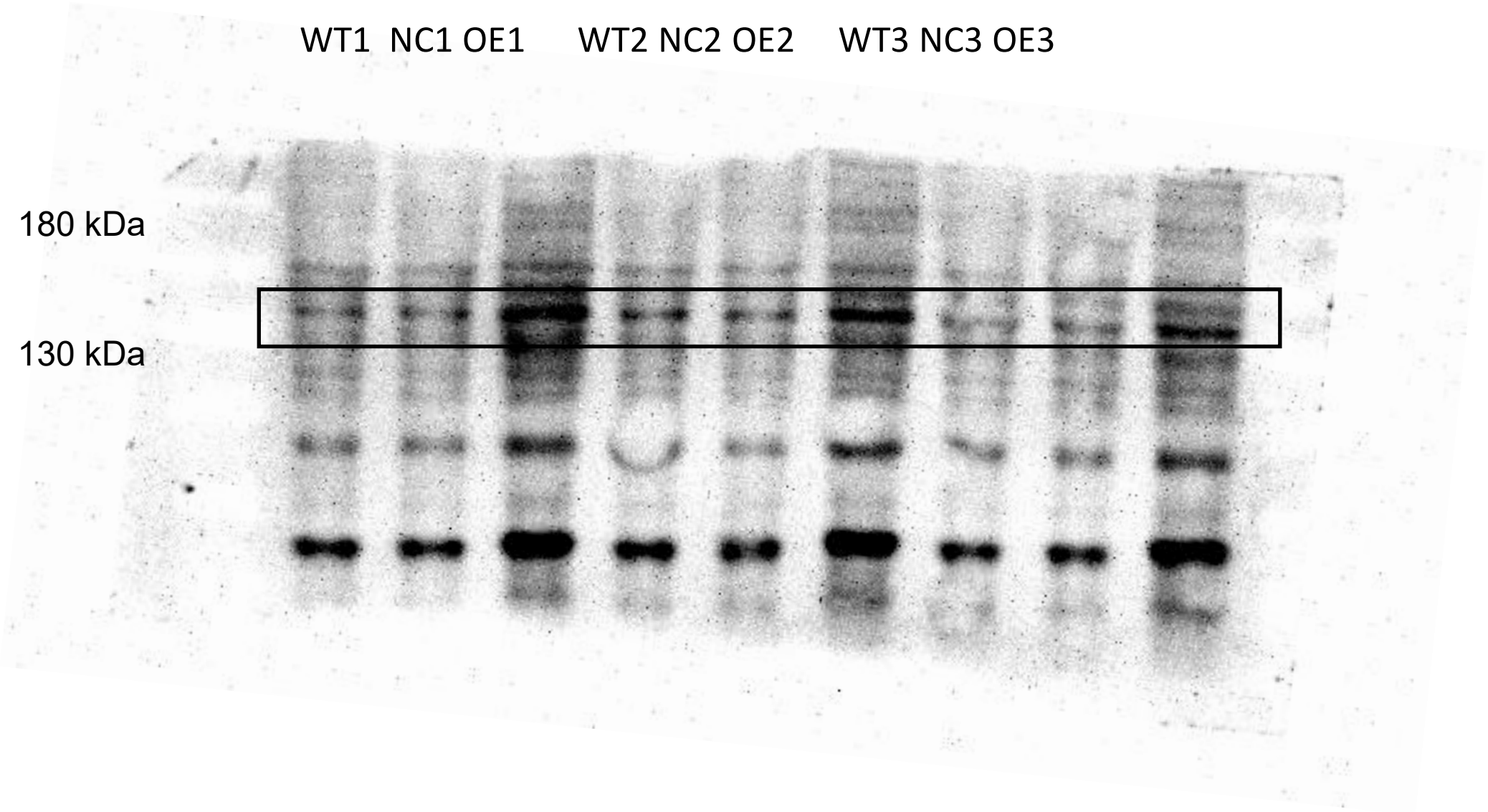

Figure S5 (OE-AKR1C2)

Vimentin 54KDa

WT1 NC1 OE1 WT2 NC2 OE2 WT3 NC3 OE3

55 kDa

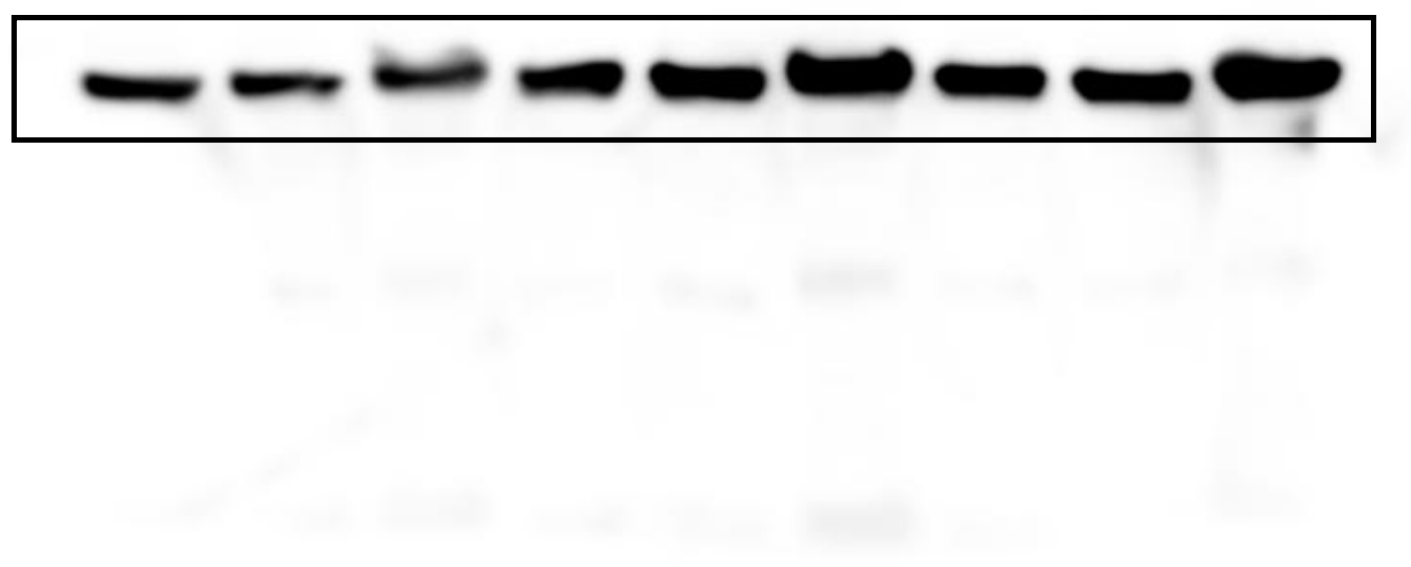

E-cadherin 125KDa

WT1 NC1 OE1 WT2 NC2 OE2 WT3 NC3 OE3

130 kDa

100 kDa

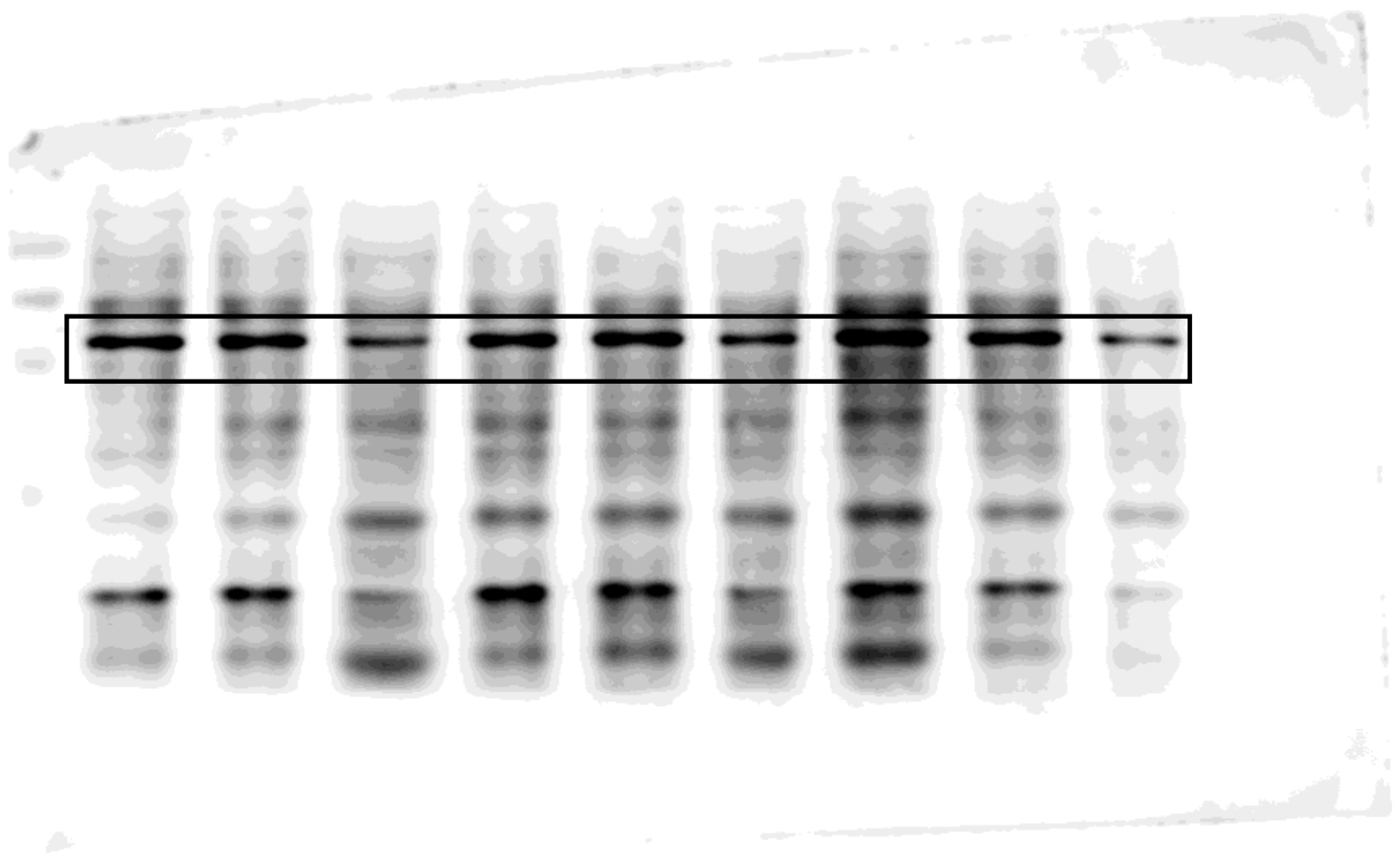

Figure S5 (OE-AKR1C2)

N-cadherin 130KDa

WT1 NC1 OE1 WT2 NC2 OE2 WT3 NC3 OE3

130 kDa

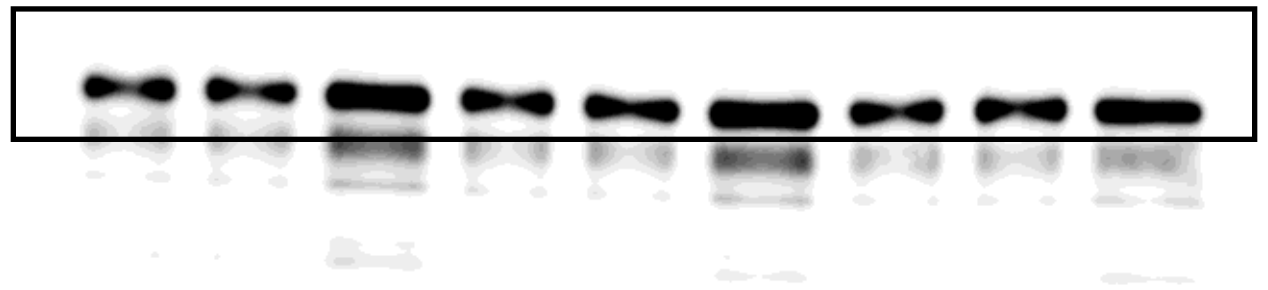

Integrin β1 135KDa

WT1 NC1 OE1 WT2 NC2 OE2 WT3 NC3 OE3

130 kDa

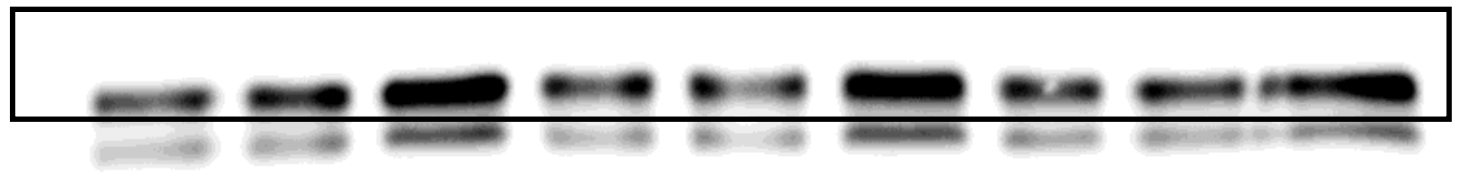

Figure S5 (OE-AKR1C2)

ZEB1 220KDa

WT1 NC1 OE1 WT2 NC2 OE2 WT3 NC3 OE3

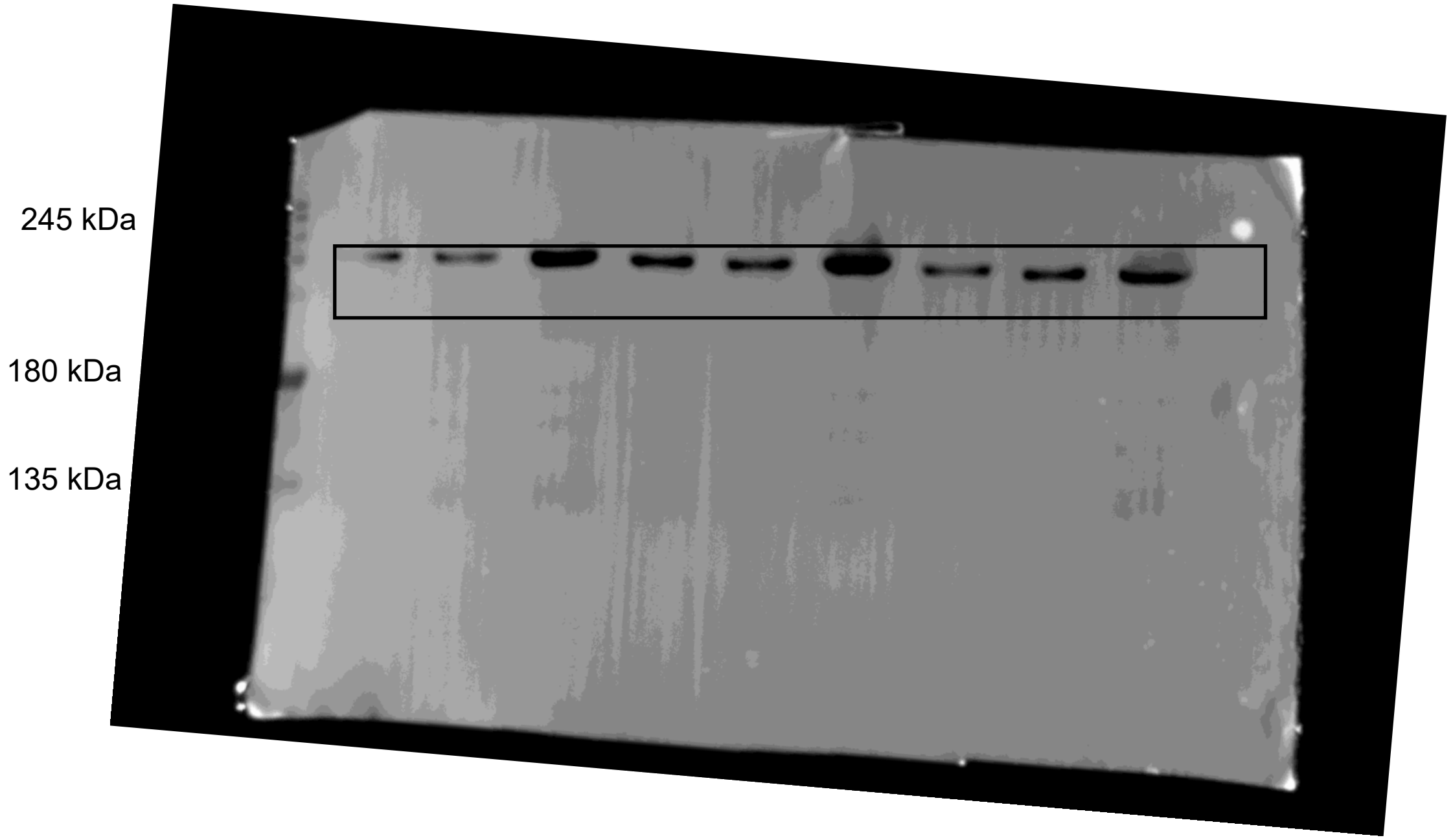

Collagen Ⅰ 220KDa

WT1 NC1 OE1 WT2 NC2 OE2 WT3 NC3 OE3

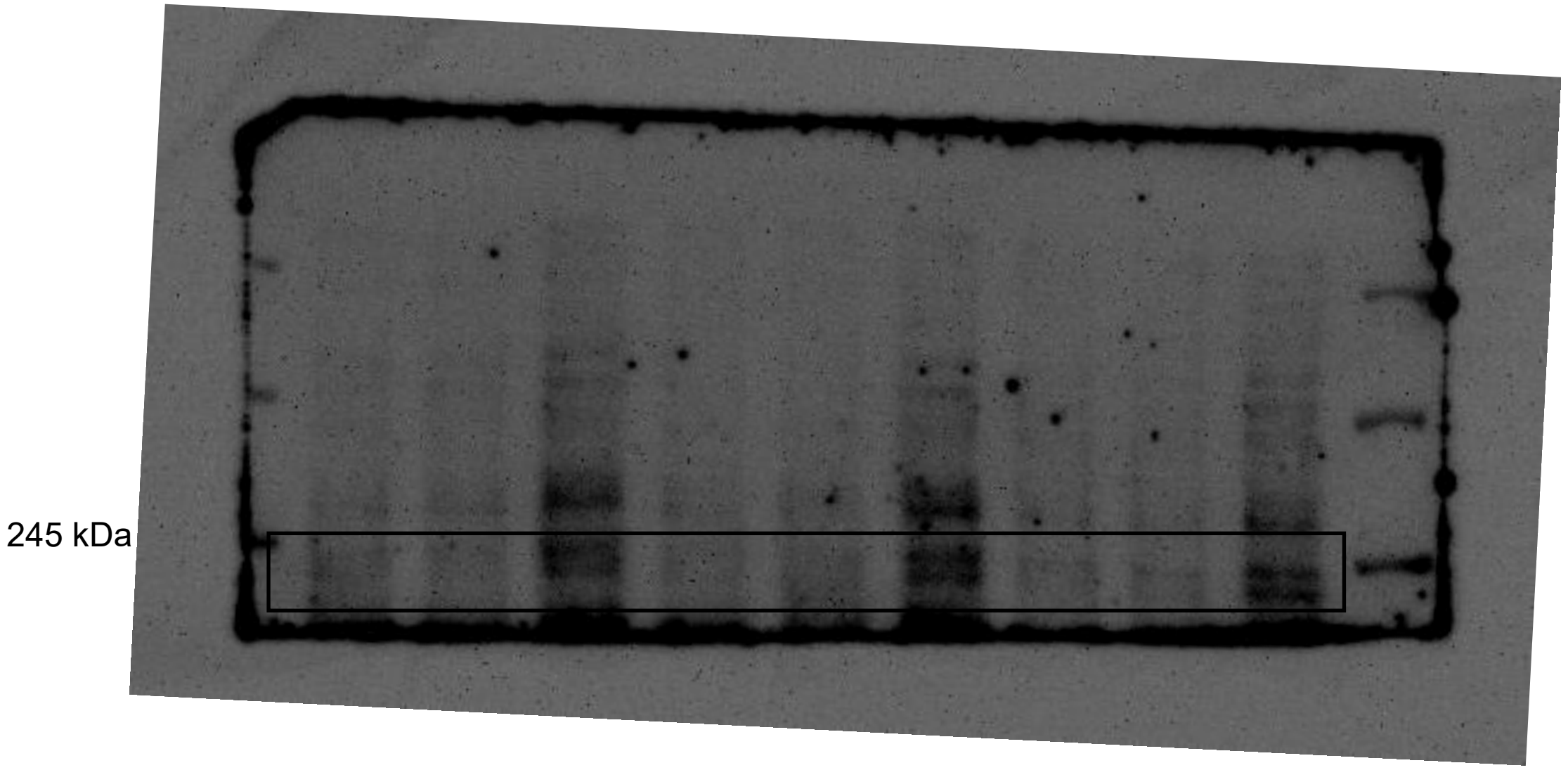

Figure S5 (OE-AKR1C2)

Integrin α4 70KDa

WT1 NC1 OE1 WT2 NC2 OE2 WT3 NC3 OE3

70 kDa

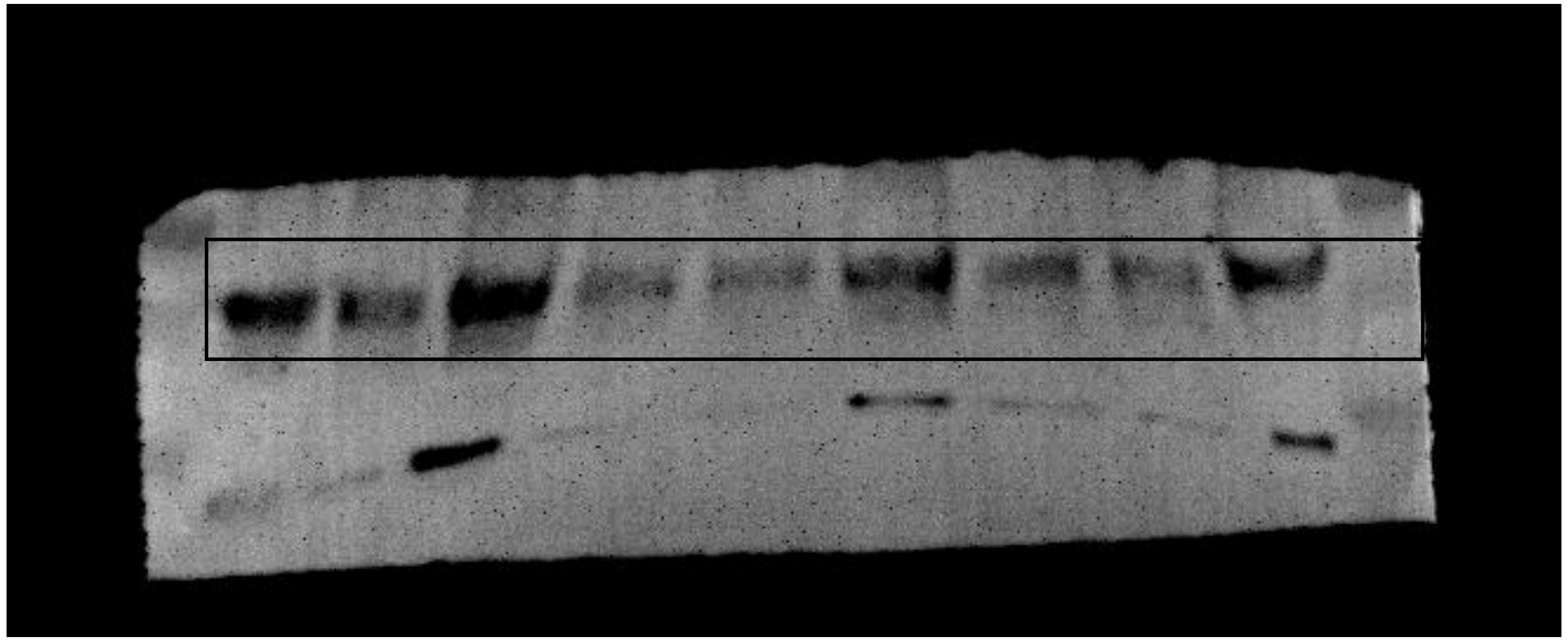

ILK 59KDa

WT1 NC1 OE1 WT2 NC2 OE2 WT3 NC3 OE3

70 kDa

55 kDa

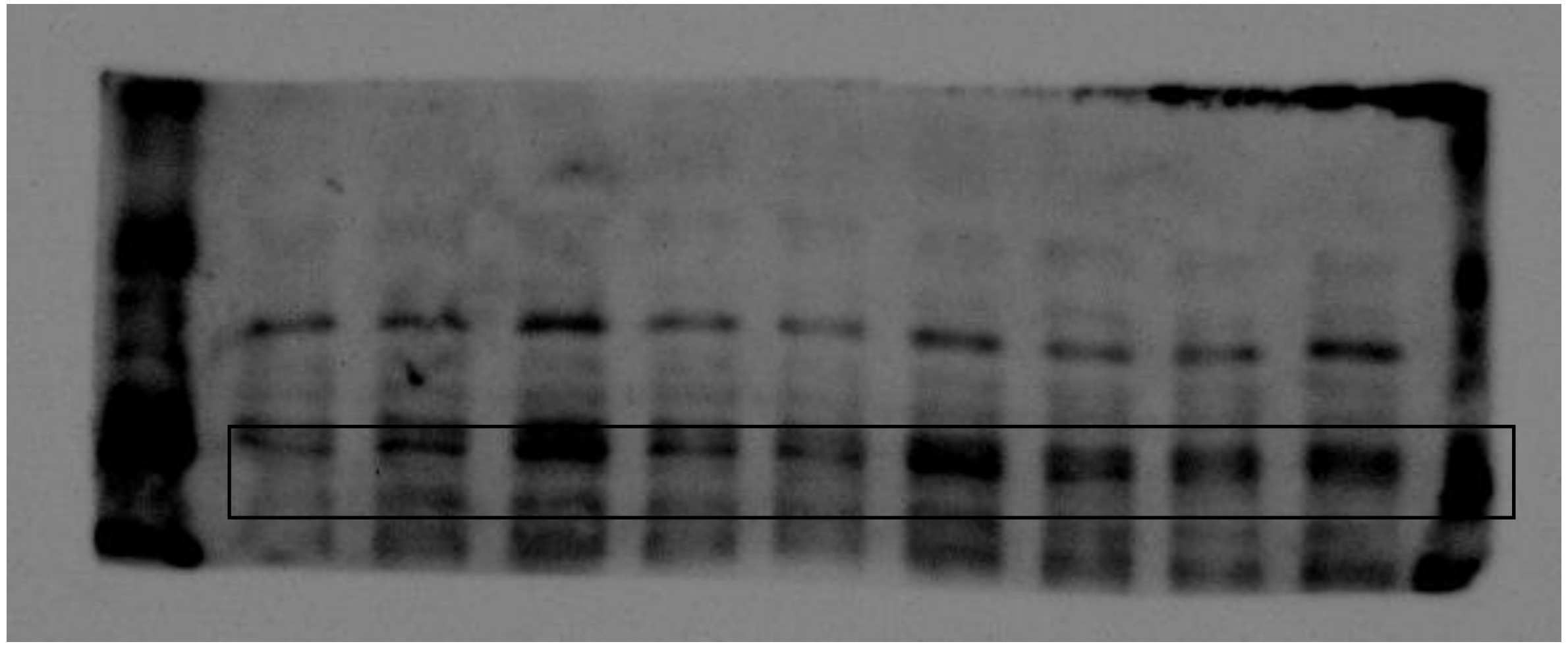

Figure S5 (OE-AKR1C2)

Collagen Ⅲ 200KDa

WT1 NC1 OE1 WT2 NC2 OE2 WT3 NC3 OE3

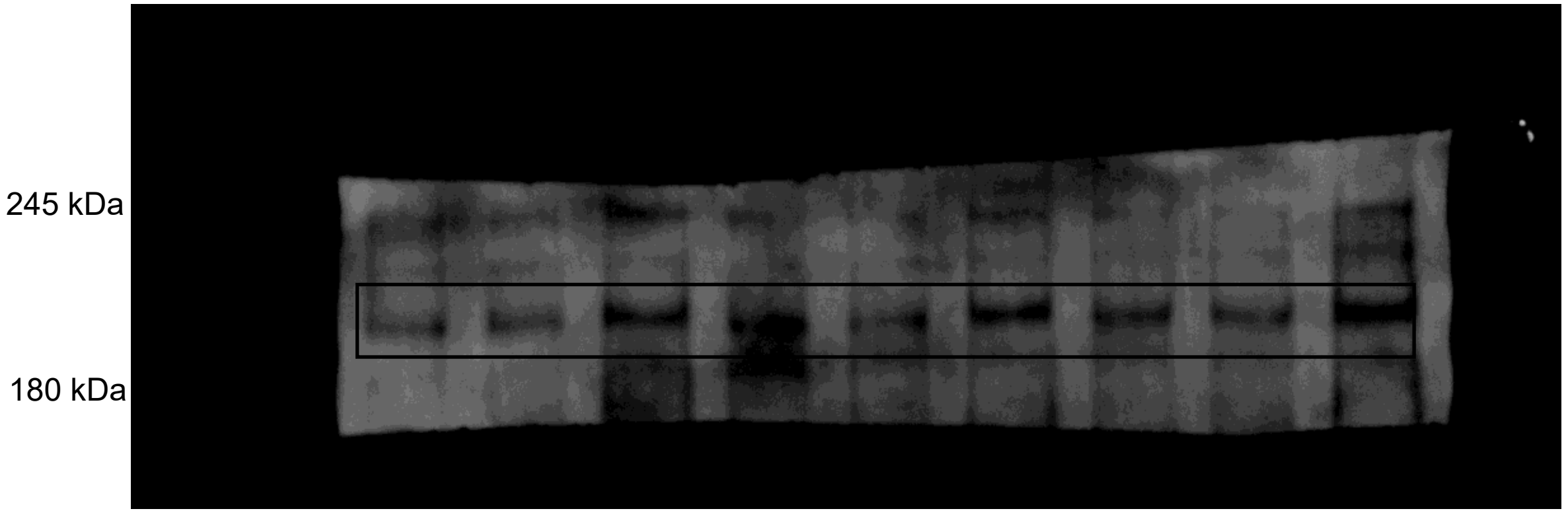

Figure S5 (OE-AKR1C2)

snail+slug 29KDa

WT1 NC1 OE1 WT2 NC2 OE2 WT3 NC3 OE3

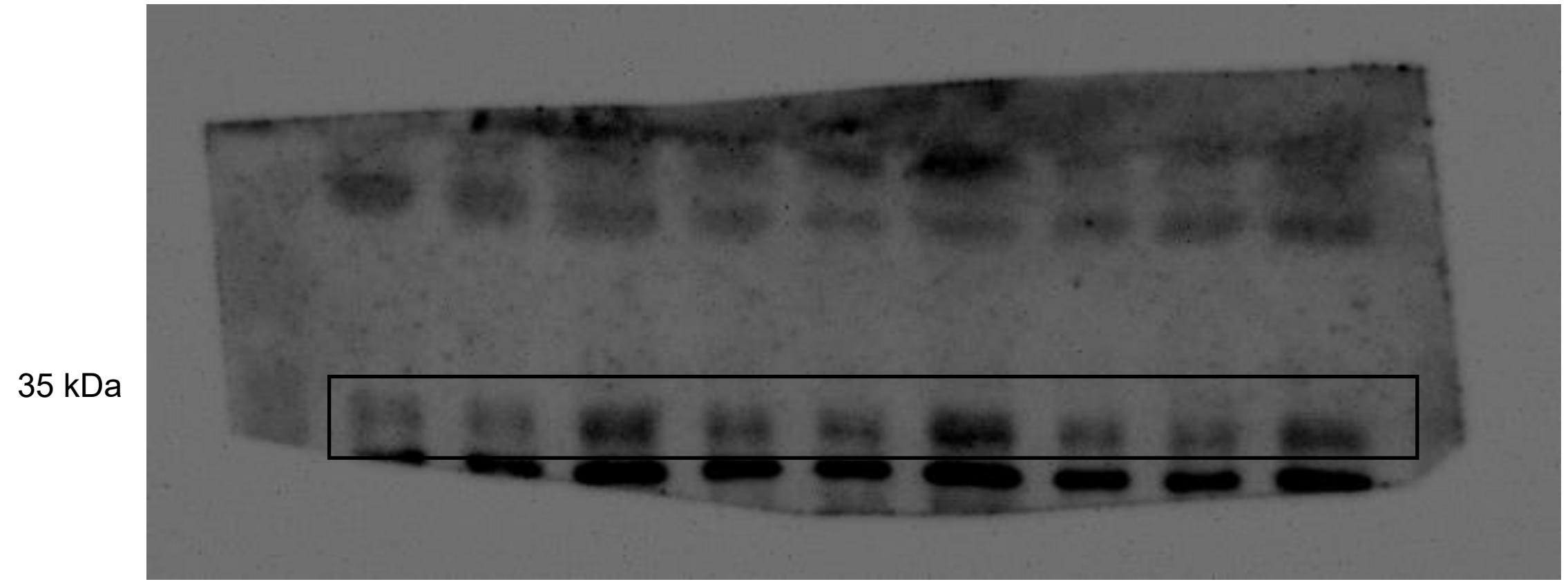

p-Paxilin 68KDa

WT1 NC1 OE1 WT2 NC2 OE2 WT3 NC3 OE3

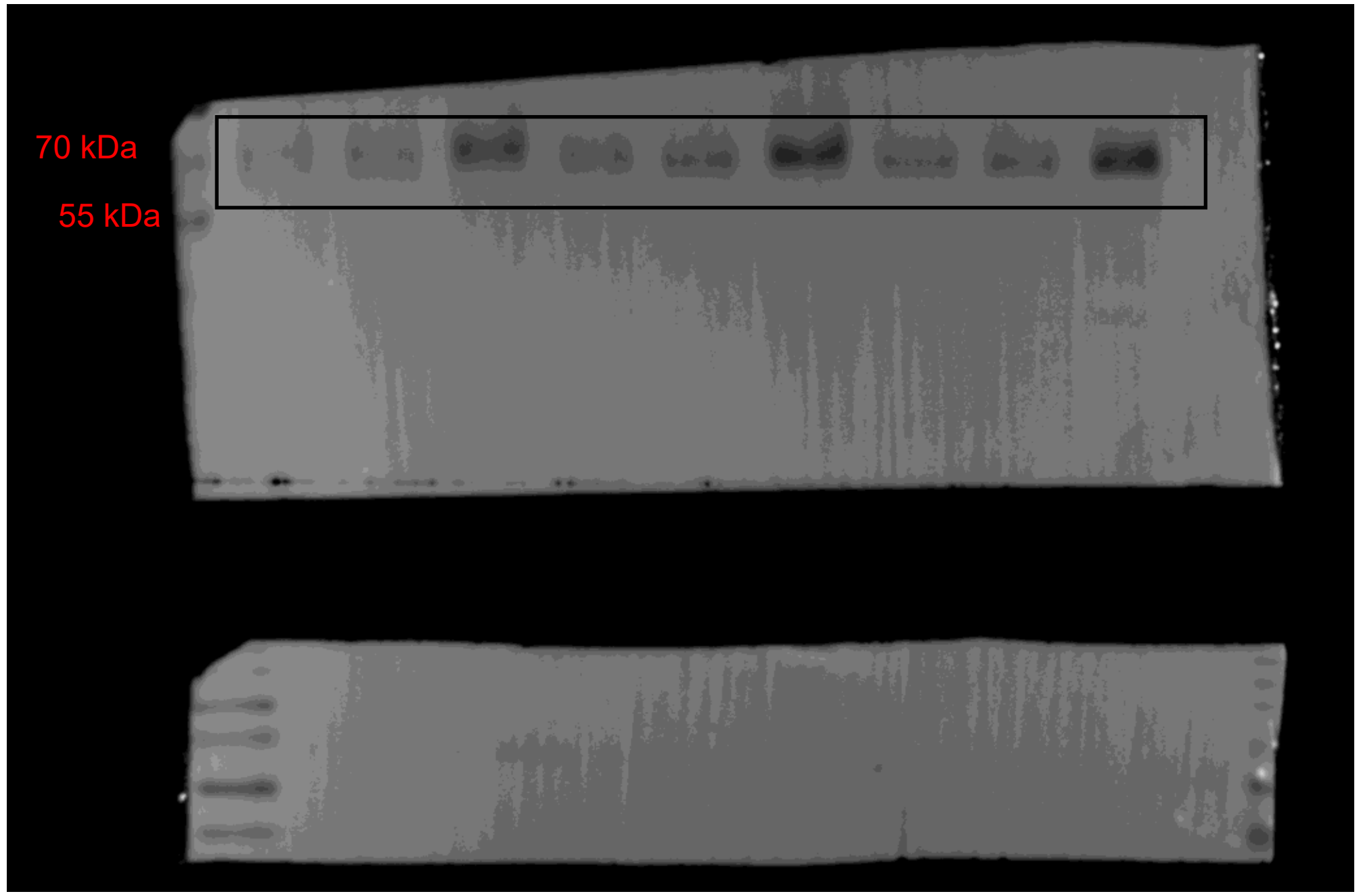

Figure S5 (OE-AKR1C2)

Paxilin 68KDa

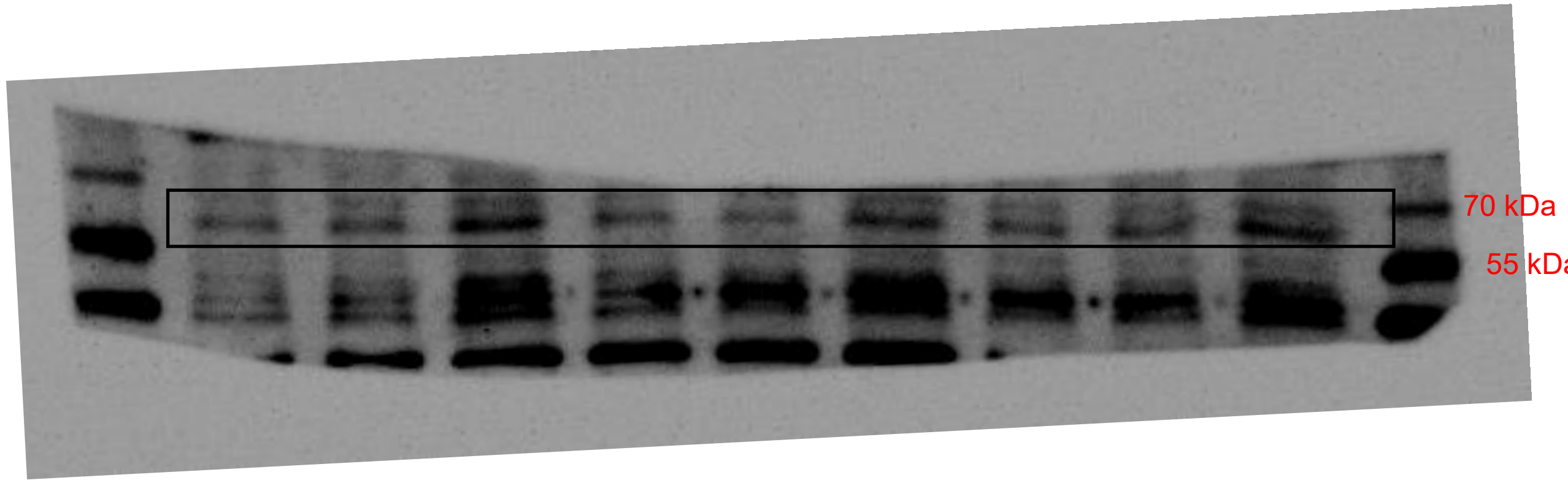

Twist1 21KDa

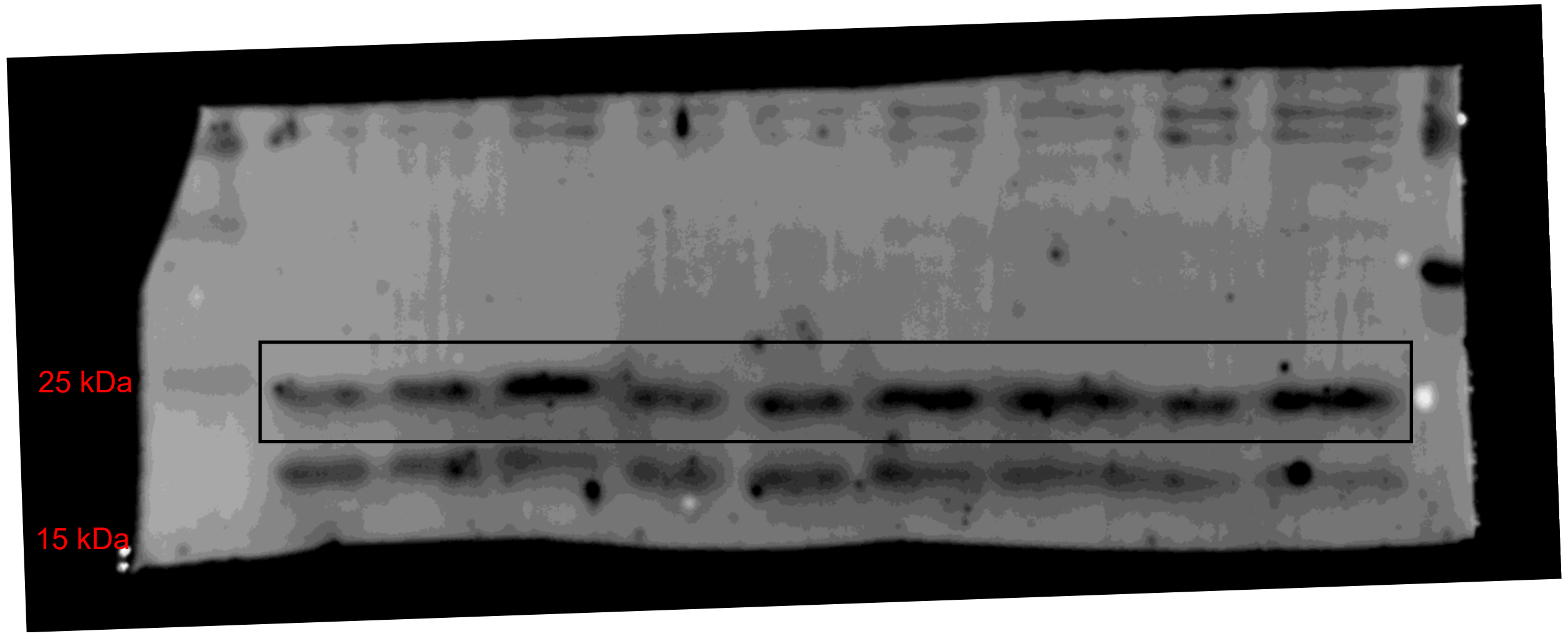

Figure S5 (OE-AKR1C2)

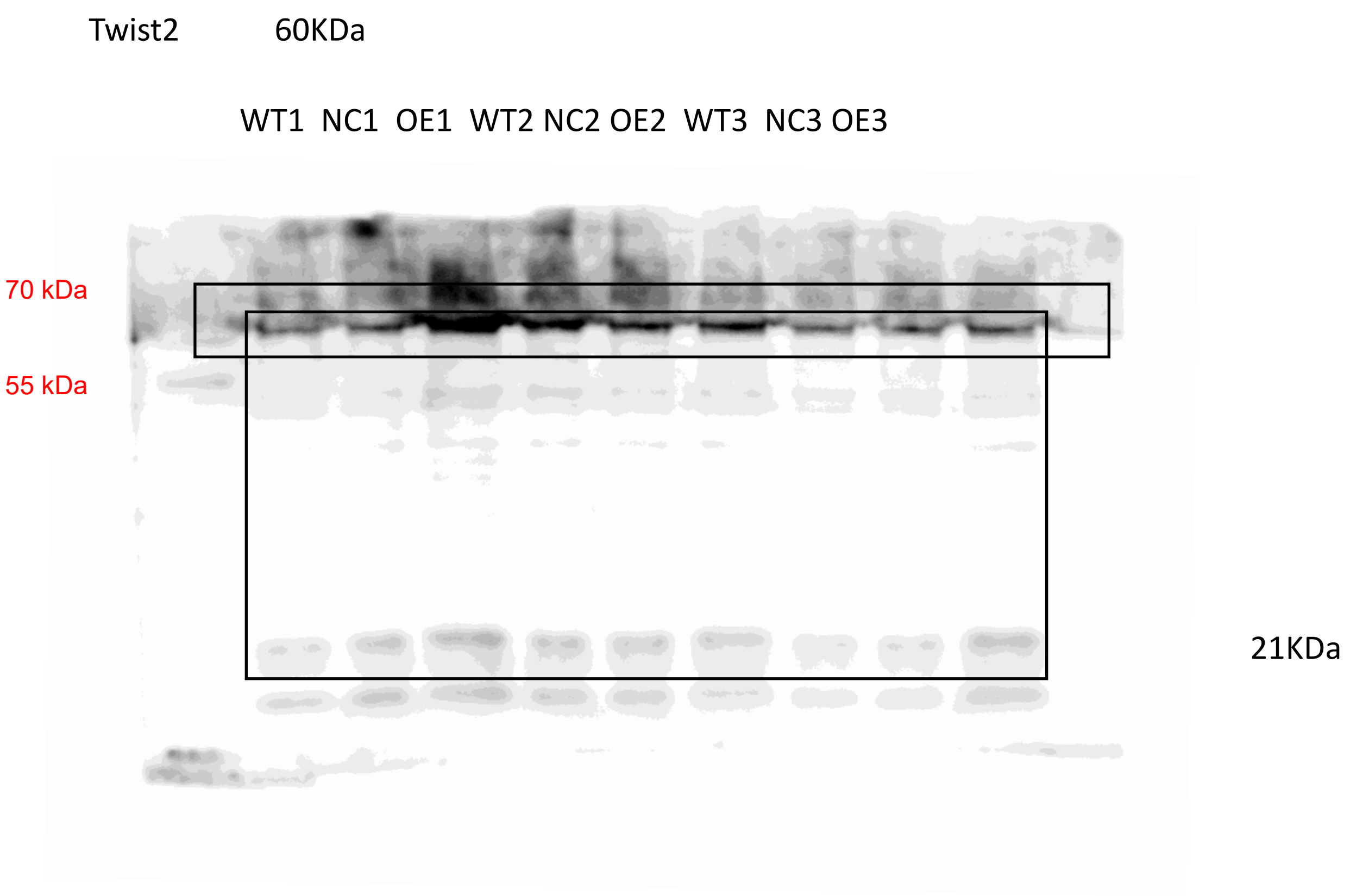

Figure S6 (KO-AKR1C2)

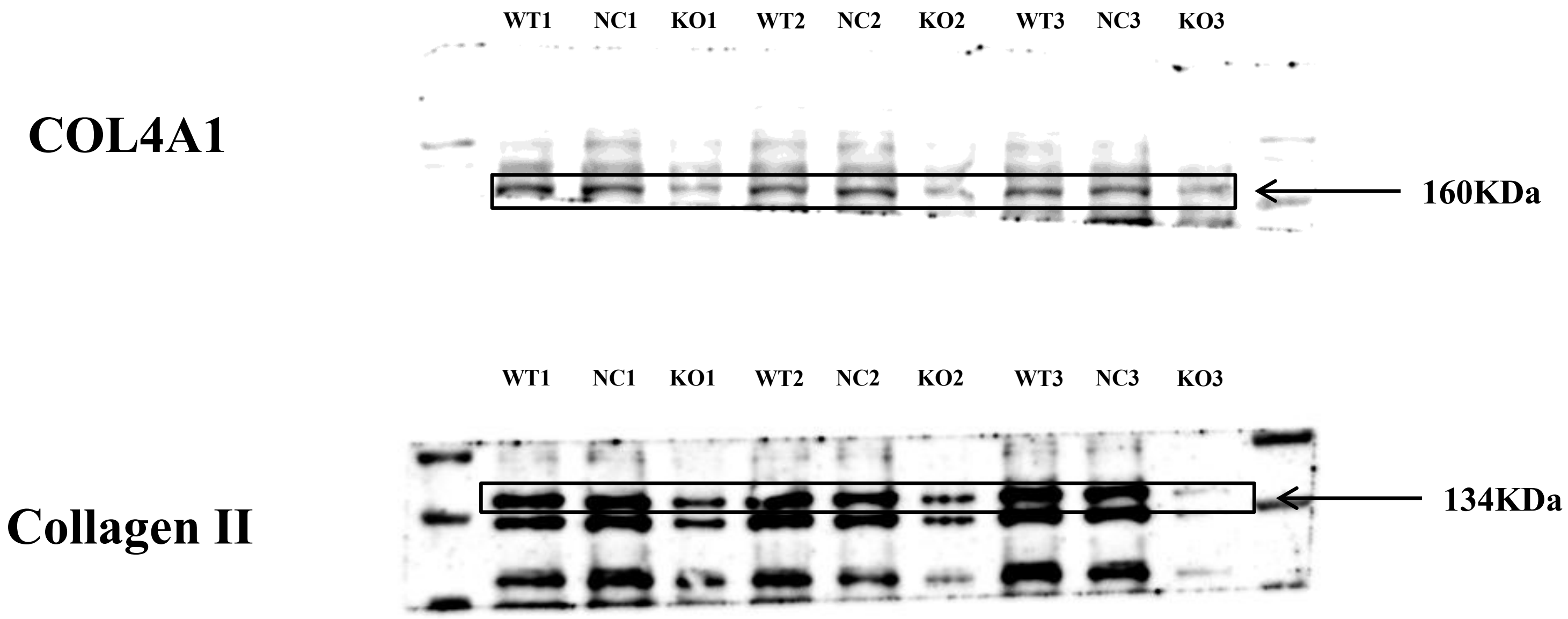

Figure S6 (KO-AKR1C2)

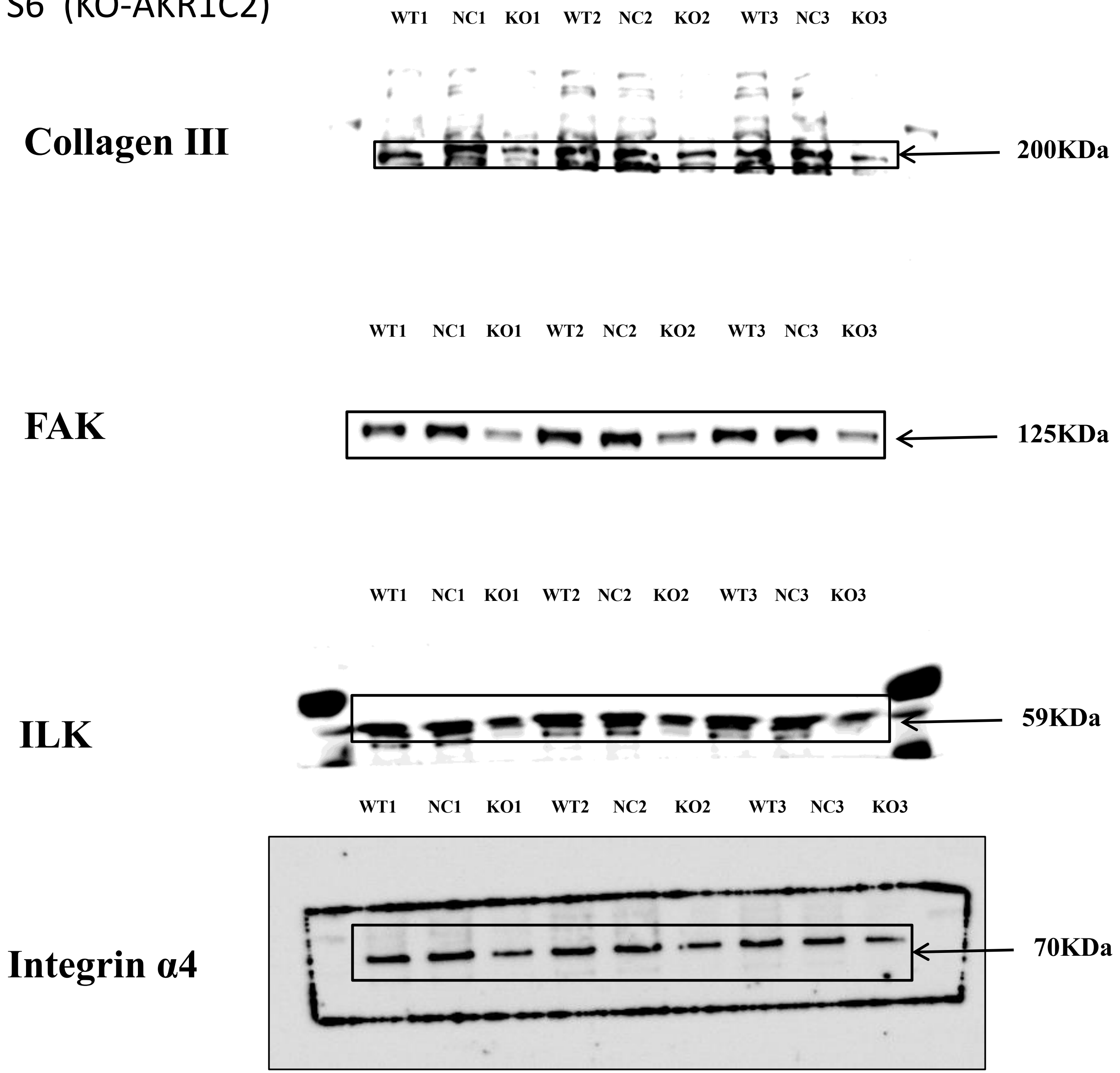

Figure S6 (KO-AKR1C2)

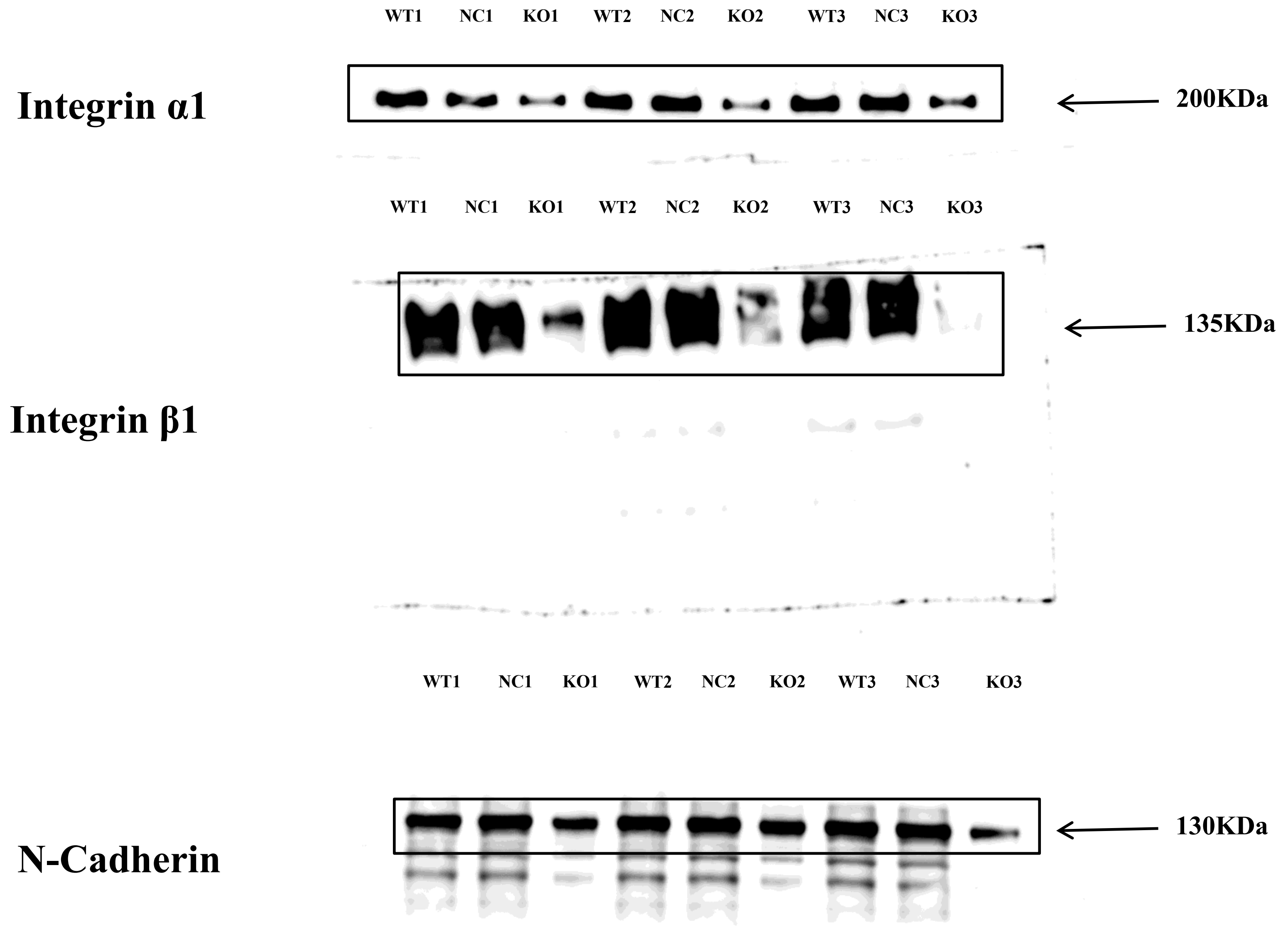

Figure S6 (KO-AKR1C2)

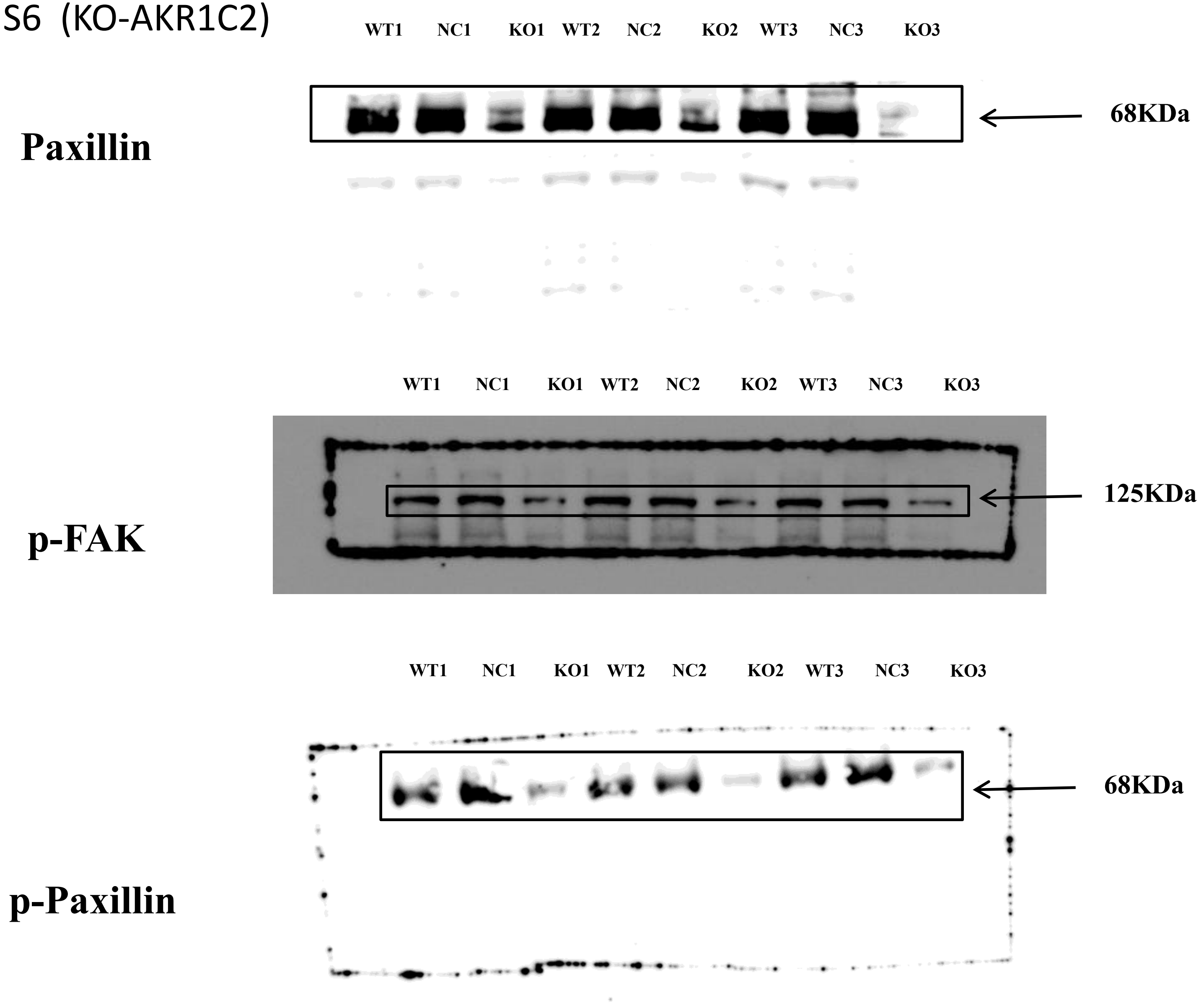

Figure S6 (KO-AKR1C2)

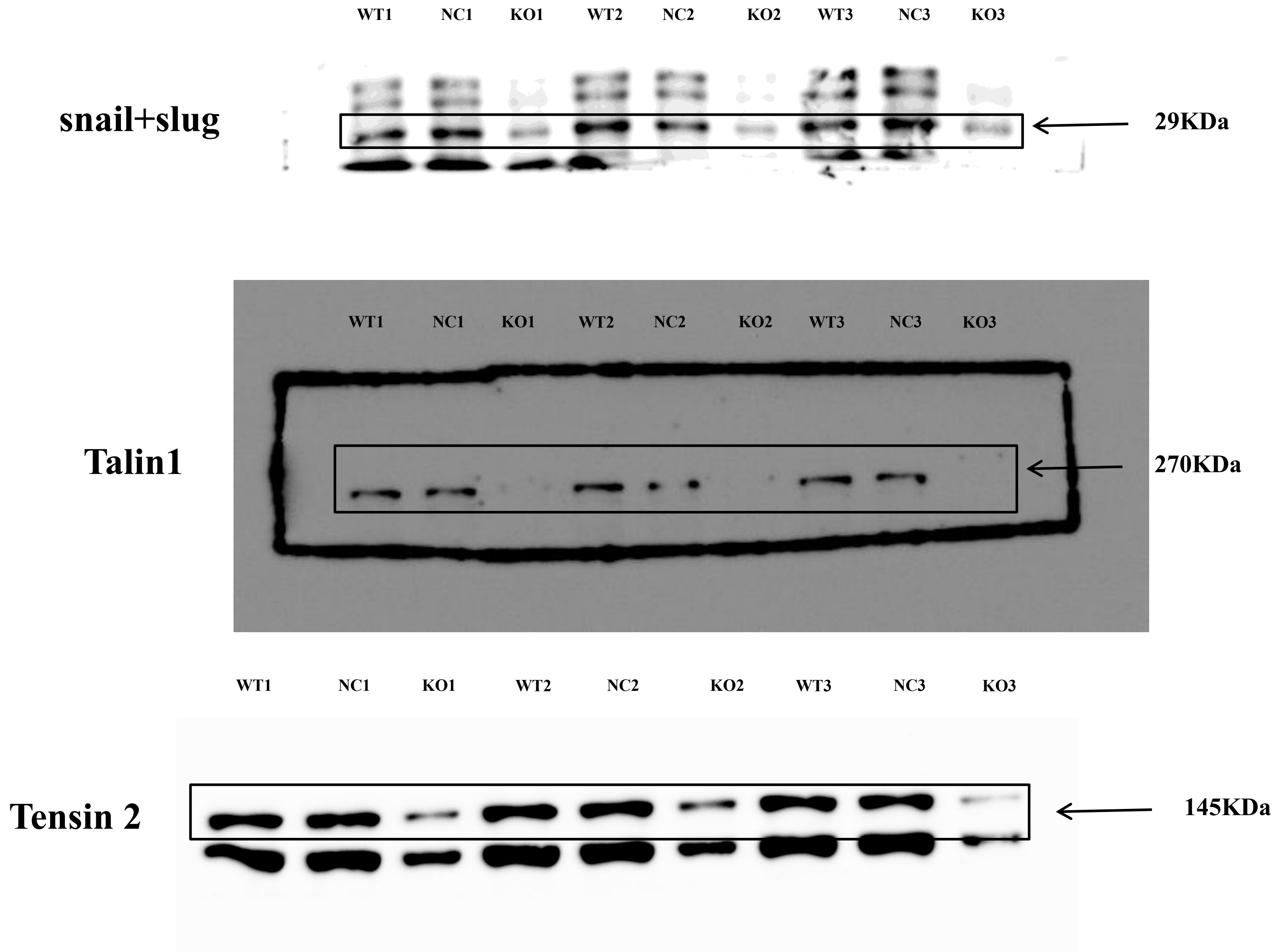

Figure S6 (KO-AKR1C2)

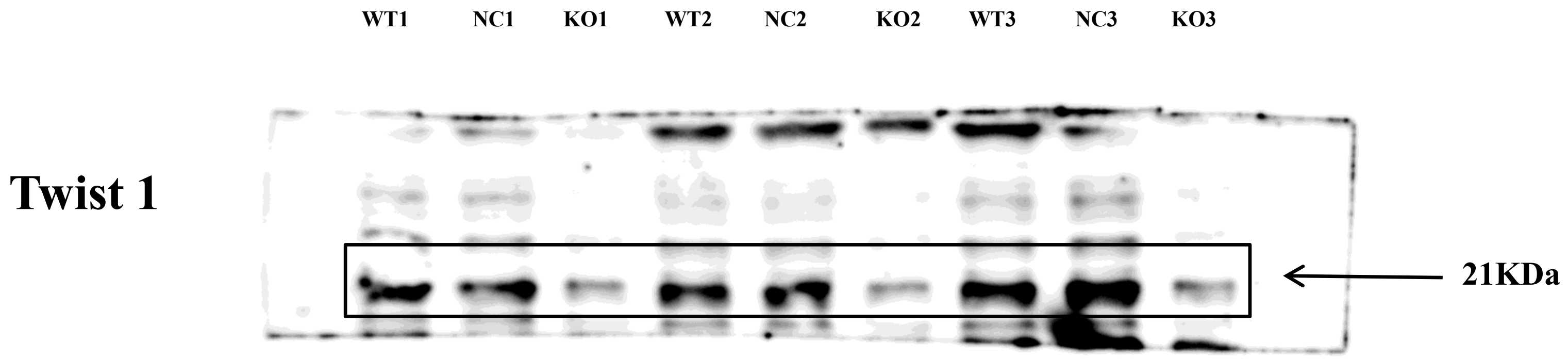

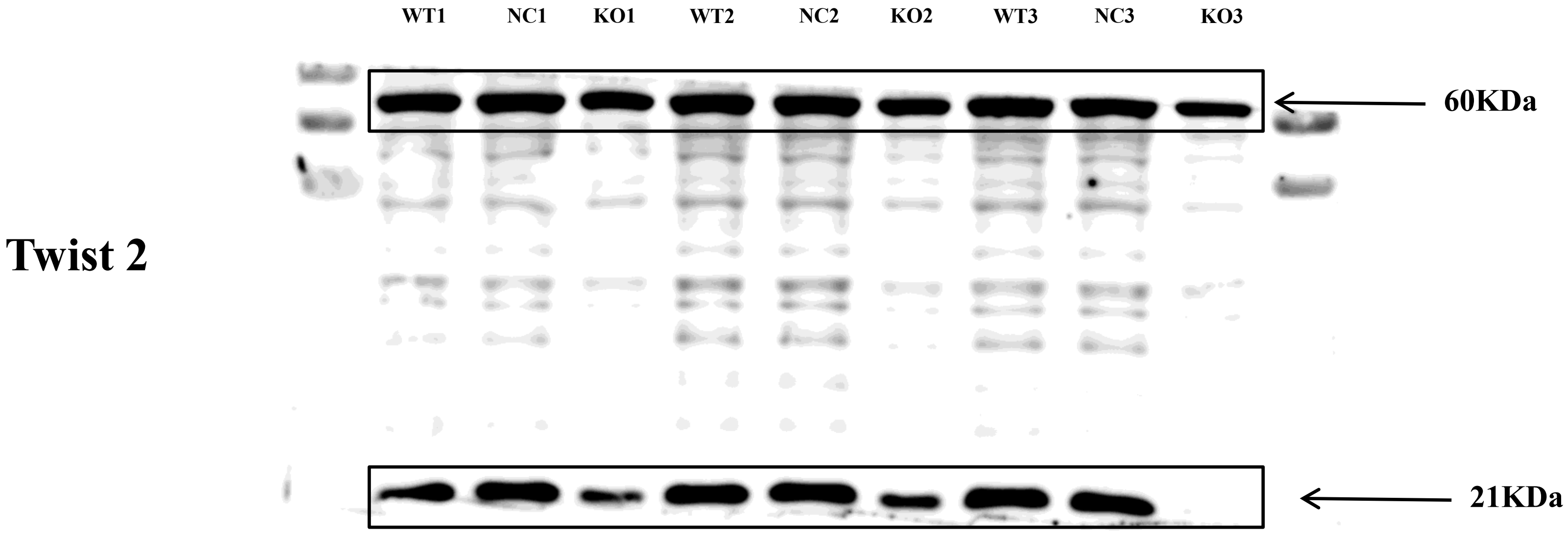

Figure S6 (KO-AKR1C2)

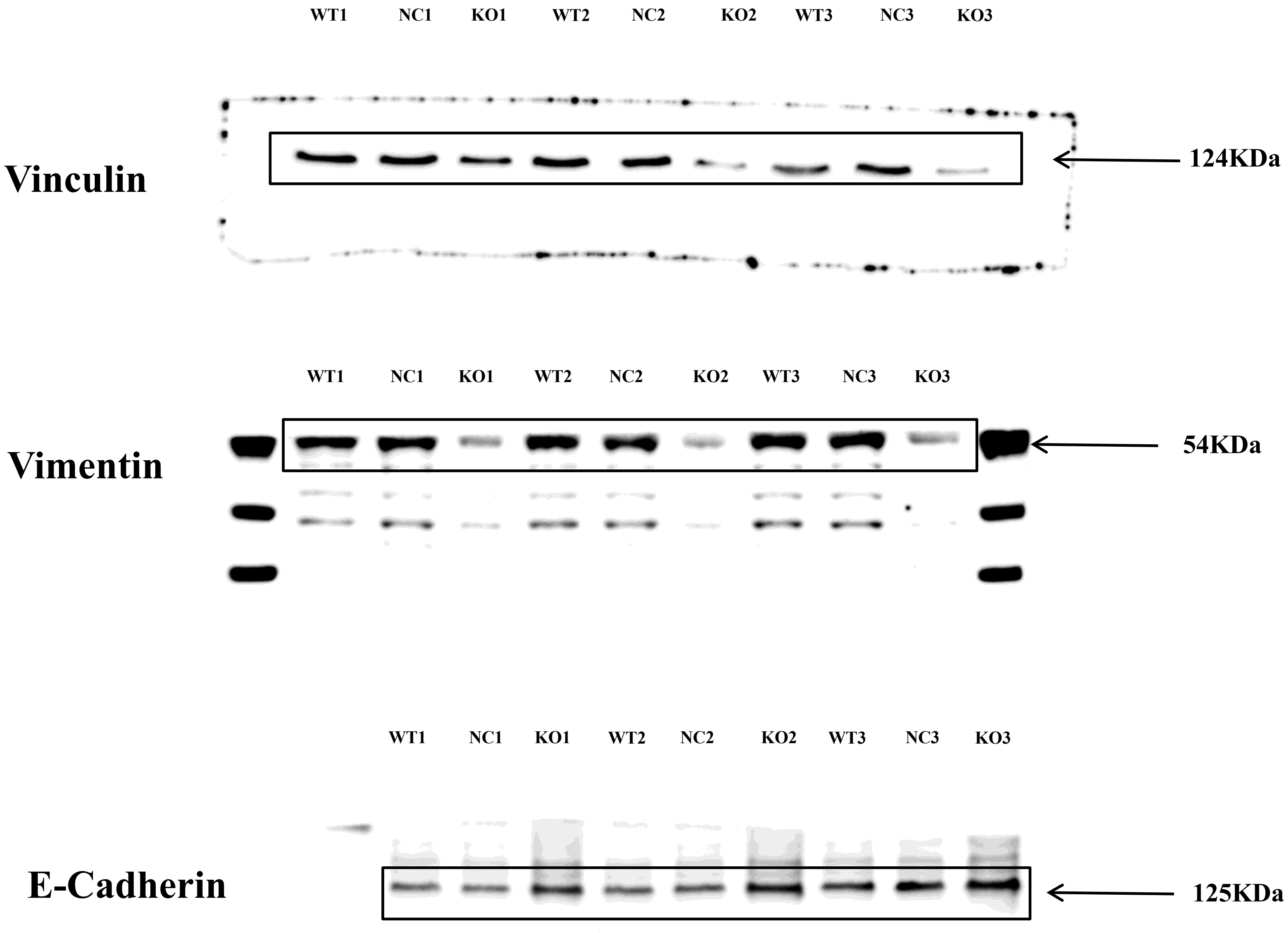

Figure S6 (KO-AKR1C2)

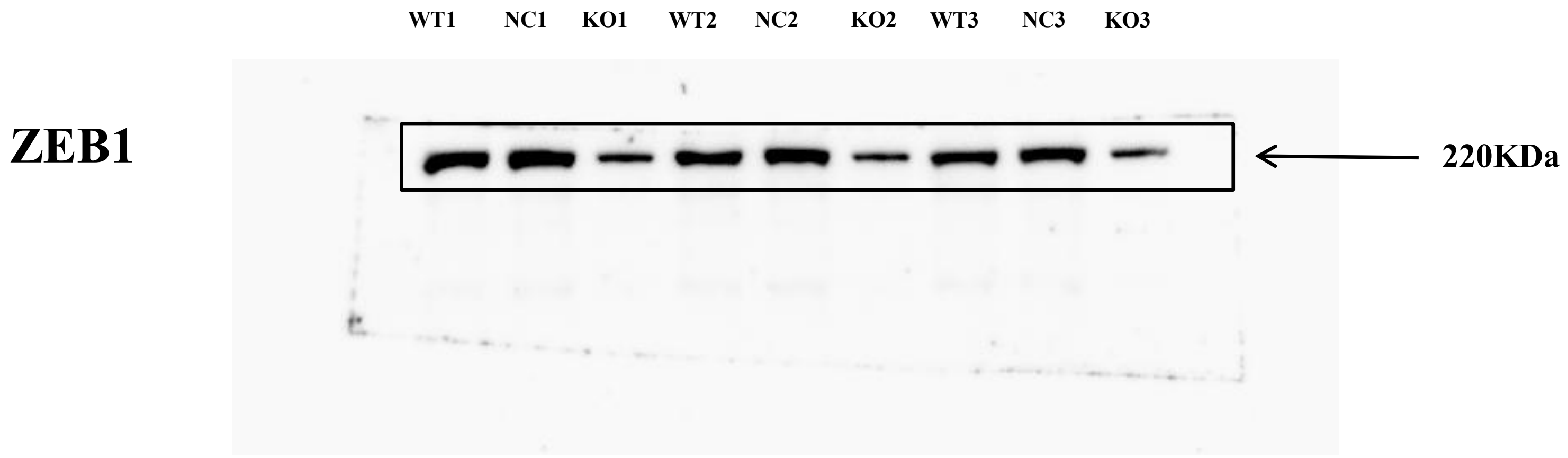

Figure S6 (OE-AKR1C2)

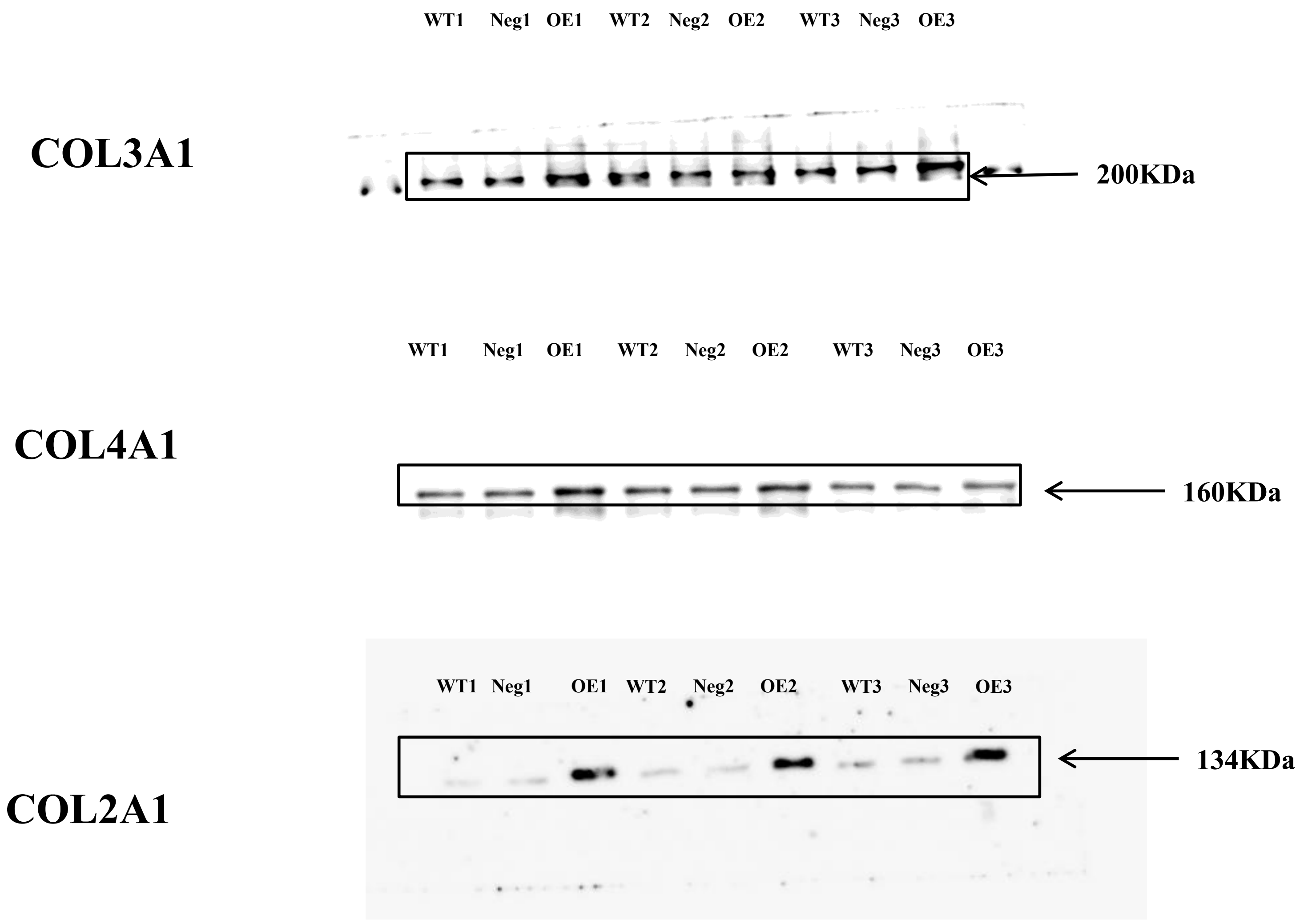

Figure S6 (OE-AKR1C2)

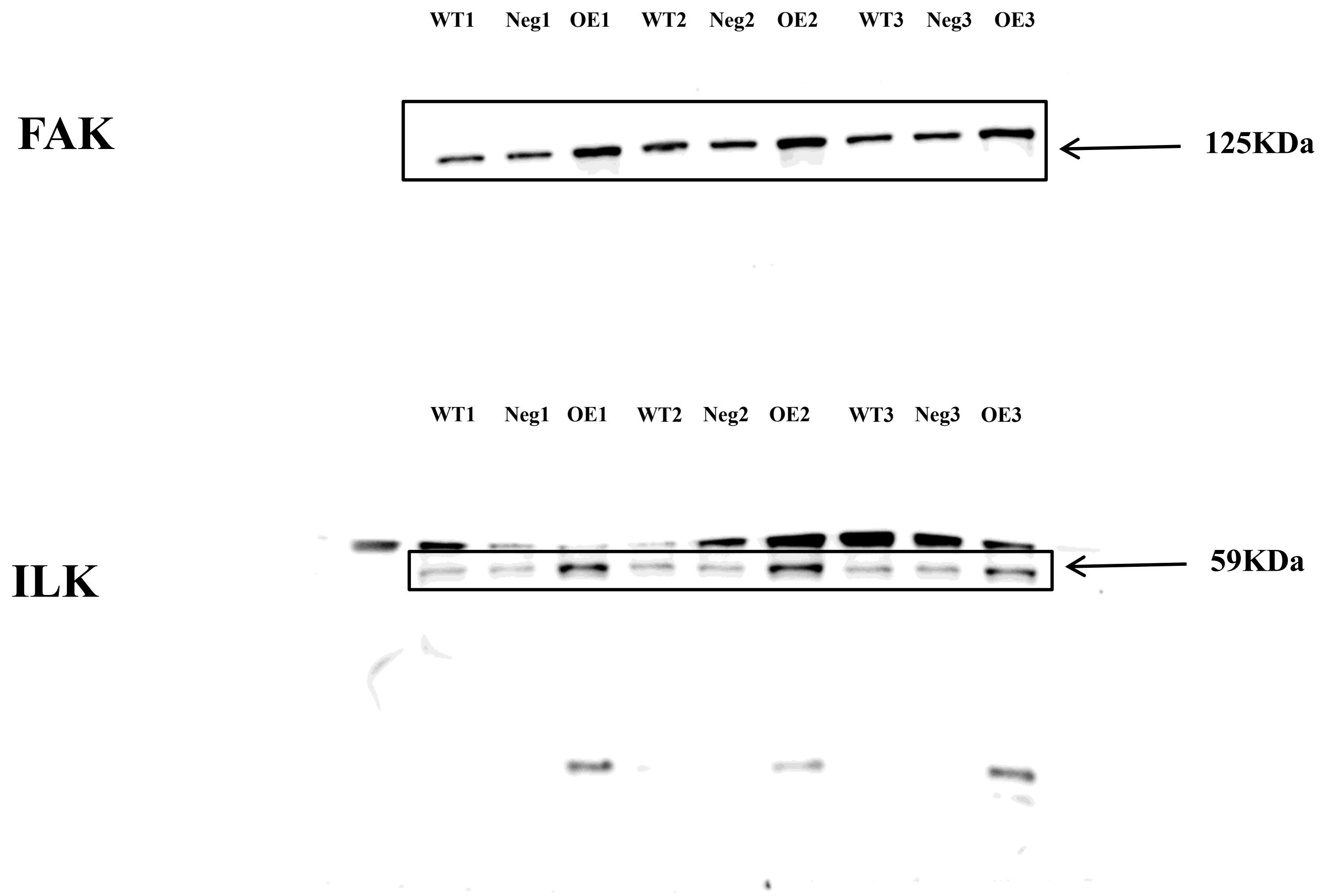

Figure S6 (OE-AKR1C2)

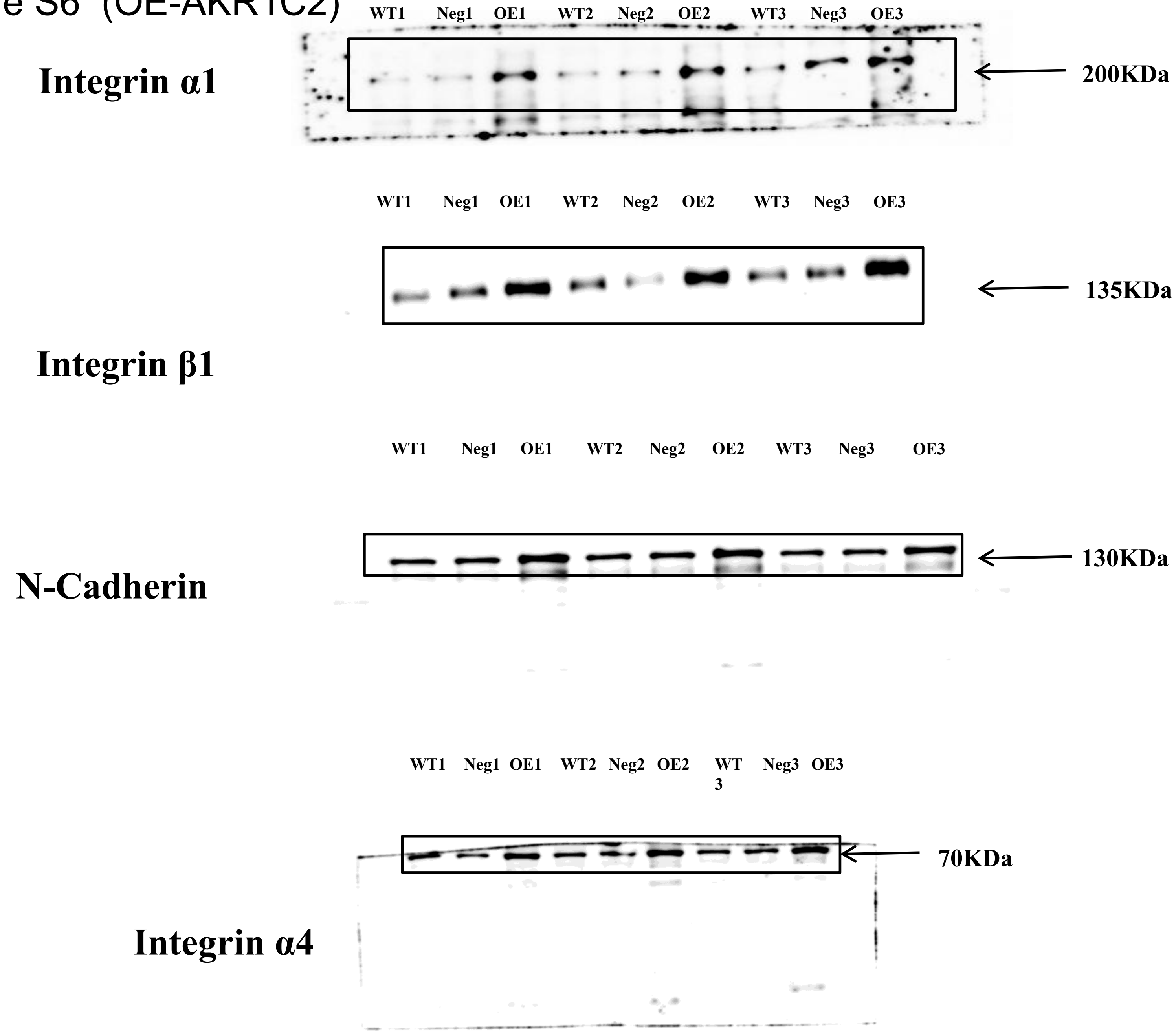

Figure S6 (OE-AKR1C2)

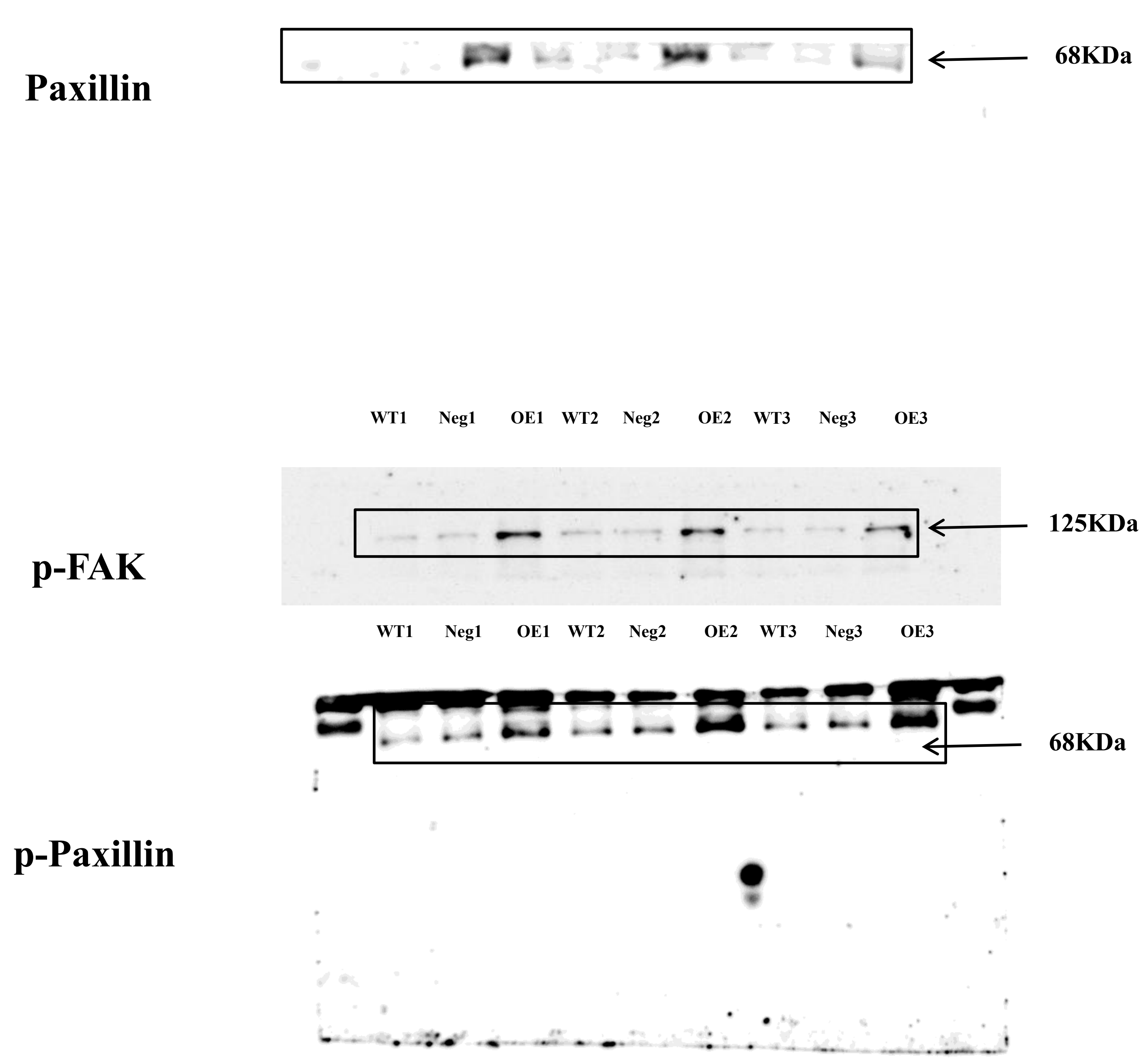

Figure S6 (OE-AKR1C2)

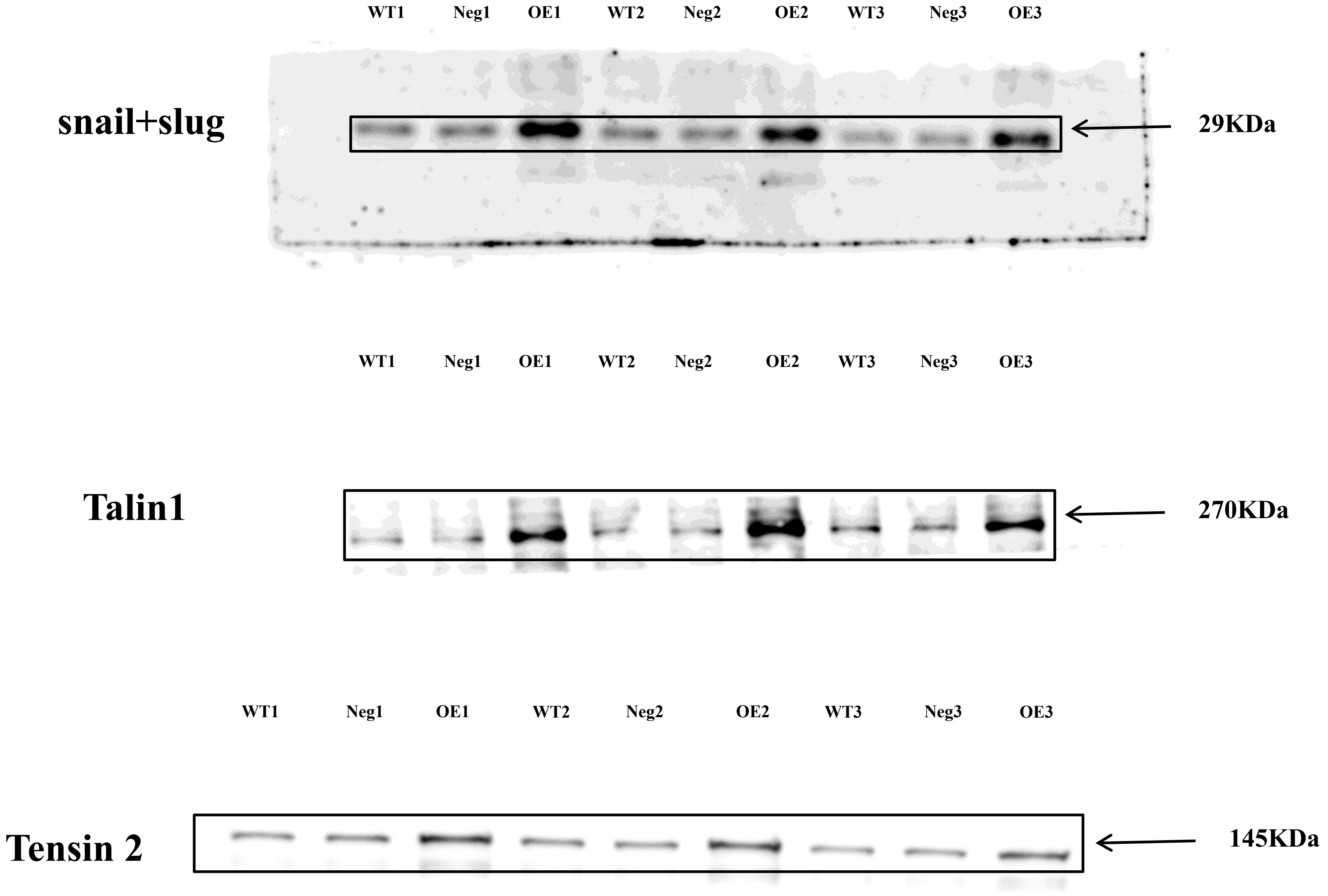

Figure S6 (OE-AKR1C2)

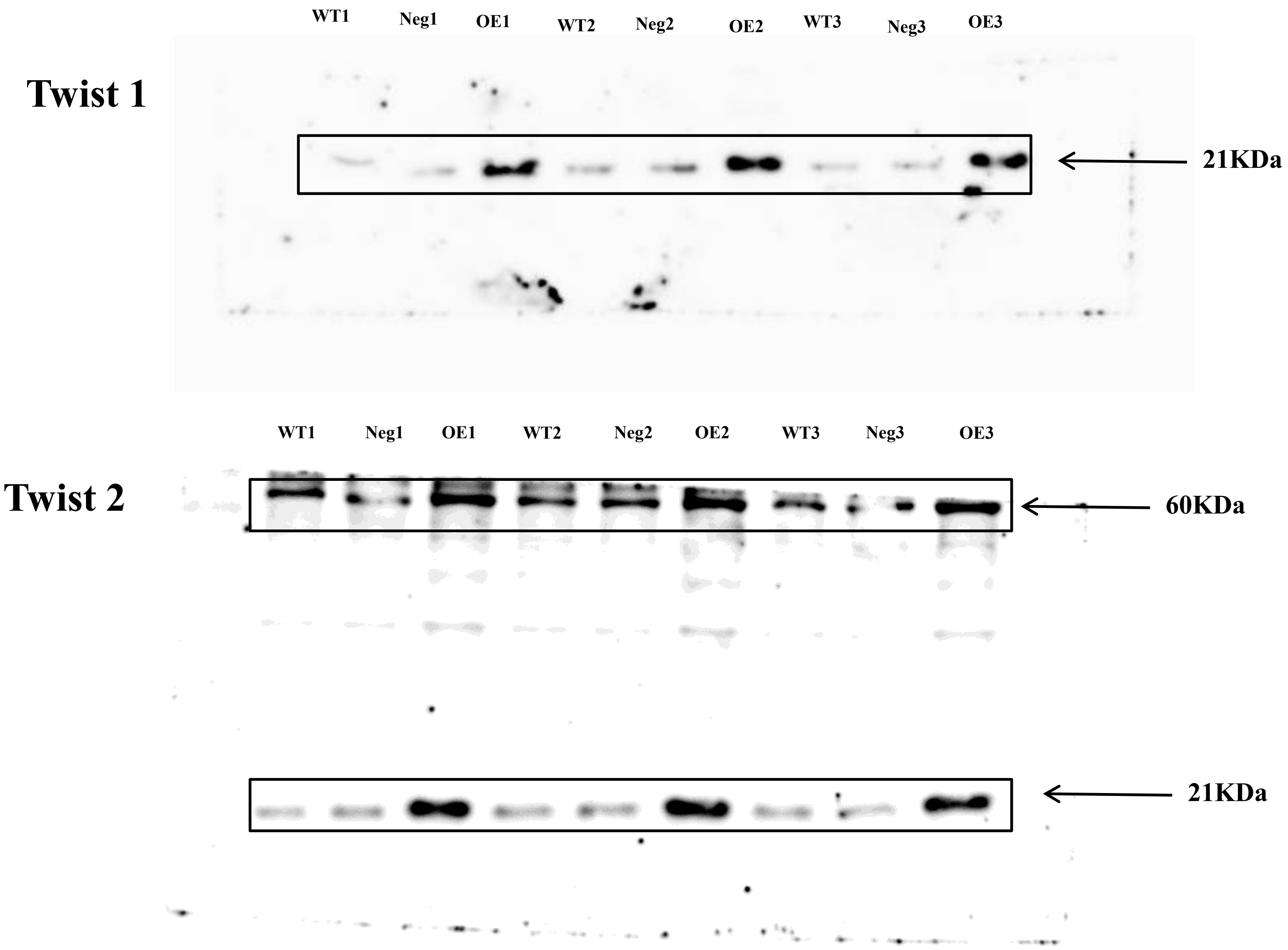

Figure S6 (OE-AKR1C2)

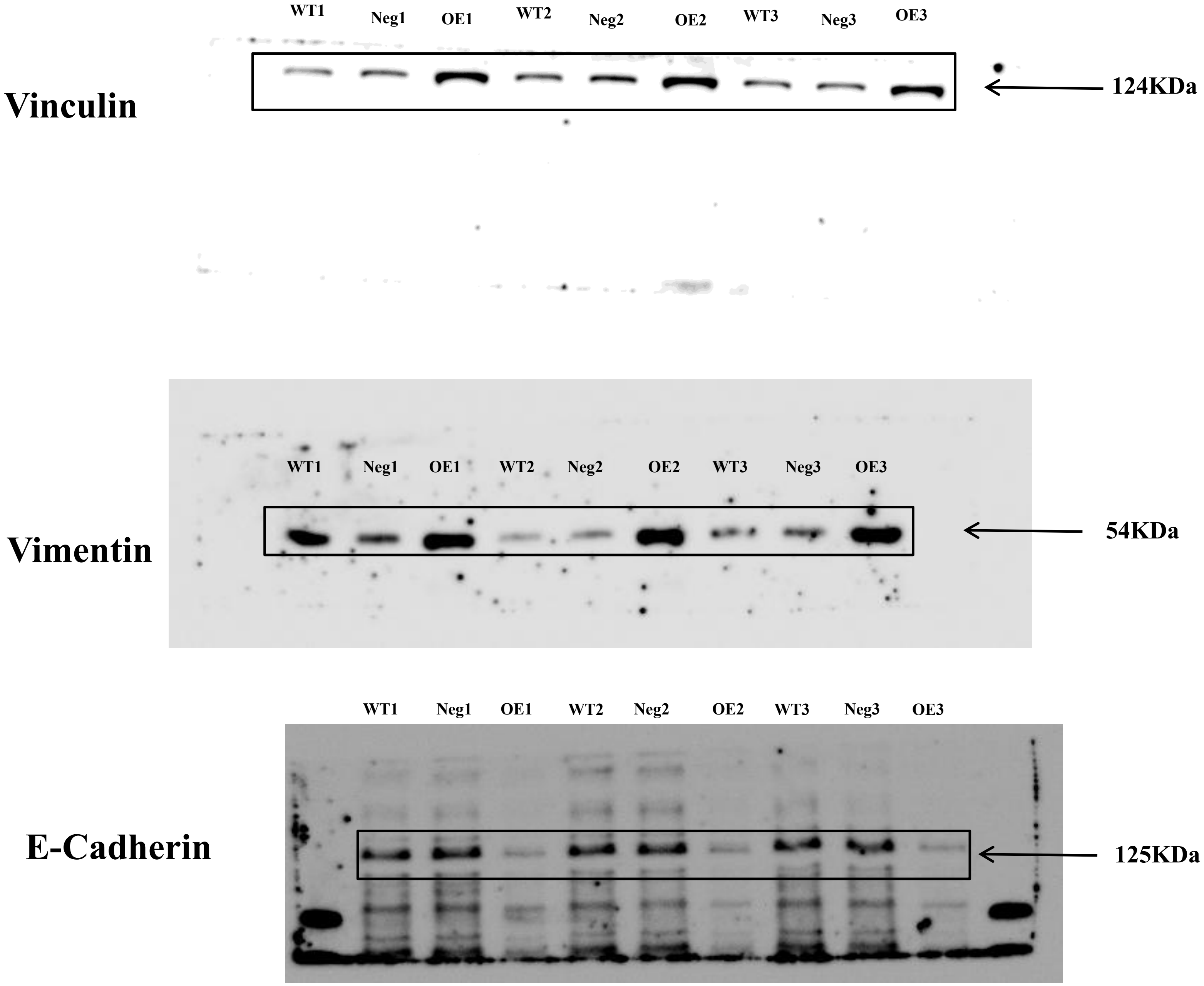

Figure S6 (OE-AKR1C2)

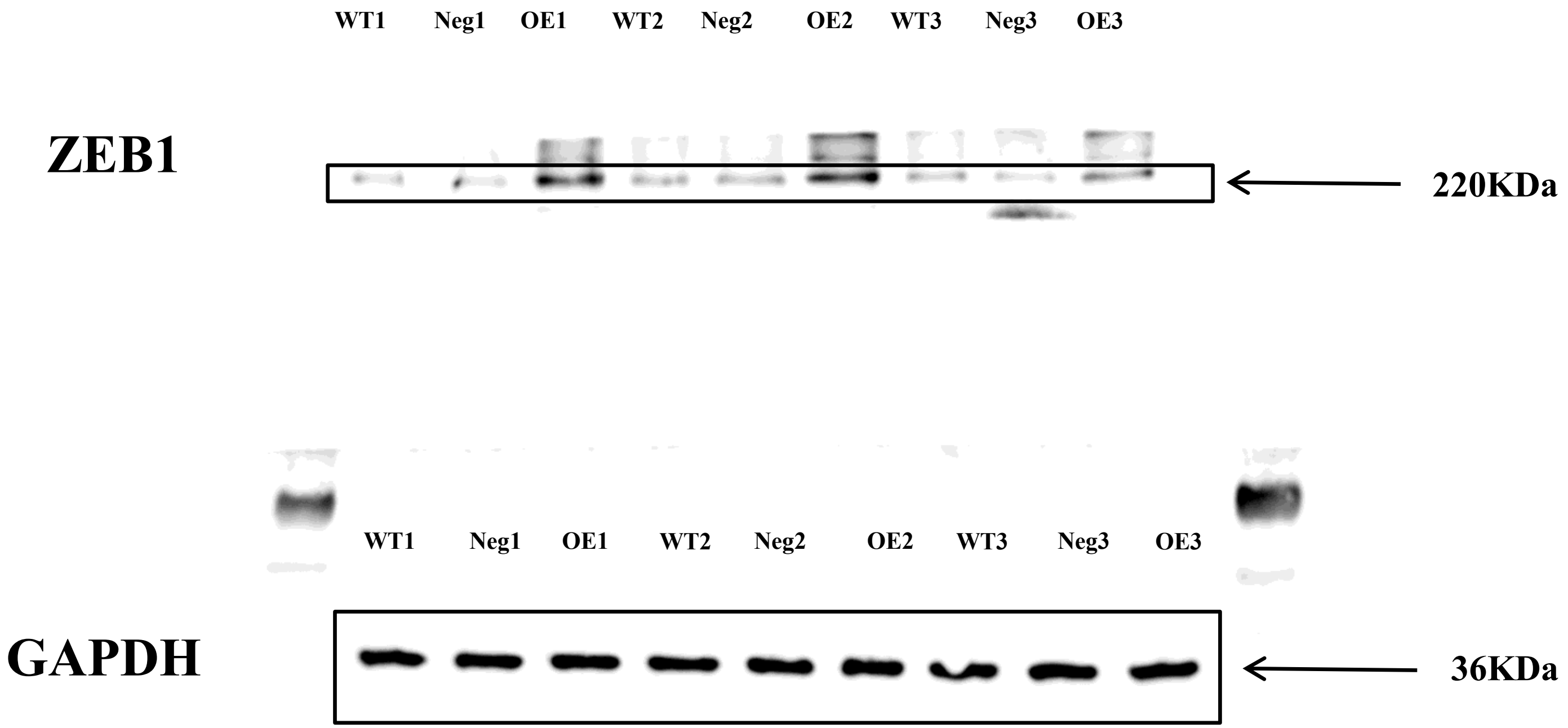

Supplementary Data

1.4 Construction of AKR1C2 stable overexpression cell lines

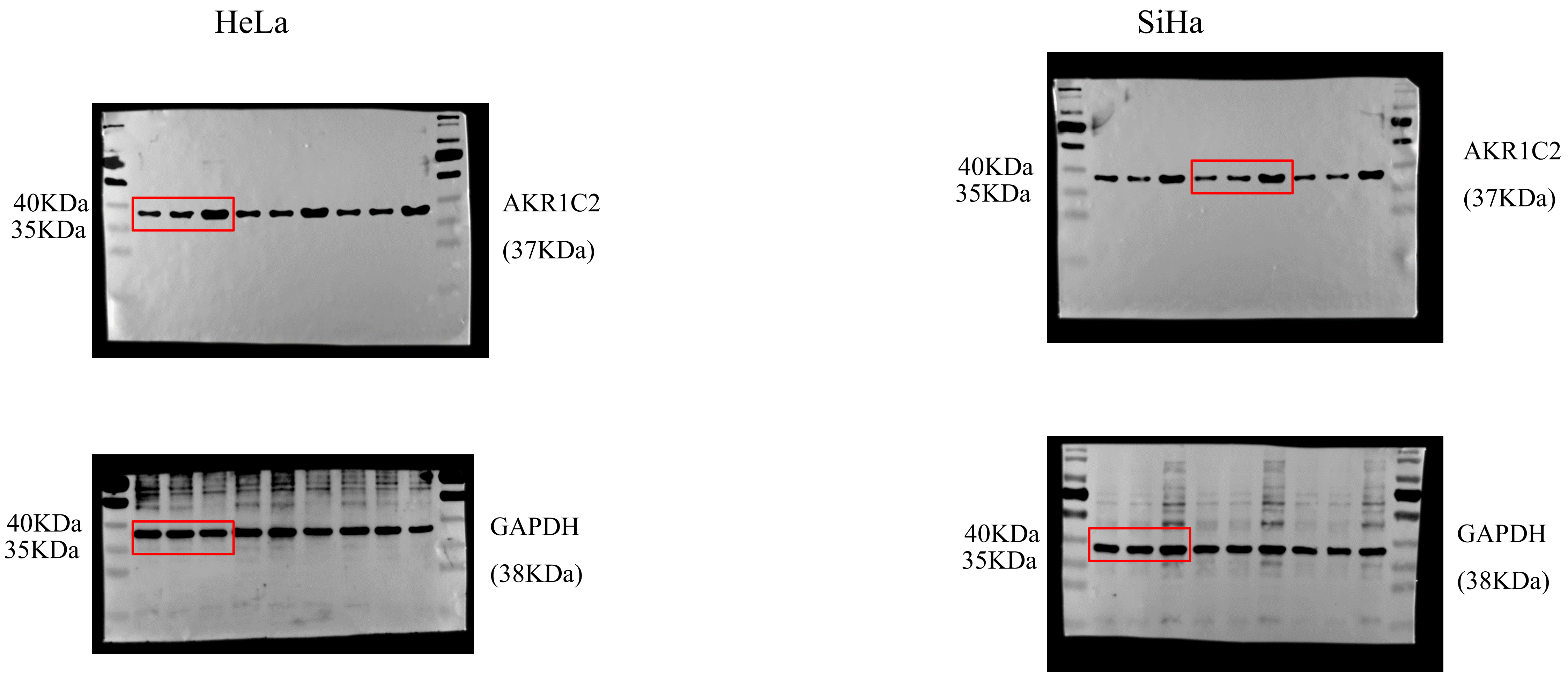

Supplementary Data

1.5 Construction of AKR1C2 gene knockout cell lines

HeLa

40KDa
35KDa

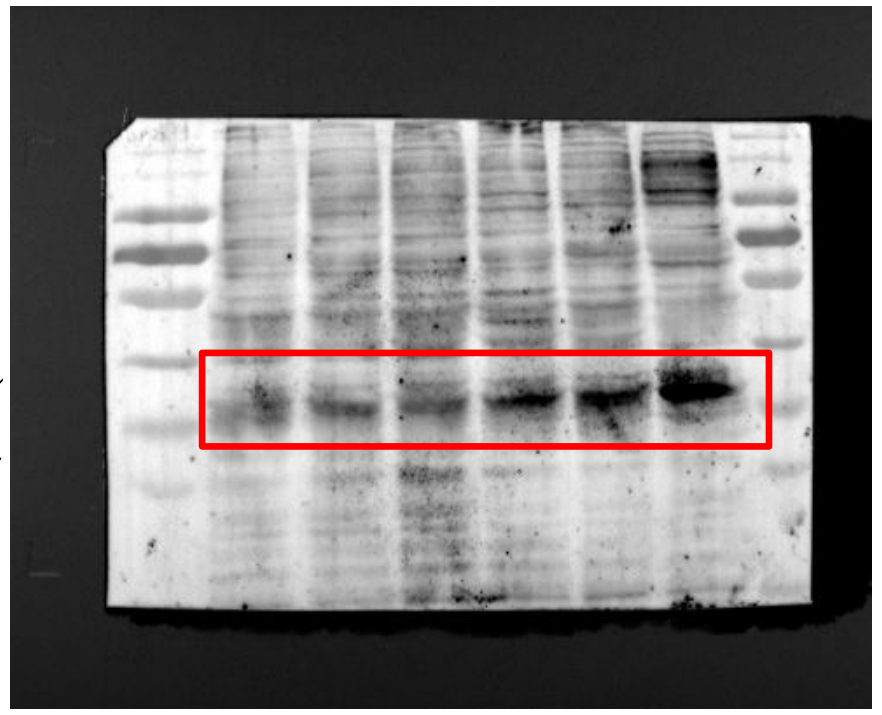

AKR1C2

(37KDa)

40KDa

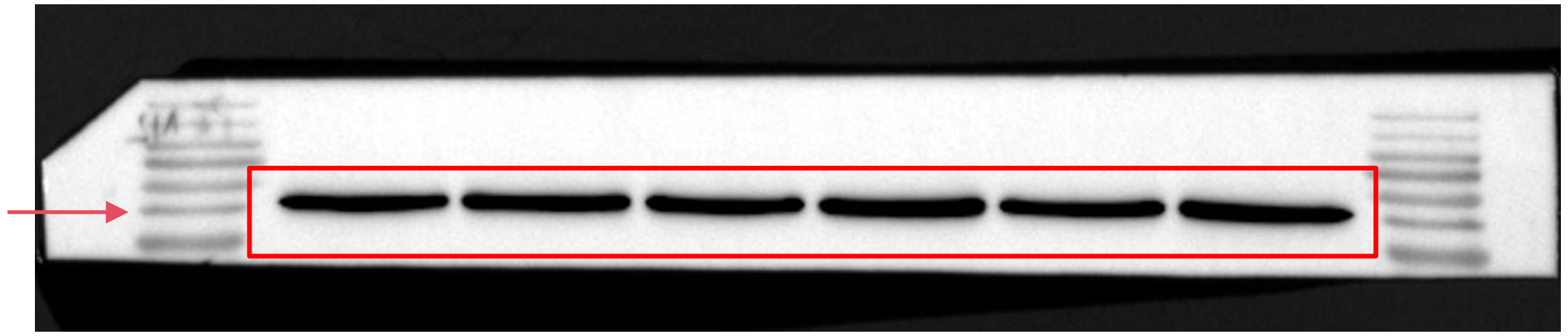

B-Actin

(42KDa)

SiHa

40KDa
35KDa

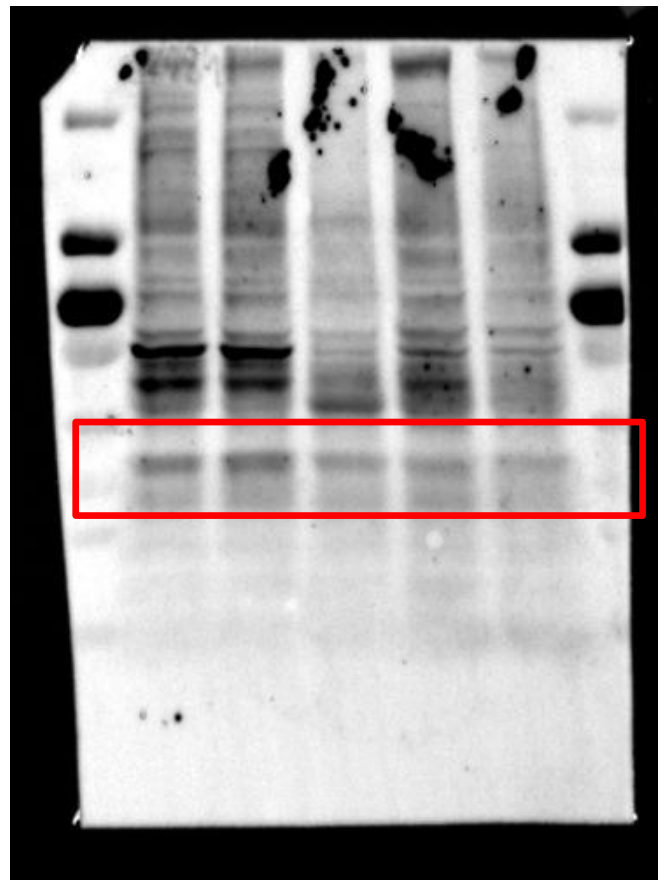

AKR1C2

(37KDa)

40KDa
35KDa

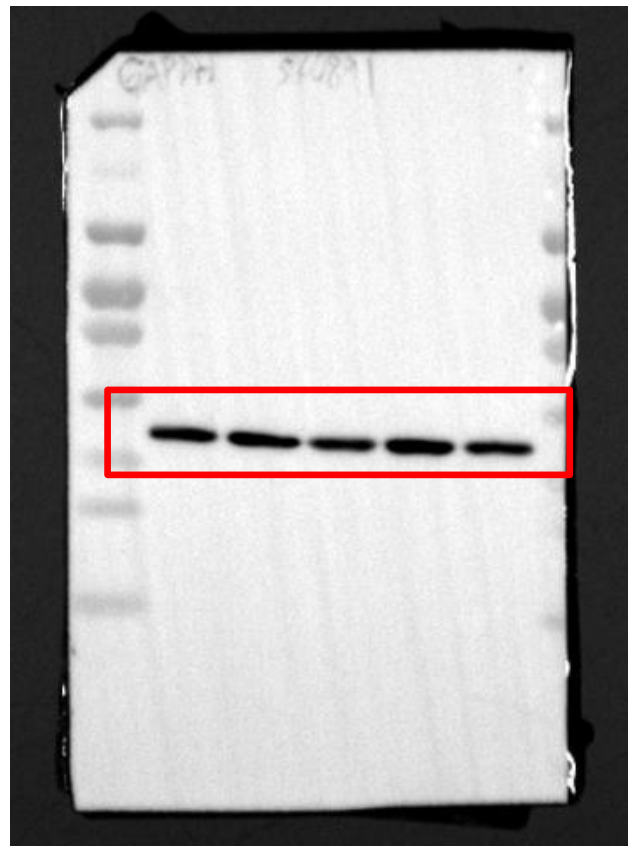

GAPDH

(38KDa)
