# Supplementary material for: AKR1C2 promotes lymph node metastasis in cervical cancer via a non-canonical nuclear function
Source: Cell Death Discov. 2026 Aug 13;12:450. doi: 10.1038/s41420-026-03287-5 (PMC13752304; doi:10.1038/s41420-026-03287-5)
Supplement: Supplementary file 2 [file 41420_2026_3287_MOESM1_ESM.docx]

Supplementary Data

## 1 Supplementary materials and methods

***1.1 Protein extraction from*** ***paraffin-embedded cervical tumors***

Protein extraction was performed on formalin-fixed paraffin-embedded cervical tissue. The sample was deparaffinized with xylene and then hydrated with graded ethanol. After hydration, the sample was washed twice with phosphate buffered saline (PBS). After removing the PBS solution, an appropriate amount of protein lysate (4% SDS, 100 mM Tris) was added and incubated at 95℃ for 10 minutes, mixed by shaking, and sonicated in an ice-water bath for 5 minutes. The samples were de-crosslinked by heating at 95℃ for 60 minutes, then reduced by adding an appropriate amount of TCEP and alkylated in CAA at 95℃ for 5minutes. The samples were sequentially centrifuged at 12000g at 4℃ for 15 minutes. The supernatant was collected, and 4x volume of pre-cooled acetone was added at -20℃ and precipitated at -20℃ for at least 4hours. Centrifuge at 12000g for 15 minutes at 4℃. Collect the precipitate and air dry. An appropriate amount of protein solution (8 M urea, 100 mM triethylammonium bicarbonate, pH = 8.5) was added to dissolve the protein pellet. Total Ion Chromatography (TIC) was used to evaluate sample quality control.

***1.2 DIA proteomics***

***Data-dependent acquisition (DDA)*** ***spectrum library construction***

Trypsin was added for protein digestion. The sample was fractionated using a C18 column (Waters BEH C18, 4.6×250 mm, 5 μm) on a Rigol L3000 HPLC system. LC-MS/MS analysis was then performed in DDA mode. For transition library construction, shotgun proteomics analyses were performed using an EASY-nLCTM 1200 UHPLC system (Thermo Fisher) coupled to a Q Exactive^TM^ HF-X mass spectrometer (Thermo Fisher) in DDA mode.

***LC-MS/MS Analysis-DIA mode***

Mobile phases A (0.1% FA in H2O) and B (0.1% FA in 80% ACN) were used to develop a gradient elution. A half sample containing 4 μg of fractional supernatant and 0.8 μl of indexed retention time (iRT) reagent was injected into an EASY-nLC^TM^ 1200 UHPLC system (Thermo Fisher) coupled to an Orbitrap Q Exactive^TM^ HF-X mass spectrometer (Thermo Fisher) operating in data-independent acquisition (DIA) mode with a spray voltage of 2.1 kV, Nanospray Flex^TM^ (ESI), and a capillary temperature of 320°C. For DIA acquisition, the m/z range covered 350 to 1500. The MS1 resolution was set to 60000 (at m/z 200), the full scan AGC target value was 5×10^5^, and the maximum ion injection time was 20 ms. Peptides were fragmented by HCD in MS2, where the resolution was set to 30000 (at 200 m/z), the AGC target was 1×10^6^, and the normalized collision energy was 27%. The raw data of the MS detection was named as “.raw”.

***DIA data analysis***

The resulting spectra from each fraction were searched separately by the search engines against the homo_sapiens_uniprot_2020_7_2.fasta database (192320 sequences): Proteome Discoverer 2.2 (PD 2.2, Thermo).

The result of the search and identification by the PD2.2 software was imported into the Spectronaut software (version 14.0, Biognosys) to generate a library. The iRT was added to the sample to correct the retention time, and the precursor ion Q value cutoff was set to 0.01. The protein quantitation results were statistically analyzed by T-test. Proteins with a fold-change > 1.2 (*p* < 0.05) were considered upregulated, and those with a fold-change < 0.83 (*p* < 0.05) were considered downregulated. These were defined as differentially expressed proteins (DEPs).

InterPro (IPR) functional analyses were performed using the interproscan program against the non-redundant protein database. DEPs were used for volcanic map analysis, cluster heat map analysis and enrichment analysis of IPR.

***1.3 IHC and predictive modeling***

Paraffin-embedded tissue blocks were sectioned to a thickness of 4 µm and mounted on polylysine-treated slides. Sections were then baked at 60°C for 2 hours, deparaffinized in two jars of xylene for 10 minutes each, and rehydrated through 100%, 95%, 85%, and 75% alcohol solutions for 5 minutes to deionized H_2_O. Sections for DEPs detection were pretreated in a Tris-EDTA solution (pH = 9.0) for 20 minutes in a microwave oven. The slides were then covered with 3% hydrogen peroxide for 10 minutes to block endogenous peroxidase activity. The slides were incubated with primary antibodies for 1 hour, followed by incubation with HRP-conjugated anti-rabbit secondary antibodies. In addition, 3,3-diaminobenzidine incubation was performed for 8 minutes to visualize immunoreactive proteins, and nuclei were counterstained with hematoxylin for 60 seconds. Appropriate positive and negative controls were also prepared to assess the quality control of this immunohistochemical staining protocol. Antibodies included rabbit polyclonal antibodies in details were shown below.

| Antibody | Company | Stata | Product No. | Dilution |
| --- | --- | --- | --- | --- |
| AKR1C2 | abcam | UK | ab96087 | 1:200 |
| GSS | abcam | UK | ab124811 | 1:150 |
| FBXO22 | abcam | UK | ab230395 | 1:600 |
| ARHGAP26 | abcam | UK | ab137085 | 1:200 |
| KRT78 | Sigma-Aldrich | USA | HPA039724 | 1:200 |
| ATP6AP2 | abcam | UK | ab64957 | 1:100 |

***1.4 Construction of AKR1C2 stable overexpression cell lines***

First, restriction endonucleases were used to digest the backbone vector to obtain a linearized vector. Subsequently, the target gene was synthesized, and the target gene fragment was amplified using primers with homologous recombination sequences. The digested vector was then ligated to the amplified gene fragment. The recombinant product was transformed, clones were selected for PCR identification, and positive clones were verified by enzyme digestion and sequencing. The correct clone was used for subsequent virus packaging.

The target gene fragment was constructed into a lentiviral vector. Vector map was designed as Overexpression group: LV-EF1A>Kozak-Human AKR1C2 CDS[NM_001393392.1]/T2A/Luciferase-CMV>EGFP/T2A/Puro; Control group: LV-EF1A>Kozak-Luciferase-CMV>EGFP/T2A/Puro. The lentiviral plasmids and helper plasmids (SL3, SL4, and SL5) were co-transfected into 293T cells. After transfection for 48 hours, the cell supernatant was collected by centrifugation for PEG concentration, ultracentrifugation, and quality testing.

HeLa and SiHa cells were seeded in 6-well plates, respectively. Lentivirus was added for transfection at an MOI of 30 for HeLa cells and 200 for SiHa cells. After transfection for 48 hours, cells were transferred to a 10 cm culture dish and cultured in fresh medium containing puromycin (1.5 μg/mL for HeLa, 2.0 μg/mL for SiHa). Cells were screened for 2-3 days and maintained with half-concentration puromycin. After amplification, RT-qPCR and Western blotting were used to identify the AKR1C2 protein abundance in stable overexpression HeLa & SiHa cell lines.

The efficiency of AKR1C2 knock-in was demonstrated in HeLa and SiHa cells by RT-qPCR and Western blotting, respectively.

***1.5 Construction of AKR1C2 gene knockout cell lines***

Genomic DNA was extracted from HeLa cells using a Simgen whole blood extraction kit. The designed primers were used to amplify the genomic sequence of the gene gRNA targeting site and its vicinity, and the above PCR products were electrophoretic with agarose gel, cut and sequenced. Cas9 protein and gRNA were incubated together, and 1 × 10^6^ cells were taken, centrifuged, and the supernatant was removed. The cell suspension was aspirated and transferred to an electroporation cuvette for electroporation. After electroporation, the cells were seeded into fresh culture medium. The genome of the obtained cells was extracted using Simgen whole blood extraction kit. The designed primers were used to amplify the human PML gene gRNA targeting site and its nearby genome sequence, and the above PCR products were subjected to agarose gel electrophoresis, cut and sequenced. Extreme dilution method was used for monoclonal plating and cultivation. The screening strategy of homozygous knockout clones is to design primers before and after gRNA amplification, and sequence the amplified products after agarose gel electrophoresis.

gRNA: TCAAATTGGCAATAGAAGCC-GGG

Primers for sequencing in Hela and SiHa cell lines

| **Cell lines** | **Primers** |
| --- | --- |
| HeLa | GGAGCTTACACTGCATTCCTTCAT |
| SiHa | GTTCCTGCTATGCCCAACCT |

AKR1C2 knock out in HeLa cell lines: TAAAAGTAAAGCTCTAGAGGCCGTCAAATTGGCAATAGAA--insert 4bp--AGAA—GCCGGGTTCCACCATATTGATTCTGCACATGTTTACAATA

TAAAAGTAAAGCTCTAGAGGCCGTCAAATTGGCAATAGAA--insert 1bp--A--GCCGGGTTCCACCATATTGATTCTGCACATGTTTACAATA

Control group in HeLa cell lines: CCTAAAAGTAAAGCTCTAGAGGCCGTCAAATTGGCAATAGAAGCCGGGTTCCACCATATTGATTCTGCACATG

Western blotting of HeLa cells showed as below.

The number of 1A6 and 1C1 was chosen as KO-AKR1C2 and KO-NC for furthermore experiment, respectively.

Sequencing results

AKR1C2 knock out in SiHa cell lines: TAAAAGTAAAGCTCTAGAGGCCGTCAAATTGGCAATAGAA--insert 1bp--A-- GCCGGGTTCCACCATATTGATTCTGCACATGTTTACAATA

Control group in SiHa cell lines: TTGCTCCCCCAGGTTCCTAAAAGTAAAGCTCTAGAGGCCGTCAAATTGGCAATAGAAGCCGGG TTCCACCATATTGATTCTGCACATGTTTACAATAATGAGG

Western blotting of SiHa cells showed as below.

The number of 1D4 and 1A1 was chosen as KO-AKR1C2 and KO-NC for furthermore experiment, respectively.

***1.6 Primers used for RT-qPCR in HeLa and SiHa cells***

| **Gene** | **Sense** | **Anti-sense** |
| --- | --- | --- |
| AKR1C2 | CCCTTCACCTTCCTTCATGATGT | GCATCCAGTTCAACCGTTTCTTA |
| GAPDH | GGAGCGAGATCCCTCCAAAAT | GGCTGTTGTCATACTTCTCATGG |
| ELFN2 | GCAGTTCCTCTTTGTCCAGC | CTCACAGTTGAAGGGGTTGC |
| SPOCK2 | CCAAGAAGAAGCCAGGCATC | TTCTCCTCCTCATCCTCCCA |
| SCX | GCTCTCCAAGATTGAGACGC | GCGGTCCTTGCTCAACTTTC |
| MGP | AGCCTGATCCTTCTTGCCATCC | TCCCTATTGAGCTCGTGGAC |
| SPINT2 | TGCCTCAAGAAATGTGCCAC | GTTGGCGGTGCAGTATTCTT |
| ADAMTS1 | CCTGGTGGTGGTGAAGATCT | GCTGTGTCATAGTGCTCTGC |
| WNT6 | AGAGTGCCAGTTCCAGTTCC | AGCTCGCCCATAGAACAGG |
| SERPING1 | ACTACCCCGCATCAAAGTGA | CACCCCAGTCTCTGTCAGTT |
| CCN3 | GCCTTACCCTTGCAGCTTAC | GCCGAGTCTGTTTCAGCATC |
| CCDC80 | AGACCTATGACCACCACTGC | CTGGGTTCTGAGATGGTGGT |
| PHOSPHO1 | CTATCTAGGGACGGCAGGAT | ACTCGTTGTAGAAGCCCTCG |
| EFEMP2 | GACTGCCATAACTTGCCTGG | CCCATGTCACACTCGTTCAC |
| GPLD1 | TGACGTGTCTAAGGTGGCAT | CCCCAATCAGTCCAGAGGTT |
| VIT | AGTGGAGGAGGCTTCAAGAC | GAGCGAGTAGAAGCCGTTTG |
| TLL1 | CGAACCCGTCTAAGCAAAGG | GGGGTCACAGAAACTCTCCA |
| ADAMTSL4 | GGAGACCCAGGAGATTCGAG | GTAGGGGACGGAATAGCCTC |
| OLFML2A | TCCAGAAGTCTCCAGCCAAG | TGAAGTTTTCCAGGTTGCGG |
| FN1 | TAGCTTTGTGGTCTCCTGGG | AAACTCTGCTCCCCATCCTC |
| LOXL4 | CAAGTCTGCGGATCACATGG | TGGAGCCATTGAGAGTGAGG |
| COL5A1 | GGACTATGACTACGTGCCCA | TTGGAGGAGTTGGAGGTGTC |
| FBN1 | CAATTTGGAGGCTGGGAACG | TCACACCTCTCTGCAGATCT |
| PDGFB | TTGTGCGGAAGAAGCCAATC | GAATGGTCACCCGAGTTTGG |
| LOX | GCACACACACAGGGATTGAG | GGTGAAATTGTGCAGCCTGA |
| LOXL2 | GGAGGATGCTGGTGTGAGAT | GTGCCAATACCAGGTCTCCT |
| APOE | ATGAAGGTTCTGTGGGCTGC | GGTAATCCCAAAAGCGACCC |
| CCN2 | TCTTCGGTGGTACGGTGTAC | TGTCTTCCAGTCGGTAAGCC |
| ETS1 | TCGATCTCAAGCCGACTCTC | CATTCACAGCCCACATCACC |
| TGFBI | CCTGAGAGACCTGCTGAACA | GTCTTGGCTGAGTCTGGGAT |
| CCN5 | TGTAAGCAGGACCCCAGTTT | ACTGTTTTGTGGACTGCGAC |
| NAV2 | GCTGCTTCCTTGTGGCTATC | CGTCCCTCTCGAAGCACTAT |
| COL7A1 | CCCAGTACCGCATCATTGTG | CGTTGCATCTGACACCACAA |
| ENTPD2 | GTCTACACCCACAGCTTCCT | ATGGTGCATGGTGACTGGTA |
| LTBP2 | CTACCCTGGCTACACTCTG | CCAGACAAGTGTAGGAGCCA |
| ITGB2 | ATGTGGATGAGAGCCGAGAG | ACTGGGACTTGAGCTTCTCC |
| H4C13 | GCCAAACGCAAGACAGTCAC | GGCCTTTGAGGAACCGTGTA |

***1.7*** ***Antibodies used for Western blotting in HeLa and SiHa cells***

| **Antibody** | **Company** | **Stata** | **Product No.** |
| --- | --- | --- | --- |
| COL2A1 | proteintech | USA | 28459-1-AP |
| COL3A1 | abclonal | China | A3795 |
| COL4A1 | abclonal | China | A10710 |
| E-Cadherin | proteintech | USA | 20874-1-AP |
| FAK | CST | USA | 71433T |
| P-FAK (Tyr397) | CST | USA | 8556T |
| ILK | proteintech | USA | 12955-1-AP |
| Integrin α1 | CST | USA | 15574T |
| Integrin α4 | CST | USA | 8440T |
| Integrin β1 | CST | USA | 34971T |
| N-Cadherin | abcam | UK | ab76011 |
| Paxillin | proteintech | USA | 10029-1-Ig |
| P-Paxillin (Tyr118) | CST | USA | 69363S |
| SNAIL+SLUG | abcam | UK | ab180714 |
| Talin-1 | CST | USA | 4021T |
| Tensin 2 | CST | USA | 11990S |
| Twist | abclonal | China | A7314 |
| Twist2 | proteintech | USA | 11752-1-AP |
| Vimentin | abcam | UK | ab92547 |
| Vinculin | CST | USA | 4650T |
| ZEB1 | abcam | UK | ab203829 |

***1.8 Function studies***

***Cell counting kit-8 (CCK-8) assays***

The cells (non-treated cells, transfected cells) in the logarithmic growth phase were collected, and the number of cells was adjusted to 5×10^4^ cells/ml and seeded into 96-well plates (100μl/well), with 6 replicates per group. The wells without cells were used as blank control. The cells were incubated for 80 h. WST-8 (Cell Titer 96 Aqueous One Solution Cell Proliferation Assay solution) and DMEM were mixed at a ratio of 1:10, then 200μl of the mixture was added to each well and incubated for 2 h. Their absorbance value was detected at 490 nm using a microplate reader (Bio-Tek EPOCH2, VT, USA).

***Cell adhesion assays***

A 96-well plate was coated with 10ug/ml fibronectin, collagen, laminin and fibrinogen, respectively, at room temperature for 1 hour. The coating solution was removed and 200ul of thermally denatured 1% BSA (prepared with BSA at high temperature and pressure) was added and the well plate was incubated at 37°C for 1 hour. Serum-free culture medium was used to submerge the well plate twice. After the cells were digested with trypsin, the digestion was stopped with serum-containing culture medium, and then the cells were washed with serum-free culture medium and resuspended. After cell counting, 5×10^4^ cells were seeded into a pretreated 96-well plate. The cells were incubated in a cell incubator for 12 hours. PBS was used for washing 3 times. The CCK-8 method was used to detect the OD value in the 96-well plate and the cell adhesion rate was calculated.

***Wound-healing assays***

A wound-healing assay was performed in cells transiently transfected with siRNA or overexpression plasmid. Cells were seeded in 6-well plates (2x10^6^ cells per well) and when grown to full confluent density, a crossing scrape was performed in each well using a sterile 20μl pipette tip. Detached cells were washed away with PBS. Crossing scrape images were captured using an inverted microscope (OLYMPUS IX73, Tokyo, Japan) at 0 hours and 48 hours in the same fields.

***Transwell invasion assays***

Transwell invasion assays were performed using chambers (Corning, Michigan, USA) according to the manufacturer’s instructions. The upper chamber was seeded with 2x10^6^ cells in serum-free DMEM. The bottom chamber contained DMEM with 10% FBS. After incubation for 48 hours, fixed with methanol for 15 minutes and stained with 0.1% crystal violet for 30 minutes at room temperature, the cells on the upper surface of the membranes were removed with a cotton swab. The bottom surface of the membranes was observed and collected under a microscope (NIKON ECLIPSE 80i, Tokyo, Japan) at x100 magnification in four randomly selected fields per well for any migrated cells.

***Apoptosis assays***

The cells were collected at the logarithmic growth phase, and the number of cells was adjusted to 1 × 10^6^/m. The cells were divided into different treatment groups (si-RNA interference group, overexpression group, normal group) and cultured in the cell incubator for 48 h. Cells in each group were collected by trypsin digestion without EDTA, washed twice with pre-cooled PBS, and then binding buffer was added to gently blow into a single cell suspension. After mixing with 5μl Annexin V-FITC, 5μl propidium iodide was added; placed at room temperature, dark reaction for 10 minutes; then flow cytometry (BD, LSR-Fortessa) was used to detect and calculate the percentage of apoptosis (including early and late).

***1.9 RNA sequencing***

Total RNA was quantified using the Bioanalyzer 2100 system (Agilent). mRNA was isolated via poly-T magnetic beads, fragmented, and reverse-transcribed into cDNA. Libraries were constructed using Illumina-compatible adaptors, size-selected (370–420 bp), and amplified with Phusion polymerase. Sequencing was performed on an Illumina Novaseq platform, generating 150 bp paired-end reads (Novogene).

Raw reads were filtered to remove adapters, poly-N sequences, and low-quality reads (Q20/Q30 and GC content assessed). Clean reads were aligned to the reference genome using HISAT2 (v2.0.5) for splice-aware mapping. Transcript assembly and quantification were performed via StringTie (v1.3.3b). Gene expression levels were calculated as FPKM using feature Counts (v1.5.0-p3).

Differentially expressed genes (DEGs) were identified using DESeq2 (with biological replicates; adjusted *p* ≤ 0.05) or edge R (without replicates; |fold change| ≥ 2, *p* ≤ 0.05). Enriched GO terms were analyzed with cluster Profiler (corrected *p* < 0.05). The cluster Profiler R package was used to test the statistical enrichment of differential expression genes in KEGG pathways.

***1.10 Immunofluorescence (IF) assays***

Cells were seeded in a 24-well plate. After 48 hours of adherence, the cells were fixed with 4% paraformaldehyde for 15 minutes at room temperature, followed by permeabilization with 0.1% Triton X-100 for 15 minutes. The cells were then incubated with the primary antibody overnight at 4°C. The following day, a FITC-conjugated secondary antibody was applied and incubated at 37°C for 60 minutes. Finally, the nuclei were stained with DAPI for 15 minutes in the dark. Images were acquired using a fluorescence microscope.

***1.11*** ***Antibodies used for Co-IP***

| **Antibody** | **Company** | **Stata** | **Product No.** |
| --- | --- | --- | --- |
| PIP | Cloud-Clone-Crop | China | PAM035Hu01 |
| KRT9 | Abmart | China | TN26662S |
| KRT2 | Cloud-Clone-Crop | China | PAA491Hu01 |
| SOD1 | Immunoway | China | YM8065 |
| NQO1 | Abmart | China | T56710S |
| GCLM | Immunoway | China | YM8359 |
| ALDH3A1 | Proteintech | USA | 15578-1-AP |

***1.12 Construction of AKR1C2 truncation mutants and domain mapping***

To map the H4C13-interacting domain of AKR1C2, three truncated mutants were generated based on the full-length AKR1C2 coding sequence (NM_001393392.1). The truncation fragments were designed to cover three equal segments of the 323-amino-acid protein: Fragment 1 (amino acids 1–107), Fragment 2 (amino acids 108–215), and Fragment 3 (amino acids 216–323). Each fragment was amplified by PCR and cloned into a HA-tagged lentiviral vector (LV-EF1A>HA/IRES/Puro) following the same procedure described in Supplementary Materials and Methods 1.4, yielding three constructs: LV-EF1A>AKR1C2-1-107-HA/IRES/Puro, LV-EF1A>AKR1C2-108-215-HA/IRES/Puro, and LV-EF1A>AKR1C2-216-323-HA/IRES/Puro. The full-length AKR1C2 construct (LV-EF1A>AKR1C2-HA/IRES/Puro) was generated as a control. All constructs were verified by DNA sequencing. HeLa and SiHa cells were transfected with the truncation plasmids using Lipofectamine 2000 (Invitrogen) according to the manufacturer's instructions, as described in the Cell transfection section of Methods.

Nuclear and cytoplasmic fractions were extracted using a Nuclear and Cytoplasmic Protein Extraction Kit (Beyotime, No. P0027) following the manufacturer's protocol. Briefly, transfected HeLa or SiHa cells were harvested, resuspended in cytoplasmic extraction buffer, and incubated on ice for 15 minutes. After centrifugation at 12,000 × g for 5 minutes, the supernatant was collected as the cytoplasmic fraction. The nuclear pellet was then resuspended in nuclear extraction buffer, incubated on ice for 30 minutes with vortexing every 10 minutes, and centrifuged at 12,000 × g for 10 minutes to obtain the nuclear fraction. The distribution of AKR1C2 truncation mutants was assessed by Western blotting using anti-HA antibody (Proteintech, No. 66006-2-Ig). β-actin (Proteintech, No. 66009-1-Ig) and Lamin B1 (Proteintech, No. 12987-1-AP) were used as cytoplasmic and nuclear loading controls, respectively.

Co-immunoprecipitation assays for truncation mutants were performed as described in the Co-IP section of Methods. Briefly, HeLa or SiHa cells were transfected with HA-tagged AKR1C2 full-length or truncation constructs. After 48 hours, cells were lysed in IP lysis buffer. Cell lysates were incubated with anti-HA antibody (Proteintech, No. 66006-2-Ig) and Protein A/G magnetic beads (Thermo Scientific™, No. 88803) overnight at 4°C with rotation. The beads were washed three times with lysis buffer, and bound proteins were eluted by boiling in SDS loading buffer. Immunoprecipitates were analyzed by Western blotting with anti-H4C13 antibody (Immunoway, No. YM8088) to detect endogenous H4C13, and with anti-HA antibody to confirm the expression of AKR1C2 truncation mutants.

***1.13 Tumor-implanted zebrafish model***

A visual zebrafish model of lymphatic vessels (*Tg[lyve1b:DsRed]* line) and a wild-type AB line were purchased from *Hunter Biotech Company (https://www.zhunter.com/)*. We testcrossed *Tg(lyve1b:DsRed)* with the wild-type AB line to obtain heterozygous embryos of the *Tg(lyve1b:DsRed)* line. Embryos at 48 hours post-fertilization were randomly selected from the same clutch and allocated to experimental groups (at least 10 zebrafish per group). For primary cancer models, approximately 150-200 tumor cells (HeLa and SiHa) were labeled in vitro with 10 μM DiO (Invitrogen, inc) and injected into the perivitelline space of each embryo using an Eppendorf microinjector. After injection, embryos were assessed for successful transplantation and eligible embryos were retained for further experiments. Embryos were detected by fluorescence microscopy to visualize metastatic foci in the trunk and tail region of each embryo. Zebrafish embryos were transferred to 24-well plates (1 embryo/well) and incubated at 33°C for another 24 h. Tumor cell metastasis was monitored by fluorescence microscopy (Nikon Eclipse C1). The viability of tumor-implanted zebrafish was determined by assessing heartbeat, blood circulation and physical movement. For the lymphatic vessel tumor cell metastasis model, we measured and counted DsRed and DiO (green) positive areas in the trunk and tail regions of zebrafish using ImageJ software.

***1.14 Orthotopic xenograft model of NOD/SCID mice***

Subcutaneous tumors were established by injecting 5×10^6^ tumor cells, either AKR1C2-overexpressing or control HeLa cells, suspended in 100μL of culture medium into the dorsal flanks of female NOD/SCID mice. These subcutaneous tumors were then excised and dissected into approximately 2 mm³ fragments in DMEM. These fragments served as donor tissue for orthotopic implantation into the uterine cervix of recipient NOD/SCID mice.

For transplantation, recipient mice were anesthetized by inhalation of isoflurane (2-3% in oxygen). A small midline incision was made to expose the cervix, and a single 2 mm³ tumor fragment was sutured onto the cervical surface with 9-0 silk suture. The abdominal wall and skin were then closed in layers. On days 7, 21, and 35 post-transplantation, mice from each cell line group were injected with D-fluorescein, anesthetized, and subjected to *in vivo* imaging to determine cervical tumor and lymph node metastasis (Clinx IVScopeEQ Capture, Shanghai, China).

In addition to bioluminescence imaging, the animals underwent daily monitoring for indications of distress, such as changes in body weight, activity levels, and grooming behavior. Humane endpoints were predefined as: (1) a reduction in body weight exceeding 20% of the original weight; (2) sustained signs of pain or distress, including hunched posture or lethargy; (3) tumor ulceration or infection when externally visible. None of the mice reached these endpoints prior to the scheduled conclusion, and all were euthanized at the predetermined endpoint on day 35.

At the terminal time point (day 35 post-transplantation), mice were euthanized via carbon dioxide (CO₂) inhalation followed by cervical dislocation, in accordance with the AVMA Guidelines for the Euthanasia of Animals. The primary tumor volume was calculated using the formula: volume = (length × width²) / 2, and body weight was recorded. Primary tumor lesions, as well as pelvic and abdominal lymph nodes, were harvested for pathological examination using H&E staining to detect metastasis.

## 2 Supplementary Figures

**Figure S1** Flowchart of the training set with 101 patients’ selection.

**Figure S2.** MS-based analysis of ECM-related proteins in LNM vs NLNM. (A) Heatmap of ECM-related DEPs. Expression patterns of TNXB, KRT3, KRT17, and KRT78 across 18 LNM and 18 NLNM samples. Red: high abundance; blue: low abundance. TNXB was upregulated, while KRT3, KRT17, and KRT78 were downregulated in LNM samples. Detailed protein information is provided in Supplementary file. (B) Spearman correlation between AKR1C2 and ECM-related DEPs. Bar plot showing correlation coefficients (r) between AKR1C2 and the four ECM-related DEPs. Positive correlations: red; negative correlations: blue. AKR1C2 showed a positive correlation trend with KRT78 (r = 0.304, p = 0.072), while correlations with TNXB (r = -0.174, p = 0.310), KRT17 (r = -0.195, p = 0.253), and KRT3 (r = -0.058, p = 0.739) were weaker.

**Figure S3** Calibration curve. (A)-(D) Calibration curve for AKR1C2, ARHGAP26, ATP6AP2, Model b in the training set, respectively. (E)-(H) Calibration curve for AKR1C2, ARHGAP26, ATP6AP2, Model b in the validation set, respectively.

**Figure S4** Kaplan-Meier curves for 5-year DFS and OS based on IRS. (A)-(B) DFS and OS for AKR1C2 in the training set. (C)-(D) DFS and OS for ARHGAP26 in the training set. (E)-(F) DFS and OS for ATP6AP2 in the training set. (G)-(H) DFS and OS for AKR1C2 in the validation set. (I)-(J) DFS and OS for ARHGAP26 in the validation set. (K)-(L) DFS and OS for ATP6AP2 in the validation set.

**Figure S5** AKR1C2 regulated the proliferation and apoptosis of cervical cancer cells in vitro. (A) and (B) Bar plots showing the relative adhesion ability of cells with different AKR1C2 expression cultured on plates precoated with different extracellular matrix components. (C) and (D) CCK-8 assays for HeLa and SiHa cells to determine the effect of AKR1C2 expression on cell proliferation. (E) and (F) Apoptosis assays for HeLa and SiHa cells to determine the effect of AKR1C2 expression on cell apoptosis. Bar plots counting the cell apoptosis ratio were shown on the right of representative bright field images. *p* values in all bar plots were calculated by non-parametric t-test. *p* values: **p* < 0.05, ***p* < 0.01, ****p* < 0.001, ns, not significantly different.

**Figure S6** AKR1C2 regulates the expression of extracellular matrix- and EMT-associated proteins in HeLa cells. (A-B) Western blotting showing the expression of ECM-related proteins (COL2A1, COL3A1, COL4A1), adhesion complex proteins (FAK, p-FAK, Paxillin, p-Paxillin, Talin-1, Vinculin, Tensin 2), integrins (α1, α4, β1), EMT markers (E-cadherin, N-cadherin, Vimentin), and EMT transcription factors (SNAIL+SLUG, Twist, Twist2, ZEB1) upon AKR1C2 knockout and overexpression. GAPDH served as a loading control. Bar plots show the relative protein abundance levels normalized to GAPDH. *p* values in all bar plots were calculated by one-way ANOVA test. *p* values: **p* < 0.05, ***p* < 0.01, ****p* < 0.001, *****p* < 0.0001, ns, not significantly different.

**Figure S7** AKR1C2 regulates the expression of extracellular matrix- and EMT-associated proteins in SiHa cells. (A-B) Western blotting showing the expression of ECM-related proteins (COL2A1, COL3A1, COL4A1), adhesion complex proteins (FAK, p-FAK, Paxillin, p-Paxillin, Talin-1, Vinculin, Tensin 2, ILK), integrins (α1, α4, β1), EMT markers (E-cadherin, N-cadherin, Vimentin), and EMT transcription factors (SNAIL+SLUG, Twist, Twist2, ZEB1) upon AKR1C2 knockout and overexpression. GAPDH served as a loading control. Bar plots show the relative protein abundance levels normalized to GAPDH. *p* values in all bar plots were calculated by one-way ANOVA test. *p* values: **p* < 0.05, ***p* < 0.01, ****p* < 0.001, *****p* < 0.0001, ns, not significantly different.

**Figure S8** The mRNA abundance of other 33 genes from GO enrichment pathways regulated by AKR1C2 in HeLa cells. *p* values in all bar plots were calculated by one-way ANOVA test. *p* values: **p* < 0.05, ***p* < 0.01, ****p* < 0.001, *****p* < 0.0001, ns, not significantly different.

**Figure S9** The mRNA abundance of other 33 genes from GO enrichment pathways regulated by AKR1C2 in SiHa cells. *p* values in all bar plots were calculated by one-way ANOVA test. *p* values: **p* < 0.05, ***p* < 0.01, ****p* < 0.001, *****p* < 0.0001, ns, not significantly different.

**Figure S10** The relationship between mRNA abundance of 34 genes and DFS from GEPIA database. ELFN2 is unavailable due to no records in the database.

**Figure S11** The relationship between mRNA abundance of 34 genes and OS from GEPIA database. ELFN2 is unavailable due to no records in the database.

**Figure S12** Co-IP assays confirming the interaction between protein AKR1C2 and candidates.

## 3 Supplementary Tables

**Table S1**. Clinical and pathological characteristics of 101 patients with cervical cancer in the training set

| Characteristic | N (%) |
| --- | --- |
| Age, mean (range), years | 53.3 (32-75) |
| Stage |  |
| IB1 | 43 (42.6) |
| IB2 | 11 (10.9) |
| IIA1 | 29 (28.7) |
| IIA2 | 18 (17.8) |
| Tumor diameter, mean (SD), cm | 3.6 (1.4) |
| Histological type |  |
| Squamous cell carcinoma | 92 (91.1) |
| Adenocarcinoma | 6 (5.9) |
| Adenosquamous carcinoma | 3 (3.0) |
| Grade |  |
| G1 | 0 (0.0) |
| G2 | 52 (51.5) |
| G3 | 49 (48.5) |
| Pathological findings |  |
| Stromal invasion depth ˃1/2 |  |
| No | 19 (18.8) |
| Yes | 82 (81.2) |
| LVSI |  |
| No | 76 (75.2) |
| Yes | 25 (24.8) |
| PMI |  |
| No | 98 (97.0) |
| Yes | 3 (3.0) |
| RMI |  |
| No | 99 (98.0) |
| Yes | 2 (2.0) |
| LNM |  |
| No | 77 (76.2) |
| Yes | 24 (23.8) |
| Adjuvant radiotherapy |  |
| No | 29 (28.7) |
| Yes | 72 (71.3) |

LVSI, lymphovascular space invasion; PMI, parametrial involvement; RMI, resection margin involvement; LNM, lymph node metastasis.

**Table S2**. Patients’ baseline characteristics of 101 patients in the training set

| Characteristic | Before matching | | | After matching | | |
| --- | --- | --- | --- | --- | --- | --- |
|  | LNM | NLNM | *p* ^a^ | LNM | NLNM | *p* ^b^ |
| Number (%) | 24 (23.8%) | 77 (76.2%) |  | 18 (50.0%) | 18 (50.0%) |  |
| Age, y |  |  | 0.025 |  |  | 0.416 |
| <50 | 9 (37.5) | 24 (31.2) |  | 6 (33.3) | 5 (27.8) |  |
| 50-59 | 13 (54.2) | 25 (32.5) |  | 10 (55.6) | 7 (38.9) |  |
| ≥60 | 2 (8.3) | 28 (36.4) |  | 2 (11.1) | 6 (33.3) |  |
| Stage |  |  | 0.004 |  |  | 0.826 |
| IB1 | 6 (25.0) | 37 (48.1) |  | 6 (33.3) | 3 (16.7) |  |
| IB2 | 3 (12.5) | 8 (10.4) |  | 2 (11.1) | 6 (33.3) |  |
| IIA1 | 5 (20.8) | 24 (31.2) |  | 3 (16.7) | 6 (33.3) |  |
| IIA2 | 10 (41.7) | 8 (10.4) |  | 7 (38.9) | 3 (16.7) |  |
| Tumor diameter, cm |  |  | 0.001 |  |  | 0.831 |
| ≤2 | 0 (0.0) | 19 (24.7) |  | 0 (0.0) | 1 (5.6) |  |
| 2.1-4 | 11 (45.8) | 42 (54.5) |  | 9 (50.0) | 8 (44.4) |  |
| >4 | 13 (54.2) | 16 (20.8) |  | 9 (50.0) | 9 (50.0) |  |
| Histologic type |  |  | 0.054 |  |  | 0.671 |
| Squamous cell | 19 (79.2) | 73 (94.8) |  | 16 (88.9) | 17 (94.4) |  |
| Adenocarcinoma | 3 (12.5) | 3 (3.9) |  | 2 (11.1) | 1 (5.6) |  |
| Adenosqumous | 2 (8.3) | 1 (1.3) |  | 0 (0.0) | 0 (0.0) |  |
| Grade |  |  | 0.035 |  |  | 0.636 |
| G1 | 0 (0.0) | 0 (0.0) |  | 0 (0.0) | 0 (0.0) |  |
| G2 | 9 (37.5) | 43 (55.8) |  | 7 (38.9) | 9 (50.0) |  |
| G3 | 15 (62.5) | 34 (44.2) |  | 11 (61.1) | 9 (50.0) |  |
| Stromal invasion depth ˃1/2 |  |  | 0.038 |  |  | 0.634 |
| No | 1 (4.2) | 18 (23.4) |  | 0 (0.0) | 1 (5.6) |  |
| Yes | 23 (95.8) | 59 (76.6) |  | 18 (100.0) | 17 (94.4) |  |
| LVSI |  |  | 0.001 |  |  | 0.481 |
| No | 12(50.0) | 64 (83.1) |  | 8 (44.4) | 11 (61.1) |  |
| Yes | 12 (50.0) | 13 (16.9) |  | 10 (55.6) | 7 (38.9) |  |
| PMI |  |  | 0.140 |  |  | 1.000 |
| No | 22 (91.7) | 76 (98.7) |  | 17 (94.4) | 17 (94.4) |  |
| Yes | 2 (8.3) | 1 (1.3) |  | 1 (5.6) | 1 (5.6) |  |
| RMI |  |  | 0.006 |  |  | 0.634 |
| No | 24 (100.0) | 75 (97.4) |  | 18 (100.0) | 17 (94.4) |  |
| Yes | 0 (0.0) | 2 (2.6) |  | 0 (0.0) | 1 (5.6) |  |

LVSI, lymph vascular space invasion; PMI, parametrial involvement; RMI, resection margin involvement; LNM, lymph node metastasis. *p* ^a^ for the χ2 test. *p* ^b^ for likelihood ratio test following conditional logistic regression.

**Table S3**. Ten DEPs with FC > 2.0 in the training set

| Protein ID | Gene | Up or Down (LNM vs. NLNM) | FC (LNM vs. NLNM) | log2FC (LNM vs. NLNM) | *p* |
| --- | --- | --- | --- | --- | --- |
| B4DK69 | AKR1C2 | up | 3.379554241 | 1.756832969 | 0.003633736 |
| A0A5C2GD80 | n/a | up | 2.728614456 | 1.448168561 | 0.014048318 |
| A0A2R8Y430 | GSS | up | 2.426104915 | 1.27864194 | 0.047316165 |
| A0A5C2GYH4 | n/a | up | 2.343135812 | 1.228440577 | 0.029883371 |
| A0A5C2FZE9 | n/a | up | 2.214007378 | 1.14666003 | 0.023813599 |
| H3BRE0 | FBXO22 | up | 2.208616316 | 1.143142814 | 0.00884945 |
| A0A0S2Z508 | ARHGAP26 | up | 2.058973881 | 1.041925529 | 0.005278154 |
| Q8N1N4 | KRT78 | down | 0.36213324 | -1.465407488 | 0.011836014 |
| B4DJM5 | n/a | down | 0.309782694 | -1.690671542 | 0.039208395 |
| A0A1B0GTB0 | ATP6AP2 | down | 0.228165848 | -2.131845232 | 0.043193009 |

DEPs, differentially expressed proteins; FC, fold change; LNM, lymph node metastasis; NLNM, non-lymph node metastasis. The protein code comes from the Uniprot database (https://www.uniprot.org/).

**Table S4**. Univariate and multivariate analyses of risk factors for LNM by binary logistic regression models in the training set

| Risk factors | Univariate Analysis | | Multivariate Analysis | |
| --- | --- | --- | --- | --- |
|  | OR (95% CI) | *p* | aOR (95% CI) | *p* |
| AKR1C2 | 1.484 (1.210-1.819) | <0.001 | 1.544 (1.190-2.005) | 0.001 |
| GSS | 1.073 (0.879-1.310) | 0.490 | - | 0.777 |
| FBXO22 | 1.041 (0.829-1.307) | 0.731 | - | 0.174 |
| ARHGAP26 | 1.180 (1.013-1.375) | 0.033 | 1.496 (1.146-1.954) | 0.003 |
| KRT78 | 1.127 (0.908-1.398) | 0.277 | - | 0.910 |
| ATP6AP2 | 0.776 (0.647-0.929) | 0.006 | 0.504 (0.350-0.725) | <0.001 |

None of the listed covariates had multicollinearity. The Hosmer–Lemeshow test for binary multivariate logistic regression yielded a chi-squared value of 5.233 (*p* = 0.631). Risk factors included 6 differentially expressed proteins. LNM, lymph node metastasis; OR, odds ratio; aOR, adjusted odds ratio; CI, confidence interval.

**Table S5**. Predictive model performance and proteins for LNM in the training set and validation set, respectively

| Characteristics | AUC, 95% CI | Sensitivity, % | Specificity, % | PPV, % | NPV, % |
| --- | --- | --- | --- | --- | --- |
| Training set |  |  |  |  |  |
| AKR1C2 | 0.706 (0.565-0.846) | 58.3 | 89.6 | 63.6 | 87.3 |
| ARHGAP26 | 0.635 (0.508-0.763) | 83.3 | 41.6 | 30.8 | 88.9 |
| ATP6AP2 | 0.681 (0.573-0.790) | 75.0 | 58.4 | 36.0 | 88.2 |
| Model a | 0.890 (0.824-0.955) | 95.8 | 72.7 | 52.3 | 98.2 |
| Model b | 0.920 (0.865-0.975) | 91.7 | 84.4 | 64.7 | 97.0 |
| Validation set |  |  |  |  |  |
| AKR1C2 | 0.714 (0.599-0.830) | 55.2 | 87.7 | 47.1 | 90.8 |
| ARHGAP26 | 0.633 (0.527-0.739) | 89.7 | 36.3 | 21.8 | 94.6 |
| ATP6AP2 | 0.630 (0.530-0.730) | 75.9 | 50.7 | 23.4 | 91.4 |
| Model a | 0.861 (0.801-0.920) | 93.1 | 66.4 | 35.5 | 98.0 |

LNM, lymph node metastasis; AUC, area under the receiver operating characteristic curve; PPV, positive predictive value; NPV, negative predictive value. Model a consists of three proteins, and Model b consists of three proteins and two clinical variables.

**Table S6**. Univariate and multivariate analyses of risk factors for LNM by binary logistic regression models in the training set

| Risk factors | Univariate Analysis | | Multivariate Analysis | |
| --- | --- | --- | --- | --- |
|  | OR (95% CI) | *p* | aOR (95% CI) | *p* |
| AKR1C2 | 1.484 (1.210-1.819) | <0.001 | 1.764 (1.241-2.508) | 0.002 |
| GSS | 1.073 (0.879-1.310) | 0.490 | - | 0.179 |
| FBXO22 | 1.041 (0.829-1.307) | 0.731 | - | 0.115 |
| ARHGAP26 | 1.180 (1.013-1.375) | 0.033 | 1.694 (1.131-2.536) | 0.011 |
| KRT78 | 1.127 (0.908-1.398) | 0.277 | - | 0.590 |
| ATP6AP2 | 0.776 (0.647-0.929) | 0.006 | 0.399 (0.219-0.725) | 0.003 |
| Age | 0.977 (0.935-1.022) | 0.320 | - | 0.172 |
| Stage | 1.440 (1.073-1.933) | 0.015 | - | 0.275 |
| Tumor diameter | 2.159 (1.442-3.232) | <0.001 | 3.783 (1.625-8.809) | 0.002 |
| Histological type | 3.106 (1.089-8.860) | 0.034 | 29.280 (1.667-514.282) | 0.021 |
| Grade | 1.265 (0.613-2.609) | 0.525 | - | 0.075 |

None of the listed covariates had multicollinearity. The Hosmer–Lemeshow test for binary multivariate logistic regression yielded a chi-squared value of 4.334 (*p* = 0.826). Risk factors included 6 differentially expressed proteins and 5 preoperative clinical variables. LNM, lymph node metastasis; OR, odds ratio; aOR, adjusted odds ratio; CI, confidence interval.

**Table S7**. Univariate and multivariate analyses of prognostic factors for 5-year DFS and OS by Cox proportional hazards regression models in the training set

| Prognostic factor | 5-year DFS | | | | 5-year OS | | | |
| --- | --- | --- | --- | --- | --- | --- | --- | --- |
|  | Univariate Analysis | | Multivariate Analysis | | Univariate Analysis | | Multivariate Analysis | |
|  | HR (95% CI) | *p* | aHR (95% CI) | *p* | HR (95% CI) | *p* | aHR (95% CI) | *p* |
| AKR1C2 >4 | 3.359 (1.414–7.981) | 0.006 | 3.359 (1.414–7.981) | 0.006 | 4.369 (1.733–11.017) | 0.002 | 4.369 (1.733–11.017) | 0.002 |
| GSS >8 | 2.053 (0.478–8.820) | 0.333 | - | 0.524 | 2.265 (0.521–9.854) | 0.276 | - | 0.506 |
| FBXO22 >3 | 1.887 (0.439–8.105) | 0.393 | - | 0.455 | 1.603 (0.368–6.978) | 0.529 | - | 0.644 |
| ARHGAP26 >4 | 2.447(0.823–7.273) | 0.107 | - | 0.268 | 2.796 (0.809–9.658) | 0.104 | - | 0.311 |
| KRT78 >8 | 3.429 (0.459–25.640) | 0.230 | - | 0.715 | 3.678 (0.488–27.733) | 0.206 | - | 0.772 |
| ATP6AP2 ≤4 | 0.575 (0.238–1.389) | 0.219 | - | 0.288 | 0.615 (0.238–1.587) | 0.315 | - | 0.368 |

Cox proportional hazards regression models for multivariable analysis. All listed covariates were not found to have multicollinearity. Prognostic factors included 6 differentially expressed proteins. HR, hazard ratio; aHR, adjusted hazard ratio; CI, confidence interval.

**Table S8**. Univariate and multivariate analyses of prognostic factors for 5-year DFS and OS by Cox proportional hazards regression models in the training set

| Prognostic factor | 5-year DFS | | | | 5-year OS | | | |
| --- | --- | --- | --- | --- | --- | --- | --- | --- |
|  | Univariate Analysis | | Multivariate Analysis | | Univariate Analysis | | Multivariate Analysis | |
|  | HR (95% CI) | *p* | aHR (95% CI) | *p* | HR (95% CI) | *p* | aHR (95% CI) | *p* |
| AKR1C2 >4 | 3.359 (1.414–7.981) | 0.006 | 2.726 (1.137–6.537) | 0.025 | 4.369 (1.733–11.017) | 0.002 | 3.558 (1.397–9.066) | 0.008 |
| GSS >8 | 2.053 (0.478–8.820) | 0.333 | - | 0.355 | 2.265 (0.521–9.854) | 0.276 | - | 0.398 |
| FBXO22 >3 | 1.887 (0.439–8.105) | 0.393 | - | 0.618 | 1.603 (0.368–6.978) | 0.529 | - | 0.951 |
| ARHGAP26 >4 | 2.447(0.823–7.273) | 0.107 | - | 0.234 | 2.796 (0.809–9.658) | 0.104 | - | 0.242 |
| KRT78 >8 | 3.429 (0.459–25.640) | 0.230 | - | 0.568 | 3.678 (0.488–27.733) | 0.206 | - | 0.640 |
| ATP6AP2 ≤4 | 0.575 (0.238–1.389) | 0.219 | - | 0.899 | 0.615 (0.238–1.587) | 0.315 | - | 0.998 |
| Age | 0.665 (0.378–1.167) | 0.155 | - | 0.276 | 0.745 (0.408–1.361) | 0.338 | - | 0.530 |
| Stage | 1.242 (0.859–1.797) | 0.249 | - | 0.140 | 1.357 (0.905–2.035) | 0.140 | - | 0.495 |
| Tumor diameter | 4.587 (2.067–10.182) | <0.001 | 4.163 (1.877–9.231) | <0.001 | 4.257 (1.811–10.006) | 0.001 | 3.714 (1.585–8.702) | 0.003 |
| Histological type | 1.139 (0.443–2.929) | 0.788 | - | 0.832 | 1.288 (0.500–3.317) | 0.600 | - | 0.654 |
| Grade | 1.007 (0.428–2.372) | 0.987 | - | 0.402 | 0.872 (0.344–2.212) | 0.774 | - | 0.225 |

Cox proportional hazards regression models for multivariable analysis. All listed covariates were not found to have multicollinearity. Prognostic factors included 6 differentially expressed proteins and 5 preoperative clinical variables. HR, hazard ratio; aHR, adjusted hazard ratio; CI, confidence interval.

**Table S9**. Clinical and pathological characteristics of 175 patients with cervical cancer in the validation set

| Characteristic | N (%) |
| --- | --- |
| Age, mean (range), years | 48.0 (28-79) |
| Stage |  |
| IB1 | 114 (65.1) |
| IB2 | 20 (11.4) |
| IIA1 | 30 (17.1) |
| IIA2 | 11 (6.3) |
| Tumor diameter, mean (SD), cm | 2.9 (1.2) |
| Histological type |  |
| Squamous cell carcinoma | 158 (90.3) |
| Adenocarcinoma | 16 (9.1) |
| Adenosquamous carcinoma | 1 (0.6) |
| Grade |  |
| G1 | 5 (2.9) |
| G2 | 84 (48.0) |
| G3 | 86 (49.1) |
| Pathological findings |  |
| Stromal invasion depth ˃1/2 |  |
| No | 76 (43.4) |
| Yes | 99 (56.6) |
| LVSI |  |
| No | 147 (84.0) |
| Yes | 28 (16.0) |
| PMI |  |
| No | 175 (100.0) |
| Yes | 0 (0.0) |
| RMI |  |
| No | 174 (99.4) |
| Yes | 1 (0.6) |
| LNM |  |
| No | 146 (83.4) |
| Yes | 29 (16.6) |
| Adjuvant radiotherapy |  |
| No | 51 (29.1) |
| Yes | 124 (70.9) |

LVSI, lymphovascular space invasion; PMI, parametrial involvement; RMI, resection margin involvement; LNM, lymph node metastasis.

**Table S10**. Univariate and multivariate analyses of risk factors for LNM by binary logistic regression models in the validation set

| Risk factors | Univariate Analysis | | Multivariate Analysis | |
| --- | --- | --- | --- | --- |
|  | OR (95% CI) | *p* | aOR (95% CI) | *p* |
| AKR1C2 | 1.361 (1.166-1.589) | <0.001 | 1.458 (1.225-1.734) | <0.001 |
| ARHGAP26 | 1.212 (1.060-1.386) | 0.005 | 1.392 (1.164-1.666) | <0.001 |
| ATP6AP2 | 0.846 (0.757-0.946) | 0.003 | 0.728 (0.624-0.848) | <0.001 |

None of the listed covariates had multicollinearity. The Hosmer–Lemeshow test for binary multivariate logistic regression yielded a chi-squared value of 3.462 (*p* = 0.839). Risk factors included 3 differentially expressed proteins. LNM, lymph node metastasis; OR, odds ratio; aOR, adjusted odds ratio; CI, confidence interval.

**Table S11**. Univariate and multivariate analyses of risk factors for LNM by binary logistic regression models in the validation set

| Risk factors | Univariate Analysis | | Multivariate Analysis | |
| --- | --- | --- | --- | --- |
|  | OR (95% CI) | *p* | aOR (95% CI) | *p* |
| AKR1C2 | 1.361 (1.166-1.589) | <0.001 | 1.475 (1.229-1.770) | <0.001 |
| ARHGAP26 | 1.212 (1.060-1.386) | 0.005 | 1.445 (1.194-1.748) | <0.001 |
| ATP6AP2 | 0.846 (0.757-0.946) | 0.003 | 0.689 (0.578-0.821) | <0.001 |
| Age | 0.520 (0.262-1.032) | 0.062 | 0.340 (0.140-0.826) | 0.017 |
| Stage | 0.968 (0.640-1.466) | 0.880 | - | 0.990 |
| Tumor diameter | 1.146 (0.633-2.074) | 0.653 | - | 0.663 |
| Histological type | 1.436 (0.472-4.363) | 0.524 | - | 0.785 |
| Grade | 1.934 (0.890-4.204) | 0.096 | - | 0.076 |

None of the listed covariates had multicollinearity. The Hosmer–Lemeshow test for binary multivariate logistic regression yielded a chi-squared value of 6.704 (*p* = 0.569). Risk factors included 3 differentially expressed proteins and 5 preoperative clinical variables. LNM, lymph node metastasis; OR, odds ratio; aOR, adjusted odds ratio; CI, confidence interval.

**Table S12**. Univariate and multivariate analyses of prognostic factors for 5-year DFS and OS by Cox proportional hazards regression models in the validation set

| Prognostic factor | 5-year DFS | | | | 5-year OS | | | |
| --- | --- | --- | --- | --- | --- | --- | --- | --- |
|  | Univariate Analysis | | Multivariate Analysis | | Univariate Analysis | | Multivariate Analysis | |
|  | HR (95% CI) | *p* | aHR (95% CI) | *p* | HR (95% CI) | *p* | aHR (95% CI) | *p* |
| AKR1C2 >4 | 3.122 (1.208–8.068) | 0.019 | 3.122 (1.208–8.068) | 0.019 | 3.827 (1.206–12.143) | 0.023 | 3.827 (1.206–12.143) | 0.023 |
| ARHGAP26 >4 | 2.313(0.669–8.001) | 0.185 | - | 0.173 | 2.183 (0.477–9.999) | 0.315 | - | 0.331 |
| ATP6AP2 ≤4 | 0.587 (0.220–1.565) | 0.287 | - | 0.428 | 0.816 (0.259–2.575) | 0.729 | - | 0.998 |

Cox proportional hazards regression models for multivariable analysis. All listed covariates were not found to have multicollinearity. Prognostic factors included 3 differentially expressed proteins. HR, hazard ratio; aHR, adjusted hazard ratio; CI, confidence interval.

**Table S13**. Univariate and multivariate analyses of prognostic factors for 5-year DFS and OS by Cox proportional hazards regression models in the validation set

| Prognostic factor | 5-year DFS | | | | 5-year OS | | | |
| --- | --- | --- | --- | --- | --- | --- | --- | --- |
|  | Univariate Analysis | | Multivariate Analysis | | Univariate Analysis | | Multivariate Analysis | |
|  | HR (95% CI) | *p* | aHR (95% CI) | *p* | HR (95% CI) | *p* | aHR (95% CI) | *p* |
| AKR1C2 >4 | 3.122 (1.208–8.068) | 0.019 | 3.122 (1.208–8.068) | 0.019 | 3.827 (1.206–12.143) | 0.023 | 3.827 (1.206–12.143) | 0.023 |
| ARHGAP26 >4 | 2.313(0.669–8.001) | 0.185 | - | 0.173 | 2.183 (0.477–9.999) | 0.315 | - | 0.331 |
| ATP6AP2 ≤4 | 0.587 (0.220–1.565) | 0.287 | - | 0.428 | 0.816 (0.259–2.575) | 0.729 | - | 0.998 |
| Age | 0.912 (0.473–1.761) | 0.785 | - | 0.923 | 1.084 (0.506–2.319) | 0.836 | - | 0.661 |
| Stage | 1.222 (0.798–1.870) | 0.357 | - | 0.364 | 1.215 (0.723–2.040) | 0.462 | - | 0.479 |
| Tumor diameter | 1.291 (0.652–2.558) | 0.464 | - | 0.494 | 1.479 (0.633–3.457) | 0.366 | - | 0.443 |
| Histological type | 0.052 (0.000–24.513) | 0.346 | - | 0.116 | 0.053 (0.000–97.065) | 0.444 | - | 0.210 |
| Grade | 1.058 (0.452–2.479) | 0.896 | - | 0.845 | 0.984 (0.343–2.825) | 0.977 | - | 0.707 |

Cox proportional hazards regression models for multivariable analysis. All listed covariates were not found to have multicollinearity. Prognostic factors included 3 differentially expressed proteins and 5 preoperative clinical variables. HR, hazard ratio; aHR, adjusted hazard ratio; CI, confidence interval.
